# Diastereoselective, Diversifiable Synthesis and Biological Evaluation of the Virginiamycin Inducers, the Virginiae Butanolides

**DOI:** 10.1002/cbic.202500386

**Published:** 2025-08-14

**Authors:** Kylie G. Castator, Manuela Frias‐Gomez, Lauren E. Wilbanks, Elizabeth I. Parkinson

**Affiliations:** ^1^ James Tarpo Jr. and Margaret Tarpo Department of Chemistry Purdue University West Lafayette IN 47907 USA; ^2^ Borch Department of Medicinal Chemistry and Molecular Pharmacology Purdue University West Lafayette IN 47907 USA

**Keywords:** natural products, quorum sensing, tetR repressors

## Abstract

*Streptomyces* species are renowned for their ability to produce bioactive natural products (NPs) via biosynthetic gene clusters (BGCs). However, many BGCs remain transcriptionally silent under standard laboratory conditions. Among the key regulatory mechanisms for NP biosynthesis are the *γ*‐butyrolactone (GBL) signaling molecules, which have been widely studied for their role in repressor‐molecule circuits. While the *S. coelicolor* butanolides (SCBs) and A‐factor from *S. griseus* have been extensively studied, the virginiae butanolides (VBs) from *S. virginiae,* which alleviate repression of the biosynthesis of the antibiotic virginiamycins via binding to the cluster situated TetR‐like repressor BarA, remain understudied. This is in large part due to limited access to enantiopure VBs. Herein, we report a diastereoselective and diversifiable route to access the VB hormones, starting from a protected (*R*)‐paraconyl alcohol intermediate. A library of VB derivatives was synthesized and tested for their ability to alleviate repression of BarA using a newly developed green fluorescent protein (GFP) reporter assay. The synthesis and assay described herein established the most quantitative structure–activity relationship (SAR) analysis of the VBs to date. Overall, this study provides new tools for probing NP regulation in *Streptomyces* and enables new strategies for BGC activation using synthetic GBL molecules.

## Graphical abstract

Production of natural products is tightly regulated, resulting in many silent biosynthetic gene clusters. BarA is a repressor that downregulates production of the virginiamycin antibiotics. The VBs are ligands for BarA that reverse its repression of virginiamycin production. Herein, we report a diversifiable synthesis of the VBs and a GFP‐assay to rapidly assess their abilities to induce natural product production.

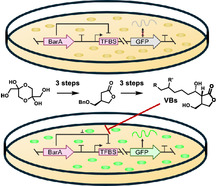

## 1 Introduction

**Figure 1:**
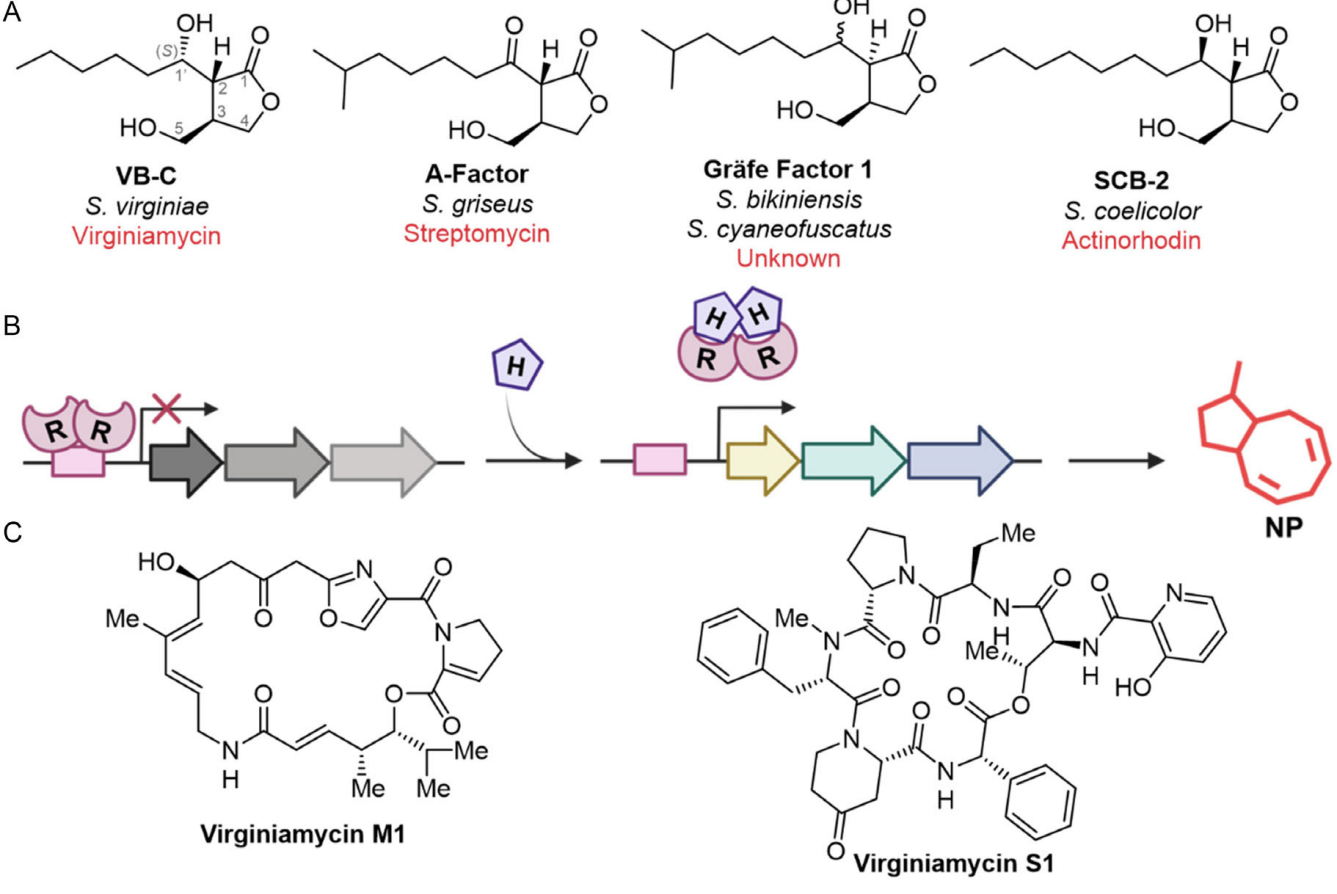
*γ*‐butyrolactone signaling molecules and regulation of biosynthetic gene clusters (BGCs) in *Streptomyces*. A) Representative chemical structures of *γ*‐butyrolactones from different *Streptomyces* species. B) Schematic overview of biosynthetic gene cluster (BGC) regulation mediated by a transcriptional regulator. Transcriptional regulator (R) represses downstream gene expression, but in the presence of a chemical inducer (H), repression is alleviated, enabling NP production. C) Chemical structures of the BarA‐regulated NPs in *S. virginiae*: Virginiamycin M1 and S1.

Natural products (NPs) from the soil‐dwelling bacteria *Streptomyces* have been an essential source of leads for drug discovery and agricultural applications. These NPs exhibit a wide variety of activities, including antimicrobial, immunosuppressant, and anticancer.^[^ ^1^ ^]^ Genome sequencing has revealed that *Streptomyces* contain a large number of cryptic (i.e. uncharacterized) biosynthetic gene clusters (BGCs), suggesting that there are still a large number of NPs to be found within this genus.^[^ 2, 3, 4 ^]^ Many of these BGCs are ‘silent’ or not expressed at detectable levels under standard laboratory conditions, making isolation of these NPs extremely challenging.^[^ ^5^ ^,^ ^6^ ^]^ Many approaches have been explored to access NPs from these silent BGCs, including deletion of repressors, promoter swapping, heterologous expression, and induction with small molecule stressors.^[^ ^5^ ^,^ ^7^ ^]^ One area that is relatively underexplored is using regulatory molecules that control NP transcription. One of the largest classes of such molecules in *Streptomyces* are the quorum sensing *γ*‐butyrolactones (GBLs), which bind to cluster situated TetR‐like repressors and alleviate repression of transcription of the NP's BGCs (**Figure** **1**).^[^ 7, 8, 9, 10 ^]^

**Scheme 1:**
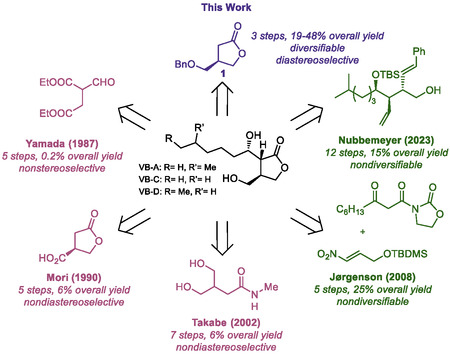
Synthetic approaches to access the VB‐type molecules.

Four main families of GBLs exist: *virginiae* but anolides (VBs), A‐factor, Gräfe factors, and *Streptomyces coelicolor* butanolides (SCBs) (Figure 1A).^[^ ^8^ ^]^ These families differ based on the oxidation state and stereochemistry of the C1′ carbon. GBLs have been found to upregulate specific NPs through binding their cognate repressor. For example, A‐factor regulates the antituberculosis therapeutic streptomycin, a World Health Organization essential medicine. Despite their essential role in regulating these important NPs, surprisingly little has been done using GBLs to chemically induce the production of NPs in wild‐type *Streptomyces* strains, likely because of the challenge of accessing the GBLs. Generally, GBLs are produced at very low titers (0.06—1.1 µg/L for VBs),^[^ ^11^ ^,^ ^12^ ^]^ and the synthesis of enantiopure molecules can be quite challenging. While there have been significant investigations into structure activity relationships (SARs) of the A‐factor and SCB type GBLs,^[^ ^13^ ^–^ ^15^ ^]^ exploration of the VBs has been more limited.^[^ ^12^ ^,^ ^16^ ^]^ The VBs are known to bind the TetR‐like repressor BarA and alleviate its repression of production of virginiamycin,^[^ ^17^ ^]^ a veterinary antibiotic consisting of two NPs *M*_I_ and *S*_I_ that act synergistically to inhibit the 50S ribosomal subunit (Figure 1C).^[^ ^18^ ^]^ Commercial virginiamycin is currently produced by large‐scale fermentation, but the generation of high yielding strains has been challenging.^[^ ^18^ ^,^ ^19^ ^]^ Interestingly, addition of chemically synthesized VB‐C has been shown to induce production of virginiamycin *M*_I_ and *S*_I_ between two and nine‐fold, depending on culture conditions.^[^ 20, 21, 22, 23 ^]^ While this is a promising result, these studies used racemic molecule. Other studies have shown that having enantiopure GBLs is important for getting optimal induction of other NPs.^[^ ^24^ ^]^ Although others have qualitatively explored the ability of racemic VB derivatives to induce virginiamycin production,^[^ ^16^ ^]^ no quantitative analyses exploring the ability of these derivatives to derepress BarA have been performed. This is despite the fact that BarA has clearly been demonstrated to repress virginiamycin production, with mutant strains lacking BarA producing virginiamycin hours earlier than wild type strains.^[^ ^25^ ^,^ ^26^ ^]^ Previous SARs enabled initial hypotheses on necessary properties for derepression but generally relied on low‐throughput zone of inhibition studies of *S. virginiae* supernatant after exposure to VBs. Although these assays gave a great initial understanding of the activities of VBs, more quantitative data is needed for applications of these molecules both in fermentation and synthetic biology. While syntheses of the enantio‐ and diastereomerically pure VBs have since been reported,^[^ ^11^ ^,^ 27, 28, 29, 30 ^]^ routes were either long or not easily diversifiable, hindering the SAR studies of enantiopure derivatives (**Scheme** **1**).

Described herein, we have developed an easily diversifiable route to the VBs. Specifically, this efficient route depends on generation of a key common intermediate, benzyl protected (*R*)‐paraconyl alcohol (**1**), which can be derivatized via acylation and stereoselectively reduced using a Noyori hydrogenation to give stereochemically pure VBs. Access to enantiomerically and diastereomerically pure derivatives, combined with a BarA reporter assay, enabled the rapid determination the most active VB‐type compounds. Long‐term, this work will enable improved production of NPs including virginiamycin by leveraging the application of VB‐type molecules in fermentation on industrial scale.

## 2 Synthesis of VBs

### 2.1 Previous Routes to VBs

Previous routes to the VBs have taken one of two approaches: 1) generation of a paraconyl alcohol intermediate followed by derivatization (e.g. Yamada, Mori, and Takabe, Scheme 1, mauve)^[^ ^11^ ^,^ ^29^ ^,^ ^30^ ^]^ or 2) initial sidechain installation followed by enantioselective generation of the butyrolactone ring (e.g. Jørgenson and Nubbemeyer, Scheme1, green).^[^ ^27^ ^,^ ^28^ ^]^ Yamada first synthesized racemic VB‐C by accessing a racemic common intermediate in two steps from diethyl formylsuccinate.^[^ ^11^ ^]^ While this allowed quick access to VB‐C, the synthesis was generally low yielding and lacked any asymmetric reactions. Mori and Takabe both accessed the natural VB‐type molecules from enzymatically generated chiral precursors to the common (*R*)‐paraconyl alcohol intermediate.^[^ ^29^ ^–^ ^31^ ^]^ Unfortunately, both syntheses lacked diasterelective reductions of the exocyclic (C1′) ketone and were generally low yielding. Jørgenson accessed VB‐D in five steps by first utilizing a chiral organocatalyzed 1,4 addition followed by lactonization.^[^ ^28^ ^]^ After three additional steps, they were able to diastereoselectively reduce the exocyclic ketone and transform the nitro group into the hydroxy lactone. Although they were the first to employ an asymmetric reduction and had good yields, beginning the route with the aliphatic side chain means this route is not easily diversifiable and thus is not amenable to accessing a library of these compounds. In 2023, a novel route to VB‐A was developed by Nubbemeyer and coworkers, in which they accessed VB‐A stereoselectively in 12 steps from an unsaturated carbonyl starting material.^[^ ^27^ ^]^ While this route was generally high yielding, similar to the Jørgenson route, it is not amenable to diversification.

### 2.2 Accessing the Enantiopure Common Intermediate 1

When designing our synthetic route, we aimed for a diversifiable and stereoselective synthesis. To do this, we sought to use an enantioenriched intermediate that could then undergo acylation followed by stereospecific reduction. We previously accessed the TBS protected (*R*)‐paraconyl alcohol with high yields and enantioselectivity (95%).^[^ ^14^ ^]^ In route to the VBs, we were successfully able to acylate the TBS‐protected intermediate with a large variety of sidechains; however, attempts at utilizing a CBS reduction to access the exocyclic hydroxyl group failed to provide adequate diastereomeric ratios (dr) and the Noyori hydrogenation conditions as reported by Jørgenson only resulted in the loss of the silyl protecting group.^[^ ^14^ ^]^ Additionally, utilizing a chiral ruthenium catalyst under atmospheric pressure proved unfruitful.^[^ ^32^ ^]^

**Scheme 2:**
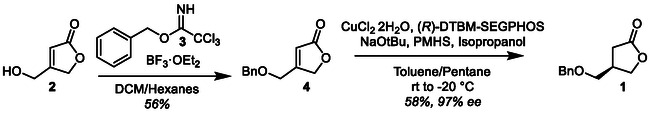
Synthesis of benzyl protected (*R*)‐paraconyl alcohol (**1**).

Due to the many undesirable results when utilizing the silane protecting group, we decided to employ the use of the benzyl protecting group. The benzyl protected paraconyl alcohol (1, **Scheme** **2**) has previously been accessed by Takabe and coworkers, via a lipase catalyzed acylation and three rounds of kinetic resolution of *N, N*‐dialkylbutanamide in a low yield of 21% but impressive enantioselectivity (99% ee).^[^ ^30^ ^]^ In route to synthesize Factor‐I, an autoregulator isolated from *S. viridocchromogenes*, the enantiomer of this common intermediate, benzyl protected (*S*)‐paraconyl alcohol was accessed via a bakers’ yeast reduction of 3‐benzyloxymethylbutenolide in a good enantioselectivity (95% ee) but still a generally low yield (34% yield).^[^ ^33^ ^]^ We aimed to improve access to the benzyl protected paraconyl alcohol. As previously described, we obtained the unprotected butenolide (**2**) via a Wittig olefination and subjected it to benzyl protection under slightly acidic conditions using benzyltrichloroacedimidate (**3**) and boron trifloride diethyletherate to give the benzyl protected butenolide (**4**). This reaction proceeded in good yield (56% yield). We initially attempted the same asymmetric hydrogenolysis reaction on **3** that was previously utilized with the TBS protected butenolide.^[^ ^14^ ^]^ While we were able to enantioselectivity access **1** in 52% yield and 88% ee using the previously reported (*R*)‐tol‐BINAP ligand, we found that switching the to (*R*)‐DTBM‐SEGPHOS resulted in higher yields (58%) and enantioselectivities (97% ee). These results are similar to those recently observed for the TBS protected butenolide.^[^ ^34^ ^]^

**Scheme 3:**
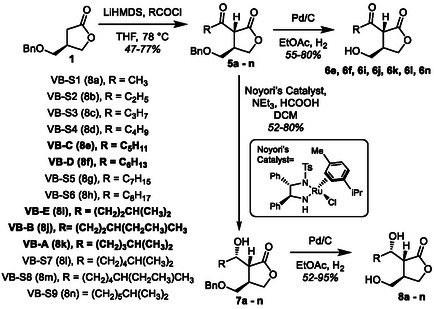
Access to the VB‐type molecules. Bold side chains indicate natural VB side chains.

### 2.3 Accessing VB Molecules

With **1** in hand, we were able to access the protected A‐factor derivatives of the each of the natural VB‐type molecules as well as synthetic derivatives (**5a‐n,** **Scheme** **3**). Acylation of each sidechain was carried out with their respective acyl‐chloride and LiHMDS to give yields between 47 and 77%. It was found that using LiHMDS in replacement of NaHMDS led to higher yields.^[^ ^14^ ^,^ ^35^ ^]^ Single diastereomers were then isolated via column chromatography. Benzyl deprotection of the of the acylation products enabled access to A‐factor and a handful of A‐factor type derivatives (**6**).

**Scheme 4:**
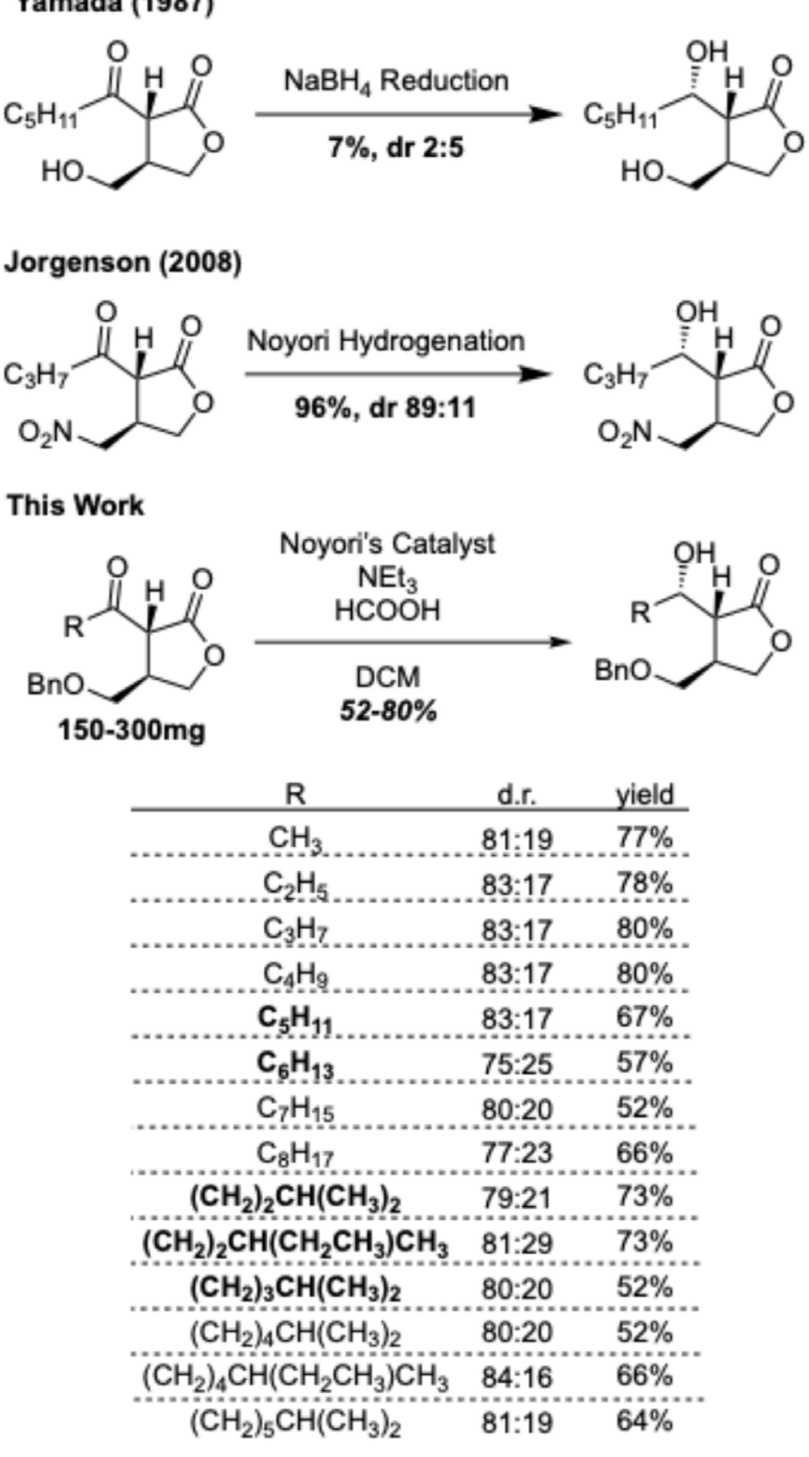
Synthetic approaches C1’ reduction.

Next, we aimed to diastereoselectively reduce the exocyclic carbonyl. Many previous attempts have been unsuccessful at the late‐stage installation of this stereocenter in good diastereomeric ratios and yields. During the first synthesis of the VBs, Yamada performed a sodium borohydridride reduction of keto VB‐C.^[^ ^11^ ^]^ Unfortunately, the VB‐type molecules were a minor product, with the reduction instead favoring the stereochemistry associated with the SCB‐type molecules (2:5 VB:SCB). Mori had similar results when they utilized sodium borohydride to access both the VB‐type and Gräfe factor type molecules, with yields ranging from 22 to 38%.^[^ ^30^ ^]^ It should be noted that the two aforementioned publications came before the corrected stereochemical assignment of these molecules made by Yamada in 1991, wherein NOE experiments confirmed the absolute configuration of VBs to be (2*R*,3*R*,1′*S*), correcting the previously reported configuration of (2*R*,3*S*,1′*R*).^[^ ^36^ ^]^ Based on this data, few attempts have since been made at the diastereoselective synthesis of the VBs.^[^ ^27^ ^,^ ^28^ ^]^ Our asymmetric reduction was inspired by the success of the Noyori Hydrogenation on a similar substrate, carried out by Jørgenson and coworkers (**Scheme** **4**).^[^ ^28^ ^]^ We were able to apply Noyori's hydrogenation conditions to the benzyl protected **1** resulting in good stereoselectivies (dr 77:23‐84:16) and yields (52–80% yield of the desired diastereomer, **7a‐n**). After this reduction, deprotection with hydrogen gas and palladium on carbon resulted in the final VB‐type molecules in good yield (52% to 94%, **8a‐n**). With the VBs in hand, we also wanted to explore the structure of the Gräfe factors. Others have previously suggested that the original structure of the Gräfe factors was misreported. Specifically, it has been suggested that the alpha and beta protons on the lactone ring are trans, not the originally reported cis (Scheme 1). If this were the case, VB‐A and Gräfe factor 1 would be the same structure. Herein, we have directly compared the NMRs from synthetic VB‐A with the previously published NMR spectra for Gräfe factor 1 (Figure S6 and Table S7, Supporting Information). These spectra aligned nearly perfectly, providing very strong support for the misassignment of the original Gräfe factor 1 structure. Overall, we were able to access 14 enantio‐ and diastereopure VB‐type molecules in moderate to good overall yields (19–48% from **1**). Additionally, this route is highly diversifiable, enabling rapid synthesis of additional derivatives.

### 2.4 BarA GFP Reporter Assay

With a rapid method for generating the VBs and their derivatives in enantio‐ and diastereopure forms, we wished to explore the ability of these molecules to derepress BarA, the cognate repressor of the VBs that normally regulates production of virginiamycin in *S. virginiae*. Previous studies by Kinoshita et al. used Electrophoretic Mobility Shift Assay (EMSA) to demonstrate that natural VB‐type hormones disrupt BarA‐DNA binding in a dose‐dependent manner.^[^ ^17^ ^]^ Nihira et al. evaluated racemic analogs of VB‐C using a clear‐zone assay with *Bacillus subtilis* as an indicator to investigate their SAR in inducing virginiamycin production.^[^ ^16^ ^]^ While both of these assays provided foundational data and insights, they yield semiquantitative results and may not be ideally suited for efficiently screening numerous analogs or precisely quantifying their activities. Fluorescence‐based reporter assays have been successfully adopted in various genetic circuits, including in quantitative SAR analyses of related hormone/repressor system.^[^ ^14^ ^,^ 37, 38, 39, 40 ^]^ They offer a rapid, sensitive, and more quantitative method to assess binding affinity, allowing for an efficient screening of a broader range of molecules.

**Figure 2:**
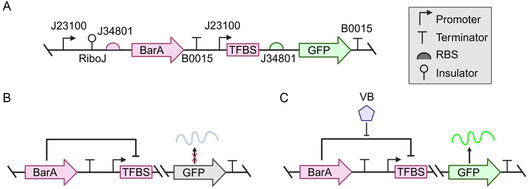
Schematic overview of GFP‐biosensor reporter plasmid (pTOTAL‐barA) . A) Detailed plasmid construct showing BioBrick parts and IDs (e.g., B0015).^[^ ^49^ ^]^ B) Without inducer, the transcriptional regulator protein (BarA) binds to its transcription factor binding site (TFBS), repressing expression of reporter protein (GFP). C) In the presence of a chemical inducer (VB), BarA undergoes a conformational change, dissociating from DNA, leading to GFP expression.

**Figure 3:**
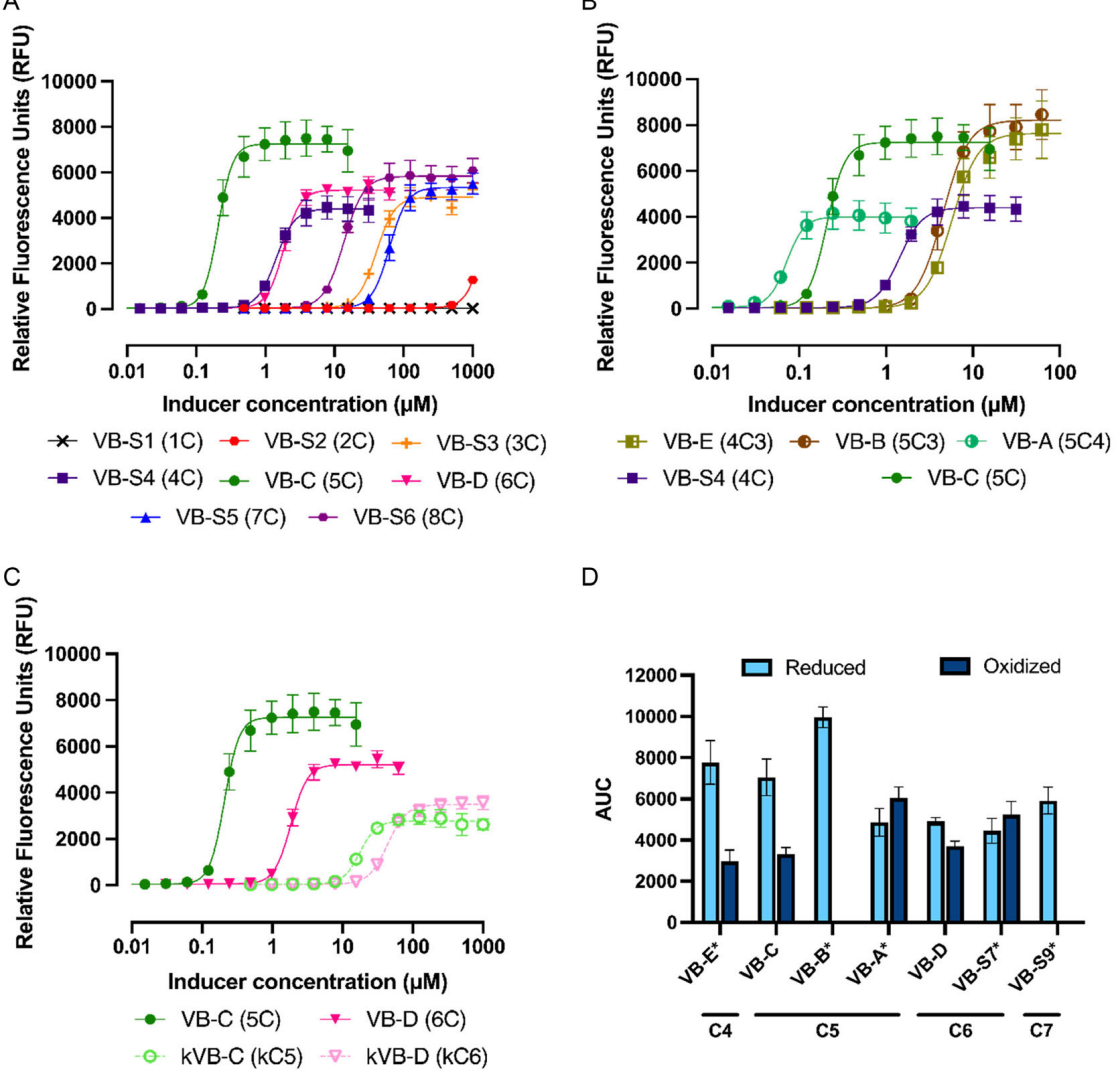
Evaluation of VB‐type hormones activity using plasmid‐based GFP reporter assay. The notation in parentheses after the compound names in this figure (e.g., 4C) refers to the length of the carbon chain extending from the exocyclic hydroxyl group, with the number indicating the number of carbon atoms. If additional numbers are included (e.g., 4C3), they indicate the position of a methyl‐branch along that chain. Oxidized forms of the hormones are denoted with a lower‐case k. A) Hormones with varying chain lengths, B) differing branching patters, and C) oxidized versus reduced forms. Data shown are averages of at least 3 biological replicates, with error bars indicating the standard error of the mean. D) Area under the curve (AUC) comparison for reduced (alcohol, light blue) and oxidized (ketone, dark blue) VB‐type and Gräfe factor hormones across different chain lengths (C4–C7). Asterisks (*) denote hormones containing branched side chains. Missing AUC values (VB‐B and VB‐S9) indicate incomplete dose–response curves, even at highest tested concentrations. Full dose–response curves, dissociation constants (*K*_D_), maximum response (*E*_max_), and Hillslope for all hormones can be found in the Figure S2 and Table S1, Supporting Information**.**

Building upon our previously published plasmid‐based ScbR GFP reporter assay in *E. coli*, in which the repressor ScbR from *S. coelicolor* was tested against the SCB‐type molecules,^[^ ^14^ ^]^ we constructed a BarA vector containing both the *barA* gene downstream of a J23100 promoter and the *gfp* gene downstream of a previously identified BarA TFBS^[^ ^41^ ^]^ (**Figure** **2**). We chose to modify the vector compared to the original plasmid to include the genetic insulator RiboJ^[^ ^42^ ^]^ downstream of the *barA* promoter. The addition of RiboJ reduced leaky GFP expression by increasing BarA protein expression. The *gfp* promoter was changed to a strong *E. coli* ‐10/−35 sequence (J23100) with the BarA TFBS downstream, affording high GFP expression upon BarA derepression. The resulting vector had a baseline fluorescence of 32 RFU and a maximum induced fluorescence of 9235 RFU, a 293‐fold change over baseline. This assay enabled the determination of binding parameters including the dissociation constant (*K*_D_), maximal response (*E*_max_), Hill coefficient, and area under the curve (AUC) for each of the 21 molecules tested (**Figure** **3**, Figure S2, and Table S1, Supporting Information). We chose to particularly focus on the AUC, which integrates the information from the *K*_D_, *E*_max_ and Hill coefficient values, providing a comprehensive assessment of ligand potency and efficacy.^[^ ^38^ ^,^ ^43^ ^]^

We first chose to investigate the effects of sidechain length by investigating different straight chain VB‐type molecules (Figure 3A). The natural straight chain VBs include VB‐C and VB‐D. VB‐C stands out across all parameters as it exhibited the highest *E*_max_, greatest fold change, strongest binding affinity, and largest AUC of all the straight chain VBs. In contrast, VB‐D exhibited potent binding affinity, but its overall activity was notably lower, which is reflected by its reduced AUC compared to VB‐C. Investigation of the unnatural VB derivatives revealed that short‐chain derivatives (one or two carbons following the exocyclic hydroxyl, VB‐S1 and VB‐S2, respectively) showed minimal or no activity, clearly defining a three‐carbon length as the minimal requirement for significant BarA interaction and GFP induction. This threshold aligns with SAR reported by Nihira et al. who similarly identified the three‐carbon derivative as the minimum necessary to observe biological activity.^[^ ^16^ ^]^ However, we observed a deviation in the SAR, where VB‐S3 exhibited slightly improved activity compared to VB‐S4. Specifically, VB‐S3 had a higher *E*_max_ and AUC, but VB‐S4 had a more potent binding affinity. These subtle differences are not observable when using previous qualitative assays. Longer‐chain derivatives such as VB‐S5 and VB‐S6 achieved a high maximum response and a notable activity, aligning with previous finding where the n‐heptyl and n‐octyl were the most active unnatural derivatives.^[^ ^16^ ^]^ These derivatives were nearly equally effective as the natural VB‐D. However, they are less effective than the natural VB‐C. Overall, these results emphasize how subtle variations in acyl chain length can significantly affect both binding affinity and downstream activity, positioning VB‐C as the optimal ligand in this system.

To further explore the structural differences in ligands interactions with BarA, we observed the effects of branching in VB‐type molecules. Many natural GBLs, including VB‐A, VB‐B, and VB‐E, have branched side chains, likely due in part to the high utilization of branched chain fatty acids by *Streptomyces*.^[^ ^44^ ^]^ VB‐E and VB‐B exhibit higher maximum responses and overall activity compared to their linear counterparts (VB‐S4 and VB‐C, respectively), despite displaying reduced binding affinities (Figure 3B). The longer unnatural branched VBs (VB‐S7−9) follow similar trends as the observed for the natural VBs, with some slight differences. Among the seven‐carbon derivatives VB‐S9 has a higher *E*_max_, AUC and fold change compared to its linear counterpart VB‐S5, despite having weaker binding affinity. While VB‐S8 exhibits a similar AUC to VB‐S9 but a lower fold change and *E*_max_. Lastly, VB‐S7 is outperformed by VB‐D across all parameters. This highlights the critical roles of both chain length and branching in modulating repressor function, which is consistent with our previous findings from the ScbR/SCBs regulatory system.^[^ ^14^ ^]^ Given the natural preference of *Streptomyces* for branched‐chain fatty acids over the straight‐chain counterparts used by bacteria like *E. coli*, it underlies the superior performance of the branched VBs, suggesting a potential mechanism for selectivity. Additionally, it suggests that using *E. coli* for a chassis for production of GBLs, as has previously been done,^[^ ^37^ ^,^ ^45^ ^]^ may not be the ideal host for heterologous expression of GBLs. Among the natural branched VBs, VB‐A stood out due to its very potent binding (*K*_D_ = 72 nM), making it one of the most favorable ligands overall. However, it does have a lower *E*_max_, resulting in its overall activity being lower than that of VB‐B or VB‐E. This brings up an important point that more potent binding does not necessarily mean improved derepression. This likely relates to the need for the ligand to not just bind but also induce conformational changes in the repressor, to effectively cause derepression, as has been previously observed for other GBL‐type ligands interacting with their cognate repressors.^[^ ^46^ ^,^ ^47^ ^]^

Lastly, the impact of ligand oxidation state was also explored, as it has been noted to play a crucial role in influencing both binding affinity and regulatory activity in this system and related ones.^[^ ^14^ ^,^ ^16^ ^]^ In general, reduced (alcohol) derivatives showed greater maximum responses and fold change than their oxidized counterparts (A‐factor type analogs), reflecting a general preference for the reduced state (Figure 3C). Nevertheless, exceptions such as the oxidized form of VB‐A and kVB‐7 highlighted the context‐dependent nature of oxidation effects, where oxidation could selectively enhance either potency or efficacy depending on the specific ligand structure. Our results not only provide a quantitative way to understand BarA‐ligand interactions but also indicate the intriguing possibility that virginiamycin biosynthesis in *S. virginiae* could also be induced or even enhanced by non‐native hormones.

### 2.5 Molecular Modeling for Improved Understanding of Ligand Specificity

**Figure 4:**
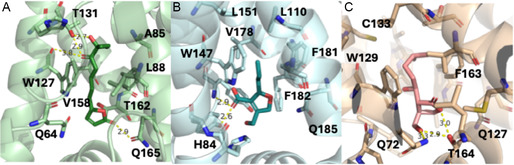
Comparison of AvaR1, mmfR, and barA and their cognate ligands. A) Crystal structure of AvaR1 (light green) with its ligand avenolide (dark green) with key residues indicated (PDB 6WP9). B) Crystal structure of MmfR (light blue) with its ligand MMF2 (teal) with key residues indicated (PDF 6SRN). C) AlphaFold3 model of BarA (light tan) with its ligand VB‐E (peach) docked into its predicted ligand binding site. Residues believed to be key to the interaction are indicated. Docking scores, along with images of other VBs docked with BarA, can be found in Figure S3, S4 and Table S5, Supporting Information**.**

To further explore the potential reasons for the ligand specificity of BarA, we chose to perform docking studies of the ligands with an AlphaFold3 model^[^ ^48^ ^]^ of BarA. To date, only two GBL‐type quorum sensing molecules have been crystallized with their respective repressors: avenolide with AvaR1 and MMF2 with MmfR (**Figure** **4**A,B).^[^ ^46^ ^,^ ^47^ ^]^ In both cases, key Trp (127 for AvaR1, 147 for MmfR), Val (158 for AvaR1, 178 for MmfR), and Phe (161 for AvaR1, 181 for MmfR) residues surround the alkyl chain. These residues are generally very well conserved across this class of repressors and are also seen in BarA (W129, V160, and F163, Figure 4C). Gratifyingly, docking of VB‐E revealed a similar docking pose with BarA to the crystal structures of avenolide with AvaR1 and MMF2 with MmfR. Specifically, the alkyl chain of VB‐E is similarly surrounded by these key residues. Additionally, the butanolide core of VB‐E appears to make hydrogen bonding interactions with T164 similar to the hydrogen bonding observed between the core of avenolide and T162. These similarities give us confidence in the prediction of the poses generated in the docking studies. After the successful docking of VB‐E, we docked additional derivatives into the model of BarA with varying results. Very short chains (e.g. VB‐S1 and VB‐S2) had comparable docking scores to that of the longer side chains but lacked consistency in docking poses (Figure S3, S4, and Table S5, Supporting Information). The lack of a uniform docking pose is consistent with their poor activity in the GFP‐assay. Increasing chain length (e.g. VB‐S3 and VB‐S4) had slightly improved docking scores and docking poses more similar to that observed with VB‐E. However, as the chain length continued to extend (e.g. VB‐D and VB‐S5), docking scores decreased drastically. This is despite the fact that the docking pose of VB‐D was relatively consistent with the medium length aliphatic chains. Similarly, the kVBs generally had the best docking scores despite generally performing less well in the GFP assay compared to their reduced counterparts. This discrepancy in docking score and activity might be related to the fact that increased binding does not necessary correlate with increased derepression. Another reason for this discrepancy could be explained by the fact that the keto‐derivatives exist in equilibrium with their hemiketal, resulting in molecules that likely have different binding affinities. Overall, while simple docking studies are useful for determining the chain lengths that likely fit into a binding site and potentially helping to determine important interacting residues, it seems unlikely that docking alone will be able to predict active ligands. Instead, more complex, computationally expensive molecular dynamics studies that evaluate the change in repressor conformation upon binding will likely be necessary for such predictions.

## 3 Conclusions

Understanding the systems that regulate production of NPs is important for improved production of known NPs and potentially for applications in discovery of novel NPs. BarA is known to repress production of the virginiamycin from *S. viriginiae*, with the VB quorum sensing molecules acting as key signals in alleviating this repression. Herein, we describe efficient and diversifiable synthesis of enantio‐ and diastereopure VBs as well as a rapid and quantitative BarA GFP reporter assay for their evaluation. This has enabled identification of VB‐type molecules likely capable of inducing increased production of virginiamycin, an important veterinary antibiotic. Additionally, this provides a strong basis for the potential utilization of the VBs in combination with BarA in synthetic circuits.

## Supporting Information

The authors have included additional information including materials and methods, supplemental figures, supplemental tables, and NMR spectra in the supporting information.

## Conflict of Interest

The authors declare no conflict of interest.

## Supporting information

Supplementary Material

## Data Availability

The data that support the findings of this study are available in the supplementary material of this article.
