# Supplementary material for: Diastereoselective, Diversifiable Synthesis and Biological Evaluation of the Virginiamycin Inducers, the Virginiae Butanolides
Source: Chembiochem. 2025 Aug 14;26(17):e202500386. doi: 10.1002/cbic.202500386 (PMC12442216; doi:10.1002/cbic.202500386)
Supplement: Supplementary file 1 — Supplementary Material [file CBIC-26-e202500386-s001.pdf]

Supplementary Materials

Diastereoselective, Diversifiable Synthesis and Biological Evaluation of the Virginiamycin Inducers, the Virginiae Butanolides

Kylie G. Castator,[1] Manuela Frias-Gomez,[1] Lauren E. Wilbanks,[1] Elizabeth I. Parkinson[1,2,*]

[1]James Tarpo Jr. and Margaret Tarpo Department of Chemistry, Purdue University, West Lafayette, IN, USA
[2]Borch Department of Medicinal Chemistry and Molecular Pharmacology, Purdue University, , West Lafayette, IN, USA
*corresponding author: eparkins@purdue.edu

Contents

Supplemental Biological Data 3
- **Figure S1.** Design of the plasmid for the BarA GFP reporter assay. 3
- **Figure S2.** BarA GFP reporter assay results. 4
- **Table S1.** BarA GFP reporter assay results. 5
- **Table S2.** Strains and plasmids used in this study 6
- **Table S3.** Primers used in this study 6
- **Table S4**. Sequences relevant to this study 7

Biological Materials and Methods 8
- **Strains, Media, and Cloning Conditions** 8
- **GFP Assay Vector Cloning** 8
- **GFP Induction Assay Protocol** 9
- **Flow Cytometry Analysis** 9

Molecular Docking Supplemental Data 10
- **Figure S3**: Molecular Docking of the VB derivatives in the active site of an Alpha Fold3 model of BarA. 11
- **Figure S4**: Overlays of docking of VB derivatives in an AlphaFold3 model of BarA. 12
- **Table S5.** Docking scores for VBs with an AlphaFold3 model of BarA 13
- **Molecular Docking Methods** 13

Synthetic Supplemental Data 14
- **Figure S5**: The enantiomeric excess of compound **1** 14
- 15
- 15
- **Figure S6:** Comparison of Gräfe Factor and VB NMRs. 15
- **Table S6.** Comparison of VB and Gräfe Factor ^{1}H NMRs 16
- **Table S7.** Comparison of VB and Gräfe Factor ^{13}C NMRs 16
- **Synthetic Methods** 17
- **NMR Spectra** 34

Supplementary References 84

Supplemental Biological Data

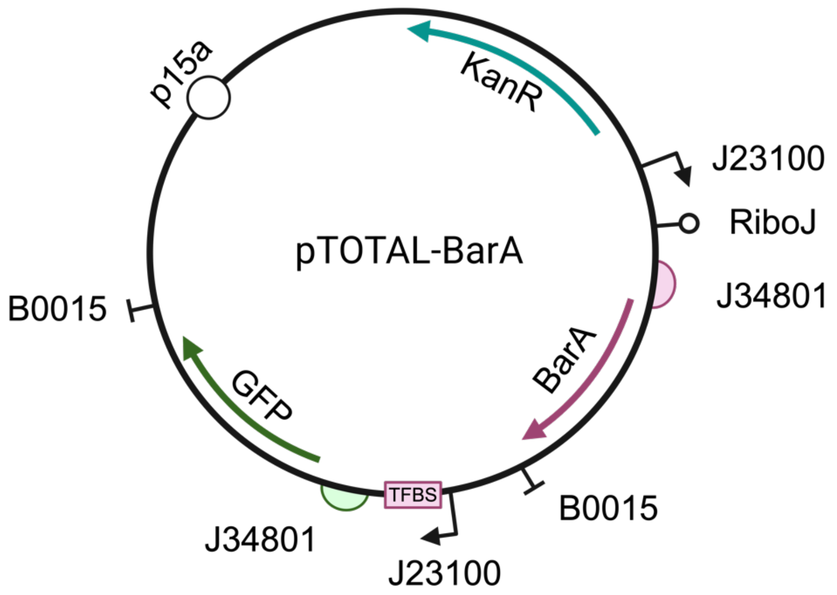

Figure S1. Design of the plasmid for the BarA GFP reporter assay. The plasmid construct shows BioBrick parts and IDs.[1]

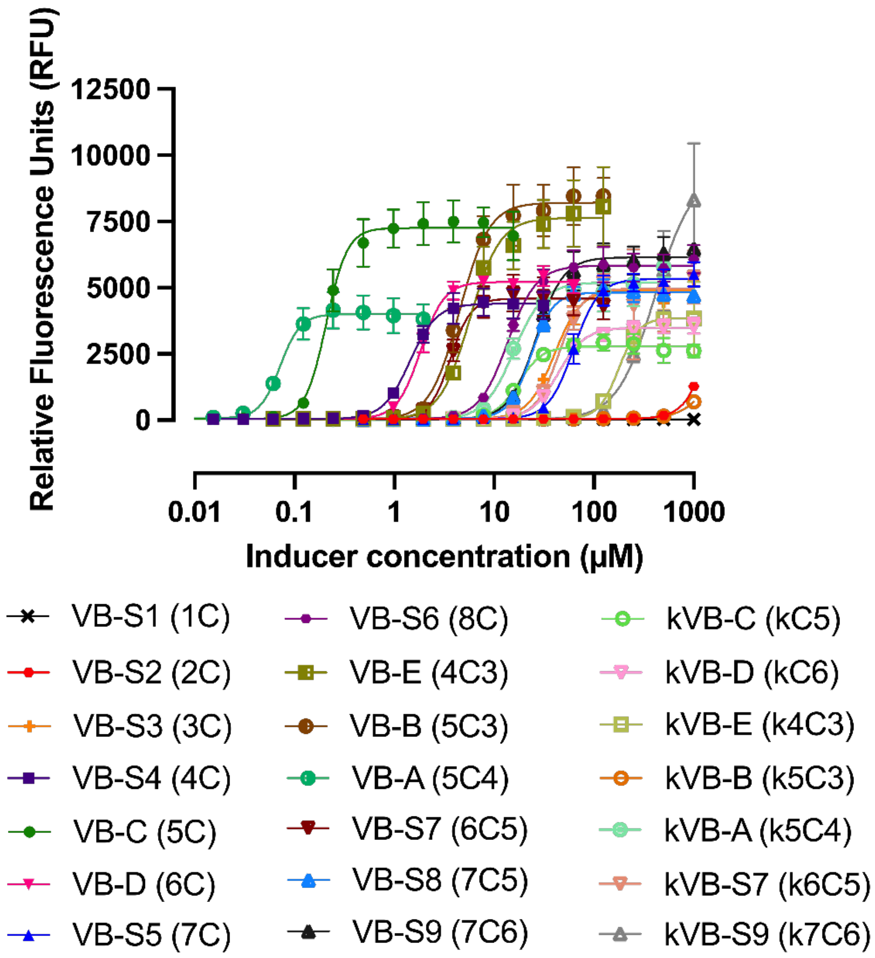

Figure S2. BarA GFP reporter assay results.

The notation in parentheses (e.g., 4C) refers to the length of the carbon chain extending from the exocyclic hydroxyl group, with the number indicating the number of carbon atoms. If additional numbers are included (e.g., 4C3), they indicate the position of a methyl-branch along that chain. Oxidized forms of the hormones are denoted with a lower-case k. Results are averages from a minimum of three biological replicates. Standard error of the mean is indicated. Curves were fitted using GraphPad Prism 10.4.1. Nonlinear regression (curve fit), log[agonist] vs response – Variable slope (four parameters).

Table S1. BarA GFP reporter assay results.

The notation in parentheses (e.g., 4C) refers to the length of the carbon chain extending from the exocyclic hydroxyl group, with the number indicating the number of carbon atoms. If additional numbers are included (e.g., 4C3), they indicate the position of a methyl-branch along that chain. Oxidized forms of the hormones are denoted with a lower-case k.

Molecule	E_{max} (RFU)	Kd (μM)	HillSlope	AUC	FoldChange
VB-S1 (1C)	--	>1000	--	--	--
VB-S2 (2C)	--	>1000	--	--	--
VB-S3 (3C)	4919	40.25	3.276	5405	175
VB-S4 (4C)	4397	1.43	3.248	4636	157
VB-C (5C)	7256	0.2078	4.124	7046	267
VB-D (6C)	5219	1.838	3.665	4913	184
VB-S5 (7C)	5335	62.54	3.434	4823	187
VB-S6 (8C)	5835	13.69	3.014	5621	210
VB-E (4C3)	7638	5.719	2.878	7769	309
VB-B (5C3)	8209	4.499	2.921	9235	305
VB-A (5C4)	4003	0.07159	4.237	4696	157
VB-S7 (6C5)	4593	3.652	4.225	4452	177
VB-S8 (7C5)	4824	23.44	3.936	5106	167
VB-S9 (7C6)	6149	26.39	3.186	5920	212
kVB-C (kC5)	2783	17.59	3.657	3328	105
kVB-D (kC6)	3493	43.75	3.216	3699	124
kVB-E (k4C3)	3856	177.8	4.319	2967	149
kVB-B (k5C3)	--	>1000	--	--	--
kVB-A (k5C4)	5204	15.82	2.896	6048	198
kVB-S7 (k6C5)	4967	44.62	3.748	5222	201
kVB-S9 (k7C6)	--	>1000	--	--	--

Table S2. Strains and plasmids used in this study

	Strain or Plasmid	Relevant Characteristics	Source or Reference
Strains	NEB®5-alpha	fhuA2Δ(argF-lacZ)U169 phoA glnV44 Φ80Δ(lacZ)M15 gyrA96 recA1 relA1 endA1 thi-1 hsdR17	NEB
	DH10B™	F– *mcrA* Δ(*mrr-hsdRMS-mcrBC*) φ80*lacZ*ΔM15 Δ*lacX74 recA1 endA1 araD139* Δ (*ara-leu*)7697 *galU galK* λ– *rpsL*(Str^R) *nupG*	Thermo Scientific™
Plasmids	pLW0003		Wilbanks et al. 2023
	pET-28b(-)		Novagen
	pRF-ScbR		Addgene #49370 Stanton et al. 2013
	pLW0010		This study
	pTOTAL-BarA		This study

Table S3. Primers used in this study

Primer Use	Primer Number	Sequence (5' -> 3')
Amplify pLW0003 to replace chloramphenicol with kanamycin resistance	1	tttgatatcgagctcgcttgg
	2	gagtgccacctgacgtc
Amplification of kanamycin cassette from pET28b(-)	3	gaaccgtaaaaaggccgcgttgctggcgtttttccataggctccgcc
	4	agatcaaaggatcttcttgagatcctttttt
Amplify pLW0003 for origin replacement with p15a	5	gaagatcctttgatcttttctacggg
	6	aaacgccagcaacgc
Amplify p15a origin	7	gaaccgtaaaaaggccgcgttgctggcgtttttccataggctccgcc
	8	tgaccccgtagaaaagatcaaaggatcttcttgagatcgttttggtctgcg

Table S4. Sequences relevant to this study

Backbone sequence	CATATGcgtaaaggagaagaacttttcactggagttgtcccaattcttgttgaattagatggtgatgttaatgggcacaaattttctgtcagtggagagggtg<br>aaggtgatgcaacatacggaaaacttacccttaaatttatttgcactactggaaaactacctgttccatggccaacacttgtcactactttcggttatggtgttca<br>atgctttgcgagatacccagatcacatgaaacagcatgactttttcaagagtgccatgcccgaaggttatgtacaggaaagaactatatttttcaaagatgac<br>gggaactacaagacacgtgctgaagtcaagtttgaaggtgatacccttgttaatagaatcgagttaaaaggtattgattttaaagaagatggaaacattcttg<br>gacacaaattggaatacaactataactcacacaatgtatacatcatggcagacaaacaaaagaatggaatcaaagttaacttcaaaattagacacaac<br>attgaagatggaagcgttcaactagcagaccattatcaacaaaatactccaattggcgatggccctgtccttttaccagacaaccattacctgtccacacaa<br>tctgccctttcgaaagatcccaacgaaaagagagaccacatggtccttcttgagtttgtaacagctgctgggattacacatggcatggatgaactatacaaa<br>taataatctagacactgatagtgctagtgtagatcactactagagccaggcatcaaataaaacgaaaggctcagtcgaaagactgggcctttcgttttatctg<br>ttgtttgtcggtgaacgctctctactagagtcacactggctcaccttcgggtgggcctttctgcgtttatacccgggtactagaggtccggcaaaaaagggcaa<br>ggtgtcaccaccctgcccttttctttaaaaccgaaaagattacttcgcgttatgcaggcttcctcgctcactgactcgctgcgctcggtcgttcggctgcggcg<br>agcggtatcagctcactcaaaggcggtaatacggttatccacagaatcaggggataacgcaggaaagaacatgtgagcaaaaggccagcaaaaggc<br>caggaaccgtaaaaaggccgcgttgctggcgtttttccataggctccgcccccctgacaagcatcacgaaatctgacgctcaaatcagtggtggcgaaac<br>ccgacaggactataaagataccaggcgtttccccctggcggctccctcgtgcgctctcctgttcctgcctttcggtttaccggtgtcattccgctgttatggccgc<br>gtttgtctcattccacgcctgacactcagttccgggtaggcagttcgctccaagctggactgtatgcacgaaccccccgttcagtccgaccgctgcgccttatc<br>cggtaactatcgtcttgagtccaacccggaaagacatgcaaaagcaccactggcagcagccactggtaattgatttagaggagttagtcttgaagtcatgc<br>gccggttaaggctaaactgaaaggacaagttttggtgactgcgctcctccaagccagttacctcggttcaaagagttggtagctcagagaaccttcgaaaa<br>accgccctgcaaggcggttttttcgttttcagagcaagagattacgcgcagaccaaaacgatctcaagaagatcctttgatcttttctacggggtcagacgct<br>cagtggaacgaaaactcacgttaagggattttggtcatgagattatcaaaaaggatcttcacctagatccttttaaattaaaaatgaagttttaaatcaatctaa<br>agtatatatgagtaaacttggtctgacagctcgaggcttggattctcaccaataaaaaacgcccggcggcaaccgagcgttctgaacaaatccagatgga<br>gttctgaggtcattactggatctatcaacaggagtccaagcgagctcgatatcaaattagaaaaactcatcgagcatcaaatgaaactgcaatttattcatat<br>caggattatcaataccatatttttgaaaaagccgtttctgtaatgaaggagaaaactcaccgaggcagttccataggatggcaagatcctggtatcggtctgc<br>gattccgactcgtccaacatcaatacaacctattaatttcccctcgtcaaaaataaggttatcaagtgagaaatcaccatgagtgacgactgaatccggtga<br>gaatggcaaaagtttatgcatttctttccagacttgttcaacaggccagccattacgctcgtcatcaaaatcactcgcatcaaccaaaccgttattcattcgtga<br>ttgcgcctgagcgagacgaaatacgcgatcgctgttaaaaggacaattacaaacaggaatcgaatgcaaccggcgcaggaacactgccagcgcatca<br>acaatattttcacctgaatcaggatattcttctaatacctggaatgctgttttcccggggatcgcagtggtgagtaaccatgcatcatcaggagtacggataaa<br>atgcttgatggtcggaagaggcataaattccgtcagccagtttagtctgaccatctcatctgtaacatcattggcaacgctacctttgccatgtttcagaaacaa<br>ctctggcgcatcgggcttcccatacaatcgatagattgtcgcacctgattgcccgacattatcgcgagcccatttatacccatataaatcagcatccatgttgg<br>aatttaatcgcggcctagagcaagacgtttcccgttgaatatggctcattttagcttccttagctcctgaaaatctcgataactcaaaaaatacgcccggtagtg<br>atcttatttcattatggtgaaagttggaacctcttacgtgcccgatcaactcgagtgccacctgacgtctaagaaaccattattatcatgacattaacctataaa<br>aataggcgtatcacgaggcagaatttcagataaaaaaaatccttagctttcgctaaggatgatttctggctgcagcgaggaagcggGGCCGGCC
BarA/TFBS-GFP gene block	acgtcatGGCCGGCCttgacggctagctcagtcctaggtacagtGTAGCTTGATCATTATAAcagctgtcaccggatgtgctttccggtctga<br>tgagtccgtgaggacgaaacagcctctacaaataattttgtttaaACTAGAaaagaggagaaagaattcATGGCAGTTCGTCATGAACG<br>TGTTGCCGTGCGTCAAGAACGTGCAGTTCGTACCCGTCAGGCAATTGTTCGTGCAGCAGCAAGCGTTTTT<br>GATGAATATGGTTTTGAAGCAGCAACCGTTGCAGAAATTCTGAGCCGTGCAAGCGTTACCAAAGGTGCAA<br>TGTATTTTCATTTTGCCAGCAAAGAAGAACTGGCACGCGGAGTTCTGGCAGAACAGACCCTGCATGTTGC<br>AGTTCCGGAAAGCGGTAGCAAAGCACAAGAACTGGTTGATCTGACCATGCTGGTTGCACATGGTATGCTG<br>CATGATCCGATTCTGCGTGCAGGCACCCGTCTGGCACTGGATCAGGGTGCAGTTGATTTTTCAGATGCAA<br>ATCCGTTTGGTGAATGGGGTGATATTTGTGCACAGCTGCTGGCCGAAGCGCAAGAACGCGGTGAAGTTC<br>TGCCGCATGTTAATCCGAAAAAGACCGGTGATTTTATTGTGGGTTGTTTTACCGGTCTGCAGGCAGTTAGC<br>CGTGTTACCAGCGATCGTCAGGATCTGGGTCATCGTATTAGCGTTATGTGGAATCATGTTCTGCCGAGCAT<br>TGTTCCGGCAAGCATGCTGACCTGGATTGAAACCGGTGAAGAACGTATTGGTAAAGTTGCAGCCGCAGCA<br>GAAGCAGCCGAAGCCGCAGAAGCATCTGAAGCAGCCAGTGATGAATAATAAtaagcggccgccactgatagtgctagt<br>gtagatcactactagagccaggcatcaaataaaacgaaaggctcagtcgaaagactgggcctttcgttttatctgttgtttgtcggtgaacgctctctactaga<br>gtcacactggctcaccttcgggtgggcctttctgcgtttataggtacctactagagaggttctgttaagtaactgaacccaatgtcgttagtgacgcttacctctta<br>agaggtgTCGACttgacggctagctcagtcctaggtacagtgctagcaGGAAAGATACATACCAACCGGTTCTTTTGAaagcttcta<br>gtaaagaggagaaatagCATATGtcggatt

FseI / NdeI

Biological Materials and Methods

Strains, Media, and Cloning Conditions

During the cloning process, E. coli strain DH5a was used for plasmid propagation. Cells were grown in LB broth with kanamycin. E. coli strain DH10B was used for all fluorescence assays. All PCR amplifications were performed using Q5 High-Fidelity 2X Master Mix (New England Biolabs, M0492S) with the following general methodology, adjusting the annealing temperature and the amplification time in accordance with the manufacturer's protocols. For each template, triplicate PCR reactions were performed. Each of the three PCR tubes receives 10 µL of Q5 High-Fidelity 2X Master Mix, 2 µL of mixed F/R primers at 500 µM each, 1 µL of 1 ng/µL plasmid template, and 7 µL of nuclease free water. Tube 1 will be amplified at the annealing temperature recommended by NEBs TM calculator. Tube 2 will receive 3% DMSO and be amplified at the recommended annealing temperature. PCR for tube 3 without DMSO was performed using the stepdown/touchdown PCR method. The annealing temperature was set 2 °C over manufacturer guidelines and the thermocycler was programed to reduce the annealing temperature by 0.3 °C every round. Thermocyclers were operated under the following general program: (1) initial denaturation at 98 °C for 30s; (2) 98 °C for 20s; (3) 60-70 °C for 15s (stepdown when appropriate) (4) 72 °C (template dependent, 20 sec per 1kb); (5) repeat steps 2–4 for a total of 30 cycles; (6) 72 °C for 2 min and (7) hold at 4 °C. All PCR reactions were run on a 0.8% agarose gel with GeneRuler 1kb plus ladder (Thermo Scientific, SM1331); successful amplification was gel excised, pooled, and DNA was extracted using the Freeze 'N Squeeze™ DNA Gel Extraction Spin Columns (Bio-Rad, 7326165) adhering to manufacturer protocol. DNA from the spin column flowthrough was aliquoted into ice cold 900 µL 100% EtOH with 30 µL 3M sodium acetate for ethanol precipitation. 1 µL of GlycobBlue (Invitrogen, AM9516) was added to the mix for DNA visualization. The DNA precipitation mix was centrifuged at 15,000 rpm, washed twice with 700 µL of 70% EtOH, then the DNA pellet was resuspended in 15 µL nuclease free water. For isothermal assembly, a 3:1 molar insert to backbone ratio was used with NEBuilder HiFi DNA Assembly MasterMix (New England Biolabs, E2621S). For restriction digest cloning, plasmids were digested according to manufacturer protocol (New England Biolabs). For restriction digest cloning, plasmids were digested according to manufacturer protocol (New England Biolabs). Briefly, 1 µg plasmids or insert were combined with 10X CutSmart buffer, 1 µL of each enzyme, and nuclease free water was added for a total volume of 50 µL. These reactions were incubated for 1 hour at 37 °C , then separated by DNA gel electrophoresis and gel extracted as previously described. The ligation reaction was prepared according to manufacturer protocol (New England Biolabs) except that a 5:1 insert to backbone ratio was used for the ligation reaction. NEB® 5-alpha Competent E. coli cells (NEB, C2987H) were used for cloning. Transformations for both isolthermal assembly and restriction digest cloning were performed according to manufacturer protocol. All clones were screened by restriction digest then confirmed by Sanger sequencing (Azenta). Vectors for analysis were transformed into DH10B competent cells (Thermo Scientific, EC0113) in accordance with manufacturer protocol. All strains and plasmids used, primers, and DNA sequences relevant to this study are listed in Tables S2, S3, and S4, respectively.

GFP Assay Vector Cloning

First, the chloramphenicol resistance gene of pLW0003 was replaced with a kanamycin resistance gene. Primers 1/2 were used to amplify the pLW0003 the backbone without the chloramphenicol resistance gene. The kanamycin resistance cassette was amplified from pET28b with primers 3/4. PCR reaction, gel extraction, clean up, isothermal assembly, and transformation were performed as described in the "Strains, Media, and Cloning Conditions" section. The

resulting vector was amplified with primers 5/6 to change the origin of replication to p15A. p15A insert was amplified from plasmid pRF-ScbR with 7/8. PCR reaction, gel extraction, clean up, isothermal assembly, and transformation were performed as described in the "Strains, Media, and Cloning Conditions" section. These modifications resulted in pLW0010 which was then further modified to create the BarA GFP induction assay vector pTOTAL-BarA. A DNA fragment that comprised the promoter[2] and a codon optimized coding region of BarA (GeneBank: BAA06981.1), as well as the GFP promoter containing the BarA TFBS was ordered from IDT. The pLW0010 vector and purchased BarA fragment were digested with FseI/NdeI and cloned using restriction digest cloning. Plasmids were isolated (Zymo, D016), and sequences were verified using Sanger sequencing.

GFP Induction Assay Protocol

A single colony was used to inoculate 5 mL LB with 50 µg/mL kanamycin. The culture was incubated for 16-18 hours at 37 °C in a Cole-Parmer TR-200D-120 Tube Rotator with rotation (40 rpm). The overnight culture was diluted 1:100 into supplemented M9 media (1X M9 salts, 1 mM thiamine hydrochloride, 0.4% glucose, 0.2% casamino acids, 2 mM $MgSO_4$, 0.1 mM $CaCl_2$) and 198 µL was dispensed into each well of a round bottom Corning™ Clear Polystyrene 96-Well Microplate. The cultures were grown to an OD_{600} of 0.1 and induced with 2 µL of 100X stock solutions of molecule dissolved in DMSO. OD_{600} reads were taken using a Molecular Devices SpectraMax iD3. After induction, the cultures were incubated for 5 hours. 1 µL of each culture was diluted into 199 µL PBS with 2mg/mL spectinomycin (final concentration 10 µg/mL spectinomycin) to arrest cell growth, incubated 20 minutes to arrest protein synthesis, then analyzed using a ThermoFisher Attune NxT Flow Cytometer. Plates that were not immediately analyzed after dilution were stored at 4 ºC and vortexed for 1 minute before flow cytometry analysis.

Flow Cytometry Analysis

An injection volume of 50 µL and a flow rate of 25 µL/min were used for analysis. Events were gated X -FSC A 10^3 to 10^5 and Y-FSC H 10^3 to 10^5 using floreada.io, mean fluorescence was calculated. The autofluorescence of a DH10B vector-empty control was subtracted from each fluorescent mean. Fold change was calculated by dividing the mean induced fluorescence by the mean DMSO control fluorescence. Data presented is an average of biological triplicate collected on separate days; error bars are +/1 standard deviation. K_D and Hill coefficients were determined by fitting the data to a Hill function.

$$f(x) = y = Y_{min} + (Y_{max} - Y_{min}) \frac{x^n}{x^n + K_D^n}$$

Molecular Docking Supplemental Data

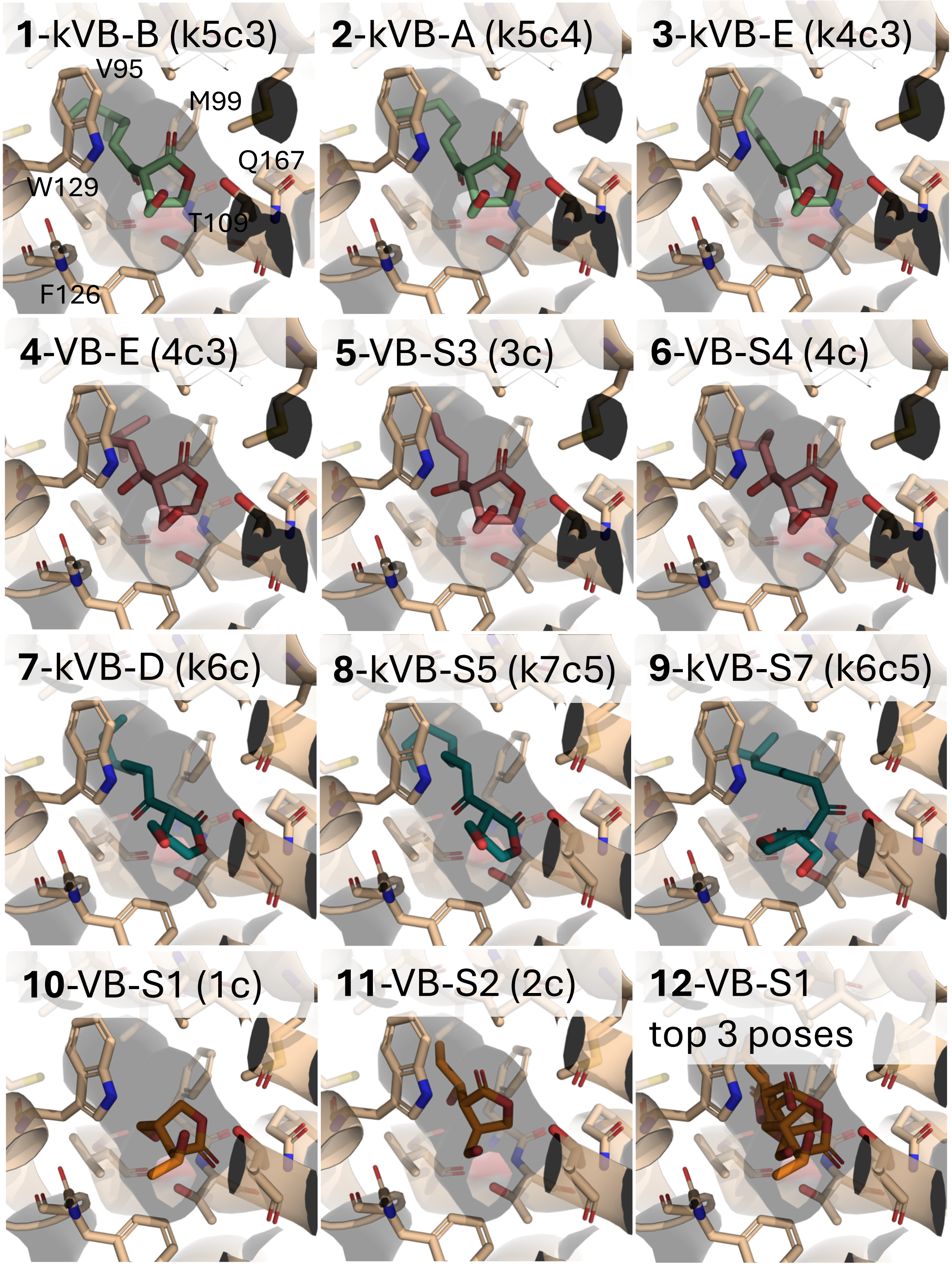

Figure S3: Molecular Docking of the VB derivatives in the active site of an Alpha Fold3 model of BarA.

The notation in parentheses (e.g., 4C) refers to the length of the carbon chain extending from the exocyclic hydroxyl group, with the number indicating the number of carbon atoms. If additional numbers are included (e.g., 4C3), they indicate the position of a methyl-branch along that chain. Oxidized forms of the hormones are denoted with a lower-case k. The frame is conserved through each pose, therefore key amino acids labelled in pose 1 apply to all 12 poses. Docking scores can be found in **Table S5**. Poses 1-3 represent the top three docking outputs. Poses 4-6 are the top three VB-type ligands with a fully reduced exocyclic hydroxyl group. Poses 7-8 represent the top three preforming keto derivatives that had six or more carbons after the exocyclic carbonyl. Poses 9-10 are the top docking output of the VB-S1 and VB-S2. Pose 12 is the top three docking poses that VB-S1, showing the disorder of the shortest chained derivative.

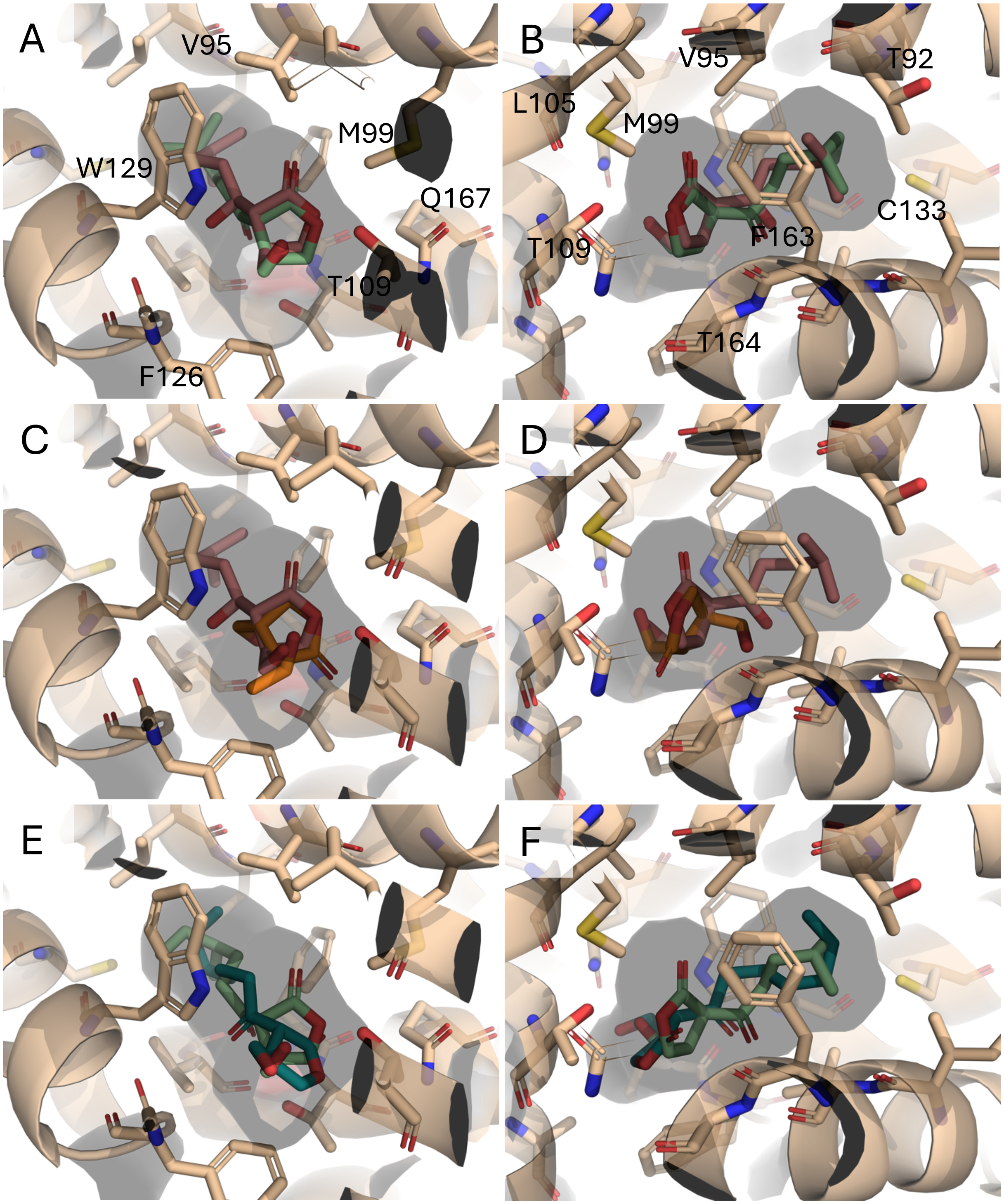

Figure S4: Overlays of docking of VB derivatives in an AlphaFold3 model of BarA.

The notation in parentheses (e.g., 4C) refers to the length of the carbon chain extending from the exocyclic hydroxyl group, with the number indicating the number of carbon atoms. If additional numbers are included (e.g., 4C3), they indicate the position of a methyl-branch along that chain. Oxidized forms of the hormones are denoted with a lower-case k. The frame of pose A is conserved through poses C and E. The frame of B is conserved through poses D and F.A) Looking at the front of the active site of poses 1 and 4 docked together. B) The back view of

poses 1 and 4 docked together. C) Looking at the front of the active site of poses 4 and 10 docked together. D) The back view of poses 4 and 10 docked together. E) Looking at the front of the active site of poses 1 and 7 docked together. F) The back view of poses 1 and 7 docked together.

Table S5. Docking scores for VBs with an AlphaFold3 model of BarA

Pose*	Ligand	Docking Score
1	kVB-B	-7.32
2	kVB-A	-7.23
3	kVB-E	-7.18
4	**VB-E**	-6.08
5	VB-S3	-5.91
6	VB-S4	-5.86
7	kVB-D	-4.30
8	kVB-S5	-4.10
9	A-factor	-3.31
10	VB-S1	-5.70
11	VB-S2	-5.65
12	VB-S1 docked three times	-5.70, -5.55, -4.77
not shown	**VB- A**	-4.98
not shown	**VB-B**	-5.64
not shown	**VB-C**	-5.12
not shown	**VB-D**	-3.35

*Images for poses can be found in **Figure S3** and **Figure S4**.

Molecular Docking Methods

An Alpha Fold3 model[3] was generated of a BarA dimer, with a predicted template modeling score of 0.9, giving us a high degree of confidence of this predictive model. This protein was exported and prepared for docking using the Protein Preparation Workflow Wizard in Schrödinger Maestro. Docking substrates (all synthesized VB and A-Factor like compounds) were prepared as 2D structures utilizing PerkinElmer's Chemdraw Software. The structures were then transferred into the Maestro workspace, and transformed into their 3D structure, and 3D energy minimalization was performed using the LigPrep tool. The protein's active site was found by identifying the key conserved tryptophan (Y129) and threonine (T164). The Receptor Grid Generation tool was then utilized to prepare a grid box (12 Å) encompassing the binding pocket identified by our two key residues. The Ligand Docking tool was then utilized to dock each prepared ligand into the BarA binding pocket. The Maestro software then calculated the docking score for each ligand and ranked each ligand by their energy within the binding pocket. The poses generated were then exported into pymol, which was utilized to compare each docking pose as well as generate pictures of each ligand within the BarA active site.

Synthetic Supplemental Data

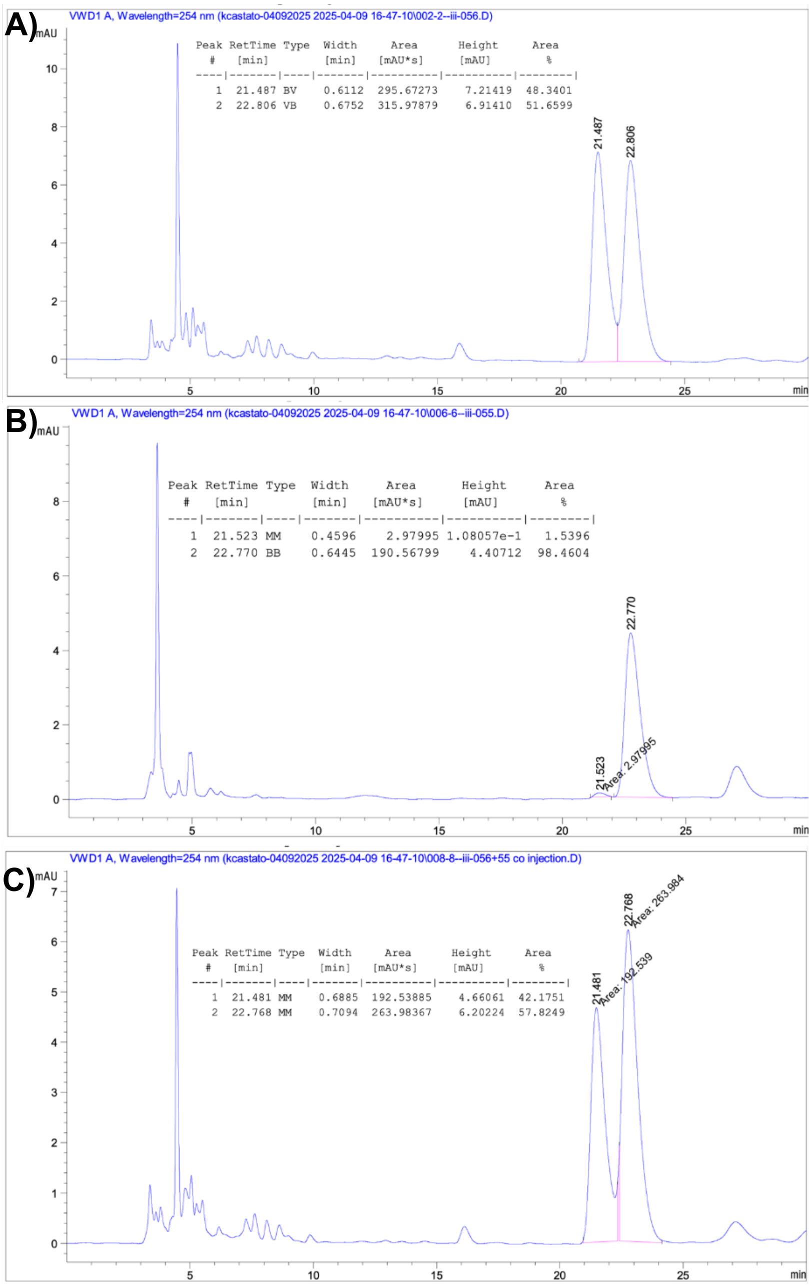

Figure S5: The enantiomeric excess of compound **1**

was determined by HPLC using a Chiralpax AD-H column in 2.5% isopropanol in hexanes at 1.0 mL/min at 30 °C with a detection at 254 nm, t_R (R) = 22.8 min and t_R (S) = 21.5 min. A) Spectrum of racemic protected alcohol, compound **1a**.[4] B) Spectrum of copper conjugate hydride addition product, compound **1**. C) Spectrum of co-injection of racemic (compound **1a**) and enantioselective reaction (compound **1**) products.

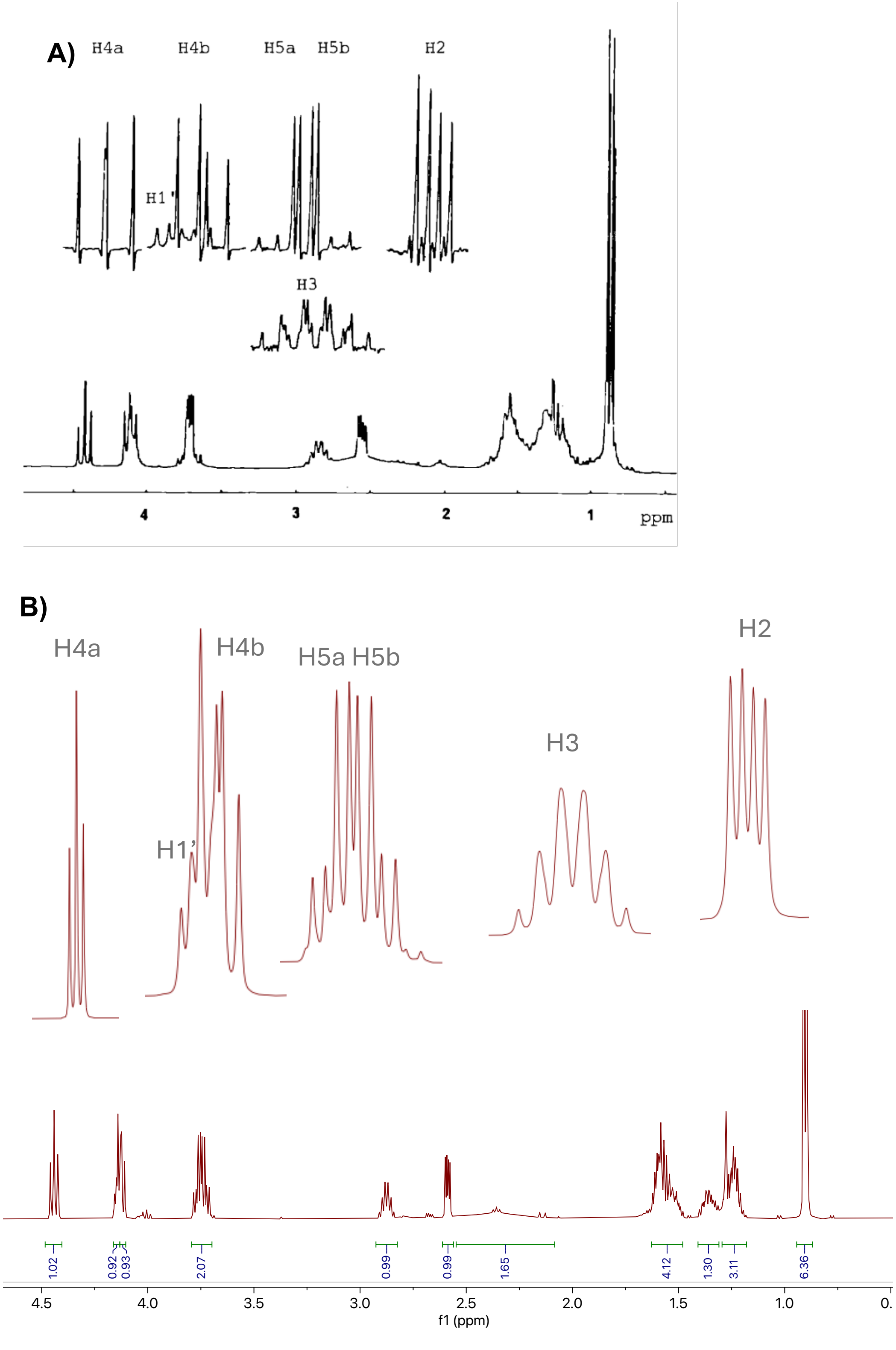

Figure S6: Comparison of Gräfe Factor and VB NMRs.

A) Previously published ^{1}H NMR of Gräfe Factor 1.[5] B) Our NMR of synthetic VB-A (compound **8k**).

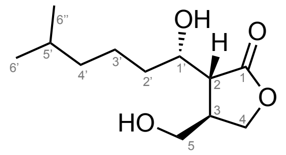

VB-A/Gräfe Factor 1 (**8k**)

Table S6. Comparison of VB and Gräfe Factor ^{1}H NMRs

	VB-A	Yamada[6]	*J*	GF-1	Gräfe Factor[5]	*J*	SVB-A	This Work	*J*
H-No.	ppm	multiplicity	Hz	ppm	multiplicity	Hz	ppm	multiplicity	Hz
H-4a	4.42	dd	9.04, 8.76	4.40		9.03	4.44	dd	8.7, 8.7
H-6	4.13	m					4.14	m	
H-4b	4.10	dd	9.0, 6.88	4.08			4.12	dd	9.0, 6.7
H-5a	3.74	m		3.75		10.49	3.75	qd	10.6, 5.8
H-5b	2.86	m		2.80		8.40, 6.69, 5.78, 5.96	2.86	m	
OH							2.34		
H-3	2.57	dd	7.40, 3.77	2.55		7.26, 3.81	2.59	dd	7.2, 3.8
H-	1.55	m		1.49			1.58		
	1.34	m					1.28		
	0.88	d	6.59	0.879			0.91	d	6.5

*Gräfe did not report multiplicity

Table S7. Comparison of VB and Gräfe Factor ^{13}C NMRs

	VB-A	GF-1	Synthetic VB-A
C-No.	Yamada	Gräfe	This Work
1	178.4	179.06	178.58
1’	70.8	70.84	70.84
4	69.4	69.77	69.48
5	63.3	63.35	63.34
2	48.1	48.34	48.16
4’	38.7	38.75	38.70
3	38.1	38.19	38.13
2’	35.1	35.19	35.09
5’	27.9	27.83	27.90
3’	23.6	23.64	23.60
6’	22.6	22.61	22.58
6’’	22.5	22.61	22.53

Synthetic Methods

General: All synthetic reactions were performed using flame-dried glassware with anhydrous solvents unless otherwise stated. Reagents of the purest grade were purchased from Fisher Scientific and used without any additional purification, unless otherwise specified. Reactions were monitored via thin layer chromatography (TLC) with plates purchased from Fisher Scientific. Reaction components were visualized using either short wave UV light (λ=254 nm) or a $KMnO_4$ stain. Products were purified by column chromatography on silica gel. Proton and Carbon NMRs were taken on a Bruker 500MHz spectrometer in chloroform-*d* purchased from Cambridge Isotopes. The chemical shift (δ) of both proton and carbon spectra are reported in parts per million (ppm) and were referenced to the residual proton signal of the corresponding solvent ($CDCl_3$ δ=7.26 ppm). Multiplicities of the proton spectra are reported as singlet (s), doublet (d), triplet (t), and multiplet (m). IR spectra were taken on a Nicolet FTIR spectrometer. HRMS data was obtained from an LTQ Orbitrap or an ultra-high performance liquid chromatography mass spectrometry (UPLC-MS). Optical rotations were measured from a Rudolph Autopool III S2 polarimeter. Chiral HPLC data was obtained on an Agilent chiral chromatograph, using a Chiralpak AD-H column under the given conditions.

4-((benzyloxy)methyl)furan-2(5*H*)-one (4)

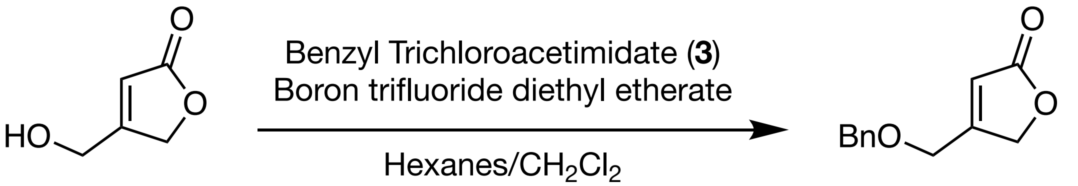

2 **4**

Compound **2**, which was synthesized via known protocol and is in correspondence with literature data,[7] (750 mg, 6.7 mmol, 1.0 equiv.) in CH_2Cl_2 (22 mL) was added to hexanes (44 mL). To this solution was added benzyl 2,2,2-trichloroacetimidate,[8] compound **3**, (1.86 mL, 10.04 mmol, 1.5 equiv.) followed by addition of boron trifluoride diethyl etherate (0.04 mL, 0.34 mmol, 0.05 equiv.). The reaction was stirred for 24 h at room temperature (20 °C to 25 °C). The reaction was filtered over a short pad of celite. The filtrate was then quenched with saturated aqueous $NaHCO_3$ (20mL) and extracted with EtOAc (3 x 50mL) The combined organic layers were then dried with Na_2SO_4, filtered and concentrated. The crude oil was purified via flash column chromatography (to afford compound **4** (765 mg, 56%) as a pale yellow oil. **TLC** R_f = 0.37 (40% EtOAc in hexanes, $KMnO_4$); **^{1}H NMR (500 MHz, $CDCl_3$)** δ 7.30-7.41 (m, 5H), 6.03 (p, J=1.8Hz, 1H), 4.84 (dt, J=1.8,0.9, 2H), 4.59 (s, 2H), 4.39 (dt, J=1.7,0.9, 2H); **^{13}C NMR (126 MHz, $CDCl_3$)** δ 173.33, 166.38, 136.88, 128.69, 128.29, 127.83, 116.05, 73.43, 71.46, 65.51; **FTIR** (Neat, cm^{-1}): *v* 3029, 2979, 2861, 1780, 17226; **HRMS** m/z calculated for $C_{12}H_{12}O_3$ $[M+H]^+$: 205.08592, found $[M+H]^+$: 205.08526. (-3.21ppm)

(*R*)-4-((benzyloxy)methyl)dihydrofuran-2(3*H*)-one (1)

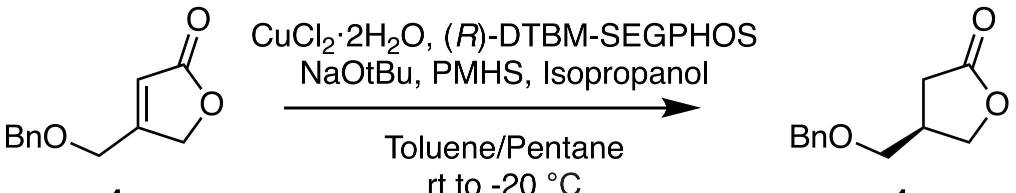

4 **1**

Copper chloride dihydrate (40.8 mg, 0.24 mmol, 0.05 equiv.), (*R*)-DTBM-SEGPHOS (283 mg, 0.24 mmol, 0.05 equiv.) and NaOtBu (94 mg, 0.98 mmol, 0.20 equiv.) were added to a flame dried round bottom flask under a nitrogen atmosphere. The round bottom flask was evacuated and refilled with nitrogen two times. Anhydrous pentane (6.52 mL) and polymethylhydrosiloxane (PMHS) (1.12 mL, 19.6 mmol, 4 equiv.) were added. The mixture was stirred for two hours at room temperature. The reaction was cooled to -20 °C, following which a solution of compound **4** (1.0 g, 4.9 mmol, 1.0 equiv.) in anhydrous toluene (6.52 mL) and isopropanol (1.5 mL, 19.6 mmol, 4 equiv.) was added dropwise over five minutes. The reaction stirred for 5 hours at -20 °C. The reaction was then allowed to warm to room temperature and was quenched with saturated aqueous NH_4Cl (15 mL). The aqueous layer was then extracted with (3 x 25 mL) with EtOAc. The organic layers were combined, dried with Na_2SO_4, filtered, and concentrated. The crude oil was purified via flash column chromatography to afford compound **1** (586 mg, 58%, 97% ee) as a colorless oil. Enantiomeric excess was determined via chiral chromatography (see **Figure S5**). **TLC** R_f = 0.37 (40% EtOAc in hexanes, $KMnO_4$); **Alpha D** $[\alpha]_D^{21}: -32.2°$ (*c* 1.0mg/ml, $CHCl_3$); **^{1}H NMR (500 MHz, CDCl3)** δ 7.42 – 7.27 (m, 5H), 4.53 (s, 2H), 4.40 (dd, *J* = 9.2, 7.5 Hz, 1H), 4.19 (dd, *J* = 9.2, 5.5 Hz, 1H), 3.52 – 3.41 (m, 2H), 2.89 – 2.78 (m, 1H), 2.61 (dd, *J* = 17.7, 9.0 Hz, 1H), 2.37 (dd, *J* = 17.4, 6.3 Hz, 1H); **^{13}C NMR (126 MHz, CDCl3)** δ 176.82, 137.63, 128.57, 127.97, 127.71, 73.42, 70.77, 70.44, 35.48, 31.18; **FTIR (**Neat, cm^{-1}): *v* 3029, 2861, 1776, 1726; HRMS: m/z calculated for $C_{12}H_{14}O_3$ $[M]^+$:206.09429, found $[M]^+$:206.0926. (-8.3ppm)

4-((benzyloxy)methyl)dihydrofuran-2(3*H*)-one (1a)

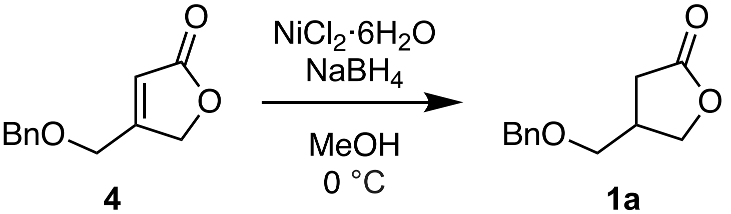

4 **1a**

To a solution of nickel chloride hexahydrate (11.6 mg, 0.049 mmol, 0.2 equiv.) and butenolide **4** (50 mg, 0.245 mmol, 1 equiv.) in methanol (2.5 mL) at 0 °C, sodium borohydride was added portion wise (27.8 mg, 0.74 mmol, 3 equiv.). Acetone was added (5 mL) to the reaction after thirty minutes of stirring at 0 °C. All solvent was then evaporated under reduced pressure. Resulting oil was then purified via flash chromatography to afford compound **1a** (11 mg, 22%). The proton nmr was in accordance with what is being reported for compound **1**.

Acylation Procedure 1

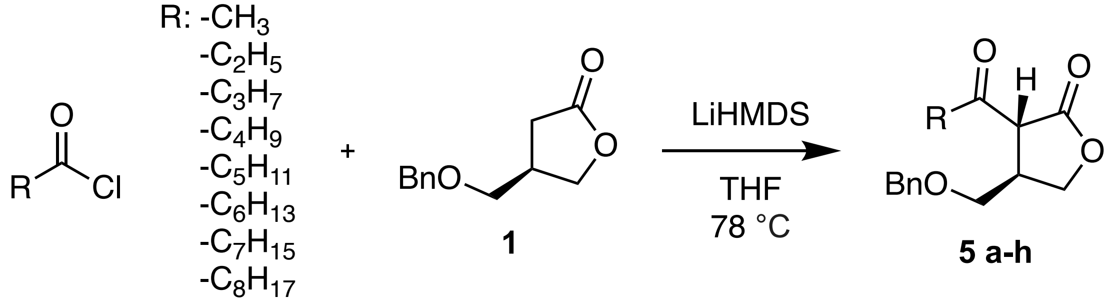

A solution of lactone (**1**) in anhydrous THF (0.1M) was cooled to -78 °C. To the solution, LiHMDS (2.5 equiv., 1M in THF) was added dropwise. The reaction stirred at -78 °C for 1.5 hours. The needed acyl chloride was then added dropwise, and the reaction stirred at -78 °C for another 2 hours. Upon completion, the reaction was quenched with saturated aqueous NH_4Cl (10mL) and extracted with EtOAc (3 x 15 mL). The combined organic layers were dried with Na_2SO_4, filtered and concentrated. The crude product was purified via flash chromatography to afford the protected keto-VBs as a single diastereomer, as observed by NMR.

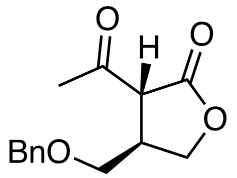

(3*R*,4*R*)-3-acetyl-4-((benzyloxy)methyl)dihydrofuran-2(3*H*)-one (**5a**, protected keto-VB-S1)
Synthesized using acetyl chloride and compound **1** via acylation procedure 1 to afford compound **5c** (70% yield) as a clear, colorless oil.
TLC R_f = 0.17 (20% EtOAc in hexanes, $KMnO_4$); **Alpha D** $[\alpha]_D^{21}: -7.9°$ (*c* 3.6mg/ml, $CHCl_3$); **^{1}H NMR (500 MHz, CDCl$_3$)** δ 7.27-7.38 (m, 5H), 4.51 (t, *J* = 4.38 Hz, 2H), 4.38 (t, *J* = 8.8 Hz, 1H), 4.15 (dd, *J* = 9.0, 6.8 Hz, 1H), 3.66 (d, *J* = 7.2 Hz , 1H), 3.48 (m, 2H), 3.31 (mf, 1H), 2.43 (s, 3H); **^{13}C NMR (126 MHz, CDCl$_3$)** δ 200.03, 172.07, 137.50, 128.59, 128.03, 127.71, 73.33, 69.31, 68.62, 65.87, 55.81, 37.17, 29.65, 15.30; **FTIR** (Neat, cm^{-1}): *v* 2921, 2856, 1778, 1722; **HRMS** m/z calculated for $C_{14}H_{16}O_4$ $[M+H]^+$:248.1049, found $[M+H]^+$:248.1037. (-4.8ppm)

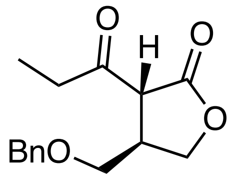

(3*R*,4*R*)-4-((benzyloxy)methyl)-3-propionyldihydrofuran-2(3*H*)-one (**5b**, protected keto-VB-S2)
Synthesized using propionyl chloride and compound 1 via acylation procedure 1 to afford compound **5b** (57% yield) as a clear, colorless oil.
TLC R_f = 0.27 (20% EtOAc in hexanes, $KMnO_4$); **Alpha D** $[\alpha]_D^{21}: -10.4°$ (*c* 3.4mg/ml, $CHCl_3$); **^{1}H NMR (500 MHz, CDCl$_3$)** δ 7.29 (m, 5H), 4.51 (s, 2H), 4.42 (t, *J* = 8.5 Hz, 1H), 4.15 (dd, *J* = 6.4, 2.4 Hz, 1H), 3.65 (d, *J* = 7.4 Hz, 1H)), 3.48 (d, *J* = 4.9 Hz, 2H), 3.32 (m, 1H), 3.00 (m, 1H), 2.58 (m, 1H), 1.09 (t, *J* = 6.9 Hz, 3H); **^{13}C NMR (126 MHz, CDCl$_3$)** δ 202.95, 172.34, 137.51, 128.58, 128.02, 127.71, 73.32, 69.45, 68.76, 54.99, 37.52, 35.91, 7.37; **FTIR** (Neat, cm^{-1}): *v* 2977, 2869, 1778, 1722; **HRMS** m/z calculated for $C_{15}H_{18}O_4$ $[M+H]^+$:262.1205, found $[M+H]^+$:262.119. (-4.8ppm)

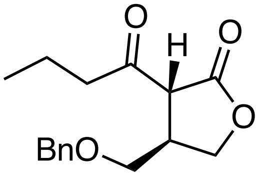

(3*R*,4*R*)-4-((benzyloxy)methyl)-3-butyryldihydrofuran-2(3*H*)-one (**5c**, protected keto-VB-S3)
Synthesized using butyryl chloride and compound **1** via acylation procedure 1 to afford compound **5c** (60% yield) as a clear, colorless oil.
TLC R_f = 0.32 (20% EtOAc in hexanes, $KMnO_4$); **Alpha D** $[\alpha]_D^{21}: -15.8°$ (*c* 1.0mg/ml, $CHCl_3$); **^{1}H NMR (500 MHz, CDCl$_3$)** δ 7.32 (m, 5H), 4.51 (s, 2H),

4.41 (s, 1H), 4.15 (s, 1H), 3.64 (s, 1H), 3.47 (s, 2H), 3.30 (s, 1H), 2.91 (s, 1H), 2.58 (s, 1H), 1.63 (s, 2H), 0.92 (s, 3H); **^{13}C NMR (126 MHz, $CDCl_3$)** δ 202.45, 172.29, 137.51, 128.58, 128.02, 127.71, 73.32, 69.41, 68.78, 55.16, 44.40, 37.46, 16.77, 13.51; **FTIR** (Neat, cm^{-1}): *v* 2964, 2873, 1770, 1716; **HRMS** m/z calculated for $C_{16}H_{20}O_4$ $[M+H]^+$: 277.14344, found $[M+H]^+$: 277.14295. (-1.76ppm)

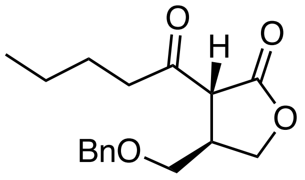

(3*R*,4*R*)-4-((benzyloxy)methyl)-3-pentanoyldihydrofuran-2(3*H*)-one (**5d**, protected keto-VB-S4)

Synthesized using pentanoyl chloride and compound **1** via acylation procedure 1 to afford compound **5d** (60% yield) as a clear, colorless oil. **TLC** R_f = 0.33 (20% EtOAc in hexanes, $KMnO_4$); **Alpha D** $[\alpha]_D^{21}: -10.4°$ (*c* 1.0mg/ml, $CHCl_3$); **^{1}H NMR (500 MHz, $CDCl_3$)** δ 7.38 – 7.27 (m, 5H), 4.51 (s, 2H), 4.40 (t, *J* = 9.0 Hz, 1H), 4.14 (dd, *J* = 8.9, 6.5 Hz, 1H), 3.65 (d, *J* = 6.9 Hz, 1H), 3.48 (d, *J* = 5.2 Hz, 2H), 3.30 (dtt, *J* = 8.2, 6.8, 5.2 Hz, 1H), 2.94 (dt, *J* = 17.8, 7.4 Hz, 1H), 2.58 (dt, *J* = 17.9, 7.2 Hz, 1H), 1.62 – 1.53 (m, 2H), 1.37 – 1.28 (m, 2H), 0.91 (t, *J* = 7.4 Hz, 3H); **^{13}C NMR (126 MHz, $CDCl_3$)** δ 202.59, 172.30, 137.51, 128.57, 128.01, 127.71, 73.32, 69.42, 68.79, 55.15, 42.28, 37.48, 25.37, 22.13, 13.84.; **FTIR** (Neat, cm^{-1}): *v* 2958, 2931, 2871, 1770, 1718; **HRMS**: m/z calculated for $C_{17}H_{22}O_4$ $[M+H]^+$:291.15909, found $[M+H]^+$:291.15819. (-3.09ppm)

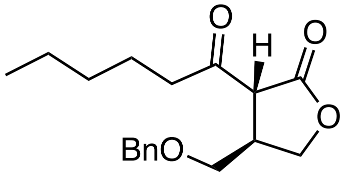

(3*R*,4*R*)-4-((benzyloxy)methyl)-3-hexanoyldihydrofuran-2(3*H*)-one (**5e**, protected keto-VB-C)

Synthesized using hexanoyl chloride and compound **1** via acylation procedure 1 to afford compound **5e** (57% yield) as a clear, colorless oil.

TLC R_f = 0.32 (20% EtOAc in hexanes, $KMnO_4$); **Alpha D** $[\alpha]_D^{21}: -9.2°$ (*c* 1.0mg/ml, $CHCl_3$); **^{1}H NMR (500 MHz, $CDCl_3$)** δ 7.26-7.38 (m, *J* = 35.8 Hz, 5H), 4.51 (s, 2H), 4.41 (t, 1H), 4.15 (s, 1H), 3.64 (s, 1H), 3.47 (s, 2H), 3.29 (s, 1H), 2.92 (s, 1H), 2.59 (s, 1H), 1.60 (s, 2H), 1.30 (s, 5H), 0.89 (s, 3H); **^{13}C NMR (126 MHz, $CDCl_3$)** δ 202.60, 172.29, 137.51, 128.58, 128.01, 127.70, 73.32, 69.42, 68.80, 55.15, 42.53, 37.48, 31.14, 22.97, 22.43, 13.91; **FTIR** (Neat, cm^{-1}) *v* 2971, 2912, 1776, 1726; **HRMS** m/z calculated for $C_{18}H_{24}O_4$ $[M+H]^+$:305.17474, found $[M+H]^+$:305.17324. (-4.91ppm)

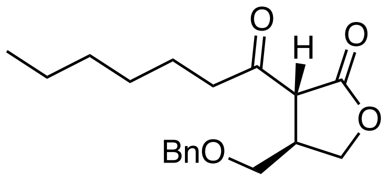

(3*R*,4*R*)-4-((benzyloxy)methyl)-3-heptanoyldihydrofuran-2(3*H*)-one (**5f**, protected keto-VB-D)

Synthesized using heptanoyl chloride and compound **1** via acylation procedure 1 to afford compound **5f** (53% yield) as a clear, colorless oil.

TLC R_f = 0.40 (20% EtOAc in hexanes, $KMnO_4$); **Alpha D** $[\alpha]_D^{21}: -10.0°$ (*c* 1.0mg/ml, $CHCl_3$); **^{1}H NMR (500 MHz, $CDCl_3$)** δ 7.38 – 7.27 (m, 5H), 4.51 (s, 2H), 4.41 (t, *J* = 8.5 Hz, 1H), 4.15 (dd, *J* = 6.5, 2.4 Hz, 1H), 3.64 (d, *J* = 6.9 Hz, 1H), 3.47 (d, *J* = 5.4 Hz, 2H), 3.35 – 3.26 (m, 1H), 2.92 (dd, *J* = 7.6, 2.7 Hz, 1H), 1.63 – 1.56 (m, 2H), 1.35 – 1.23 (m, 7H), 0.92 – 0.85 (m, 3H); **^{13}C NMR (126 MHz, $CDCl_3$)** δ 202.61, 172.30, 137.51, 128.58, 128.01, 127.70, 73.32, 69.42, 68.80, 55.14, 42.57, 37.47, 31.57, 28.66, 23.26, 22.48, 14.03; **FTIR** (Neat, cm^{-1}): *v* 2954, 2931, 2861, 1776, 1720; **HRMS** m/z calculated for $C_{19}H_{26}O_4$ $[M+H]^+$:318.1831, found $[M+H]^+$:318.1800. (-1.0ppm)

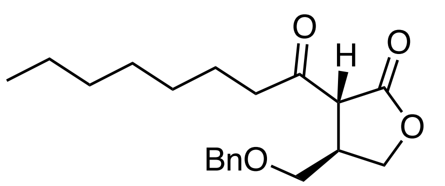

(3*R*,4*R*)-4-((benzyloxy)methyl)-3-octanoyldihydrofuran-2(3*H*)-one (**5g**, protected keto-VB-S5)
Synthesized using octanoyl chloride and compound **1** via acylation procedure 1 to afford compound **5g** (47% yield) as a clear, colorless oil.
TLC R_f = 0.41 (20% EtOAc in hexanes, $KMnO_4$); **Alpha D** $[\alpha]_D^{21}: -20.8°$ (*c* 1.0mg/ml, $CHCl_3$); **^{1}H NMR (500 MHz, $CDCl_3$)** δ 7.38 – 7.27 (m, 5H), 4.51 (s, 2H), 4.41 (t, *J* = 8.6 Hz, 1H), 4.15 (dd, *J* = 6.8, 2.4 Hz, 1H), 3.64 (d, *J* = 7.0 Hz, 1H), 3.47 (dd, *J* = 163.0, 5.0 Hz, 2H), 3.34 – 3.26 (m, 1H), 2.91 (td, *J* = 7.0, 3.5 Hz, 1H), 2.60 (dd, *J* = 7.7, 3.1 Hz, 1H), 1.64 – 1.56 (m, 2H), 1.34 – 1.20 (m, 10H), 0.91 – 0.85 (m, 4H); **^{13}C NMR (126 MHz, $CDCl_3$)** δ 202.61, 172.30, 137.51, 128.58, 128.01, 127.70, 73.32, 69.42, 68.80, 55.15, 42.58, 37.48, 31.67, 29.05, 28.95, 23.30, 22.61, 14.08; **FTIR** (Neat, cm^{-1}): *v* 2964, 2868, 1776, 1722; **HRMS** m/z calculated for $C_{20}H_{28}O_4$ $[M+H]^+$:333.20604, found $[M+H]^+$:333.20483. (-3.63ppm)

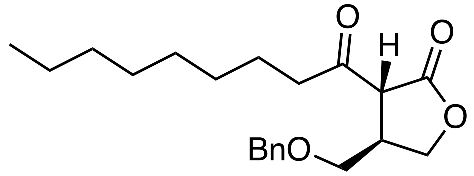

(3*R*,4*R*)-4-((benzyloxy)methyl)-3-nonanoyldihydrofuran-2(3*H*)-one (**5h**, protected keto-VB-S6)
Synthesized using nonanoyl chloride and compound **1** via acylation procedure 1 to afford compound **5h** (60% yield) as a clear, colorless oil.
TLC R_f = 0.20 (10% EtOAc in hexanes, $KMnO_4$); **Alpha D** $[\alpha]_D^{21}: -43.6°$ (*c* 1.0mg/ml, $CHCl_3$); **^{1}H NMR (500 MHz, $CDCl_3$)** δ 7.39 – 7.26 (m, 5H), 4.51 (s, 2H), 4.41 (t, *J* = 8.6 Hz, 1H), 4.15 (dd, *J* = 8.8, 6.5 Hz, 1H), 3.64 (d, *J* = 6.5 Hz, 1H), 3.47 (d, *J* = 5.1 Hz, 2H), 3.34 – 3.26 (m, 1H), 2.91 (dd, *J* = 17.8, 7.4 Hz, 1H), 2.59 (dd, *J* = 17.7, 7.4 Hz, 1H), 1.34 – 1.20 (m, 13H), 0.91 – 0.84 (m, 4H); **^{13}C NMR (126 MHz, $CDCl_3$)** δ 202.61, 172.30, 137.51, 128.58, 128.01, 127.70, 73.32, 69.42, 68.80, 55.15, 42.58, 37.48, 31.83, 29.35, 29.13, 29.00, 23.30, 22.66, 14.11; **FTIR** (Neat, cm^{-1}): *v* 2954, 2972, 2856, 1774, 1720; **HRMS** m/z calculated for $C_{21}H_{30}O_4$ $[M+H]^+$:347.22169, found $[M+H]^+$:347.22063. (-3.05ppm)

Acylation Procedure 2

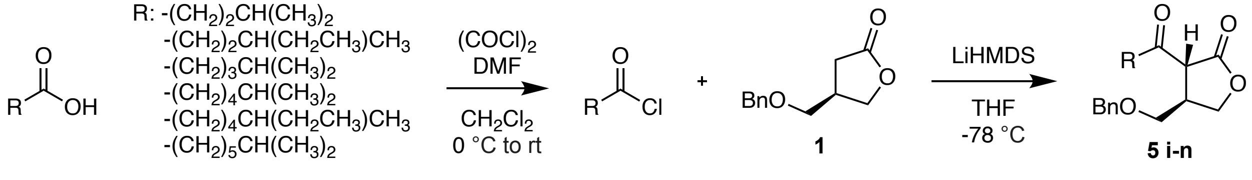

A solution of carboxylic acid (2.5 equiv.) in CH_2Cl_2 (0.4M reaction) was cooled to 0 °C. To the solution, DMF (0.13 equiv.) was added, followed by dropwise addition of oxalyl chloride (2.8 equiv.). After 30 minutes, the reaction was slowly brought to room temperature, and the reaction was allowed to stir for another 1.5 hours. The reaction was concentrated under a stream of nitrogen until the solvent had been removed. The resulting oil was dissolved in THF (0.5 mL) and then added dropwise to a pre-stirred for 1.5 hours solution of lactone **1** (1.0 equiv.) and LiHMDS (2.5 equiv., 1.0M in THF) in THF (0.1M reaction) at -78 °C. The reaction was then allowed to proceed for two hours at -78 °C. Once complete according to TLC, the reaction was quenched with saturated aqueous NH_4Cl (10 mL) and then extracted with EtOAc (3 x 15 mL). The organic layers were then combined, dried with Na_2SO_4, filtered and concentrated. The resulting oil was purified by flash chromatography. Final products were single diastereomers, as observed by NMR.

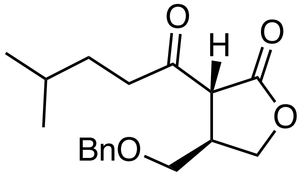

(3*R*,4*R*)-4-((benzyloxy)methyl)-3-(4-methylpentanoyl)dihydrofuran-2(3*H*)-one (**5i**, protected keto-VB-E)

Synthesized using 4-methylpentanoic acid and compound **1** via acylation procedure 2 to afford compound **5i** (53% yield) as a clear, colorless oil.

TLC R_f = 0.38 (20% EtOAc in hexanes, $KMnO_4$); **Alpha D** $[\alpha]_D^{21}: -19.0°$ (*c* 1.0mg/ml, $CHCl_3$); **^{1}H NMR (500 MHz, $CDCl_3$)** δ 7.38 – 7.26 (m, 5H), 4.51 (s, 2H), 4.41 (dd, *J* = 8.9, 8.1 Hz, 1H), 4.15 (dd, *J* = 8.9, 6.5 Hz, 1H), 3.67 (d, *J* = 6.9 Hz, 1H), 3.48 (d, *J* = 5.2 Hz, 2H), 3.33 – 3.26 (m, 1H), 2.98 – 2.90 (m, 1H), 2.63 – 2.54 (m, 1H), 1.63 – 1.44 (m, 4H), 0.92 – 0.86 (m, 7H); **^{13}C NMR (126 MHz, $CDCl_3$)** δ 202.72, 172.31, 137.51, 128.58, 128.02, 127.71, 73.32, 69.41, 68.79, 55.15, 40.66, 37.49, 32.03, 27.57, 22.45, 22.23; **FTIR** (Neat, cm^{-1}): *v* 2956, 2927, 2869, 1770, 1716; **HRMS** m/z calculated for $C_{18}H_{24}O_4$ $[M+H]^+$:305.17474, found $[M+H]^+$:305.17364. (-3.60ppm)

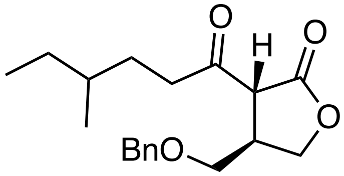

(3*R*,4*R*)-4-((benzyloxy)methyl)-3-(4-methylhexanoyl)dihydrofuran-2(3*H*)-one (**5j**, protected keto-VB-B)

Synthesized using racemic 4-methylhexanoic acid and compound **1** via acylation procedure 2 to afford compound **5j** (77% yield) as a clear, colorless oil.

TLC R_f = 0.34 (20% EtOAc in hexanes, $KMnO_4$); **Alpha D** $[\alpha]_D^{21}: -11.8°$ (*c* 1.0mg/ml, $CHCl_3$); **^{1}H NMR (500 MHz, $CDCl_3$)** δ 7.39 – 7.25 (m, 6H), 4.51 (s, 2H), 4.41 (t, *J* = 8.8 Hz, 2H), 4.15 (dd, *J* = 8.9, 6.5 Hz, 1H), 3.67 (dd, *J* = 6.9, 1.4 Hz, 1H), 3.48 (d, *J* = 5.2 Hz, 2H), 3.34 – 3.25 (m, 1H), 3.02 – 2.86 (m, 1H), 2.65 – 2.51 (m, 1H), 1.70 – 1.57 (m, 1H), 1.47 – 1.22 (m, 3H), 1.22 – 1.10 (m, 1H), 0.92 – 0.79 (m, 7H); **^{13}C NMR (126 MHz, $CDCl_3$)** δ 202.81, 172.31, 137.51, 128.58, 128.01, 127.70, 73.32, 69.42, 68.80, 55.20, 55.13, 40.42, 37.51, 37.47, 33.93, 33.88, 29.77, 29.34, 29.11, 19.00, 18.89, 11.33, 11.29; **FTIR** (Neat, cm^{-1}): *v* 2960, 2933, 2873, 1770, 1720; **HRMS** m/z calculated for $C_{19}H_{26}O_4$ $[M+H]^+$:319.19039, found $[M+H]^+$:319.1892. (-3.72ppm)

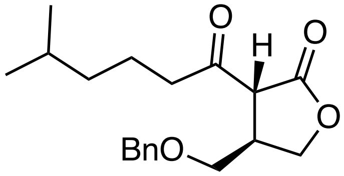

(3*R*,4*R*)-4-((benzyloxy)methyl)-3-(5-methylhexanoyl)dihydrofuran-2(3*H*)-one (**5k**, protected keto-VB-A)

Synthesized using 5-methylhexanoic acid and compound **1** via acylation procedure 2 to afford compound **5k** (69% yield) as a clear, colorless oil.

TLC R_f = 0.30 in (20% EtOAc in Hexanes); **Alpha D** $[\alpha]_D^{21}: -18.0°$ (*c* 1.0mg/ml, $CHCl_3$); **^{1}H NMR (500 MHz, $CDCl_3$)** δ 7.39 – 7.25 (m, 5H), 4.51 (s, 2H), 4.41 (t, *J* = 8.9 Hz, 1H), 4.14 (dd, *J* = 8.9, 6.5 Hz, 1H), 3.65 (d, *J* = 6.8 Hz, 1H), 3.48 (d, *J* = 5.2 Hz, 2H), 3.34 – 3.25 (m, 1H), 2.93 (dt, *J* = 17.9, 7.4 Hz, 1H), 2.56 (dt, *J* = 17.9, 7.2 Hz, 1H), 1.66 – 1.47 (m, 4H), 1.22 – 1.12 (m, 2H), 0.92 – 0.83 (m, 6H); **^{13}C NMR (126 MHz, $CDCl_3$)** δ 202.59, 172.29, 137.51, 128.58, 128.02, 127.70, 73.32, 69.42, 68.79, 55.15, 42.78, 38.18, 37.48, 27.85, 22.50, 22.45, 21.15; **FTIR** (Neat, cm^{-1}): *v* 2954, 2902, 2869, 1770, 1718; **HRMS** m/z calculated for $C_{19}H_{26}O_4$ $[M+H]^+$:319.19039, found $[M+H]^+$:319.18966. (-2.28ppm)

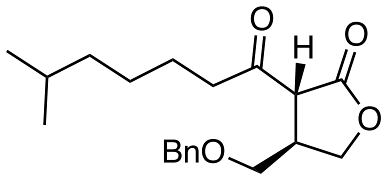

(3*R*,4*R*)-4-((benzyloxy)methyl)-3-(6-methylheptanoyl)dihydrofuran-2(3*H*)-one (**5l**, protected keto-A-factor)

Synthesized using 6-methylheptanoic acid and compound **1** via acylation procedure 2 to afford compound **5l** (52% yield) as a clear, colorless oil.

TLC R_f = 0.41 in (20% EtOAc in Hexanes); **Alpha D** $[\alpha]_D^{21}: -13.7°$ (*c* 1.9mg/ml, $CHCl_3$); **^{1}H NMR (500 MHz, $CDCl_3$)** δ 7.39 – 7.27 (m, 5H), 4.51 (s, 2H), 4.41 (t, *J* = 8.5 Hz, 1H), 4.15 (dd, *J* = 9.1, 6.3 Hz, 1H), 3.64 (d, *J* = 6.9 Hz, 1H), 3.47 (d, *J* = 5.3 Hz, 2H), 3.35 – 3.25 (m, 1H), 2.99 – 2.89 (m, 1H), 2.63 – 2.53 (m, 1H), 1.64 – 1.48 (m, 4H), 1.35 – 1.15 (m, 8H), 0.87 – 0.85 (m, 7H); **^{13}C NMR (126 MHz, $CDCl_3$)** δ 202.59, 172.30, 137.51, 128.58, 128.02, 127.70, 73.32, 69.42, 68.79, 55.14, 42.60, 38.68, 37.47, 27.82, 26.77, 23.53, 22.59; **FTIR** (Neat, cm^{-1}): *v* 2955, 2935, 2867, 1776, 1722; **HRMS** m/z calculated for $C_{20}H_{28}O_4$ $[M]^+$:332.198, found $[M]^+$:332.197. (-5.2ppm)

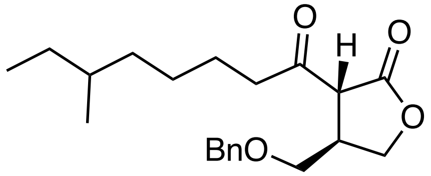

(3*R*,4*R*)-4-((benzyloxy)methyl)-3-(6-methyloctanoyl)dihydrofuran-2(3*H*)-one (**5m**, protected keto-VB-S8)
Synthesized using racemic 6-methyloctanoic acid and compound **1** via acylation procedure 2 to afford compound **5m** (70% yield) as a clear, colorless oil.

TLC R_f = 0.20 in (10% EtOAc in Hexanes); **Alpha D** $[\alpha]_D^{21}: -12.7°$ (*c* 4.2mg/ml, $CHCl_3$); **^{1}H NMR (500 MHz, $CDCl_3$)** δ 7.38 – 7.27 (m, 6H), 4.51 (s, 2H), 4.41 (d, *J* = 8.5 Hz, 1H), 4.15 (dd, *J* = 8.8, 6.5 Hz, 1H), 3.64 (d, *J* = 6.8 Hz, 1H), 3.47 (d, *J* = 5.1 Hz, 2H), 3.34 – 3.26 (m, 1H), 2.98 – 2.89 (m, 1H), 2.63 – 2.54 (m, 1H), 2.39 – 2.32 (m, 1H), 1.67 – 1.50 (m, 3H), 1.30 (s, 8H), 1.17 – 1.06 (m, 3H), 0.84 (s, 6H); **^{13}C NMR (126 MHz, $CDCl_3$)** δ 202.60, 172.30, 137.51, 128.58, 128.01, 127.70, 73.32, 69.43, 68.80, 55.14, 42.61, 37.47, 36.32, 34.23, 29.44, 26.49, 23.62, 19.16, 11.39; **FTIR** (Neat, cm^{-1}): *v* 2958, 2927, 2871, 1776, 1722; **HRMS** m/z calculated for $C_{21}H_{30}O_4$ $[M]^+$:346.2144, found $[M]^+$:346.2126. (-5.1ppm)

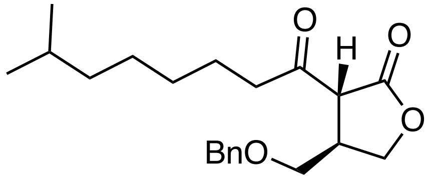

(3*R*,4*R*)-4-((benzyloxy)methyl)-3-(7-methyloctanoyl)dihydrofuran-2(3*H*)-one (**5n**, protected keto-VB-9)
Synthesized using 7-methyloctanoic acid and compound **1** via acylation procedure 2 to afford compound **5n** (64% yield) as a clear, colorless oil.

TLC R_f = 0.19 in (10% EtOAc in Hexanes); **Alpha D** $[\alpha]_D^{21}: -12.5°$ (*c* 1.3mg/ml, $CHCl_3$); **^{1}H NMR (500 MHz, $CDCl_3$)** δ 7.38 – 7.26 (m, 5H), 4.51 (s, 2H), 4.41 (t, *J* = 8.5 Hz, 1H), 4.18 – 4.08 (m, 2H), 3.65 (d, *J* = 6.9 Hz, 1H), 3.48 (d, *J* = 5.2 Hz, 2H), 3.35 – 3.25 (m, 1H), 2.93 (dt, *J* = 17.7, 7.5 Hz, 1H), 2.58 (dt, *J* = 17.8, 7.2 Hz, 1H), 1.64 – 1.45 (m, 3H), 1.27 (dq, *J* = 10.6, 5.4 Hz, 8H), 1.15 (s, 1H), 0.86 (d, *J* = 6.6 Hz, 6H); **^{13}C NMR (126 MHz, $CDCl_3$)** δ 202.59, 172.29, 137.51, 128.58, 128.01, 127.70, 73.32, 69.42, 68.80, 55.15, 42.58, 38.79, 37.47, 29.25, 27.93, 27.15, 23.33, 22.63; **FTIR** (Neat, cm^{-1}) *v* 2954, 2929, 2867, 1778, 1722; **HRMS** m/z calculated for $C_{21}H_{30}O_4$ $[M]^+$:346.2144, found $[M]^+$:346.2122. (-6.3ppm)

General Method for Keto Deprotection to Access A-factor Derivatives

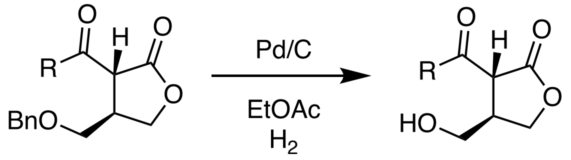

5a - n **6a - n**

Activated Pd/C (10 wt %) (0.20 equiv.) was added to a flame dried round bottom flask under a nitrogen atmosphere at room temperature. Benzyl protected A-factor type molecule in EtOAc

(0.01M) was added to the round bottom flask, where the solution was sparged with N_2 for ten minutes. The solution was sparged with H_2 for twenty minutes. The reaction then stirred overnight under H_2. The reaction was filtered using a silica plug to remove palladium. The resulting filtrate was concentrated and purified via flash chromatography to afford the A-factor type molecules.

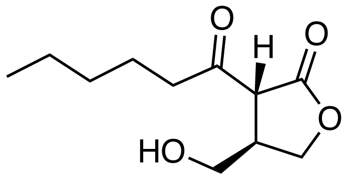

(3*R*,4*R*)-3-hexanoyl-4-(hydroxymethyl)dihydrofuran-2(3*H*)-one (**6e**, keto-VB-C)
This compound was synthesized via known protocol, with ^{1}H NMR data corresponding with the literature values.

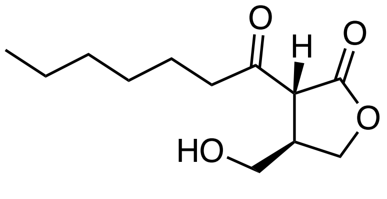

(3*R*,4*R*)-3-heptanoyl-4-(hydroxymethyl)dihydrofuran-2(3*H*)-one (**6f**, keto-VB-D)
This compound was synthesized via known protocol, with ^{1}H NMR data corresponding with the literature values.

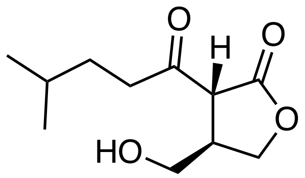

(3*R*,4*R*)-4-(hydroxymethyl)-3-(4-methylpentanoyl)dihydrofuran-2(3*H*)-one (**6i**, keto-VB-E)
Synthesized via the general deprotection protocol to afford compound **6e** (80% yield), along with hemiketal[9] (4:1 keto:hemiketal in $CDCl_3$), as a colorless oil, from compound **5i**.
TLC R_f = 0.23 (50% EtOAc in hexanes, $KMnO_4$); **Alpha D** $[\alpha]_D^{21}: -3.1°$ (*c* 1.5mg/ml, $CHCl_3$); **^{1}H NMR (500 MHz, $CDCl_3$)** δ 4.44 (dd, *J* = 9.0, 8.3 Hz, 1H), 4.15 (dd, *J* = 9.1, 6.7 Hz, 1H), 3.77 – 3.64 (m, 3H), 3.31 – 3.20 (m, 1H), 3.04 – 2.93 (m, 1H), 2.71 – 2.60 (m, 1H), 1.95 – 1.84 (m, 1H), 1.62 – 1.48 (m, 5H), 0.95 – 0.88 (m, 9H); **^{13}C NMR (126 MHz, $CDCl_3$)** δ 202.97, 172.24, 68.94, 61.92, 54.95, 40.57, 39.09, 32.05, 27.58, 22.45, 22.24; **FTIR** (Neat, cm^{-1}): *v* 3444, 2958, 2927, 2871, 1764, 1716; **HRMS** m/z calculated for $C_{11}H_{18}O_4$ $[M]^+$: 214.1205, found $[M]^+$: 214.1186. (-8.7ppm)

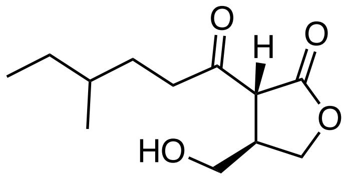

(3*R*,4*R*)-4-(hydroxymethyl)-3-(4-methylhexanoyl)dihydrofuran-2(3*H*)-one (**6j**, keto-VB-B)
This compound was synthesized via known protocol, with ^{1}H NMR data corresponding with the literature values.

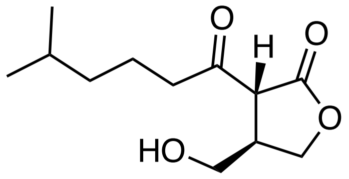

(3*R*,4*R*)-4-(hydroxymethyl)-3-(5-methylhexanoyl)dihydrofuran-2(3*H*)-one (**6k**, keto-VB-A)
This compound was synthesized via known protocol, with ^{1}H NMR data corresponding with the literature values.

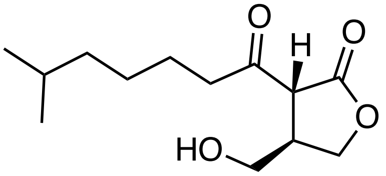

(3*R*,4*R*)-4-(hydroxymethyl)-3-(7-methyloctanoyl)dihydrofuran-2(3*H*)-one (**6l**, A-Factor)
This compound was synthesized via known protocol, with ^{1}H NMR data corresponding with the literature values.

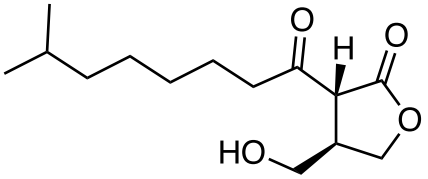

(3*R*,4*R*)-4-(hydroxymethyl)-3-(7-methyloctanoyl)dihydrofuran-2(3*H*)-one (**6n**, keto-VB-S9)
Synthesized via the general deprotection protocol to afford compound **6g** (55% yield) along with hemiketal (5:1 keto:hemiketal in $CDCl_3$), as a colorless oil, from compound **5n**.
TLC R_f = 0.30 (50% EtOAc in hexanes, $KMnO_4$); **Alpha D** $[\alpha]_D^{21}: -7.7°$ (*c* 6.4mg/ml, $CHCl_3$); **^{1}H NMR (500 MHz, $CDCl_3$)** δ 4.44 (t, *J* = 8.5 Hz, 1H), 4.15 (dd, *J* = 8.8, 6.7 Hz, 1H), 3.77 – 3.65 (m, 3H), 3.30 – 3.21 (m, 1H), 3.02 – 2.93 (m, 1H), 2.69 – 2.59 (m, 1H), 1.92 – 1.82 (m, 1H), 1.69 – 1.58 (m, 4H), 1.53 – 1.46 (m, 3H), 1.35 – 1.24 (m, 11H), 0.86 (d, *J* = 6.6 Hz, 12H); **^{13}C NMR (126 MHz, $CDCl_3$)** δ 202.87, 172.25, 68.95, 61.94, 54.96, 42.50, 39.09, 38.79, 29.26, 27.92, 27.15, 23.35, 22.63; **FTIR** (Neat, cm^{-1}): *v* 3432, 2952, 2925, 2867, 1764, 1714; **HRMS** m/z calculated for $C_{14}H_{24}O_4$ $[M]^+$: 256.1675, found $[M]^+$: 256.1663. (-4.4ppm)

General Method for Noyori Hydrogenation

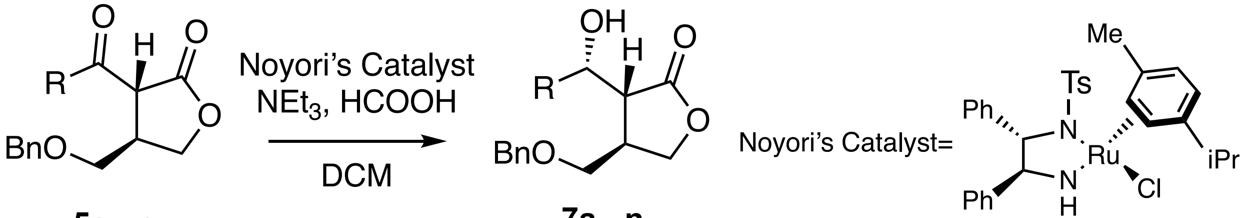

5a - n **7a - n**

A solution of protected *β*-keto ester in DCM (0.1M) was added to the RuCl(p-cymene)[(*S*,*S*)-Ts-DPEN] (0.05 equiv.), in a flame dried round bottom flask. A solution of triethylamine (2 equiv.) and formic acid (5 equiv.) in 0.5 mL of DCM was then added dropwise to the round bottom flask. The reaction stirred at room temperature for 36 hours. The reaction was then quenched with aqueous NaH_4Cl (10 mL) and extracted with EtOAc (3 x 15 mL). The combined organic layers were then dried with Na_2SO_4, filtered and concentrated. The resulting oil was the purified via flash chromatography to afford one diastereomer, as determined via NMR.

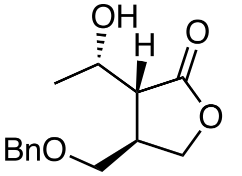

(3*R*,4*R*)-4-((benzyloxy)methyl)-3-((*S*)-1-hydroxyethyl)dihydrofuran-2(3*H*)-one (**7a**, protected VB-S1)
Synthesized via the general protocol of the Noyori Hydrogenation to afford compound **7a** (77% yield) as a colorless oil, from compound **5a**.
TLC R_f = 0.18 (40% EtOAc in hexanes, $KMnO_4$); **Alpha D** $[\alpha]_D^{21}: -21.6°$ (*c* 1.7mg/ml, $CHCl_3$); **^{1}H NMR (500 MHz, $CDCl_3$)** δ 7.39 – 7.28 (m, 4H), 4.60 – 4.48 (m, 2H), 4.41 (d, *J* = 8.7 Hz, 1H), 4.29 (pd, *J* = 6.3, 3.8 Hz, 1H), 4.16 – 4.02 (m, 1H), 3.53 (qd, *J* = 9.4, 5.8 Hz, 2H), 2.98 – 2.80 (m, 1H), 2.55 (dd, *J* = 7.7, 3.9 Hz, 1H), 2.28 (d, *J* = 6.0 Hz, 1H), 1.35 – 1.21 (m, 3H); **^{13}C NMR (126 MHz, $CDCl_3$)** δ 178.10, 137.55, 128.57, 127.99, 127.70, 73.41, 70.39, 69.62, 66.71, 49.27, 36.66, 20.84; **FTIR** (Neat, cm^{-1}): *v* 3457, 2971, 2913, 2865, 1758; **HRMS** m/z calculated for $C_{14}H_{18}O_4$ $[M]^+$:250.1205, found $[M]^+$: 250.1188. (-6.9ppm)

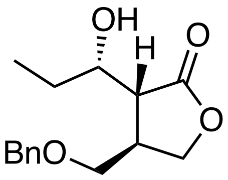

(3*R*,4*R*)-4-((benzyloxy)methyl)-3-((*S*)-1-hydroxypropyl)dihydrofuran-2(3*H*)-one (**7b**, protected VB-S2)
Synthesized via the general protocol of the Noyori Hydrogenation to afford compound **7b** (78% yield) as a colorless oil, from compound **5b**.
TLC R_f = 0.35 (40% EtOAc in hexanes, $KMnO_4$); **Alpha D** $[\alpha]_D^{21}: -31.2°$ (*c* 2.5mg/ml, $CHCl_3$); **^{1}H NMR (500 MHz, $CDCl_3$)** δ 7.40 – 7.24 (m, 5H), 4.52 (d, *J* = 5.5 Hz, 2H), 4.40 (t, *J* = 8.7 Hz, 1H), 4.16 – 4.06 (m, 1H), 4.09 – 4.01 (m, 1H), 3.57 – 3.46 (m, 2H), 2.94 (tt, *J* = 13.8, 6.0 Hz, 1H), 2.60 (dd, *J* = 7.3, 3.2 Hz, 1H), 2.08 (d, *J* = 5.3 Hz, 1H), 1.67 – 1.51 (m, 2H), 1.03 – 0.94 (m, 3H); **^{13}C NMR (126 MHz, $CDCl_3$)** δ 178.59, 137.60, 128.55, 127.97, 127.71, 73.39, 72.24, 70.39, 69.81, 47.72, 35.92, 27.80, 10.33; **FTIR** (Neat, cm^{-1}): *v* 3467, 2964, 2921, 2875, 1764; **HRMS** m/z calculated for $C_{15}H_{20}O_4$ $[M]^+$:264.1362, found $[M]^+$: 264.1344. (-6.7ppm)

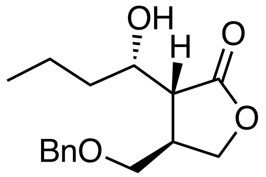

(3*R*,4*R*)-4-((benzyloxy)methyl)-3-((*S*)-1-hydroxybutyl)dihydrofuran-2(3*H*)-one (**7c**, protected VB-S3)
Synthesized via the general protocol of the Noyori Hydrogenation to afford compound **7c** (80% yield) as a colorless oil, from compound **5c**.
TLC R_f = 0.32 (40% EtOAc in hexanes, $KMnO_4$); **Alpha D** $[\alpha]_D^{21}: -29.3°$ (*c* 3.2mg/ml, $CHCl_3$); **^{1}H NMR** (500 MHz, $CDCl_3$) δ 7.40 – 7.25 (m, 5H), 4.59 – 4.46 (m, 2H), 4.40 (t, *J* = 8.7 Hz, 1H), 4.21 – 4.11 (m, 1H), 4.13 – 4.05 (m, 1H), 3.51 (qd, *J* = 9.3, 5.7 Hz, 2H), 2.93 (dddd, *J* = 13.6, 8.5, 6.7, 5.1 Hz, 1H), 2.58 (dd, *J* = 7.4, 3.2 Hz, 1H), 2.08 (d, *J* = 5.5 Hz, 1H), 1.65 – 1.23 (m, 4H), 0.98 – 0.88 (m, 3H); **^{13}C NMR** (126 MHz, $CDCl_3$) δ 178.61, 137.60, 128.55, 127.97, 127.72, 73.38, 70.42, 70.35, 69.82, 48.06, 36.84, 35.89, 19.11, 13.87; FTIR (Neat, cm^{-1}): *v* 3467, 2958, 2871, 1764; **HRMS** m/z calculated for $C_{16}H_{22}O_4$ $[M]^+$:278.1518, found $[M]^+$:278.1516. (-0.7ppm)

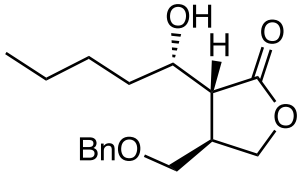

(3*R*,4*R*)-4-((benzyloxy)methyl)-3-((*S*)-1-hydroxypentyl)dihydrofuran-2(3*H*)-one (**7d**, protected VB-S4)
Synthesized via the general protocol of the Noyori Hydrogenation to afford compound **7d** (80% yield) as a colorless oil, from compound **5d**.
TLC R_f = 0.36 (40% EtOAc in hexanes, $KMnO_4$); **Alpha D** $[\alpha]_D^{21}: -42.8°$ (*c* 2.0mg/ml, $CHCl_3$); **^{1}H NMR (500 MHz, $CDCl_3$)** δ 7.40 – 7.27 (m, 5H), 4.58 – 4.49 (m, 2H), 4.40 (t, *J* = 9.1 Hz, 1H), 4.16 – 4.12 (m, 1H), 4.10 (dd, *J* = 8.9, 6.8 Hz, 1H), 3.56 – 3.47 (m, 2H), 2.97 – 2.88 (m, 1H), 2.60 (dd, *J* = 7.3, 3.2 Hz, 1H), 2.05 (d, *J* = 5.4 Hz, 1H), 1.62 – 1.23 (m, 7H), 0.94 – 0.86 (m, 3H); **^{13}C NMR (126 MHz, $CDCl_3$)** δ 178.60, 137.60, 128.55, 127.97, 127.71, 73.38, 70.72, 70.38, 69.82, 48.03, 35.90, 34.44, 28.03, 22.49, 14.00; **FTIR** (Neat, cm^{-1}): *v* 3469, 2954, 2929, 2859, 1764; **HRMS** m/z calculated for $C_{17}H_{24}O_4$ $[M]^+$:292.1675, found $[M+H]^+$:292.1661. (-4.4ppm)

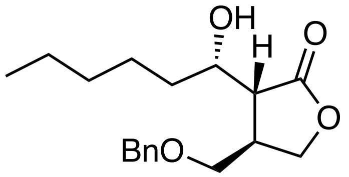

(3*R*,4*R*)-4-((benzyloxy)methyl)-3-((*S*)-1-hydroxyhexyl)dihydrofuran-2(3*H*)-one (**7e**, protected VB-C)
Synthesized via the general protocol of the Noyori Hydrogenation to afford compound **7e** (67% yield) as a colorless oil, from compound **5e**.
TLC R_f = 0.40 (40% EtOAc in hexanes, $KMnO_4$); **Alpha D** $[\alpha]_D^{21}: -31.0°$ (*c* 1.9mg/ml, $CHCl_3$); **^{1}H NMR (500 MHz, $CDCl_3$)** δ 7.38 – 7.27 (m, 5H), 4.58 – 4.48 (m, 2H), 4.39 (t, *J* = 8.7 Hz, 1H), 4.16 – 4.12 (m, 1H), 4.10 (dd, *J* = 8.9, 6.8 Hz, 1H), 3.56 – 3.46

(m, 2H), 2.98 – 2.87 (m, 1H), 2.59 (dd, *J* = 7.3, 3.2 Hz, 1H), 2.11 – 2.03 (m, 1H), 1.63 – 1.42 (m, 4H), 1.36 – 1.17 (m, 7H), 0.93 – 0.85 (m, 4H); **^{13}C NMR (126 MHz, $CDCl_3$)** δ 179.52, 138.52, 129.46, 128.88, 128.62, 74.29, 71.64, 71.30, 70.74, 48.96, 36.81, 35.63, 32.52, 26.48, 23.48, 14.94; **FTIR**(Neat, cm^{-1}): *v* 3453, 2954, 2935, 2859, 1764; **HRMS** m/z calculated for $C_{18}H_{26}O_4$ $[M]^+$:306.1831, found $[M]^+$:306.1823. (-2.7ppm)

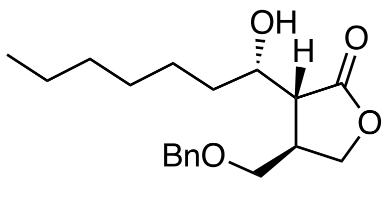

(3*R*,4*R*)-4-((benzyloxy)methyl)-3-((*S*)-1-hydroxyheptyl)dihydrofuran-2(3*H*)-one (**7f**, protected VB-D)
Synthesized via the general protocol of the Noyori Hydrogenation to afford compound **7f** (57% yield) as a colorless oil, from compound **5f**.
TLC R_f = 0.44 (40% EtOAc in hexanes, $KMnO_4$); **Alpha D** $[\alpha]_D^{21}: -2.4°$ (*c* 6.6mg/ml, $CHCl_3$); **^{1}H NMR (500 MHz, $CDCl_3$)** δ 7.39 – 7.28 (m, 4H), 4.58 – 4.48 (m, 2H), 4.39 (t, *J* = 8.9 Hz, 1H), 4.17 – 4.12 (m, 1H), 4.10 (dd, *J* = 8.9, 6.8 Hz, 1H), 3.55 – 3.49 (m, 2H), 2.97 – 2.88 (m, 1H), 2.60 (dd, *J* = 7.0, 3.4 Hz, 1H), 2.04 (d, *J* = 5.5 Hz, 1H), 1.60 – 1.24 (m, 11H), 0.93 – 0.85 (m, 3H); **^{13}C NMR (126 MHz, $CDCl_3$)** δ 178.59, 137.60, 128.55, 127.97, 127.71, 73.38, 70.73, 70.38, 69.81, 65.87, 48.03, 35.90, 34.76, 31.75, 29.10, 25.86, 22.60, 15.30, 14.07; **FTIR** (Neat, cm^{-1}): *v* 3409, 2925, 2856, 1764; **HRMS** m/z calculated for $C_{19}H_{28}O_4$ $[M+H]^+$:320.1988, found $[M]^+$: 320.1974. (-4.1ppm)

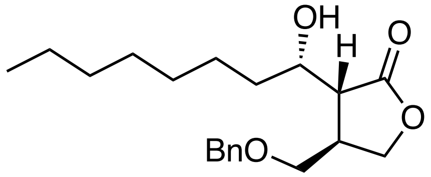

(3*R*,4*R*)-4-((benzyloxy)methyl)-3-((*S*)-1-hydroxyoctyl)dihydrofuran-2(3*H*)-one (**7g**, protected VB-S5)
Synthesized via the general protocol of the Noyori Hydrogenation to afford compound **7g** (52% yield) as a colorless oil, from compound **5g**.
TLC R_f = 0.45 (40% EtOAc in hexanes, $KMnO_4$); **Alpha D** $[\alpha]_D^{21}: -25.9°$ (*c* 1.0mg/ml, $CHCl_3$); **^{1}H NMR (500 MHz, $CDCl_3$)** δ 7.39 – 7.28 (m, 5H), 4.57 – 4.48 (m, 2H), 4.39 (t, *J* = 8.7 Hz, 1H), 4.17 – 4.12 (m, 1H), 4.10 (dd, *J* = 8.9, 6.7 Hz, 1H), 3.55 – 3.47 (m, 2H), 2.98 – 2.88 (m, 1H), 2.60 (dd, *J* = 7.2, 2.8 Hz, 1H), 2.05 (d, *J* = 5.7 Hz, 1H), 1.56 – 1.43 (m, 3H), 1.32 – 1.23 (m, 11H), 0.89 (t, *J* = 6.7 Hz, 3H); **^{13}C NMR (126 MHz, $CDCl_3$)** δ 178.62, 137.60, 128.55, 127.97, 127.71, 73.38, 70.72, 70.39, 69.83, 48.03, 35.89, 34.77, 31.80, 29.39, 29.22, 25.90, 22.65, 14.11; **FTIR** (Neat, cm^{-1}): *v* 3463, 2956, 2927, 2856, 1760; **HRMS** m/z calculated for $C_{20}H_{30}O_4$ $[M+H]^+$:335.22169, found $[M+H]^+$:335.2211. (-1.76ppm)

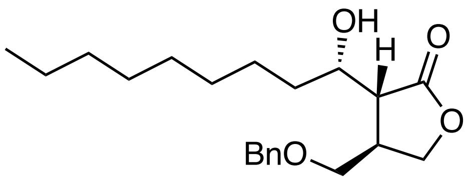

(3*R*,4*R*)-4-((benzyloxy)methyl)-3-((*S*)-1-hydroxynonyl)dihydrofuran-2(3*H*)-one (**7h**, protected VB-S6)
Synthesized via the general protocol of the Noyori Hydrogenation to afford compound **7h** (66% yield) as a colorless oil, from compound **5h**.
TLC R_f = 0.49 (40% EtOAc in hexanes, $KMnO_4$); **Alpha D** $[\alpha]_D^{21}: -21.2°$ (*c* 1.0mg/ml, $CHCl_3$); **^{1}H NMR (500 MHz, $CDCl_3$)** δ 7.42 – 7.27 (m, 5H), 4.61 – 4.48 (m, 2H), 4.42 (t, *J* = 9.0 Hz, 1H), 4.19 – 4.15 (m, 1H), 4.12 (dd, *J* = 8.9, 6.8 Hz, 1H), 3.59 – 3.47 (m, 2H), 2.95 (dtd, *J* = 8.5, 6.1, 1.7 Hz, 1H), 2.61 (dd, *J* = 7.3, 3.2 Hz, 1H), 2.11 (d, *J* = 5.4 Hz, 1H), 1.63 – 1.44 (m, 1H), 1.39 – 1.23 (m, 12H), 0.89 (d, *J* = 6.8 Hz, 3H); **^{13}C NMR (126 MHz, $CDCl_3$)** δ 178.63, 137.61, 128.54, 127.96, 127.70, 73.37, 70.72, 70.40, 69.84, 48.04, 35.88, 34.78, 31.87, 29.52, 29.44, 29.26, 25.91, 22.68,

14.13; **FTIR** (Neat, cm^{-1}): *v* 3463, 2954, 2925, 2856, 1764; **HRMS** m/z calculated for $C_{21}H_{32}O_4$ $[M+H]^+$:349.23734, found $[M+H]^+$:349.23644. (-2.57ppm)

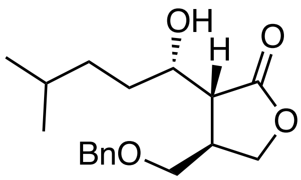

(3*R*,4*R*)-4-((benzyloxy)methyl)-3-((*S*)-1-hydroxy-4-methylpentyl)dihydrofuran-2(3*H*)-one (**7i**, protected VB-E)
Synthesized via the general protocol of the Noyori Hydrogenation to afford compound **7i** (73% yield) as a colorless oil, from compound **5i**.
TLC R_f = 0.42 (40% EtOAc in hexanes, $KMnO_4$); **Alpha D** $[\alpha]_D^{21}: -15.7°$ (*c* 2.7mg/ml, $CHCl_3$); **^{1}H NMR (500 MHz, $CDCl_3$)** δ 7.39 – 7.28 (m, 5H), 4.58 – 4.48 (m, 2H), 4.40 (t, *J* = 8.6 Hz, 1H), 4.16 – 4.07 (m, 2H), 3.51 (hept, *J* = 9.2 Hz, 2H), 2.98 – 2.89 (m, 1H), 2.61 (dd, *J* = 7.3, 3.1 Hz, 1H), 2.06 (d, *J* = 5.3 Hz, 1H), 1.60 – 1.48 (m, 3H), 1.41 – 1.32 (m, 1H), 1.29 – 1.12 (m, 2H), 0.93 – 0.83 (m, 6H); **^{13}C NMR (126 MHz, $CDCl_3$)** δ 178.60, 137.59, 128.56, 127.98, 127.71, 73.38, 71.06, 70.38, 69.82, 48.00, 35.89, 34.99, 32.63, 27.93, 22.59, 22.50; **FTIR** (Neat, cm^{-1}): *v* 3458, 2954, 2869, 1764; **HRMS** m/z calculated for $C_{18}H_{26}O_4$ $[M]^+$:306.1831, found $[M]^+$:306.1824. (-3.4ppm)

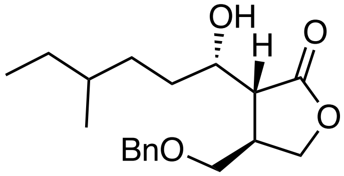

(3*R*,4*R*)-4-((benzyloxy)methyl)-3-((1*S*)-1-hydroxy-4-methylhexyl)dihydrofuran-2(3*H*)-one (**7j**, protected VB-B)
Synthesized via the general protocol of the Noyori Hydrogenation to afford compound **7j** (73% yield) as a colorless oil, from compound **5j**.
TLC R_f = 0.49 (40% EtOAc in hexanes, $KMnO_4$); **Alpha D** $[\alpha]_D^{21}: -25.4°$ (*c* 1.0mg/ml, $CHCl_3$); **^{1}H NMR (500 MHz, $CDCl_3$)** δ 7.40 – 7.28 (m, 5H), 4.57 – 4.49 (m, 2H), 4.40 (t, *J* = 8.7 Hz, 1H), 4.14 – 4.07 (m, 2H), 3.55 – 3.46 (m, 2H), 2.98 – 2.88 (m, 1H), 2.60 (dt, *J* = 7.3, 3.1 Hz, 1H), 2.09 – 2.03 (m, 1H), 1.56 – 1.43 (m, 2H), 1.38 – 1.04 (m, 5H), 0.91 – 0.80 (m, 6H); **^{13}C NMR (126 MHz, $CDCl_3$)** δ 178.75, 137.61, 128.54, 127.96, 127.71, 73.36, 71.20, 70.43, 69.90, 48.00, 35.87, 34.25, 32.67, 32.44, 29.40, 19.18, 11.39; **FTIR** (Neat, cm^{-1}): *v* 3459, 2958, 2915, 2871, 1764 ; **HRMS** m/z calculated for $C_{19}H_{28}O_4$ $[M+H]^+$:321.20604, found $[M+H]^+$:321.20542. (-1.93ppm)

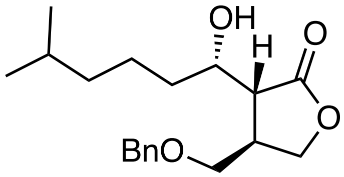

(3*R*,4*R*)-4-((benzyloxy)methyl)-3-((*S*)-1-hydroxy-5-methylhexyl)dihydrofuran-2(3*H*)-one (**7k**, protected VB-A)
Synthesized via the general protocol of the Noyori Hydrogenation to afford compound **7k** (52% yield) as a colorless oil, from compound **5k**.
TLC R_f = 0.50 (40% EtOAc in hexanes, $KMnO_4$); **Alpha D** $[\alpha]_D^{21}: -20.6°$ (*c* 1.0mg/ml, $CHCl_3$); **^{1}H NMR (500 MHz, $CDCl_3$)** δ 7.39 – 7.28 (m, 5H), 4.57 – 4.49 (m, 2H), 4.39 (t, *J* = 8.6 Hz, 1H), 4.17 – 4.07 (m, 2H), 3.55 – 3.46 (m, 2H), 2.93 (h, *J* = 6.9 Hz, 1H), 2.60 (dd, *J* = 7.3, 3.1 Hz, 1H), 2.09 (s, 1H), 1.56 – 1.43 (m, 4H), 1.37 – 1.26 (m, 2H), 1.20 – 1.14 (m, 2H), 0.91 – 0.83 (m, 6H); **^{13}C NMR (126 MHz, $CDCl_3$)** δ 178.59, 137.59, 128.55, 127.97, 127.71, 73.38, 70.73, 70.38, 69.82, 48.07, 38.71, 35.92, 34.99, 27.90, 23.69, 22.58, 22.55; **FTIR** (Neat, cm^{-1}): *v* 3459, 2958, 2915, 2871, 1764; **HRMS** m/z calculated for $C_{19}H_{28}O_4$ $[M+H]^+$:321.20604, found $[M+H]^+$:321.20512. (-2.86ppm)

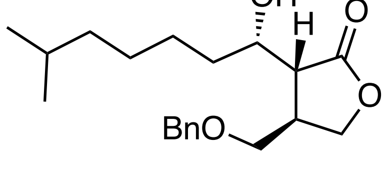

(3*R*,4*R*)-4-((benzyloxy)methyl)-3-((*S*)-1-hydroxy-6-methylheptyl)dihydrofuran-2(3*H*)-one (**7l**, protected VB-S7))
Synthesized via the general protocol of the Noyori Hydrogenation to afford compound **7l** (52% yield) as a colorless oil, from compound **5l**.

TLC R_f = 0.46 (40% EtOAc in hexanes, $KMnO_4$); **Alpha D** $[\alpha]_D^{21}: -26.6°$ (*c* 1.9mg/ml, $CHCl_3$); **^{1}H NMR (500 MHz, $CDCl_3$)** δ 7.41 – 7.30 (m, 5H), 4.60 – 4.52 (m, 2H), 4.42 (t, *J* = 8.7 Hz, 1H), 4.19 – 4.14 (m, 1H), 4.12 (dd, *J* = 8.9, 6.8 Hz, 1H), 3.58 – 3.50 (m, 2H), 2.99 – 2.91 (m, 1H), 2.62 (dd, *J* = 7.3, 3.2 Hz, 1H), 2.07 (s, 1H), 1.60 – 1.45 (m, 4H), 1.36 – 1.26 (m, 4H), 1.23 – 1.14 (m, 2H), 0.89 (dd, *J* = 6.6, 2.5 Hz, 7H); **^{13}C NMR (126 MHz, $CDCl_3$)** δ 178.61, 137.60, 128.55, 127.98, 127.71, 73.38, 70.72, 70.37, 69.82, 48.05, 38.89, 35.91, 34.80, 27.94, 27.20, 26.16, 22.62, 22.58; **FTIR** (Neat, cm^{-1}): *v* 3436, 2552, 2929, 2867, 1764; **HRMS** m/z calculated for $C_{20}H_{30}O_4$ $[M]^+$:334.2144, found $[M]^+$: 334.2124. (-6.1ppm)

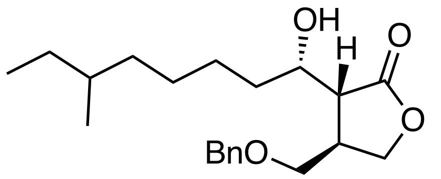

(3*R*,4*R*)-4-((benzyloxy)methyl)-3-((1*S*)-1-hydroxy-6-methyloctyl)dihydrofuran-2(3*H*)-one (**7m**, protected VB-S8)

Synthesized via the general protocol of the Noyori Hydrogenation to afford compound **7m** (66% yield) as a colorless oil, from compound **5m**.

TLC R_f = 0.50 (40% EtOAc in hexanes, $KMnO_4$); **Alpha D** $[\alpha]_D^{21}: -29.1°$ (*c* 2.9mg/ml, $CHCl_3$); **^{1}H NMR (500 MHz, $CDCl_3$)** δ 7.39 – 7.27 (m, 5H), 4.57 – 4.49 (m, 2H), 4.40 (d, *J* = 8.7 Hz, 1H), 4.16 – 4.07 (m, 2H), 3.56 – 3.47 (m, 2H), 2.93 (d, *J* = 6.6 Hz, 1H), 2.59 (dd, *J* = 7.3, 3.2 Hz, 1H), 2.07 (s, 1H), 1.57 – 1.42 (m, 3H), 1.38 – 1.21 (m, 8H), 1.19 – 1.04 (m, 3H), 0.88 – 0.82 (m, 7H); **^{13}C NMR (126 MHz, $CDCl_3$)** δ 178.62, 137.60, 128.55, 127.97, 127.71, 73.38, 70.73, 70.37, 69.83, 48.05, 36.52, 35.90, 34.81, 34.35, 29.46, 26.92, 26.90, 26.26, 19.20, 11.42; **FTIR** (Neat, cm^{-1}): *v* 3453, 2954, 2935, 2859, 1764; **HRMS** m/z calculated for $C_{21}H_{32}O_4$ $[M+H]^+$: 348.2301, found $[M]^+$: 348.2370. (-6.6ppm)

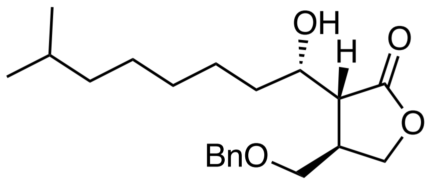

(3*R*,4*R*)-4-((benzyloxy)methyl)-3-((*S*)-1-hydroxy-7-methyloctyl)dihydrofuran-2(3*H*)-one (**7n**, protected VB-S9)

Synthesized via the general protocol of the Noyori Hydrogenation to afford compound **7n** (64% yield) as a colorless oil, from compound **5n**.

TLC R_f = 0.49 (40% EtOAc in hexanes, $KMnO_4$); **Alpha D** $[\alpha]_D^{21}: -26.3°$ (*c* 3.7mg/ml, $CHCl_3$); **^{1}H NMR (500 MHz, $CDCl_3$)** δ 7.38 – 7.27 (m, 5H), 4.57 – 4.49 (m, 2H), 4.40 (t, *J* = 8.7 Hz, 1H), 4.17 – 4.12 (m, 1H), 4.10 (dd, *J* = 8.9, 6.8 Hz, 1H), 3.55 – 3.47 (m, 2H), 2.92 (d, *J* = 6.6 Hz, 1H), 2.59 (dd, *J* = 7.3, 3.2 Hz, 1H), 2.06 (s, 1H), 1.55 – 1.45 (m, 4H), 1.29 – 1.24 (m, 5H), 1.19 – 1.12 (m, 2H), 0.87 (d, *J* = 6.6 Hz, 7H); **^{13}C NMR (126 MHz, $CDCl_3$)** δ 178.61, 137.60, 128.55, 127.97, 127.70, 73.38, 70.73, 70.39, 69.83, 48.03, 38.94, 35.90, 34.78, 29.69, 27.96, 27.31, 25.94, 22.66; **FTIR** (Neat, cm^{-1}): *v* 3452, 2952, 2927, 2858, 1764; **HRMS** m/z calculated for $C_{21}H_{32}O_4$ $[M+H]^+$: 348.2301, found $[M+H]^+$: 348.2280. (-4.6ppm)

General Protocol for Benzyl Deprotection

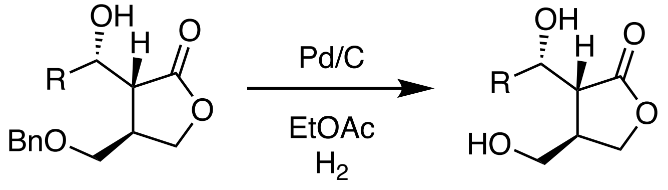

7a - n **8a - n**

Activated Pd/C (10 wt %) (0.20 equiv.) was added to a flame dried round bottom flask under a nitrogen atmosphere at room temperature. Benzyl protected VB-type molecule in EtOAc (0.01M)

was added to the round bottom flask. The reaction was sparged with N_2 for ten minutes followed by sparging with H_2 for twenty minutes. The reaction then stirred overnight under H_2. The reaction was filtered using a silica plug to remove palladium. The resulting filtrate was concentrated and purified via flash chromatography to afford pure VB type molecule.

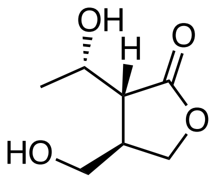

(3*R*,4*R*)-3-((*S*)-1-hydroxyethyl)-4-(hydroxymethyl)dihydrofuran-2(3*H*)-one (**8a**, VB-S1)

Synthesized via the general deprotection protocol to afford compound **8a** (71% yield) as a colorless oil, from compound **7a**.

TLC R_f = 0.18 (80% EtOAc in hexanes, KMnO₄); **Alpha D** $[\alpha]_D^{21}: -19.9°$ (*c* 1.0mg/ml, $CHCl_3$); **^{1}H NMR (500 MHz, CDCl₃)** δ 4.43 (t, *J* = 8.8 Hz, 1H), 4.28 (qd, *J* = 6.4, 4.5 Hz, 1H), 4.10 (dd, *J* = 9.1, 7.1 Hz, 1H), 3.80 – 3.70 (m, 2H), 2.84 (dtd, *J* = 13.2, 7.9, 5.8 Hz, 1H), 2.55 (dd, *J* = fzg7.7, 4.6 Hz, 2H), 1.39 (d, *J* = 6.3 Hz, 3H); **^{13}C NMR (126 MHz, CDCl₃)**; δ 178.09, 69.19, 66.95, 63.30, 49.55, 39.02, 21.18; **FTIR** (Neat, cm^{-1}): *v* 3407, 2973, 2923, 1749 ; **HRMS** m/z calculated for $C_7H_{12}O_4$ $[M]^+$: 160.0736, found $[M]^+$: 160.0739. (2.2ppm)

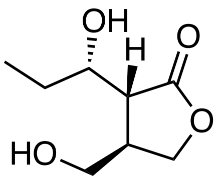

(3*R*,4*R*)-3-((*S*)-1-hydroxybutyl)-4-(hydroxymethyl)dihydrofuran-2(3*H*)-one (**8b**, VB-S2)

Synthesized via the general deprotection protocol to afford compound **8b** (52% yield) as a colorless oil, from compound **7b**.

TLC R_f = 0.30 (60% EtOAc in hexanes, KMnO₄); **Alpha D** $[\alpha]_D^{21}: -15.5°$ (*c* 2.2mg/ml, $CHCl_3$); **^{1}H NMR (500 MHz, CDCl₃)** δ 4.42 (t, *J* = 8.8 Hz, 1H), 4.10 (dd, *J* = 9.0, 6.9 Hz, 1H), 4.05 (p, *J* = 4.3 Hz, 1H), 3.74 (hept, *J* = 5.7 Hz, 2H), 2.86 (d, *J* = 6.8 Hz, 1H), 2.59 (dd, *J* = 7.3, 3.9 Hz, 1H), 2.18 (s, 1H), 1.92 (s, 1H), 1.73 – 1.58 (m, 2H), 1.02 (t, *J* = 7.4 Hz, 3H); **^{13}C NMR (126 MHz, CDCl₃)** δ 178.37, 72.36, 69.36, 63.38, 47.76, 38.17, 27.88, 10.21; **FTIR** (Neat, cm^{-1}): *v* 3415, 2966, 2923, 1745; **HRMS** m/z calculated for $C_8H_{14}O_4$ $[M]^+$:170.0892, found $[M]^+$:174.0879. (-7.3ppm)

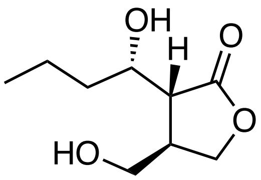

(3*R*,4*R*)-4-((benzyloxy)methyl)-3-((*S*)-1-hydroxy-5-methylhexyl)dihydrofuran-2(3*H*)-one (**8c**, VB-S3)

Synthesized via the general deprotection protocol to afford compound **8c** (76% yield) as a colorless oil, from compound **7c**.

TLC R_f = 0.34 (60% EtOAc in hexanes, KMnO₄); **Alpha D** $[\alpha]_D^{21}: -21.6°$ (*c* 1.0mg/ml, $CHCl_3$); **^{1}H NMR (500 MHz, CDCl₃)** δ 7.39 – 7.28 (m, 5H), 4.57 – 4.49 (m, 2H), 4.39 (t, *J* = 8.6 Hz, 1H), 4.17 – 4.07 (m, 2H), 3.55 – 3.46 (m, 2H), 2.93 (h, *J* = 6.9 Hz, 1H), 2.60 (dd, *J* = 7.3, 3.1 Hz, 1H), 2.09 (s, 1H), 1.56 – 1.43 (m, 4H), 1.37 – 1.26 (m, 2H), 1.20 – 1.14 (m, 2H), 0.91 – 0.83 (m, 6H); **^{13}C NMR (126 MHz, CDCl₃)** δ 178.59, 137.59, 128.55, 127.97, 127.71, 73.38, 70.73, 70.38, 69.82, 48.07, 38.71, 35.92, 34.99, 27.90, 23.69, 22.58, 22.55; **FTIR** (Neat, cm^{-1}): *v* 3415, 2960, 2933, 2875, 1751; **HRMS** m/z calculated for $C_{19}H_{28}O_4$ $[M+H]^+$:321.20604, found $[M+H]^+$:321.20512. (-2.86ppm)

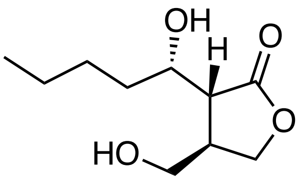

(3*R*,4*R*)-4-(hydroxymethyl)-3-((*S*)-1-hydroxypentyl)dihydrofuran-2(3*H*)-one (**8d**, VB-S4)

Synthesized via the general deprotection protocol to afford compound **8d** (57% yield) as a colorless oil, from compound **7d**.

TLC R_f = 0.46 (30% EtOAc in hexanes, KMnO₄); **Alpha D** $[\alpha]_D^{21}: -25.2°$ (*c* 4.6mg/ml, $CHCl_3$);**^{1}H NMR (500 MHz, CDCl₃)** δ 4.42 (t, *J* = 8.7 Hz, 1H), 4.15 – 4.07 (m, 2H), 3.73 (qd, *J* = 10.5, 5.9 Hz, 2H), 2.91 – 2.81 (m, 1H), 2.57 (dd, *J* = 7.3, 3.8 Hz, 1H), 1.68 – 1.21 (m, 4H), 0.97 – 0.82 (m, 3H); **^{13}C NMR (126 MHz, CDCl₃)** δ 178.47,

70.86, 69.43, 63.39, 48.12, 38.15, 34.55, 27.98, 22.51, 14.02; **FTIR** (Neat, cm^{-1}): *v* 3409, 2956, 2931, 2871, 1749; **HRMS** m/z calculated for $C_{10}H_{18}O_4$ $[M]^+$:202.1205, found $[M]^+$:202.1185. (-2.0ppm)

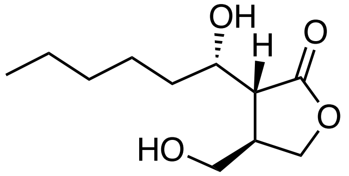

(3*R*,4*R*)-3-((*S*)-1-hydroxyhexyl)-4-(hydroxymethyl)dihydrofuran-2(3*H*)-one (**8e**, VB-C)
Synthesized via the general deprotection protocol to afford compound **8e** (91% yield) as a colorless oil, from compound **7e**.
TLC R_f = 0.23 (50% EtOAc in hexanes, $KMnO_4$); **Alpha D** $[\alpha]_D^{21}: -32.4°$ (*c* 2.8mg/ml, $CHCl_3$); **^{1}H NMR (500 MHz, $CDCl_3$)** δ 4.42 (t, *J* = 8.7 Hz, 1H), 4.16 – 4.07 (m, 2H), 3.73 (qdd, *J* = 10.5, 5.9, 1.6 Hz, 2H), 2.86 (d, *J* = 6.6 Hz, 1H), 2.57 (dd, *J* = 7.4, 3.8 Hz, 1H), 2.04 (s, 2H), 1.63 – 1.47 (m, 3H), 1.37 – 1.29 (m, 5H), 0.93 – 0.86 (m, 3H); **^{13}C NMR (126 MHz, $CDCl_3$)** δ 178.44, 70.86, 69.41, 63.37, 48.09, 38.13, 34.80, 31.59, 25.48, 22.57, 13.99; **FTIR** (Neat, cm^{-1}): *v* 3411, 2929, 2859, 1749; **HRMS** m/z calculated for $C_{11}H_{20}O_4$ $[M]^+$:216.1362, found $[M]^+$:16.1342. (-9.0ppm)

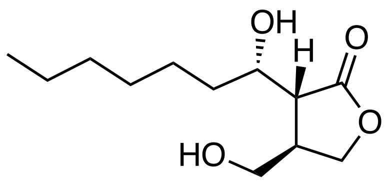

(3*R*,4*R*)-3-((*S*)-1-hydroxyheptyl)-4-(hydroxymethyl)dihydrofuran-2(3*H*)-one (**8f**, VB-D)
Synthesized via the general deprotection protocol to afford compound **8f** (95% yield) as a colorless oil, from compound **7f**.
TLC R_f = 0.24 (50% EtOAc in hexanes, $KMnO_4$); **Alpha D** $[\alpha]_D^{21}: -22.7°$ (*c* 1.7mg/ml, $CHCl_3$); **^{1}H NMR (500 MHz, $CDCl_3$)** δ 4.42 (t, *J* = 8.7 Hz, 1H), 4.15 – 4.07 (m, 2H), 3.74 (qd, *J* = 10.5, 5.9 Hz, 2H), 2.90 – 2.82 (m, 1H), 2.57 (dd, *J* = 7.3, 3.8 Hz, 1H), 2.16 (d, *J* = 48.1 Hz, 2H), 1.62 – 1.48 (m, 4H), 1.35 – 1.28 (m, 7H), 0.91 – 0.87 (m, 4H); **^{13}C NMR (126 MHz, $CDCl_3$)** δ 178.36, 70.88, 69.38, 63.40, 48.08, 38.14, 34.87, 31.76, 29.10, 25.79, 22.60, 14.07; **FTIR** (Neat, cm^{-1}): *v* 3415, 2954, 2923, 2856, 1749; **HRMS** m/z calculated for $C_{12}H_{22}O_4$ $[M]^+$:320.1518, found $[M]^+$:320.1979. (-2.7ppm)

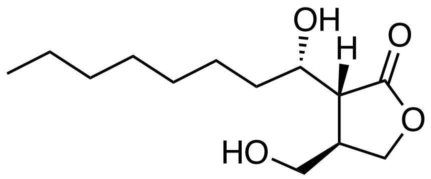

(3*R*,4*R*)-4-(hydroxymethyl)-3-((*S*)-1-hydroxyoctyl)dihydrofuran-2(3*H*)-one (**8g**, VB-5)
Synthesized via the general deprotection protocol to afford compound **8g** (94% yield) as a colorless oil, from compound **7g**.
TLC R_f = 0.25 (50% EtOAc in hexanes, $KMnO_4$); **Alpha D** $[\alpha]_D^{21}: -11.0°$ (*c* 1.0mg/ml, $CHCl_3$); **^{1}H NMR (500 MHz, $CDCl_3$)** δ 4.42 (t, *J* = 8.7 Hz, 1H), 4.15 – 4.07 (m, 2H), 3.73 (qd, *J* = 10.6, 5.8 Hz, 2H), 2.90 – 2.81 (m, 1H), 2.56 (dd, *J* = 7.2, 3.8 Hz, 1H), 2.30 (s, 1H), 2.04 (s, 1H), 1.61 – 1.48 (m, 3H), 1.39 – 1.23 (m, 10H), 0.88 (d, *J* = 6.7 Hz, 3H); **^{13}C NMR (126 MHz, $CDCl_3$)** δ 178.53, 70.86, 69.46, 63.38, 48.13, 38.14, 34.87, 31.79, 29.40, 29.23, 25.85, 22.64, 14.10; **FTIR** (Neat, cm^{-1}): *v* 3405, 2925, 2858, 1764; **HRMS** m/z calculated for $C_{13}H_{24}O_4$ $[M+H]^+$:245.17474, found $[M+H]^+$:245.17378. (-3.91ppm)

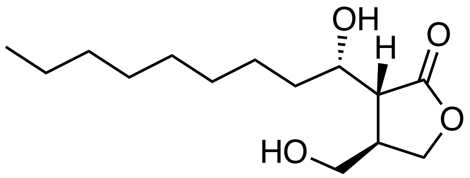

(3*R*,4*R*)-4-(hydroxymethyl)-3-((*S*)-1-hydroxynonyl)dihydrofuran-2(3*H*)-one (**8h**, VB-6)
Synthesized via the general deprotection protocol to afford compound **8h** (70% yield) as a colorless oil, from compound **7h**.
TLC R_f = 0.32 (50% EtOAc in hexanes, $KMnO_4$); **Alpha D** $[\alpha]_D^{21}: -27.0°$ (*c* 1.0mg/ml, $CHCl_3$); **^{1}H NMR (500 MHz, $CDCl_3$)** δ 4.42 (t, *J* = 8.7 Hz, 1H), 4.16 – 4.06 (m, 2H), 3.76 – 3.65 (m, 2H), 3.03 (s, 1H), 2.89 – 2.80 (m, 2H), 2.55 (dd, *J* = 7.0, 3.7 Hz, 1H), 1.64 – 1.44 (m, 3H), 1.38 – 1.22 (m, 12H), 0.89 (t, *J* = 7.0 Hz, 3H); **^{13}C NMR (126 MHz,

$CDCl_3$) δ 179.19, 70.80, 69.85, 63.35, 48.32, 38.06, 34.92, 31.87, 29.56, 29.48, 29.27, 25.90, 22.67, 14.11; **FTIR** (Neat, cm^{-1}): *v* 3417, 2956, 2929, 2858; **HRMS** m/z calculated for $C_{14}H_{25}O_4$ $[M+H]^+$:259.19039, found $[M+H]^+$:259.18958. (-3.12ppm)

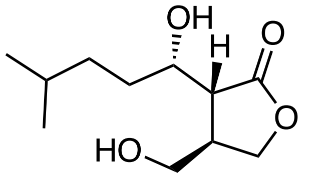

(3*R*,4*R*)-3-((*S*)-1-hydroxy-4-methylpentyl)-4-(hydroxymethyl)dihydrofuran-2(3*H*)-one (**8i**, VB-E)

Synthesized via the general deprotection protocol to afford compound **8i** (77% yield) as a colorless oil, from compound **7i**.

TLC R_f = 0.19 (60% EtOAc in hexanes, $KMnO_4$); **Alpha D** $[\alpha]_D^{21}: -24.8°$ (*c* 1.0mg/ml, $CHCl_3$); **^{1}H NMR (500 MHz, $CDCl_3$)** δ 4.42 (t, *J* = 8.7 Hz, 1H), 4.13 – 4.06 (m, 2H), 3.74 (qd, *J* = 10.5, 5.9 Hz, 2H), 2.91 – 2.83 (m, 1H), 2.57 (dd, *J* = 7.3, 3.8 Hz, 1H), 1.68 – 1.52 (m, 4H), 1.44 – 1.35 (m, 1H), 1.25 – 1.17 (m, 2H), 0.91 (dd, *J* = 6.5, 1.7 Hz, 6H); **^{13}C NMR (126 MHz, $CDCl_3$)** δ 178.38, 71.20, 69.38, 63.40, 48.07, 38.16, 34.94, 32.71, 27.95, 22.65, 22.49; **FTIR** (Neat, cm^{-1}): *v* 3415, 2954, 2923, 2850, 1749; **HRMS** m/z calculated for $C_{11}H_{20}O_4$ $[M]^+$:216.1362, found $[M]^+$:216.1344 (-8.2ppm)

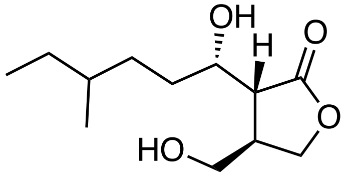

(3*R*,4*R*)-3-((1*S*)-1-hydroxy-4-methylhexyl)-4-(hydroxymethyl)dihydrofuran-2(3*H*)-one (**8j**, VB-B)

Synthesized via the general deprotection protocol to afford compound **8j** (86% yield) as a colorless oil, from compound **7j**.

TLC R_f = 0.25 (60% EtOAc in hexanes, $KMnO_4$); **Alpha D** $[\alpha]_D^{21}: -9.2°$ (*c* 1.0mg/ml, $CHCl_3$); **^{1}H NMR (500 MHz, $CDCl_3$)** δ 4.42 (t, *J* = 8.7 Hz, 1H), 4.13 – 4.05 (m, 2H), 3.78 – 3.67 (m, 2H), 2.86 (h, *J* = 6.9 Hz, 1H), 2.57 (dd, *J* = 7.4, 4.0 Hz, 1H), 2.34 (s, 2H), 1.69 – 1.46 (m, 3H), 1.41 – 1.28 (m, 3H), 1.19 – 1.10 (m, 1H), 0.87 (t, *J* = 7.2 Hz, 6H); **^{13}C NMR (126 MHz, $CDCl_3$)** δ 178.67, 71.34, 71.19, 69.53, 63.38, 48.22, 38.09, 34.28, 32.68, 29.46, 19.23, 11.34; **FTIR** (Neat, cm^{-1}): *v* 3432, 2960, 2931, 2875, 1749; **HRMS** m/z calculated for $C_{12}H_{22}O_4$ $[M+H]^+$:231.15909, found $[M+H]^+$:231.15829. (-3.46ppm)

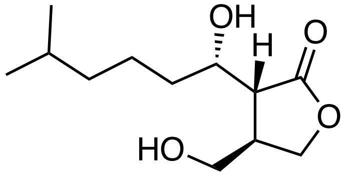

(3*R*,4*R*)-3-((*S*)-1-hydroxy-5-methylhexyl)-4-(hydroxymethyl)dihydrofuran-2(3*H*)-one (**8k**, VB-A)

Synthesized via the general deprotection protocol to afford compound **8k** (91% yield) as a colorless oil, from compound **7k**.

TLC R_f = 0.25 (60% EtOAc in hexanes, $KMnO_4$); **Alpha D** $[\alpha]_D^{21}: -16.3°$ (*c* 2.1mg/ml, $CHCl_3$); **^{1}H NMR (500 MHz, $CDCl_3$)** δ 4.44 (t, *J* = 8.7 Hz, 1H), 4.17 – 4.10 (m, 2H), 3.75 (qd, *J* = 10.6, 5.8 Hz, 2H), 2.93 – 2.82 (m, 1H), 2.59 (dd, *J* = 7.2, 3.8 Hz, 1H), 2.34 (s, 2H), 1.63 – 1.48 (m, 4H), 1.38 – 1.22 (m, 4H), 0.90 (d, *J* = 6.5 Hz, 6H); **^{13}C NMR (126 MHz, $CDCl_3$)** δ 178.58, 70.84, 69.48, 38.70, 38.13, 35.09, 27.90, 23.60, 22.58, 22.53; **FTIR** (Neat, cm^{-1}): *v* 3411, 2954, 2931, 2869, 1747; **HRMS** m/z calculated for $C_{12}H_{22}O_4$ $[M]^+$:230.1518, found $[M]^+$:230.1501. (-7.4ppm)

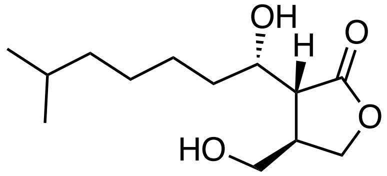

(3*R*,4*R*)-3-((*S*)-1-hydroxy-6-methylheptyl)-4-(hydroxymethyl)dihydrofuran-2(3*H*)-one (**8l**, VB-S7)

Synthesized via the general deprotection protocol to afford compound **8l** (72% yield) as a colorless oil, from compound **7l**.

TLC R_f = 0.25 (50% EtOAc in hexanes, $KMnO_4$); **Alpha D** $[\alpha]_D^{21}: -29.1°$ (*c* 3.6mg/ml, $CHCl_3$); **^{1}H NMR (500 MHz, $CDCl_3$)** δ 4.44 (t, *J* = 8.7 Hz, 1H), 4.12 (ddd, *J* = 10.1, 5.9, 2.9 Hz, 2H), 3.74 (qd, *J* = 10.7, 5.9 Hz, 2H), 2.87 (d, *J* = 6.6 Hz, 1H), 2.58 (dd, *J* = 7.1, 3.8 Hz, 1H), 1.67 – 1.46 (m, 4H), 1.39 – 1.30 (m, 3H), 1.23 – 1.16 (m, 2H), 0.89 (d, *J* = 6.6 Hz, 7H); **^{13}C NMR (126 MHz, $CDCl_3$)** δ 178.79, 70.82, 69.60, 63.34, 48.22, 38.89, 38.13, 34.90, 27.92, 27.21, 26.11, 22.62, 22.59; **FTIR** (Neat, cm^{-1}): *v* 3386,

2954, 2929, 2869, 1747; **HRMS** m/z calculated for $C_{13}H_{24}O_4$ $[M]^+$:244.171675, found $[M]^+$:244.1660. (-5.9ppm)

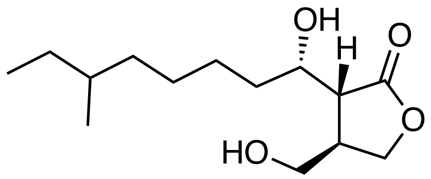

(3*R*,4*R*)-3-((1*S*)-1-hydroxy-6-methyloctyl)-4-(hydroxymethyl)dihydrofuran-2(3*H*)-one (**8m**, VB-8)
Synthesized via the general deprotection protocol to afford compound **8m** (76% yield) as a colorless oil, from compound **7m**.
TLC R_f = 0.21 (60% EtOAc in hexanes, $KMnO_4$); **Alpha D** $[\alpha]_D^{21}: -11.5°$ (*c* 3.1mg/ml, $CHCl_3$); **^{1}H NMR (500 MHz, $CDCl_3$)** δ 4.42 (t, *J* = 8.7 Hz, 1H), 4.15 – 4.06 (m, 2H), 3.72 (qd, *J* = 10.6, 5.8 Hz, 2H), 2.93 – 2.79 (m, 1H), 2.56 (dd, *J* = 7.2, 3.7 Hz, 3H), 1.66 – 1.53 (m, 2H), 1.47 (ttt, *J* = 13.5, 7.1, 3.0 Hz, 1H), 1.39 – 1.22 (m, 7H), 1.18 – 1.05 (m, 2H), 0.89 – 0.81 (m, 6H); **^{13}C NMR (126 MHz, $CDCl_3$)** δ 178.80, 70.85, 70.83, 69.60, 63.35, 48.22, 38.14, 36.54, 34.92, 34.36, 29.48, 29.46, 26.96, 26.23, 26.21, 19.21, 11.43; **FTIR** (Neat, cm^{-1}): *v* 3415, 2958, 2929, 2858, 1745; **HRMS** m/z calculated for $C_{14}H_{26}O_4$ $[M]^+$:258.1831, found $[M+H]^+$: 258.1813. (-6.9ppm)

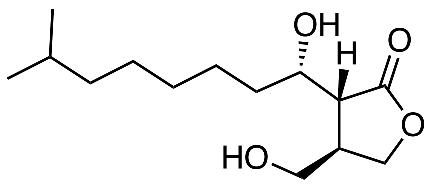

(3*R*,4*R*)-3-((*S*)-1-hydroxy-7-methyloctyl)-4-(hydroxymethyl)dihydrofuran-2(3*H*)-one (**8n,** VB-S9)
Synthesized via the general deprotection protocol to afford compound **8n** (90% yield) as a colorless oil, from compound **7n**.
TLC R_f = 0.22 (60% EtOAc in hexanes, $KMnO_4$); **Alpha D** $[\alpha]_D^{21}: -11.0°$ (*c* 2.0mg/ml, $CHCl_3$); **^{1}H NMR (500 MHz, $CDCl_3$)** δ 4.42 (t, *J* = 8.7 Hz, 1H), 4.16 – 4.08 (m, 2H), 3.74 (qd, *J* = 10.6, 5.9 Hz, 2H), 2.90 – 2.82 (m, 1H), 2.57 (dd, *J* = 7.3, 3.8 Hz, 1H), 2.08 (s, 1H), 1.85 (s, 1H), 1.65 – 1.47 (m, 4H), 1.39 – 1.26 (m, 5H), 1.21 – 1.12 (m, 2H), 0.87 (d, *J* = 6.5 Hz, 6H); **^{13}C NMR (126 MHz, $CDCl_3$)** δ 178.34, 70.89, 69.38, 63.41, 48.08, 38.92, 38.14, 34.88, 29.70, 27.95, 27.32, 25.87, 22.65; **FTIR** (Neat, cm^{-1}): *v* 3392, 2952, 2929, 2856, 1747; **HRMS** m/z calculated for $C_{14}H_{26}O_4$ $[M]^+$: 258.1831, found $[M]^+$: 258.1812. (-7.2ppm)

NMR Spectra

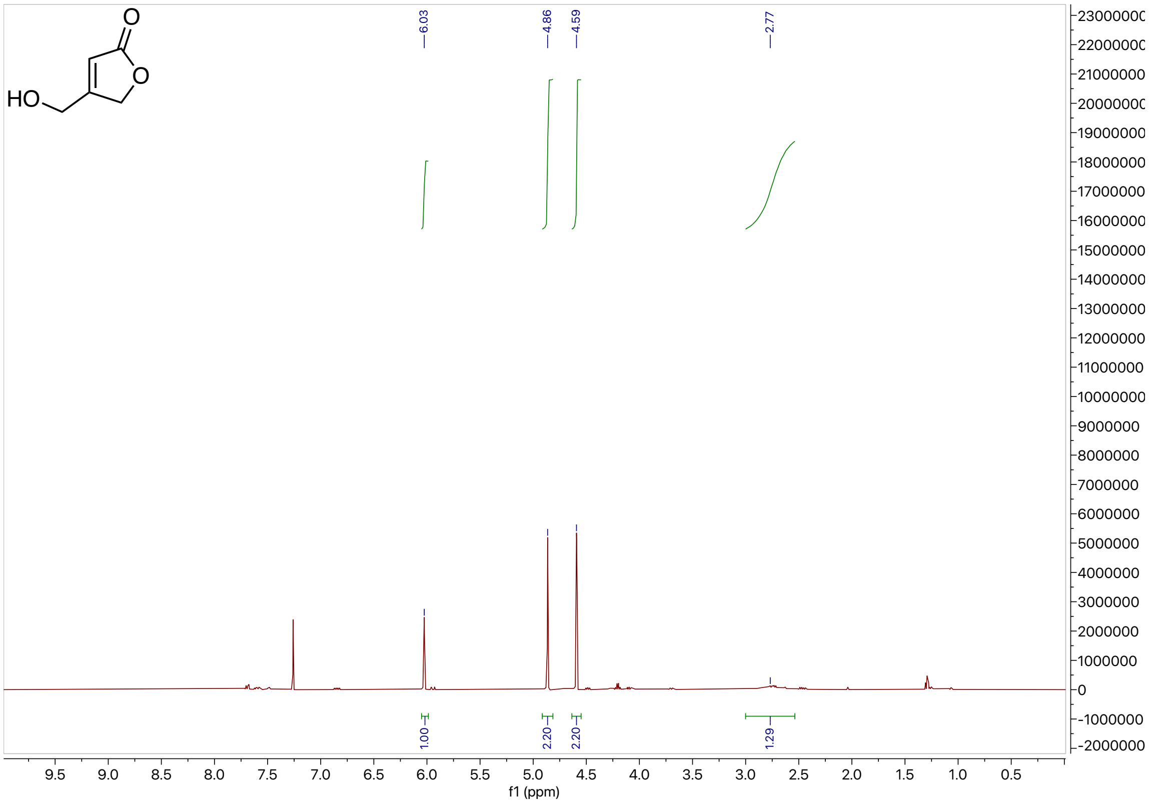

Figure S7. ^{1}H NMR Spectrum of Compound **2**

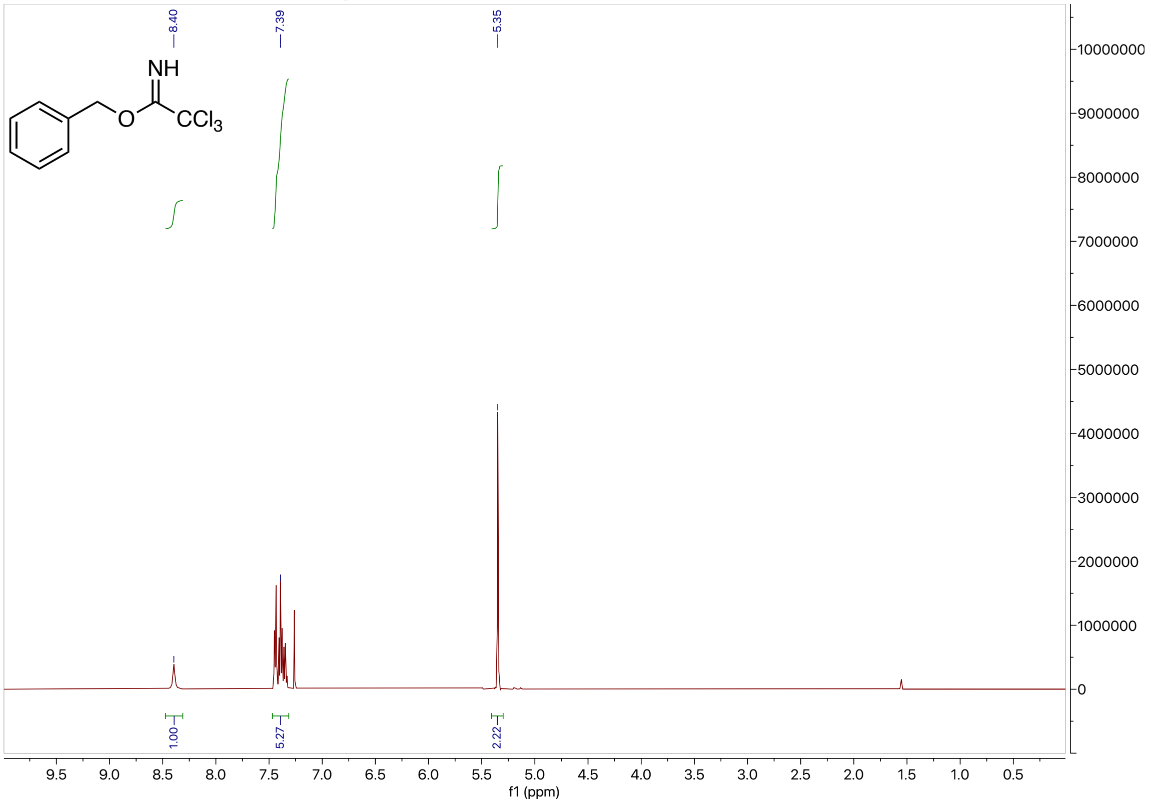

Figure S8. ^{1}H NMR Spectrum of Compound **3**

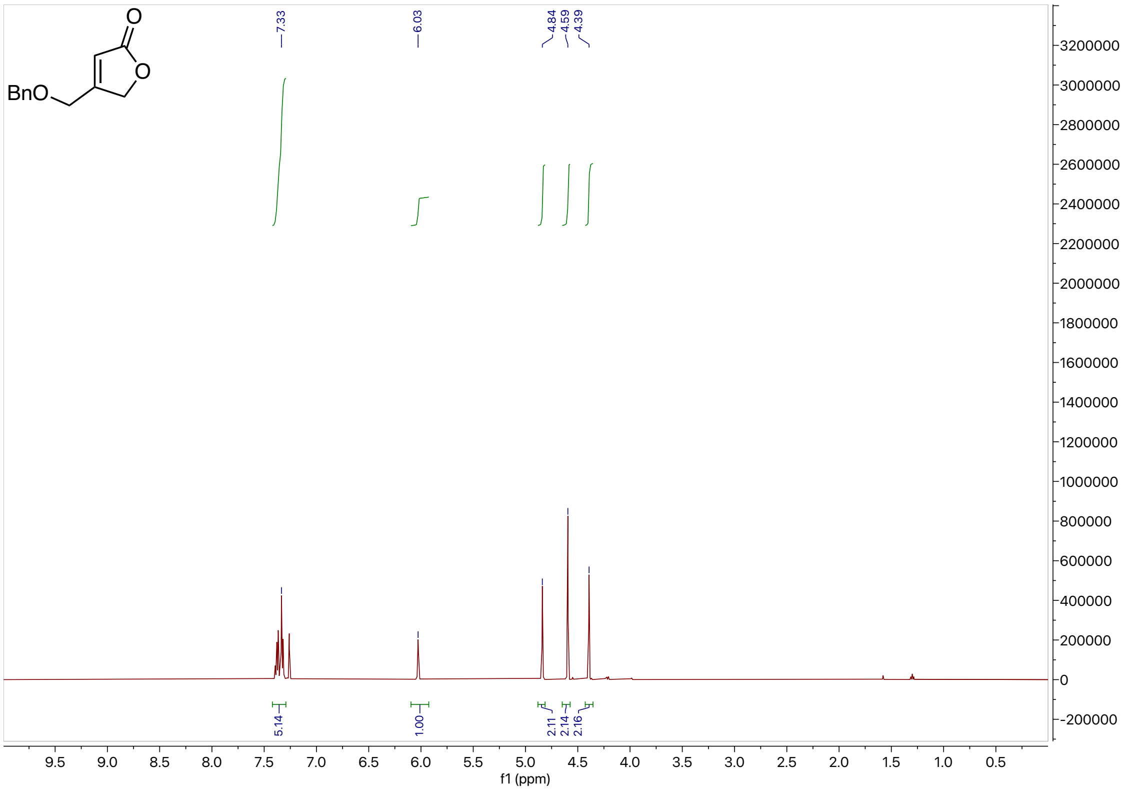

Figure S9. ^{1}H NMR Spectrum of Compound **4**

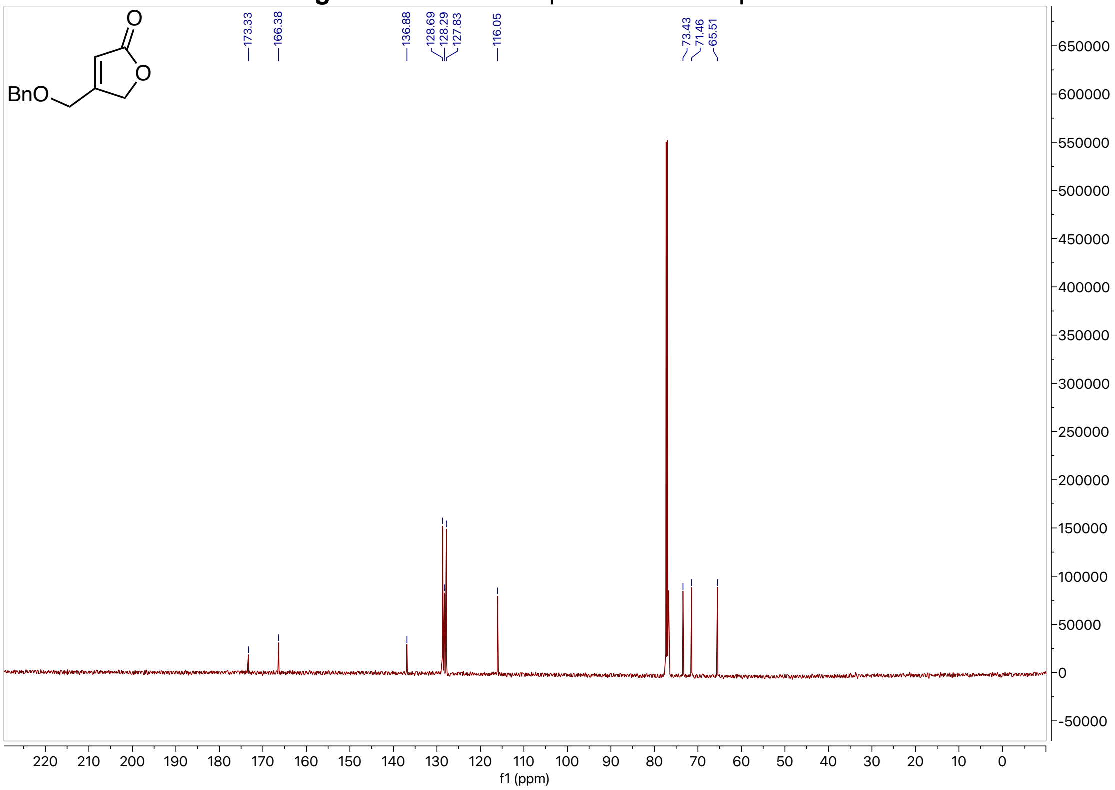

Figure S10. ^{13}C NMR Spectrum of Compound **4**

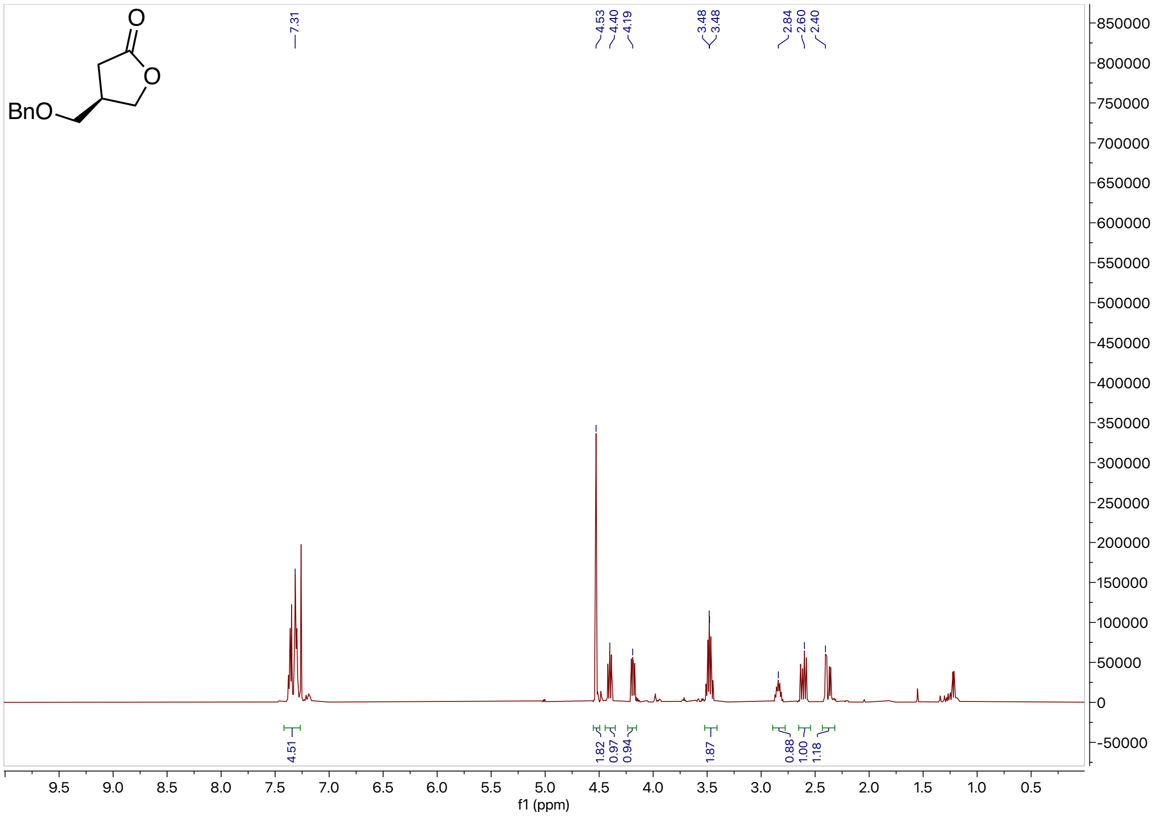

Figure S11. ^{1}H NMR Spectrum of Compound **1**

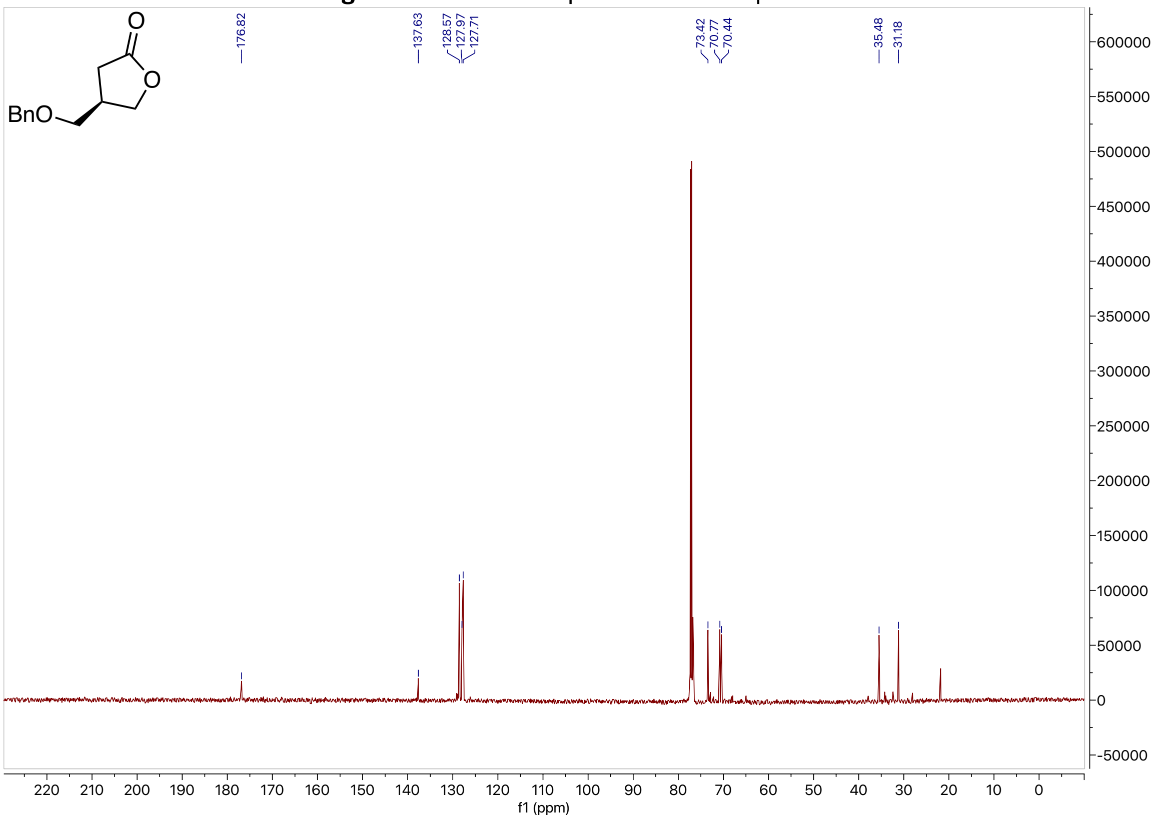

Figure S12. ^{13}C NMR Spectrum of Compound **1**

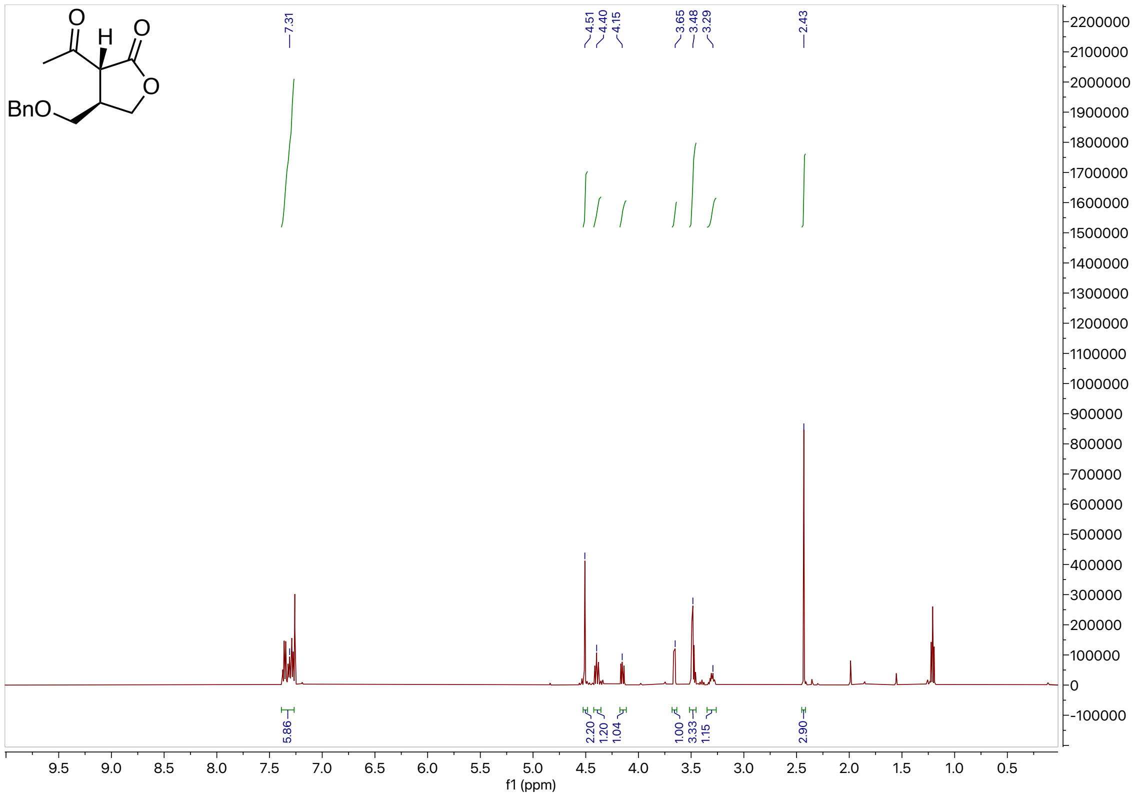

Figure S13. ^{1}H NMR Spectrum of Compound **5a**

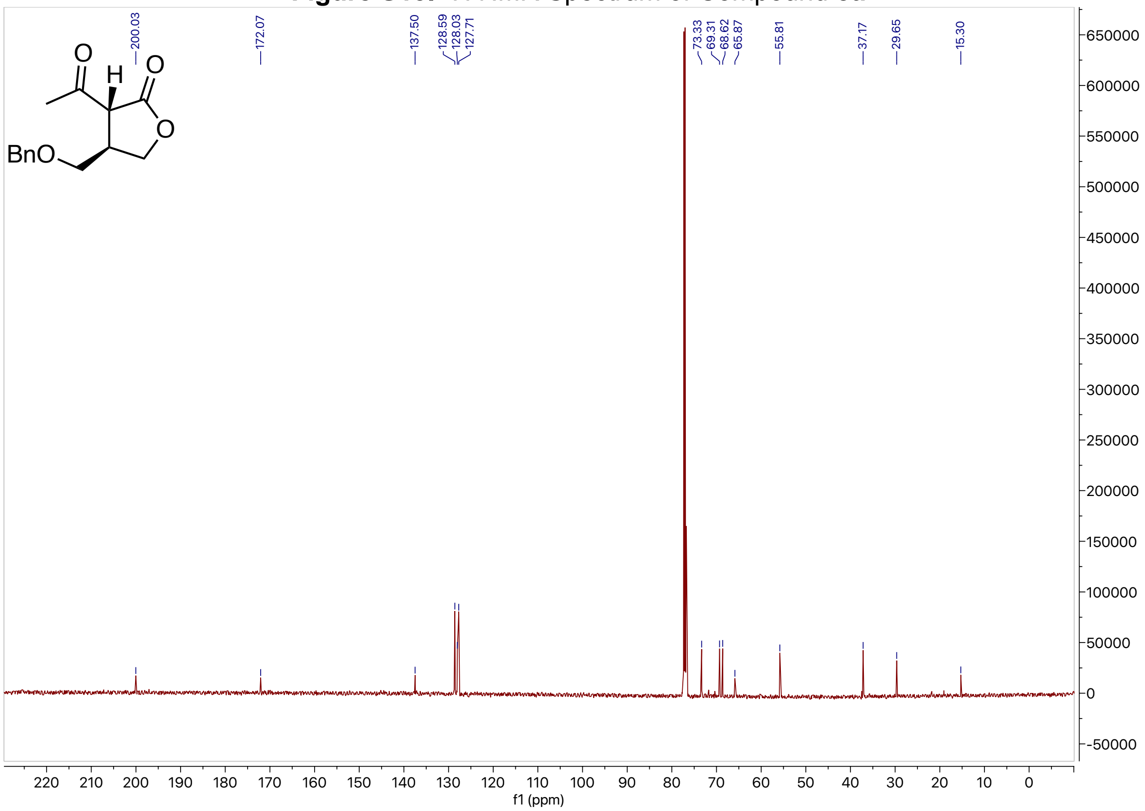

Figure S14. ^{13}C NMR Spectrum of Compound **5a**

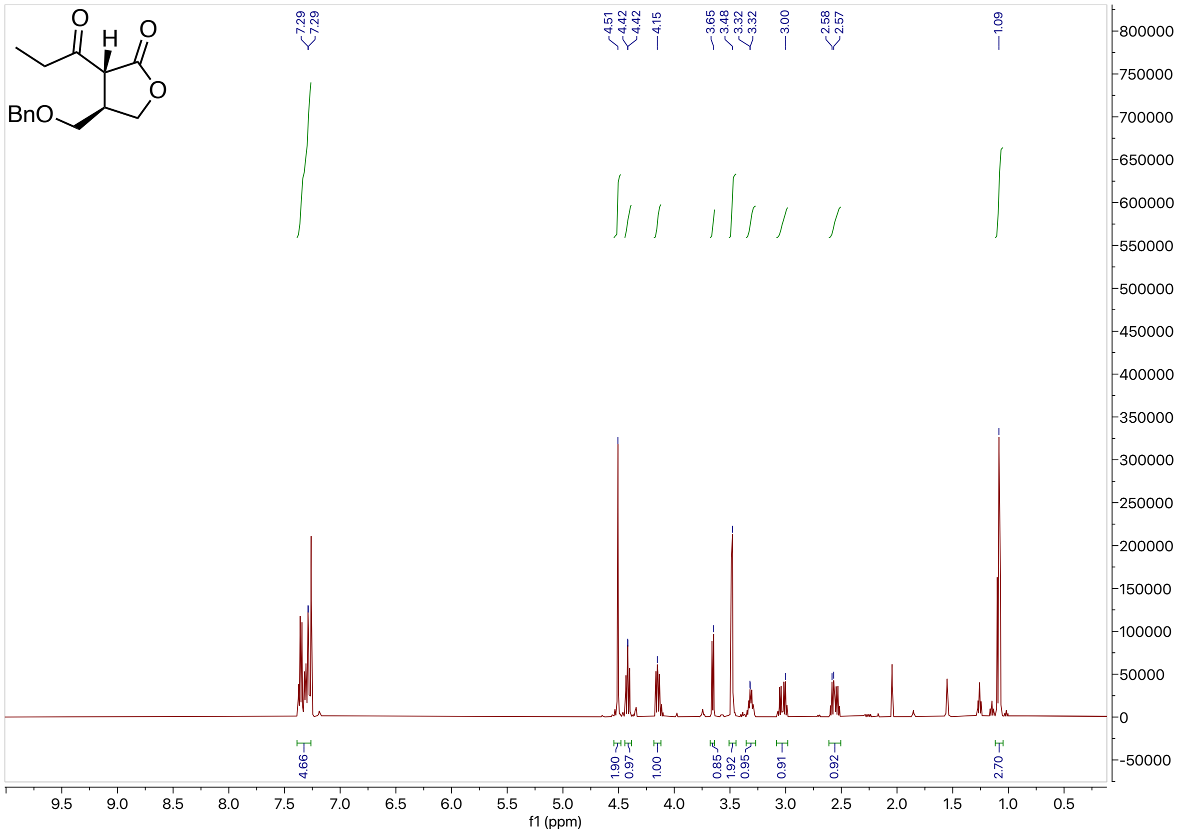

Figure S15. ^{1}H NMR Spectrum of Compound **5b**

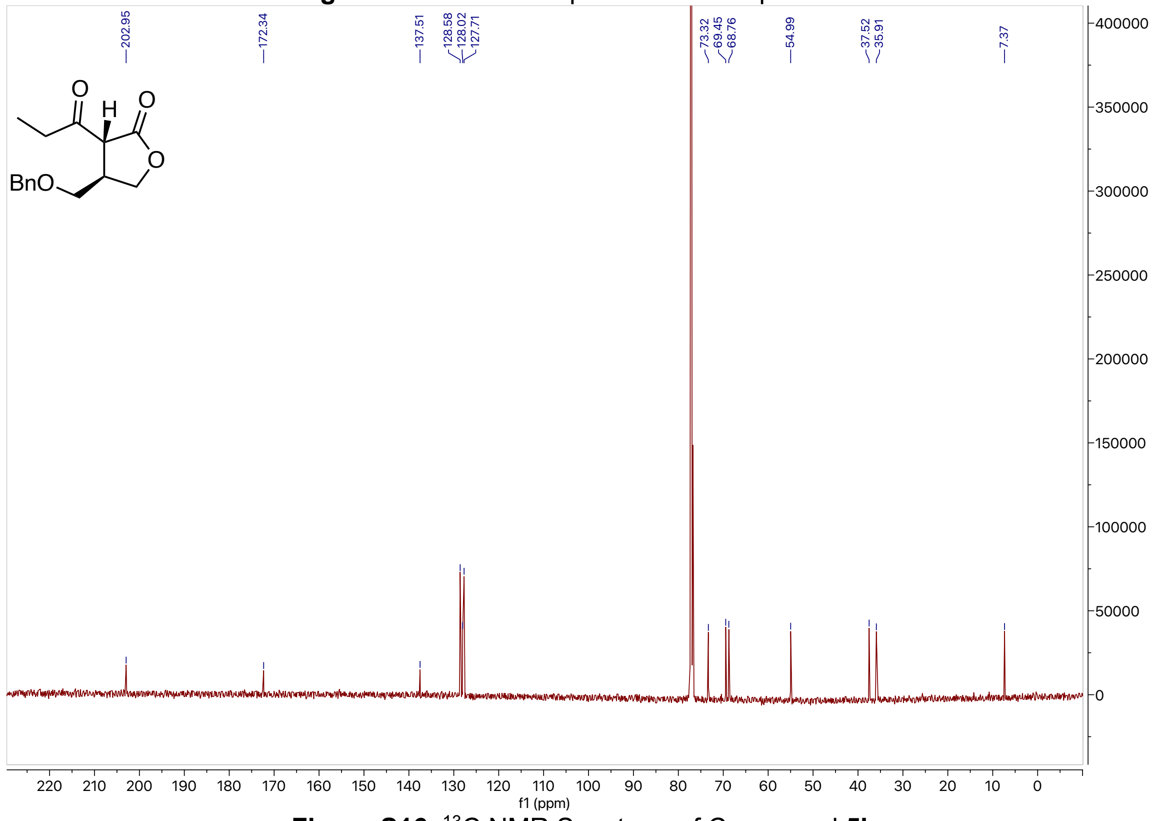

Figure S16. ^{13}C NMR Spectrum of Compound **5b**

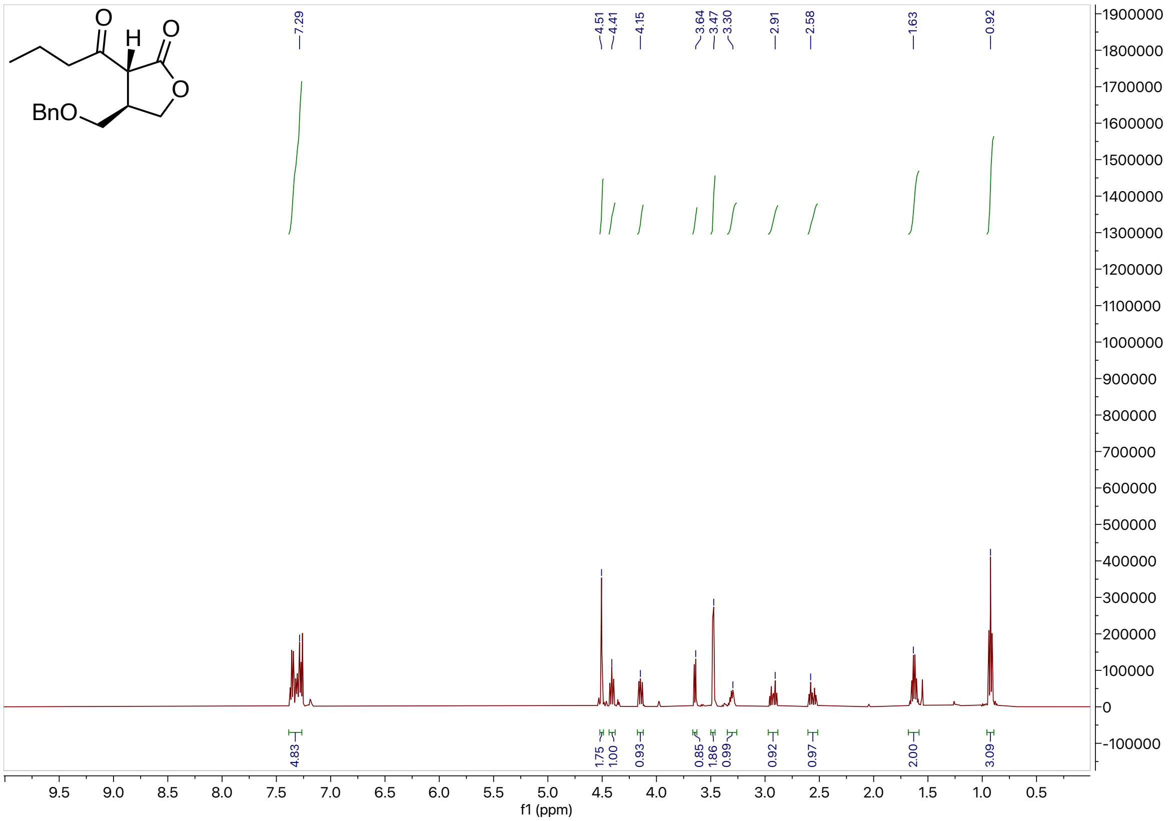

Figure S17. ^{1}H NMR Spectrum of Compound **5c**

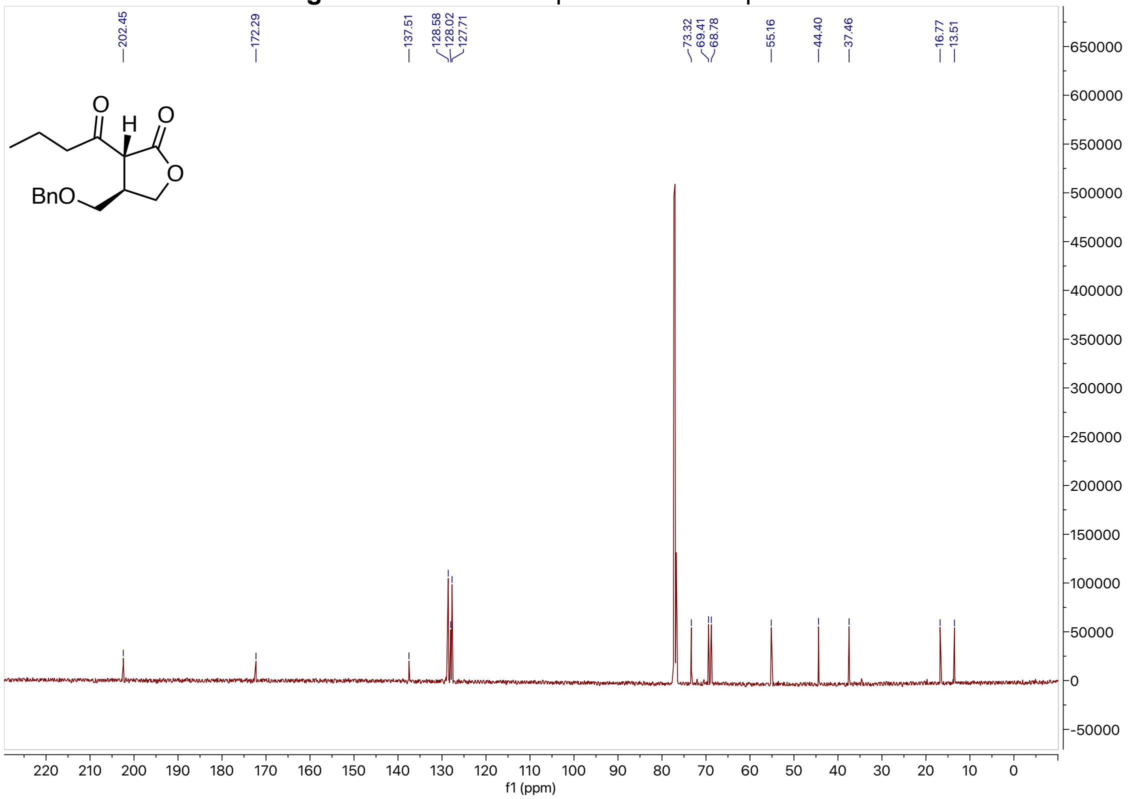

Figure 18. ^{13}C NMR Spectrum of Compound **5c**

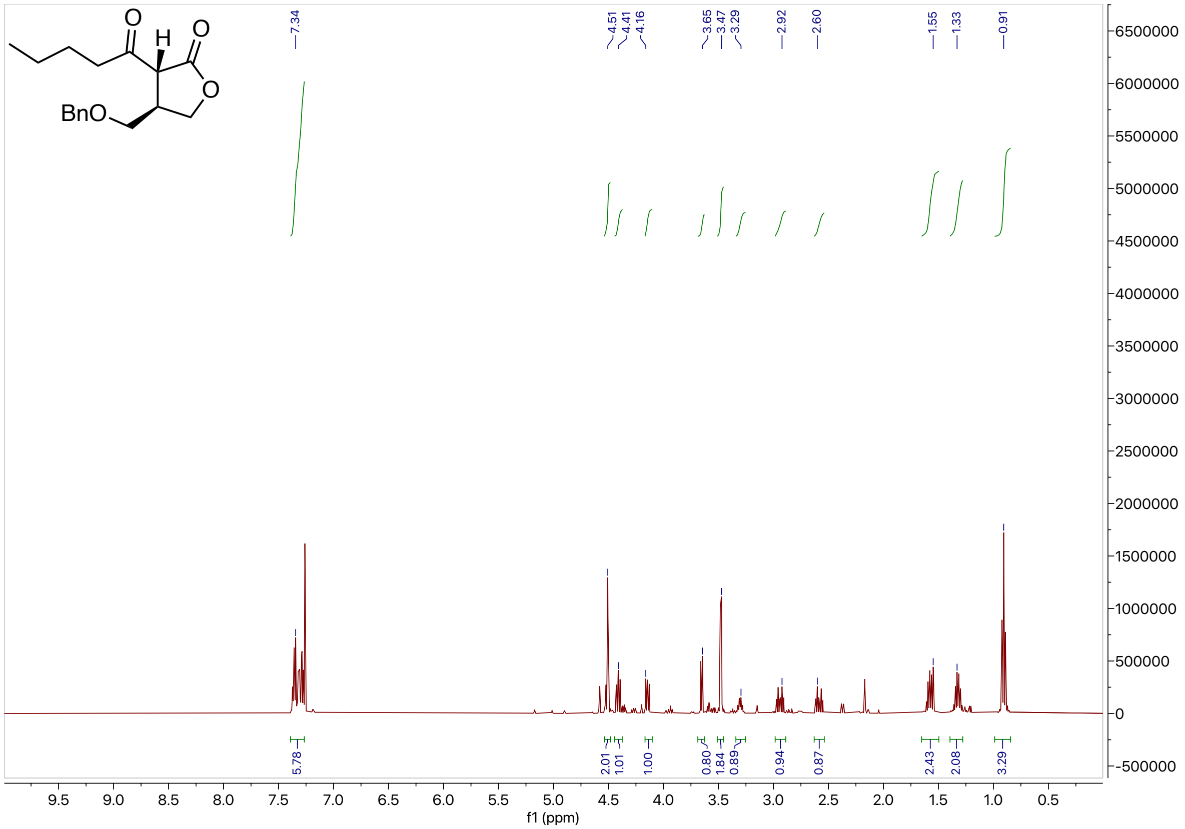

Figure S19. ^{1}H NMR Spectrum of Compound **5d**

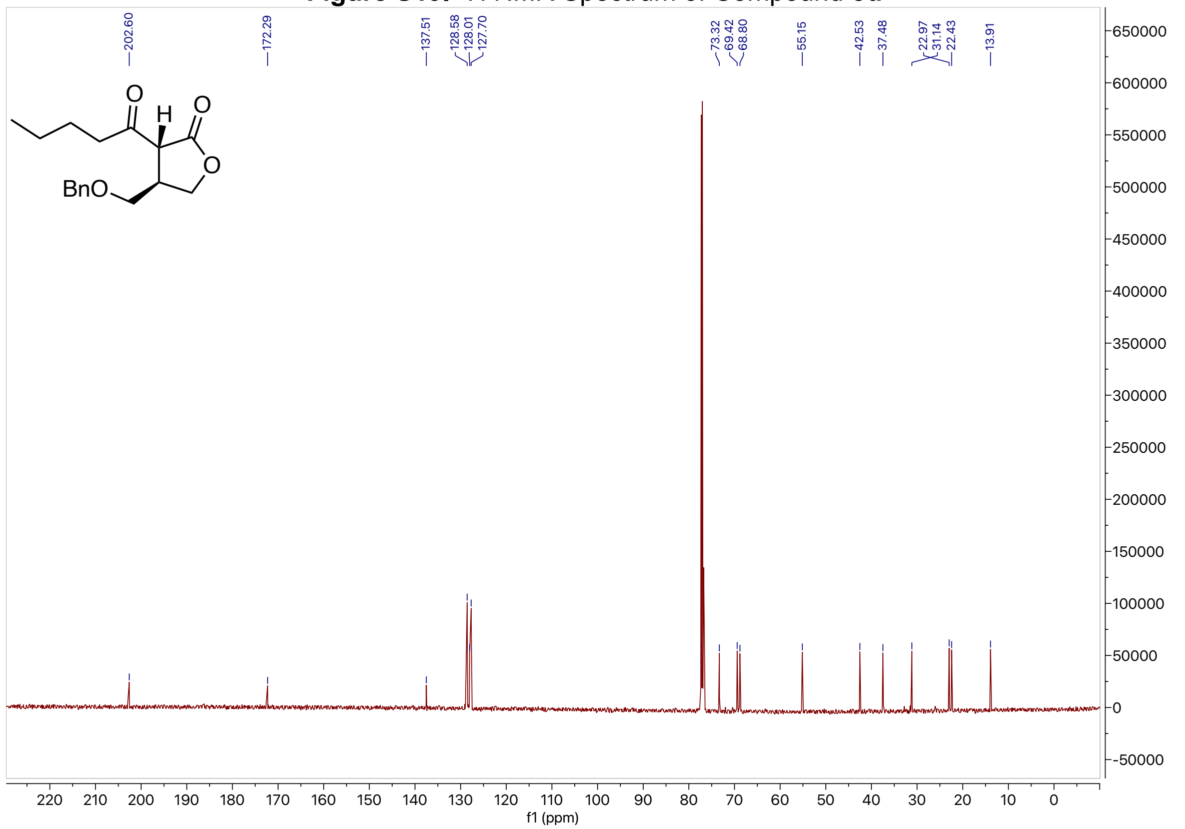

Figure S20. ^{13}C NMR Spectrum of Compound **5d**

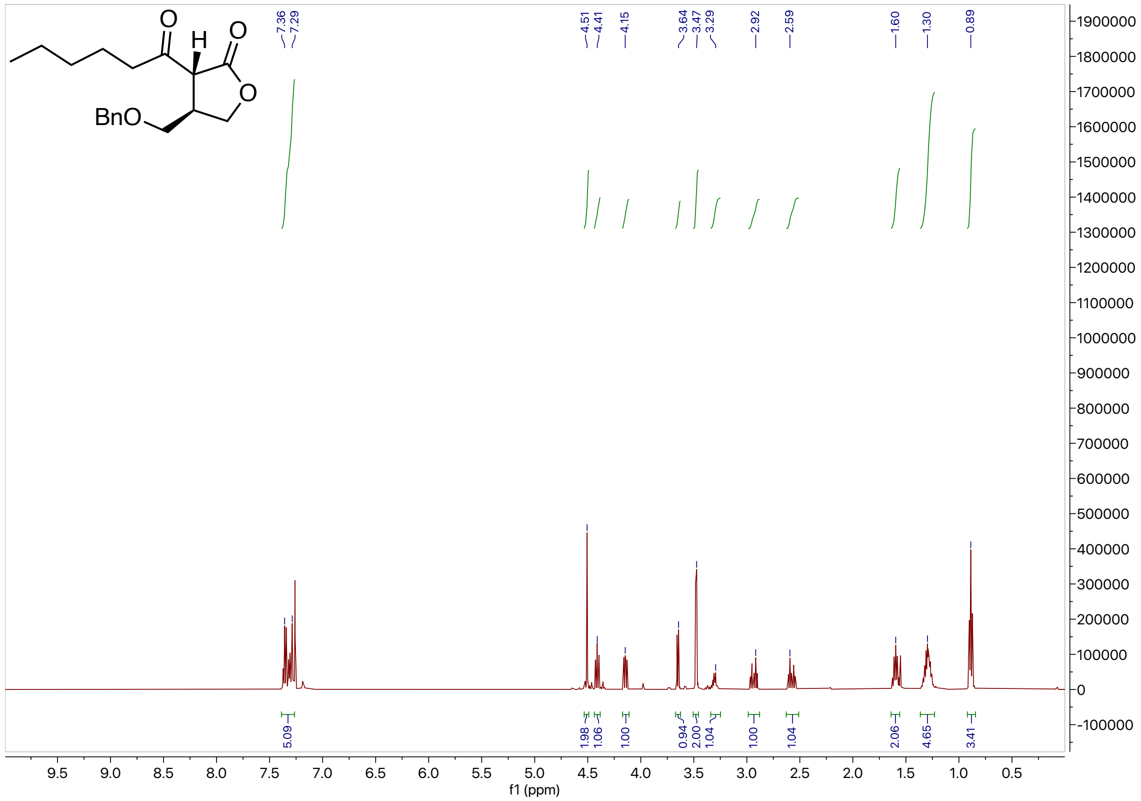

Figure S21. ^{1}H NMR Spectrum of Compound **5e**

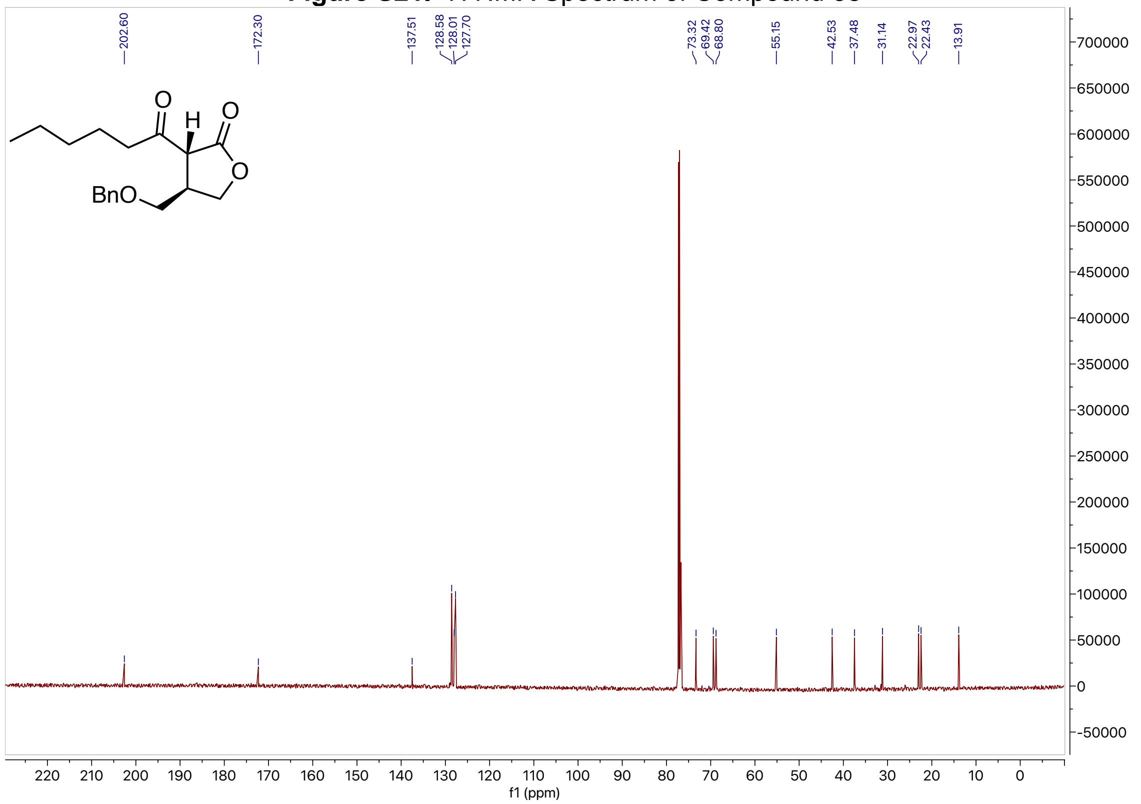

Figure S22. ^{13}C NMR Spectrum of Compound **5e**

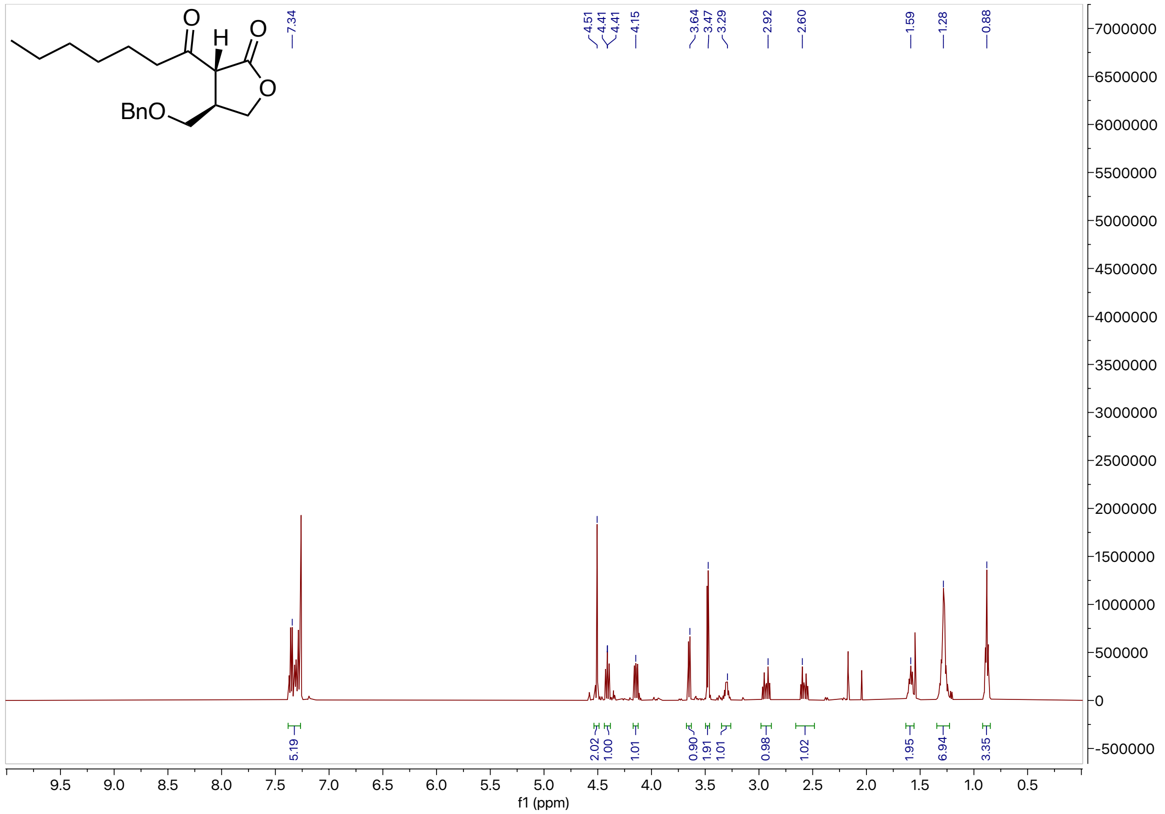

Figure S23. ^{1}H NMR Spectrum of Compound **5f**

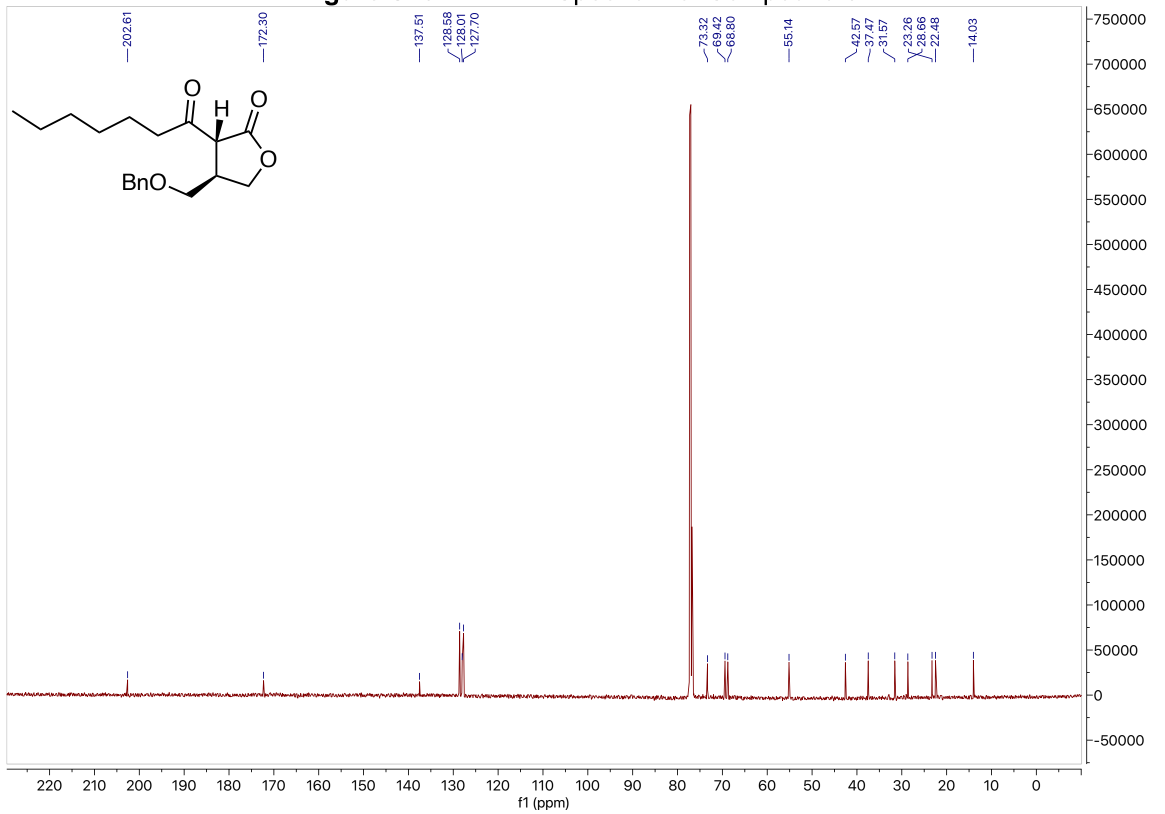

Figure S24. ^{13}C NMR Spectrum of Compound **5f**

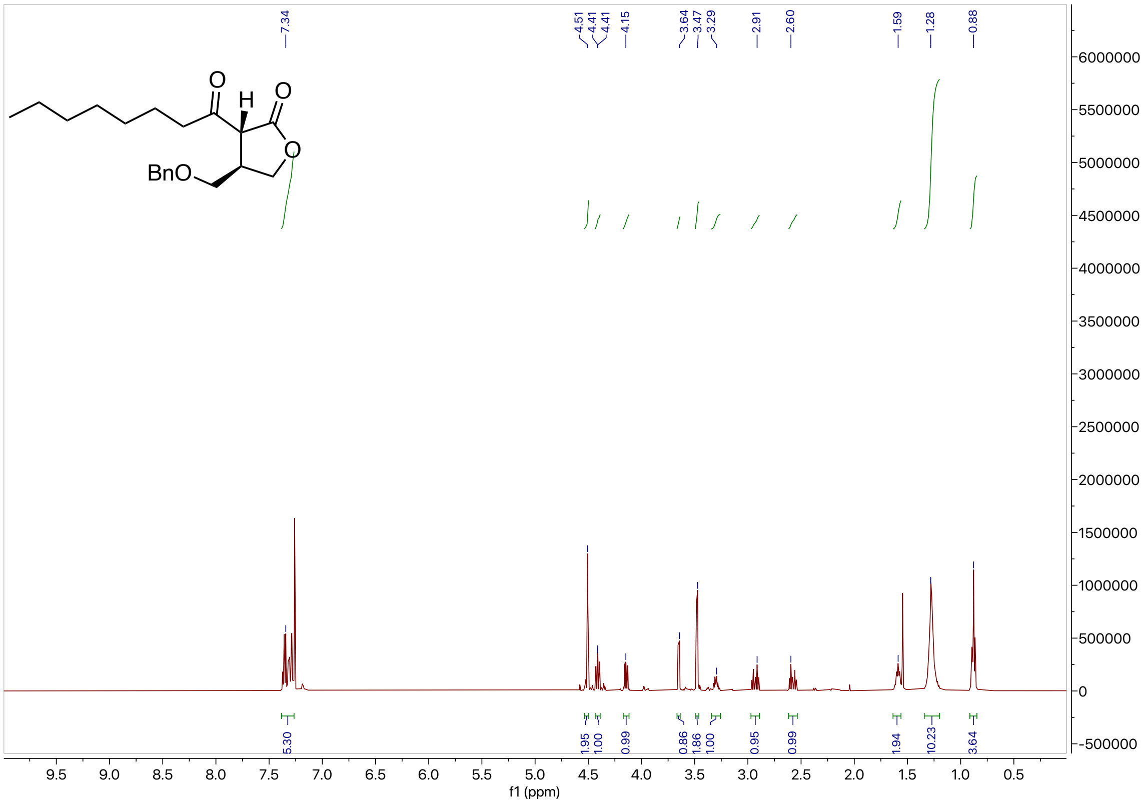

Figure S25. ^{1}H NMR Spectrum of Compound **5g**

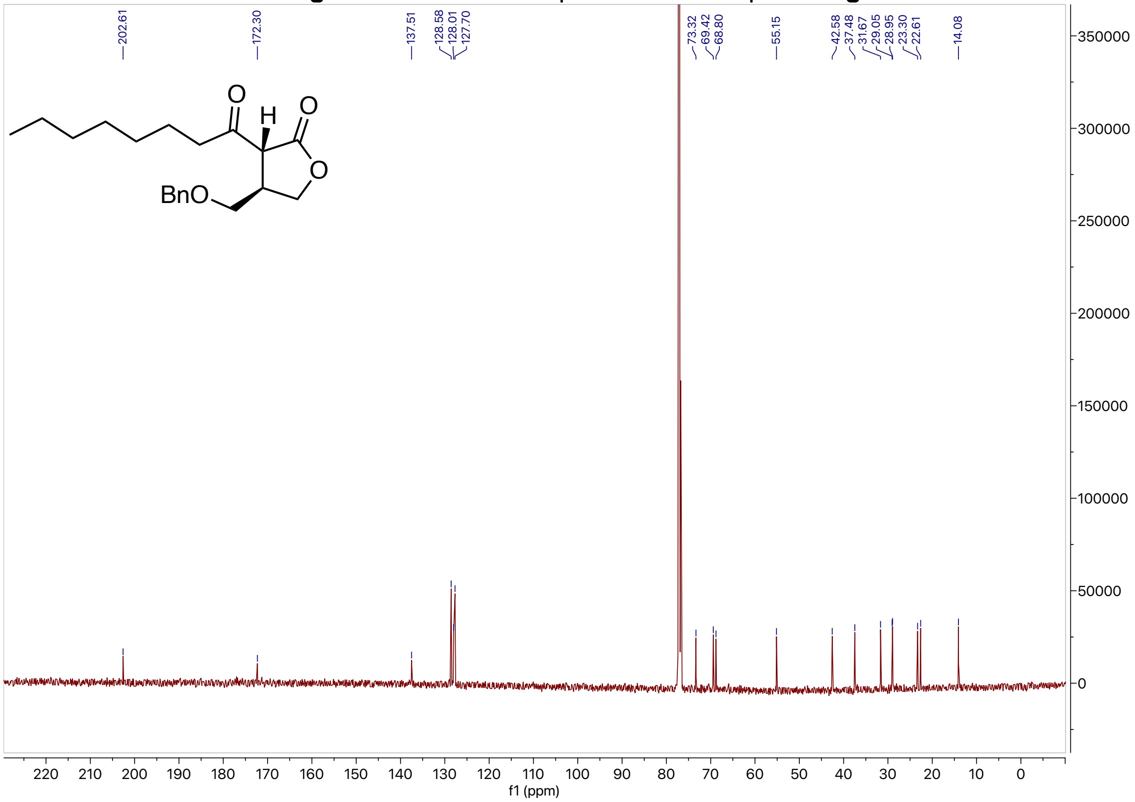

Figure S26. ^{13}C NMR Spectrum of Compound **5g**

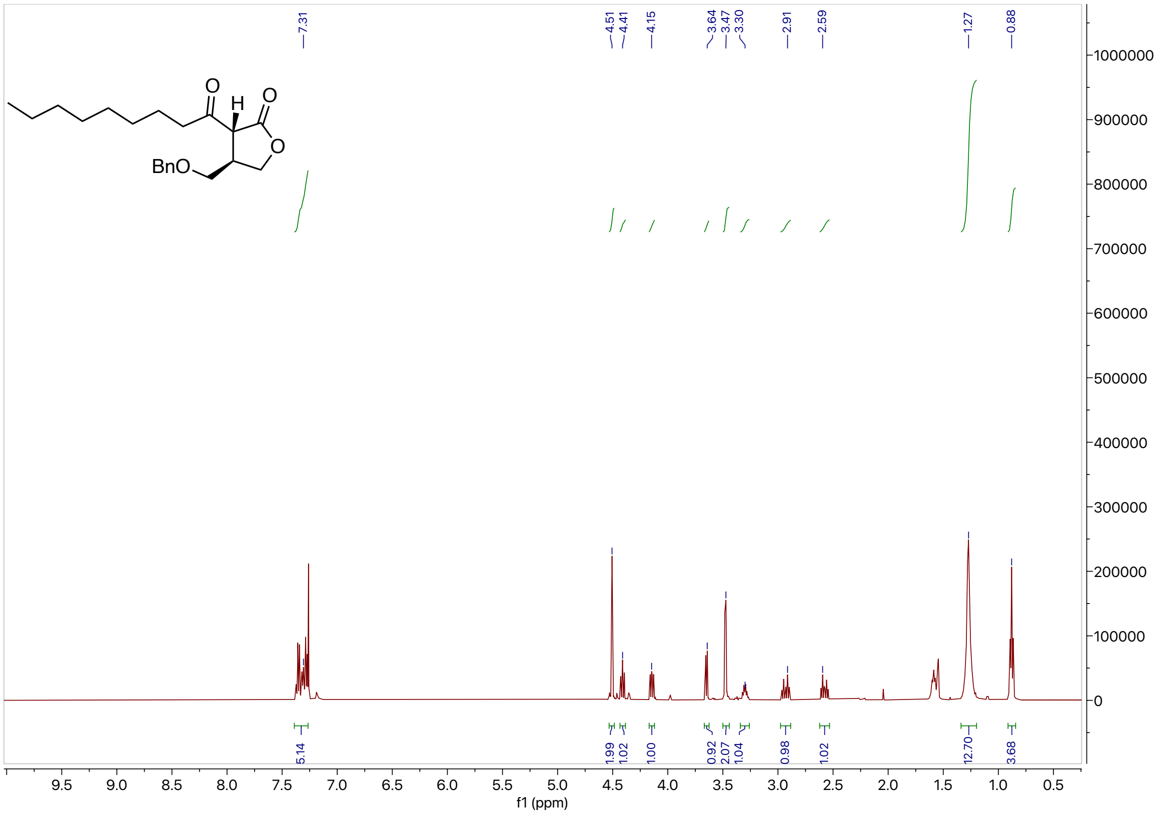

Figure S27. ^{1}H NMR Spectrum of Compound **5h**

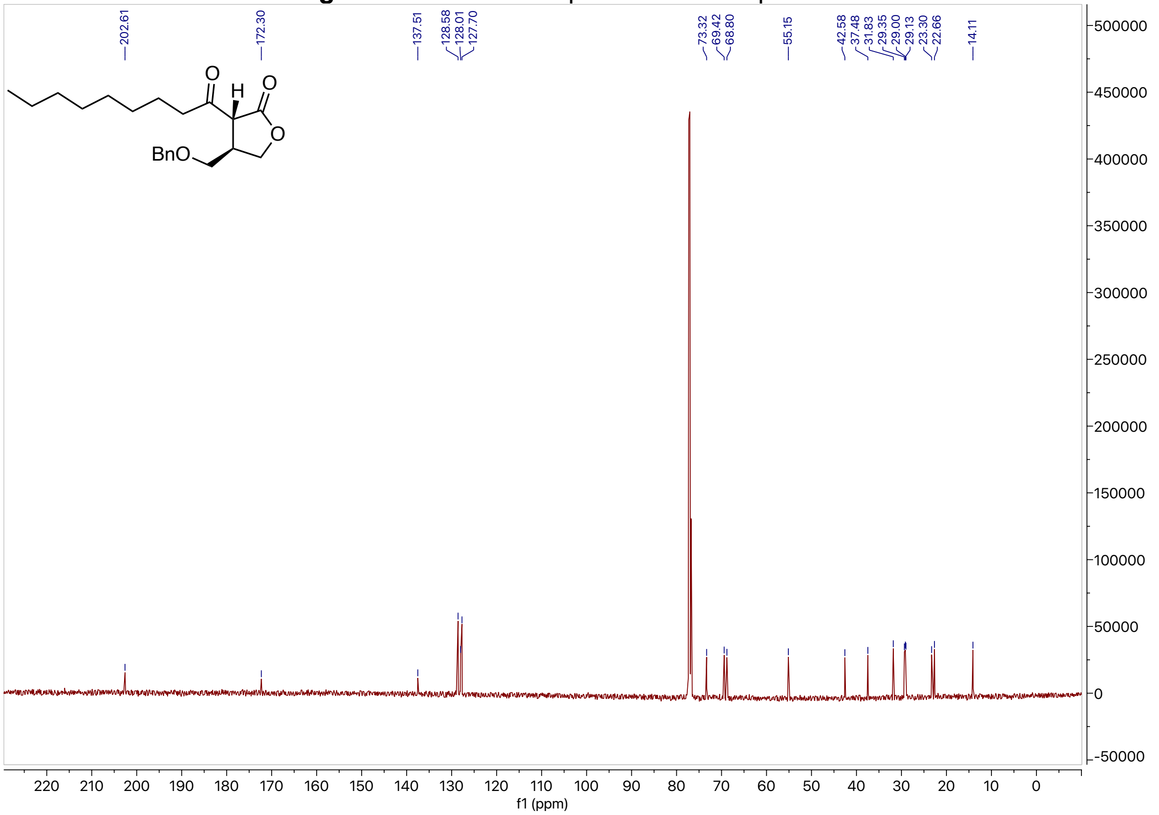

Figure S28. ^{13}C NMR Spectrum of Compound **5h**

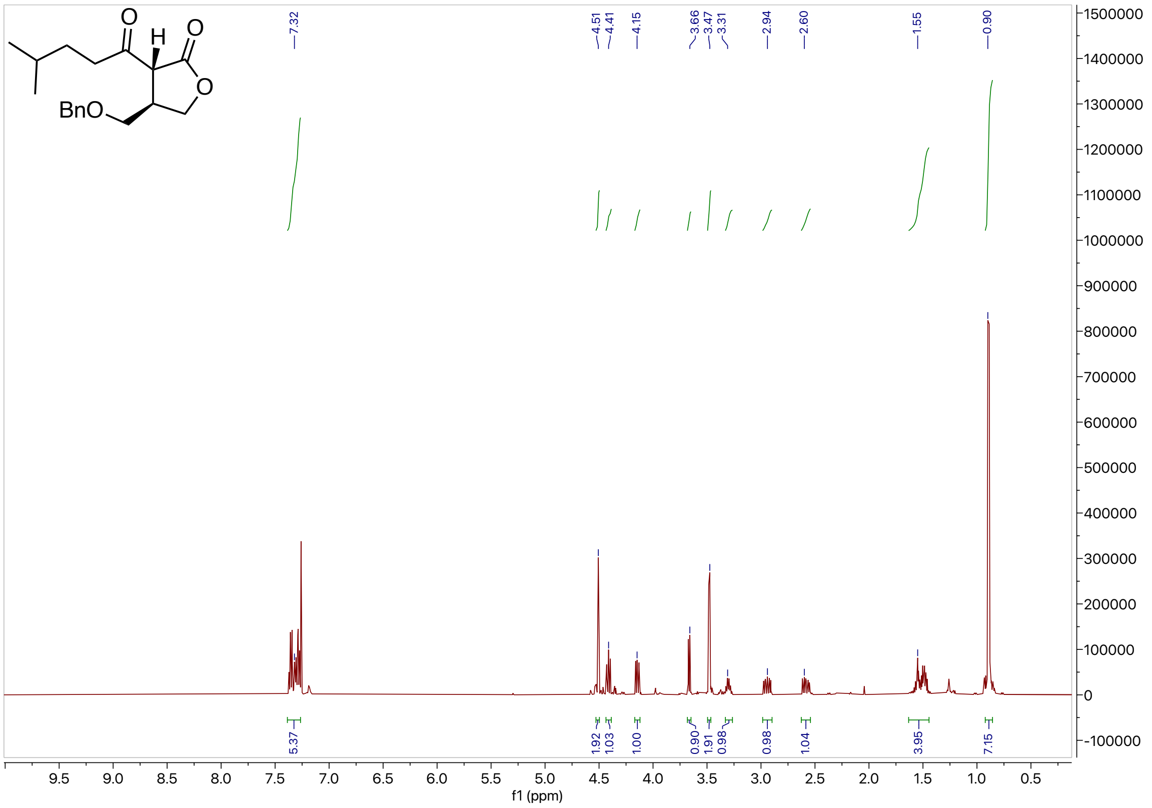

Figure S29. ^{1}H NMR Spectrum of Compound **5i**

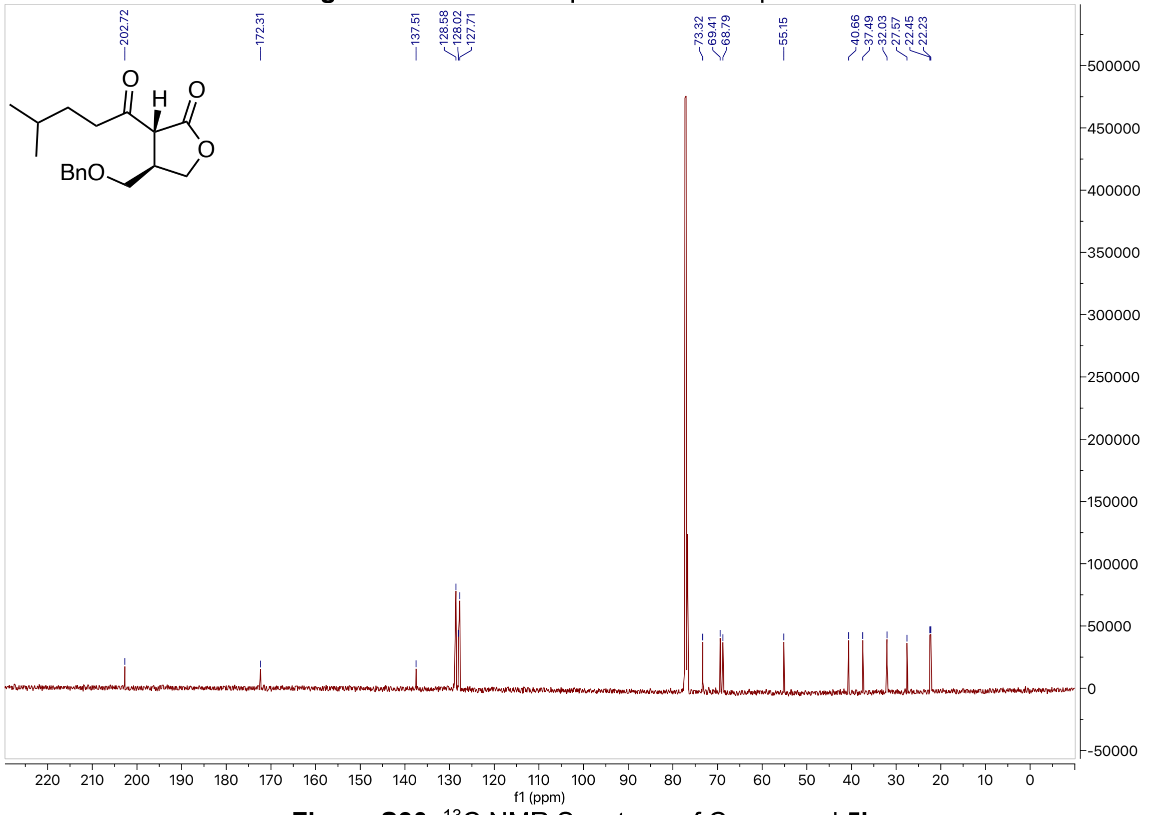

Figure S30. ^{13}C NMR Spectrum of Compound **5i**

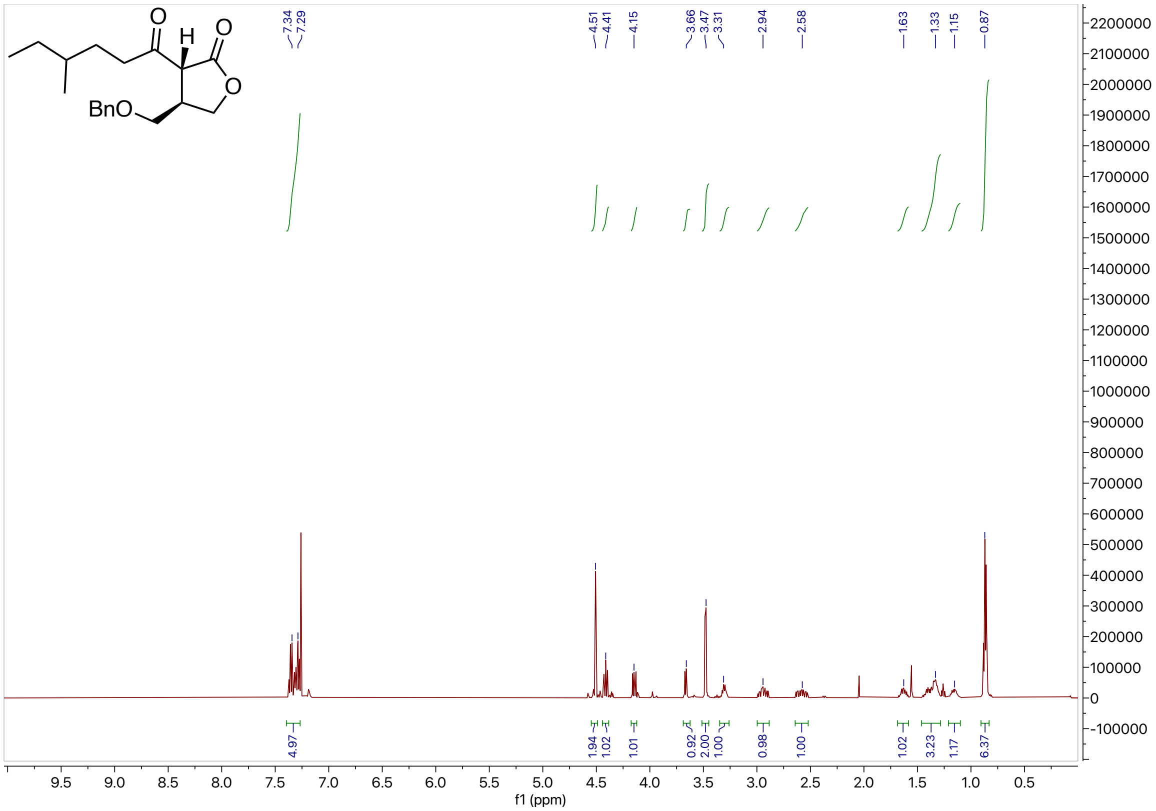

Figure S31. ^{1}H NMR Spectrum of Compound **5j**

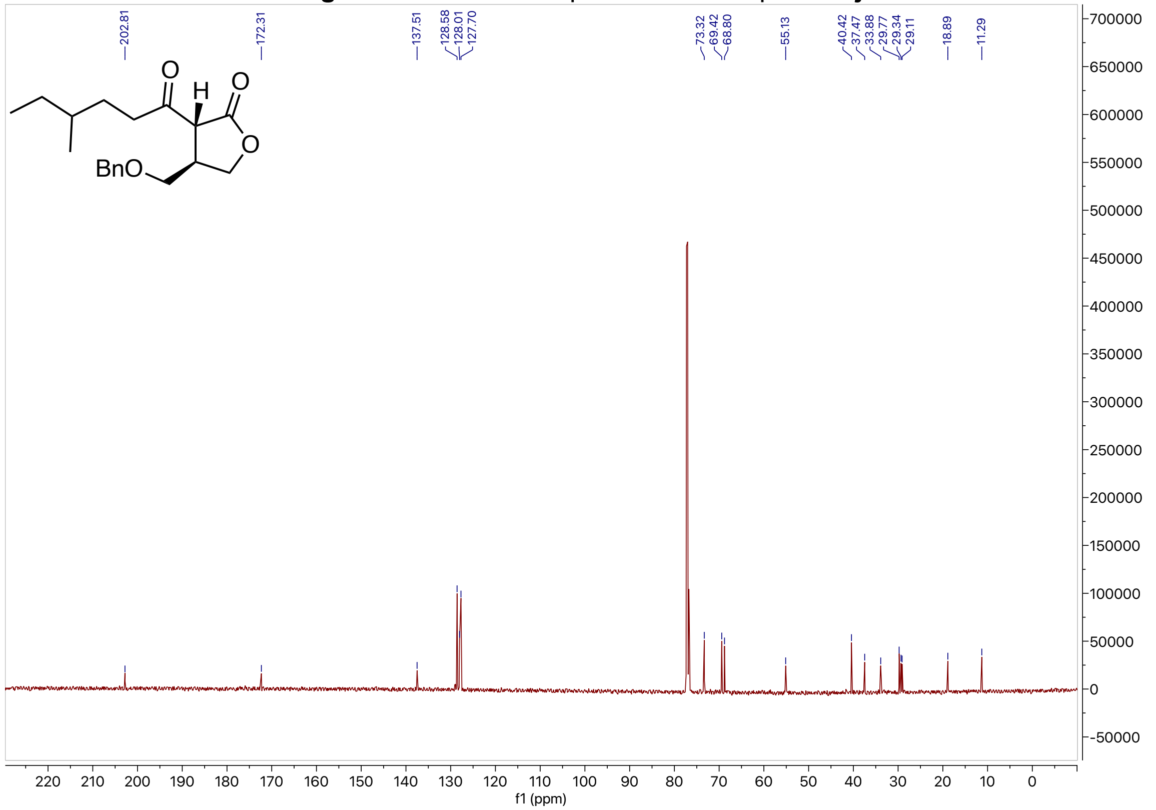

Figure S32. ^{13}C NMR Spectrum of Compound **5j**

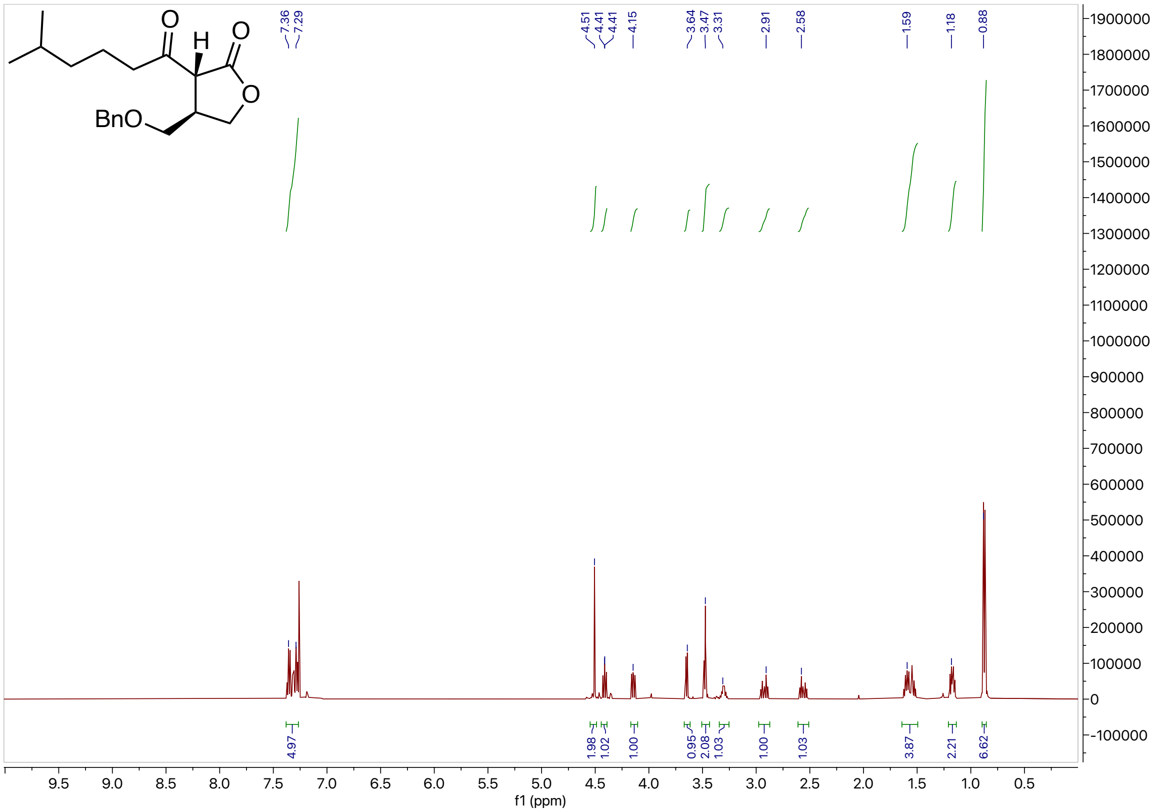

Figure S33. ^{1}H NMR Spectrum of Compound **5k**

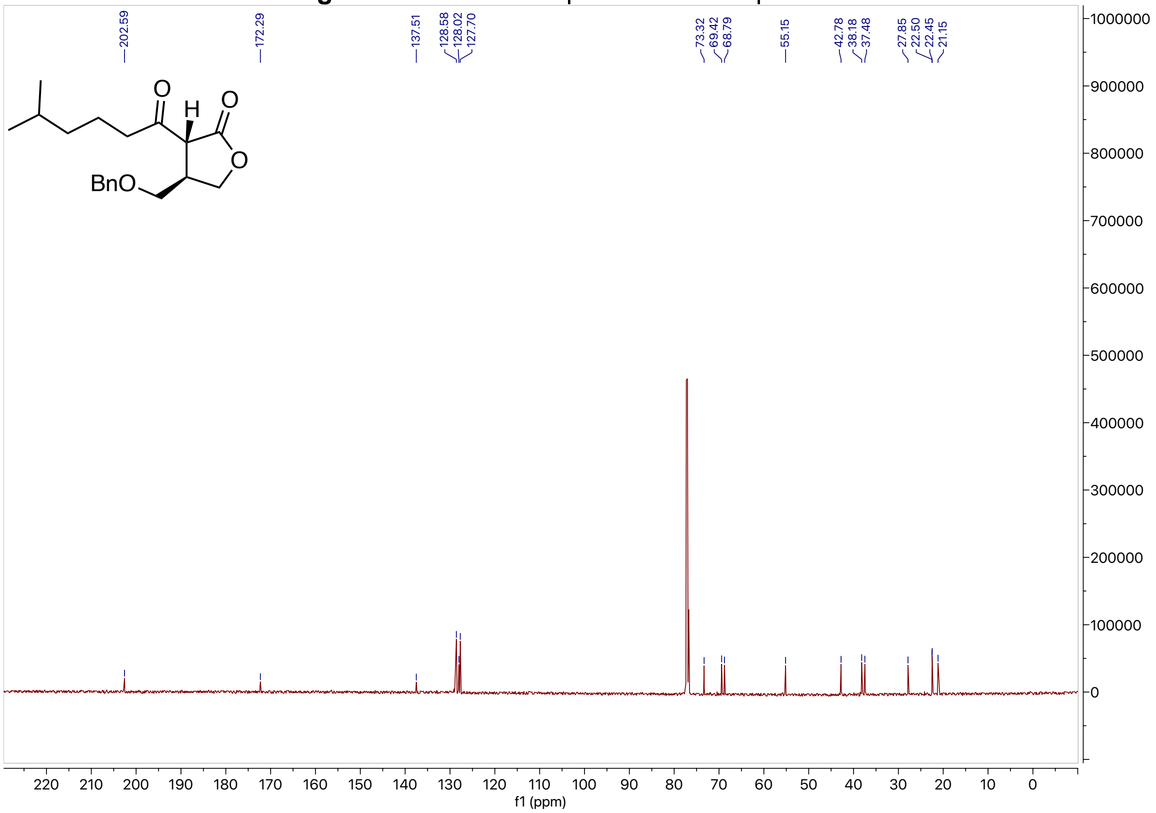

Figure 34. ^{13}C NMR Spectrum of Compound **5k**

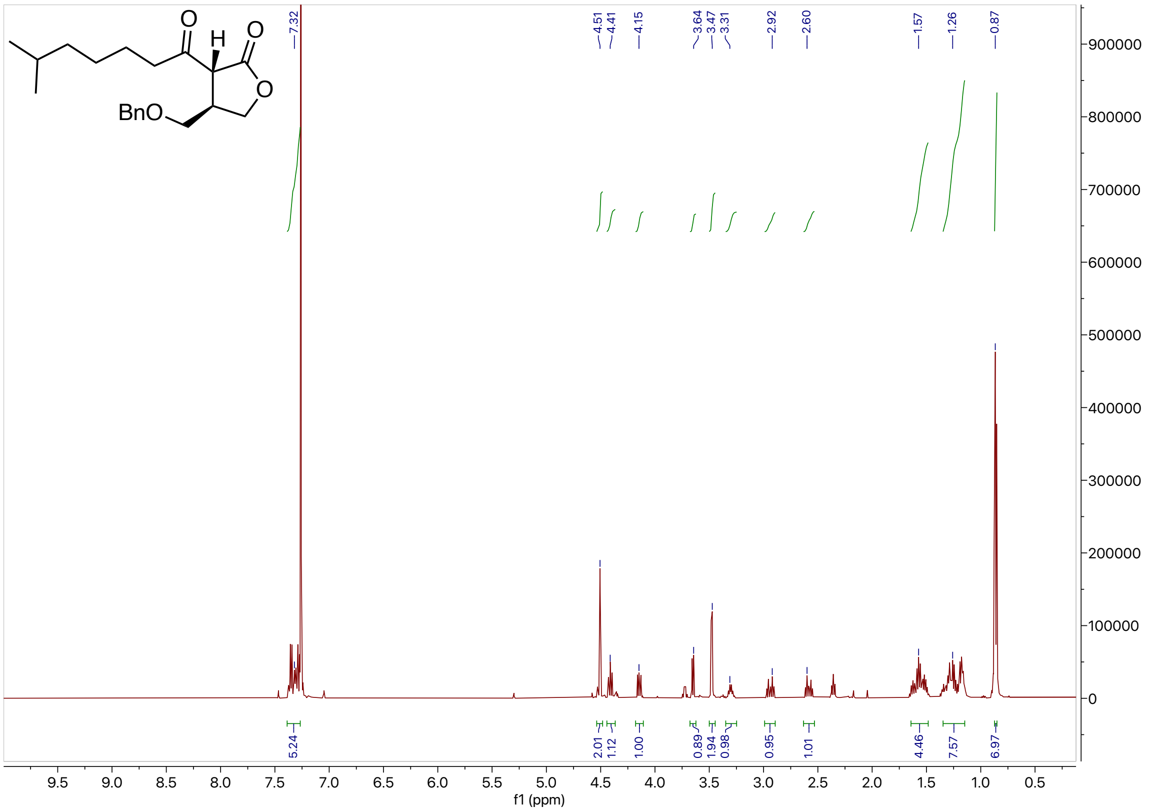

Figure S35. ^{1}H NMR Spectrum of Compound **5l**

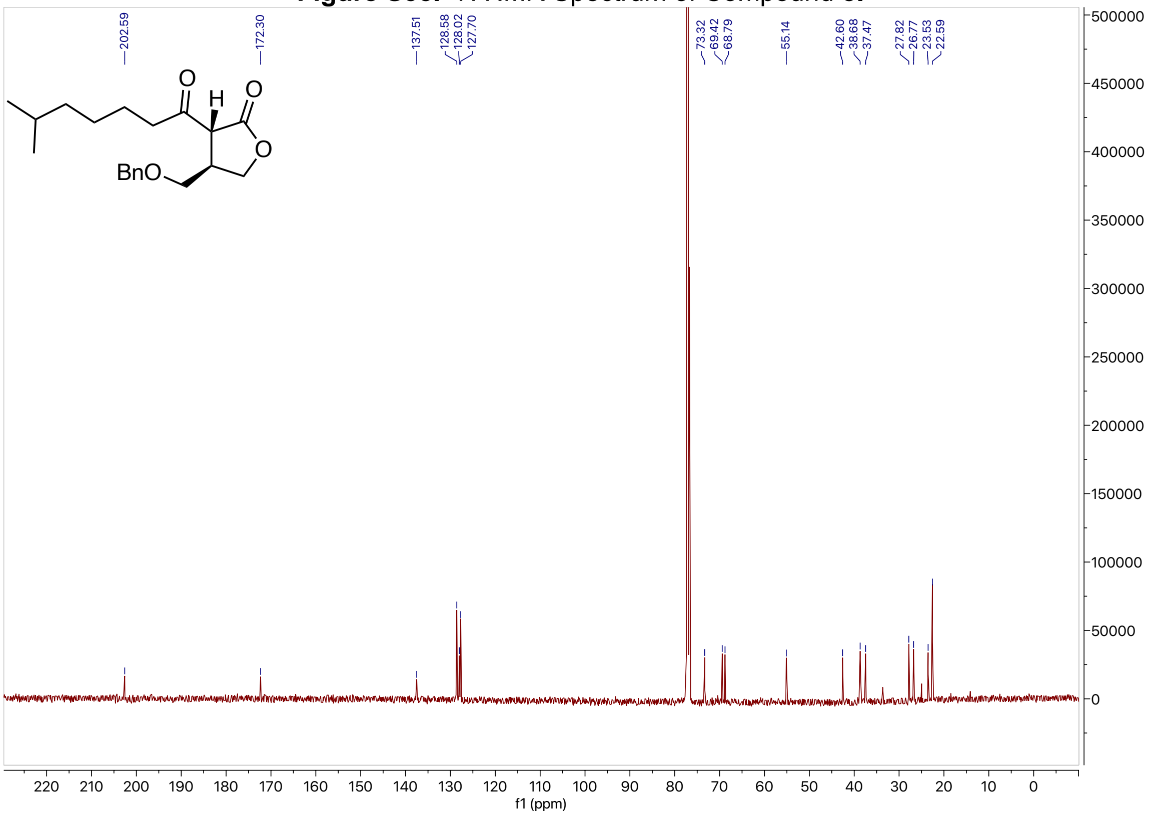

Figure S36. ^{13}C NMR Spectrum of Compound **5l**

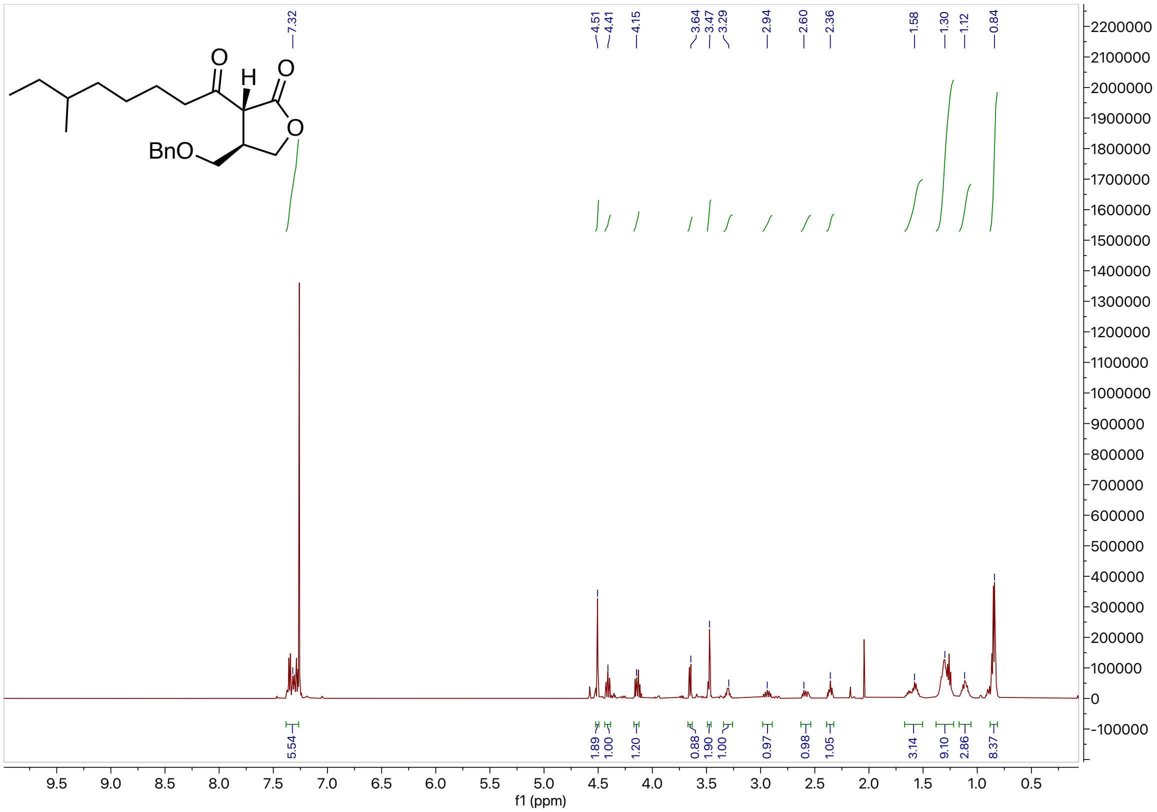

Figure S37. ^{1}H NMR Spectrum of Compound **5m**

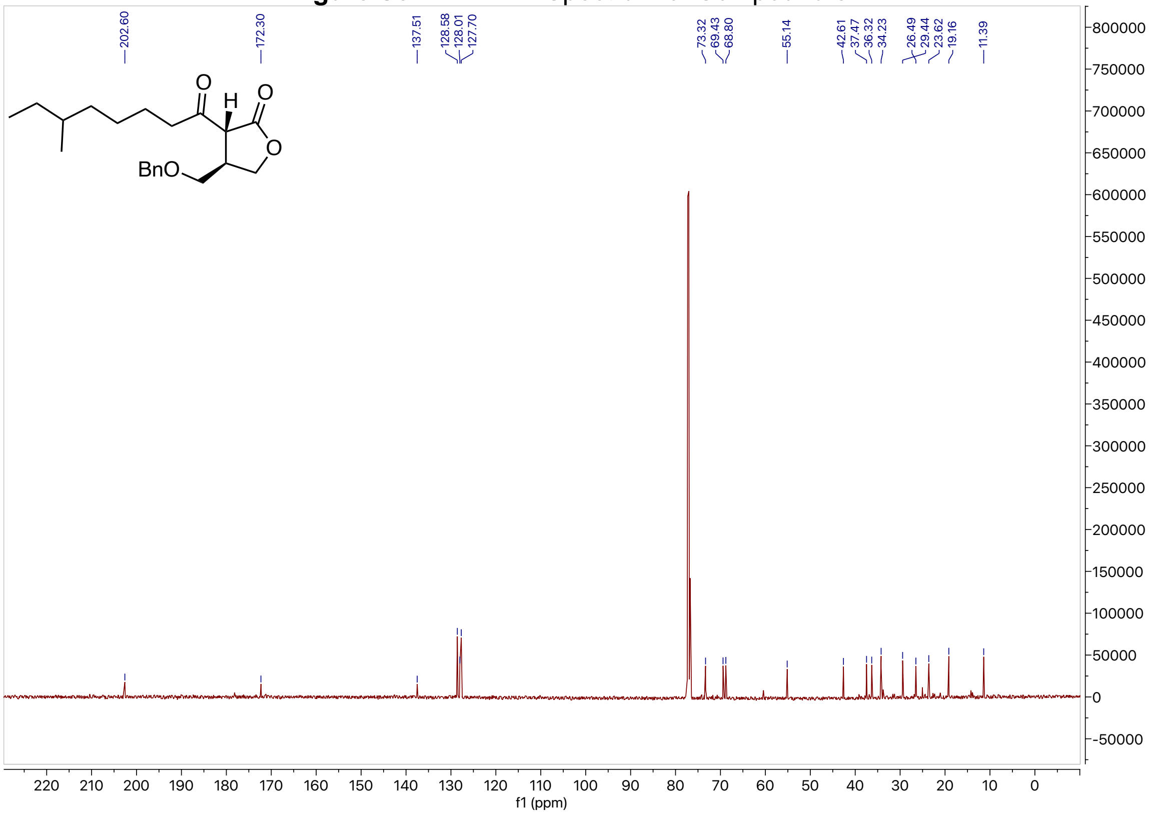

Figure S38. ^{13}C NMR Spectrum of Compound **5m**

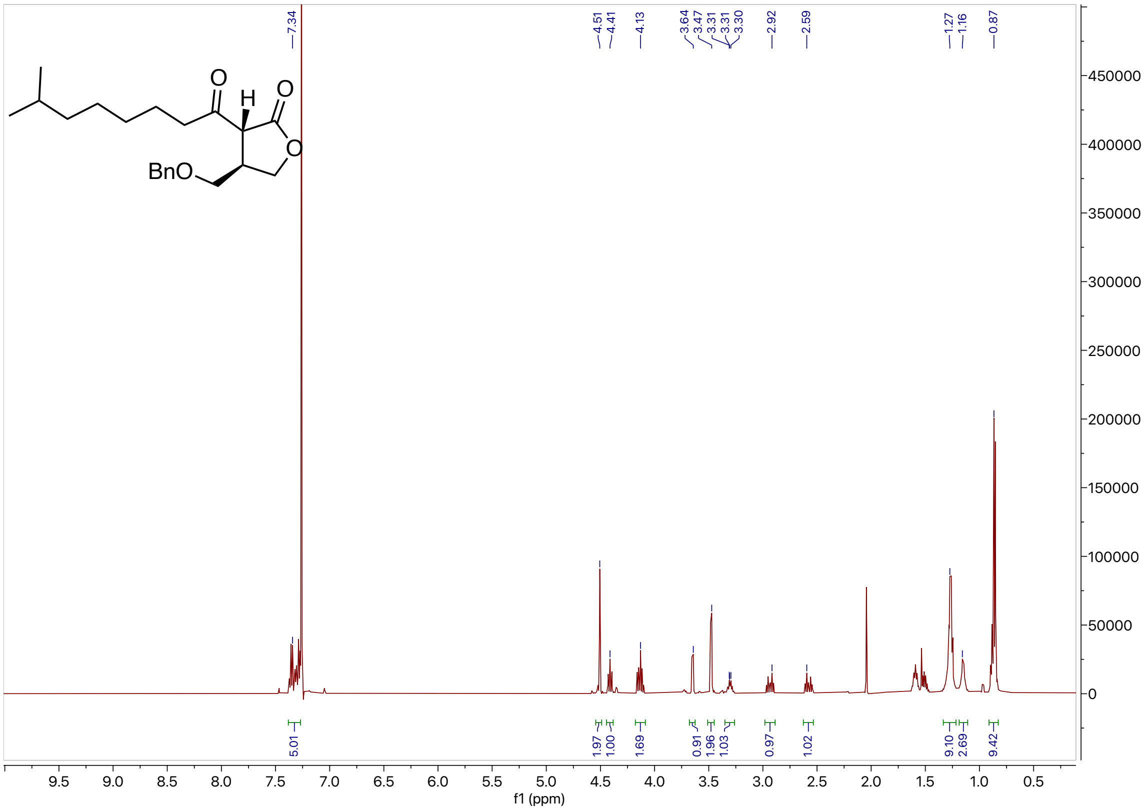

Figure S39. ^{1}H NMR Spectrum of Compound **5n**

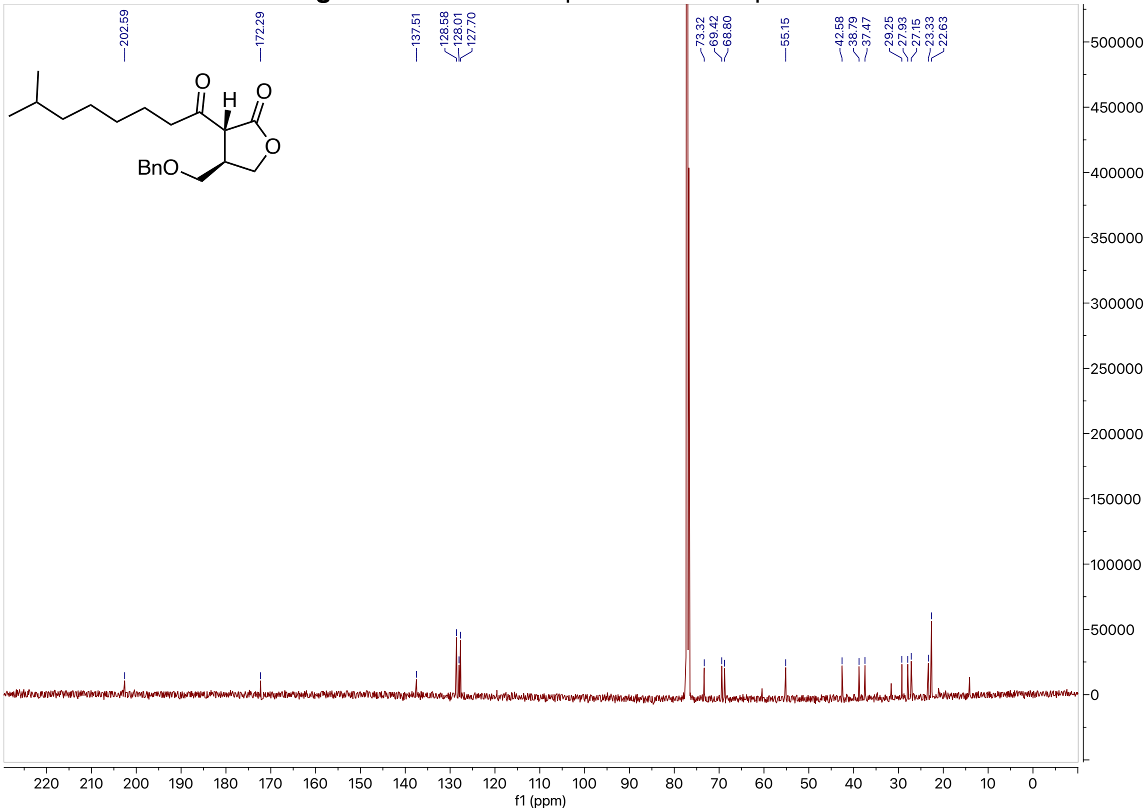

Figure S40. ^{13}C NMR Spectrum of Compound **5n**

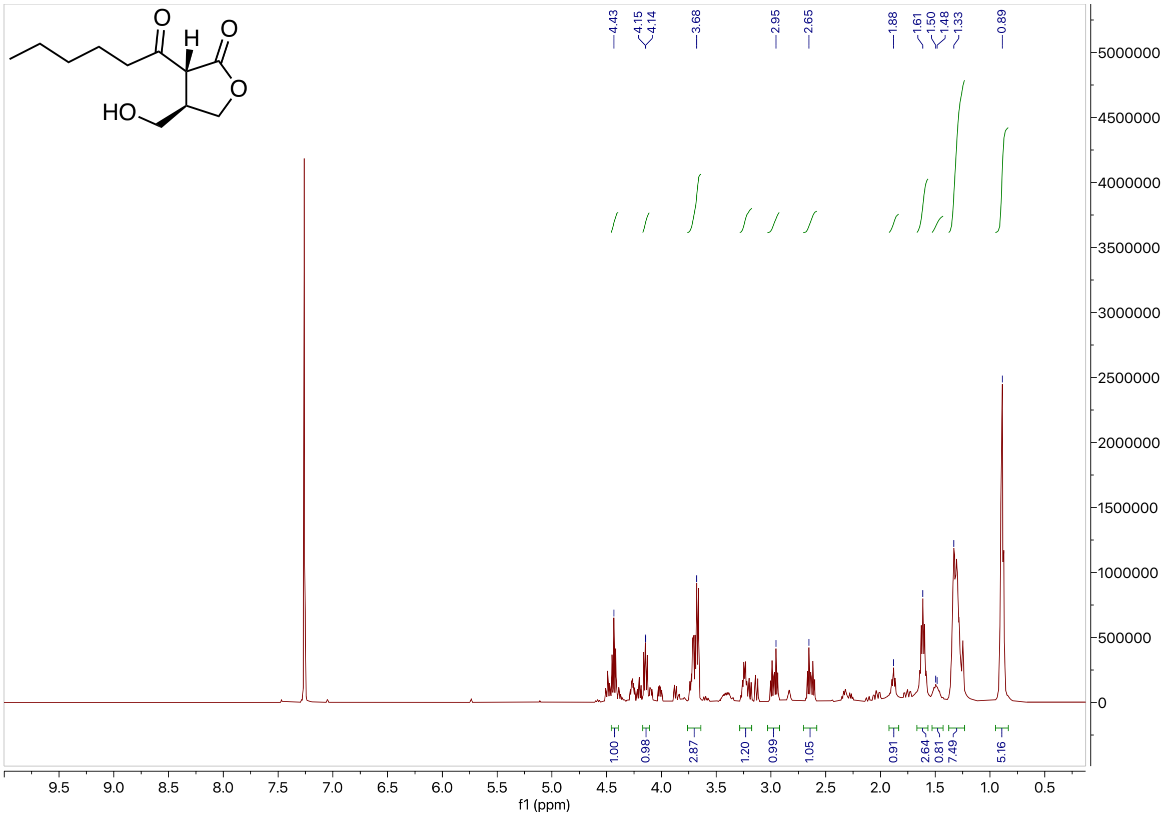

Figure S41. ^{1}H NMR Spectrum of Compound **6e**

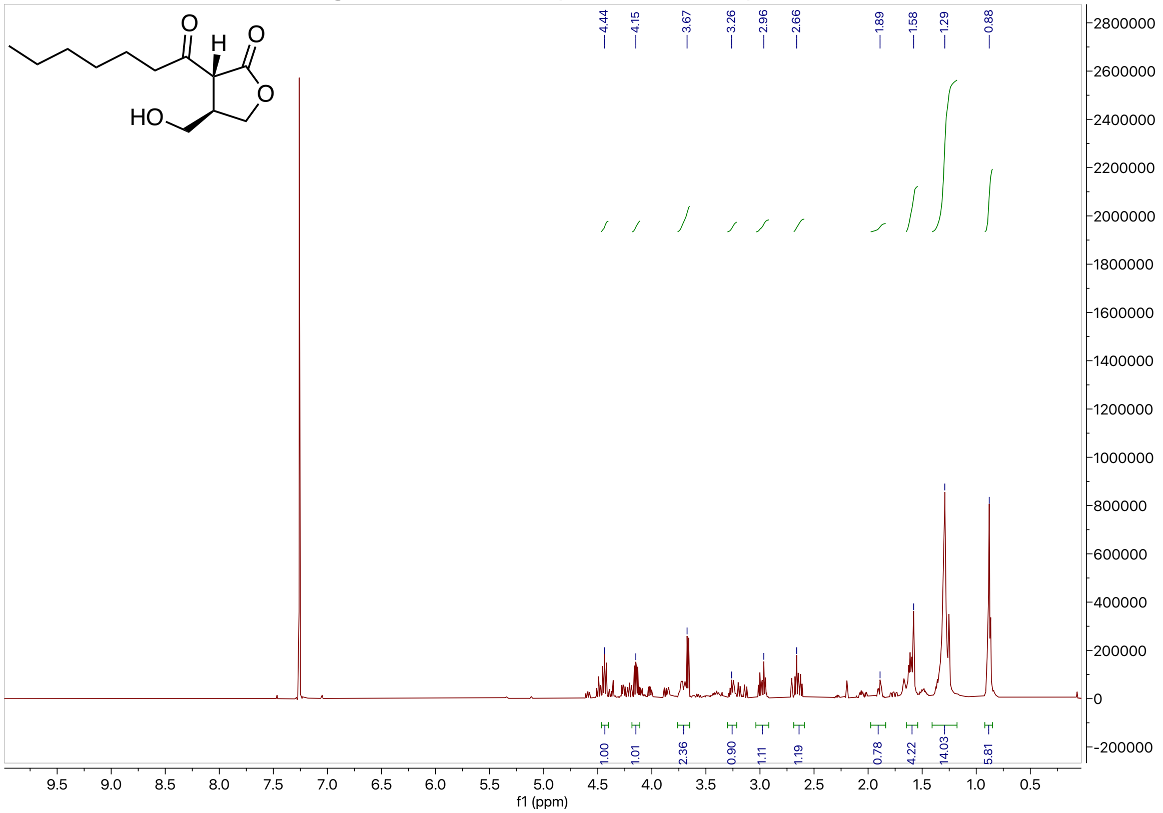

Figure S42. ^{1}H NMR Spectrum of Compound **6f**

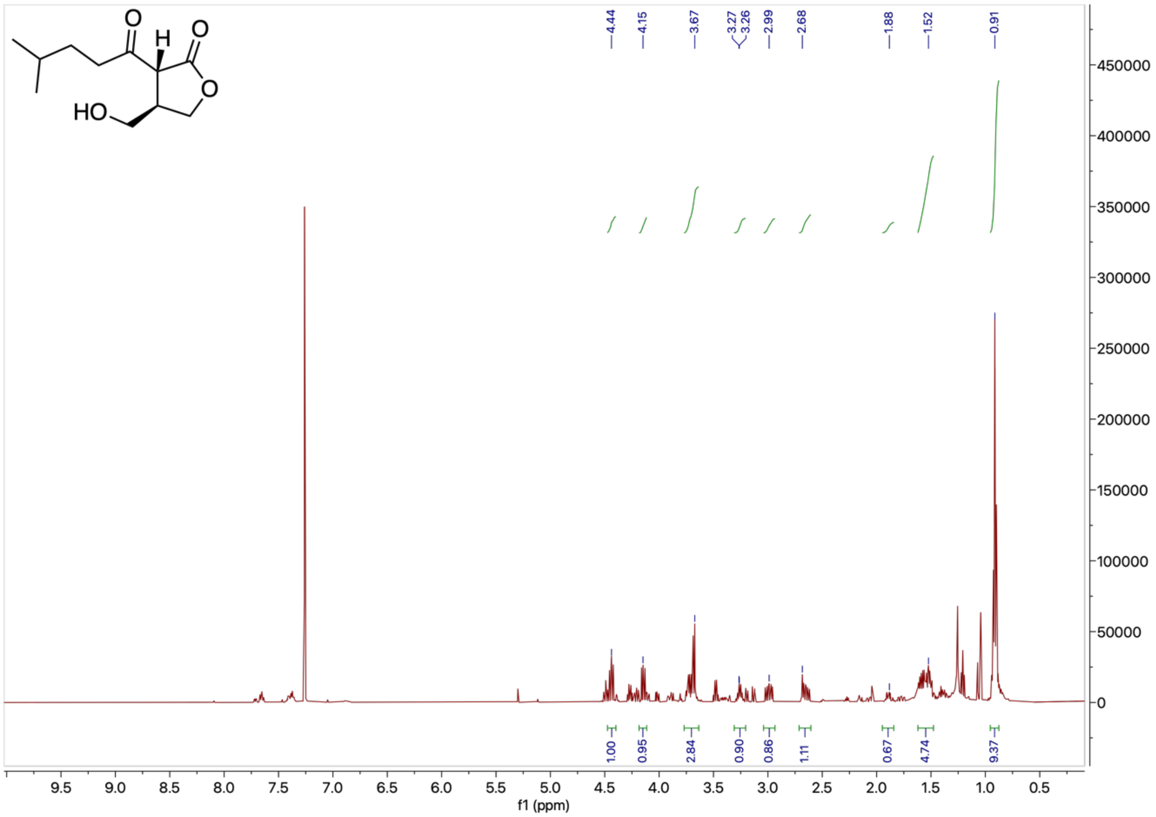

Figure S43. ^{1}H NMR Spectrum of Compound **6i**

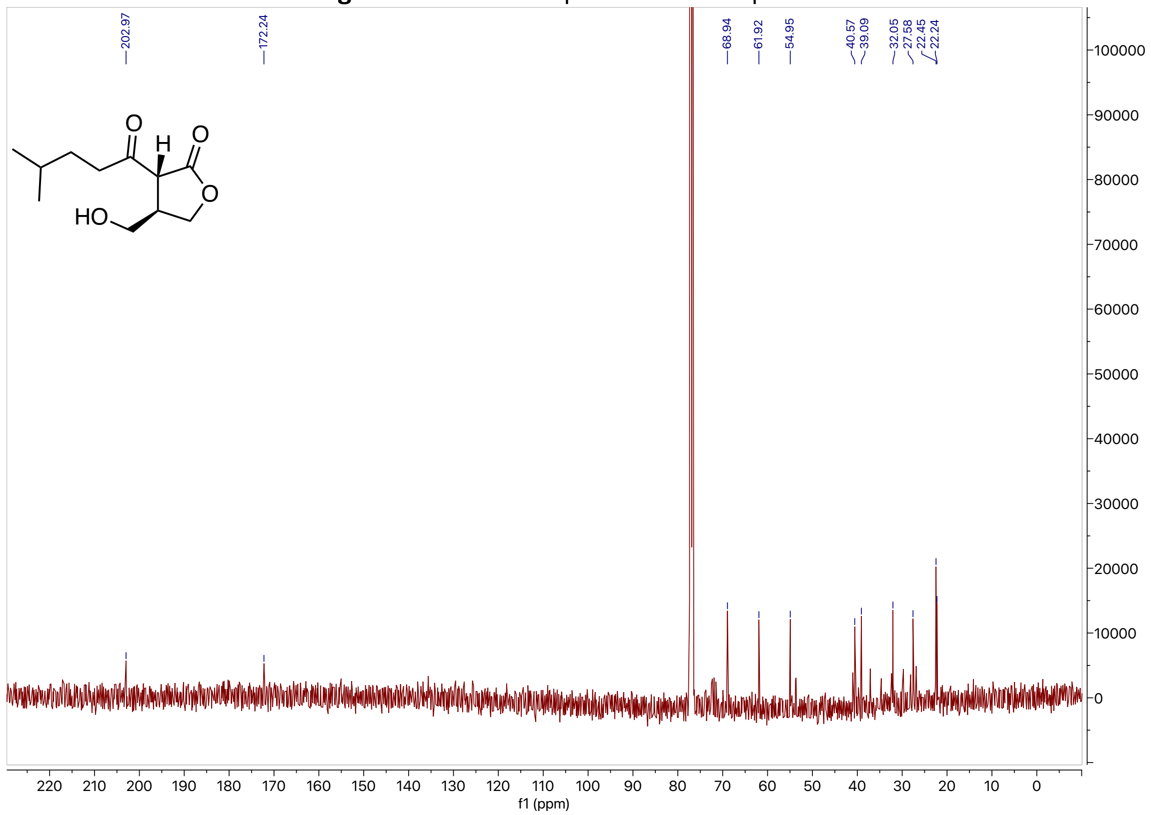

Figure S44. ^{13}C NMR Spectrum of Compound **6i**

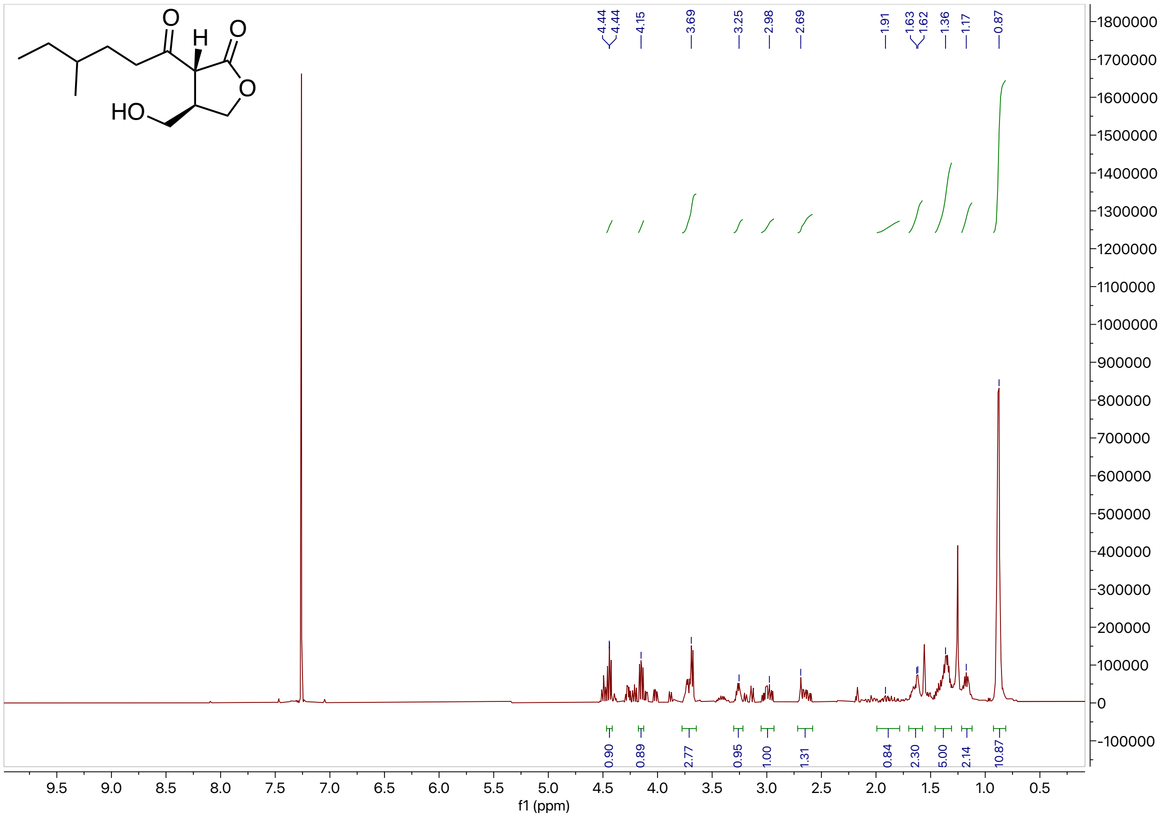

Figure S45. ^{1}H NMR Spectrum of Compound **6j**

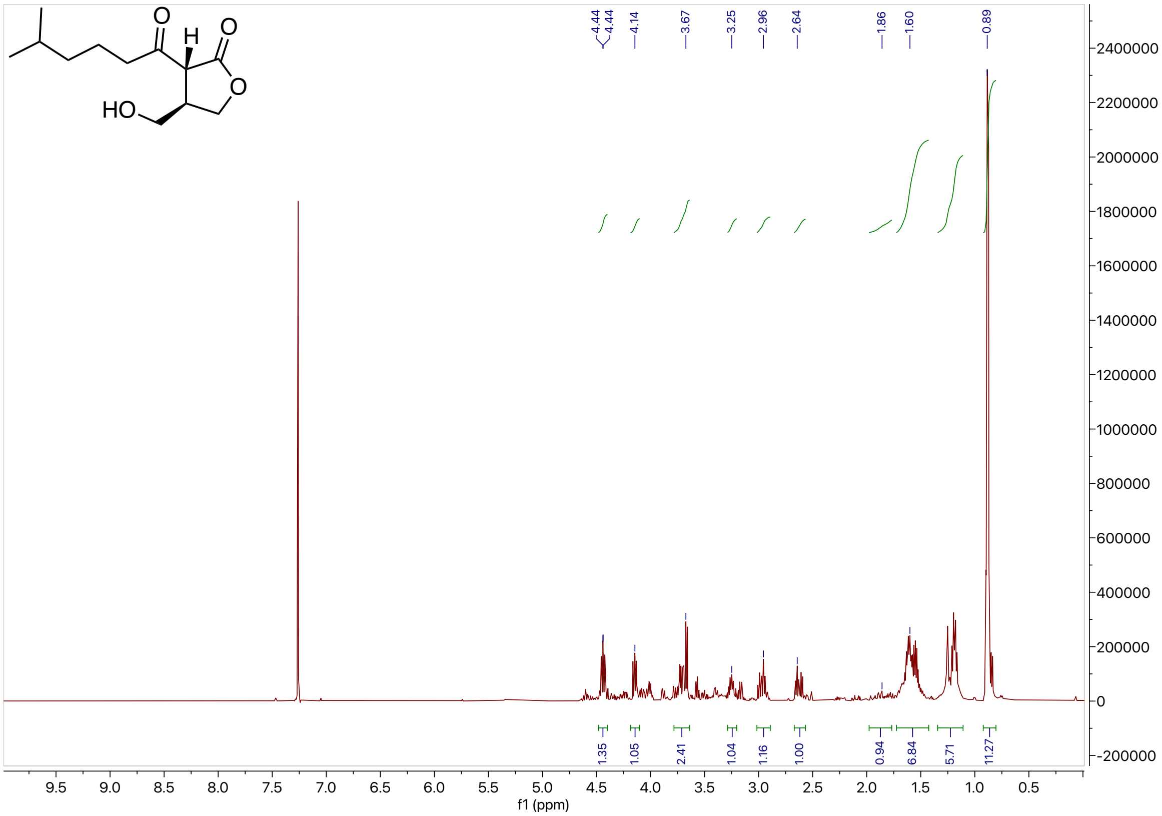

Figure S46. ^{1}H NMR Spectrum of Compound **6k**

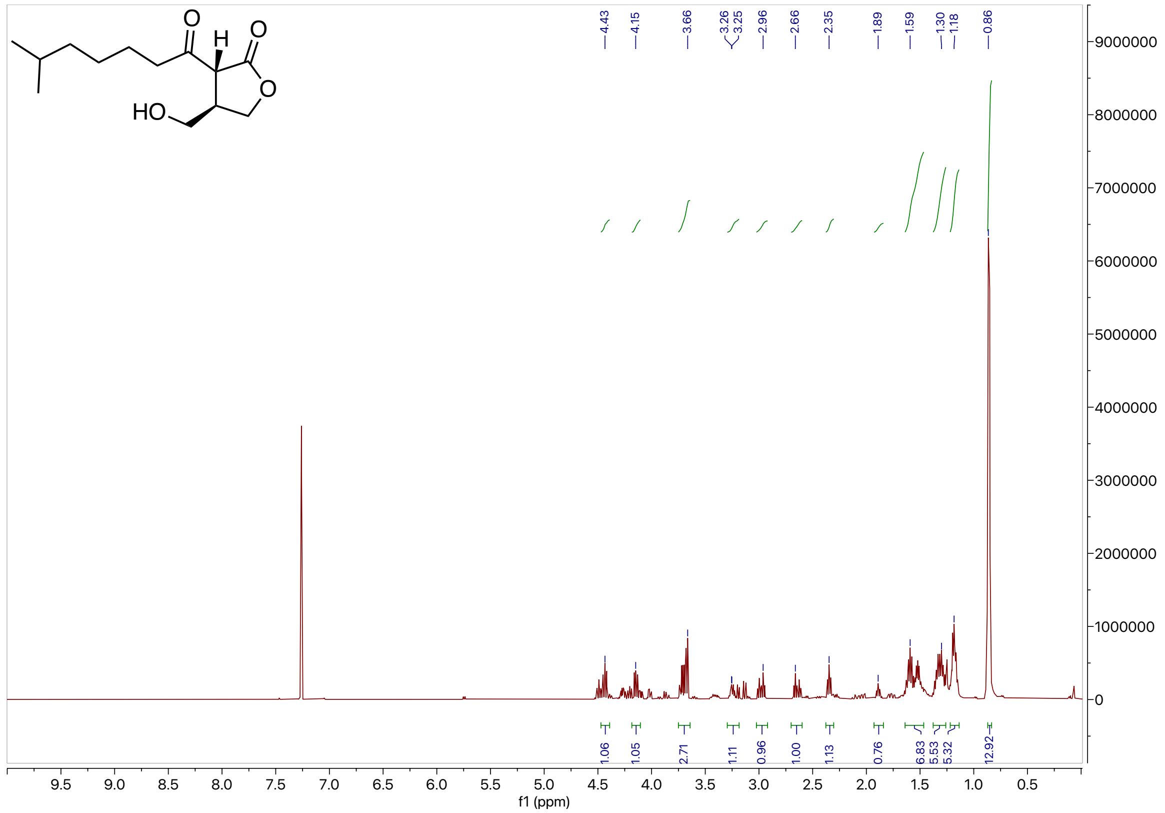

Figure S47. ^{1}H NMR Spectrum of Compound **6l**

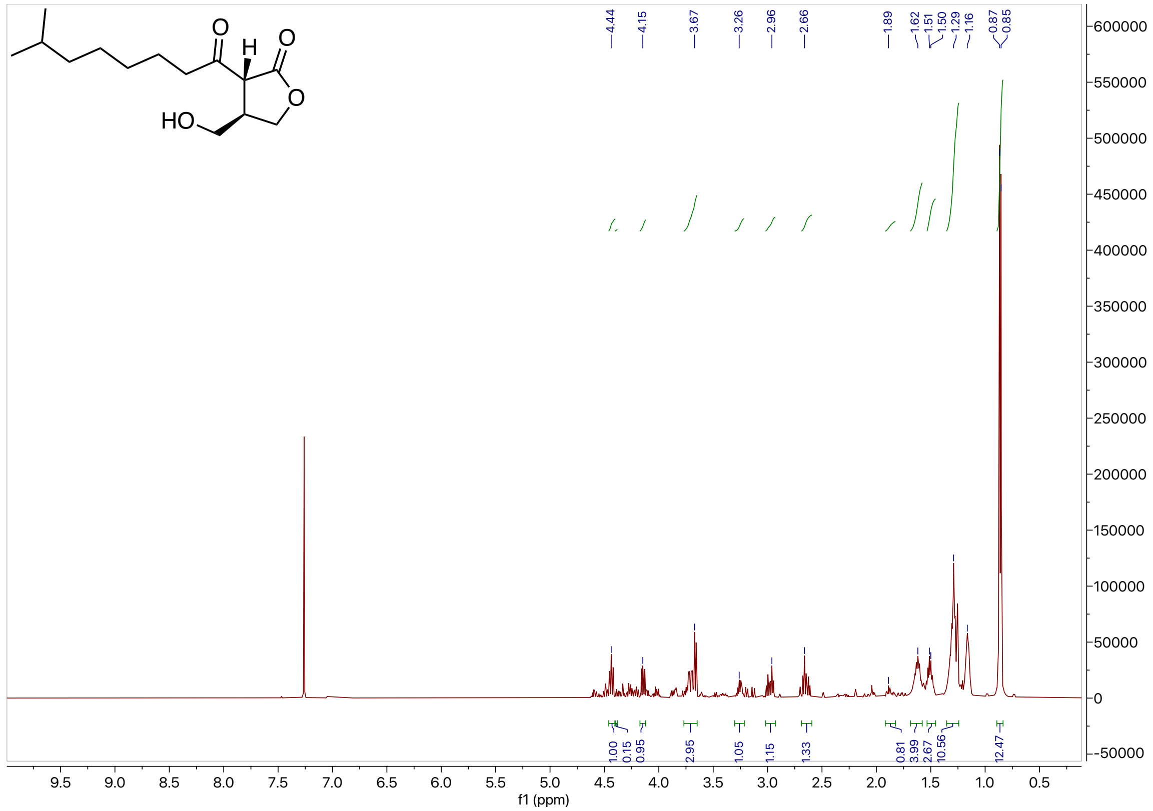

Figure S48. ^{1}H NMR Spectrum of Compound **6n**

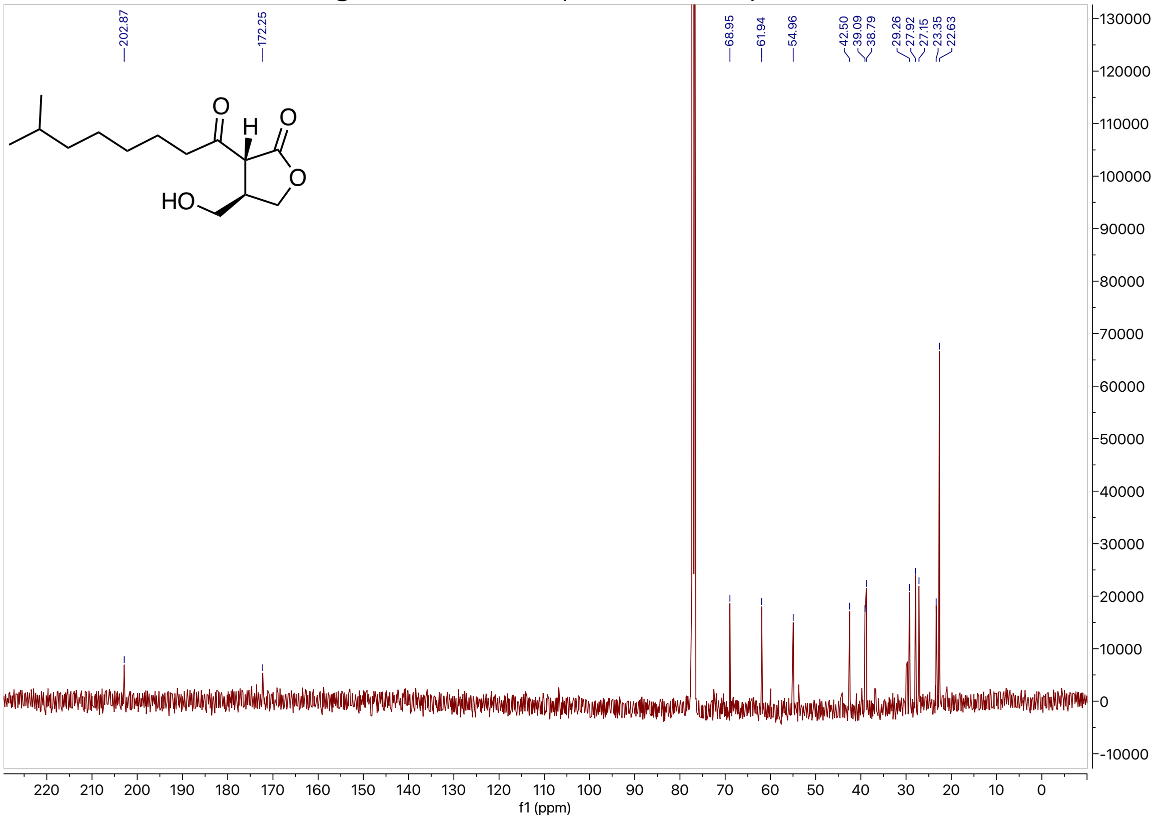

Figure S49. ^{13}C NMR Spectrum of Compound **6n**

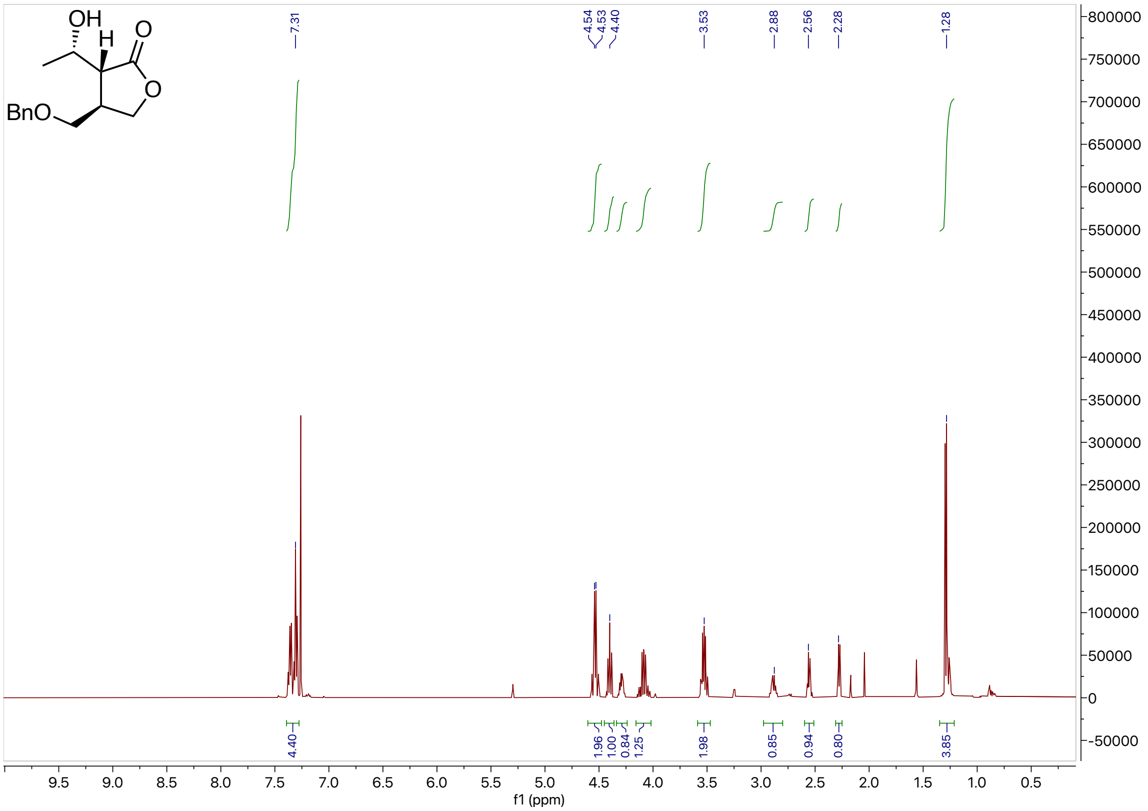

Figure S50. ^{1}H NMR Spectrum of Compound **7a**

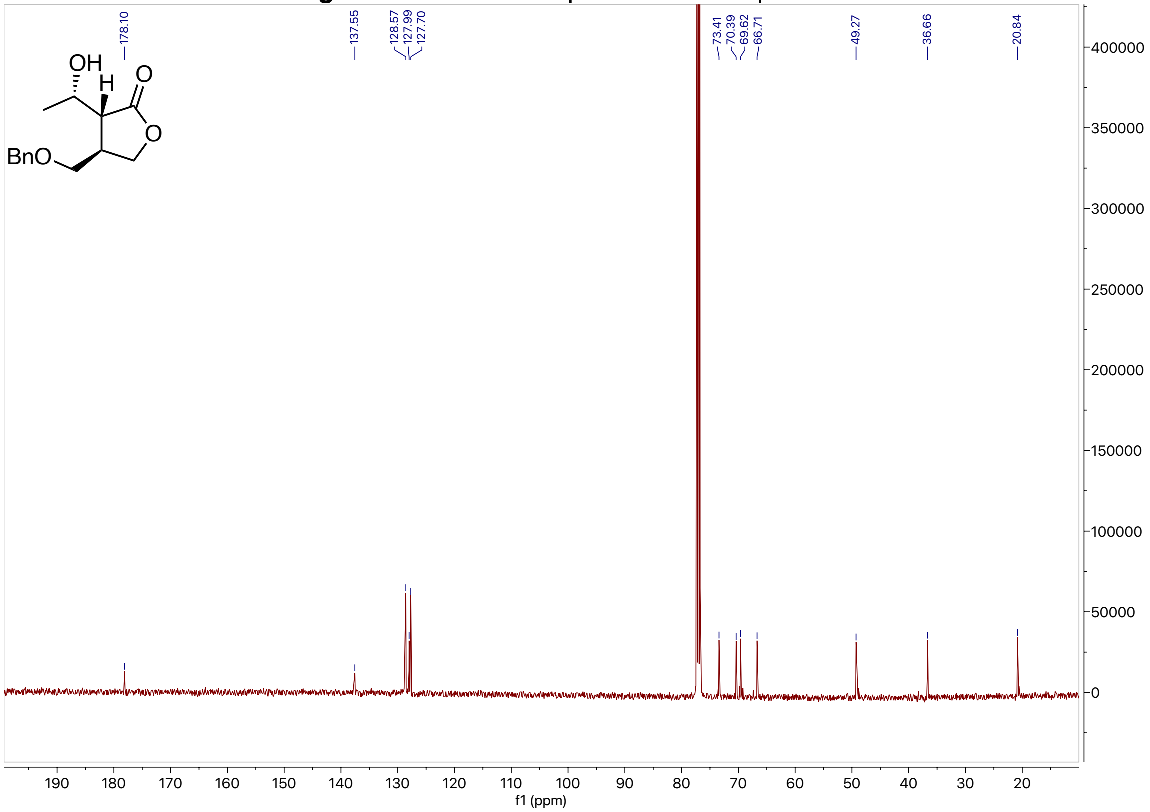

Figure S51. ^{13}C NMR Spectrum of Compound **7a**

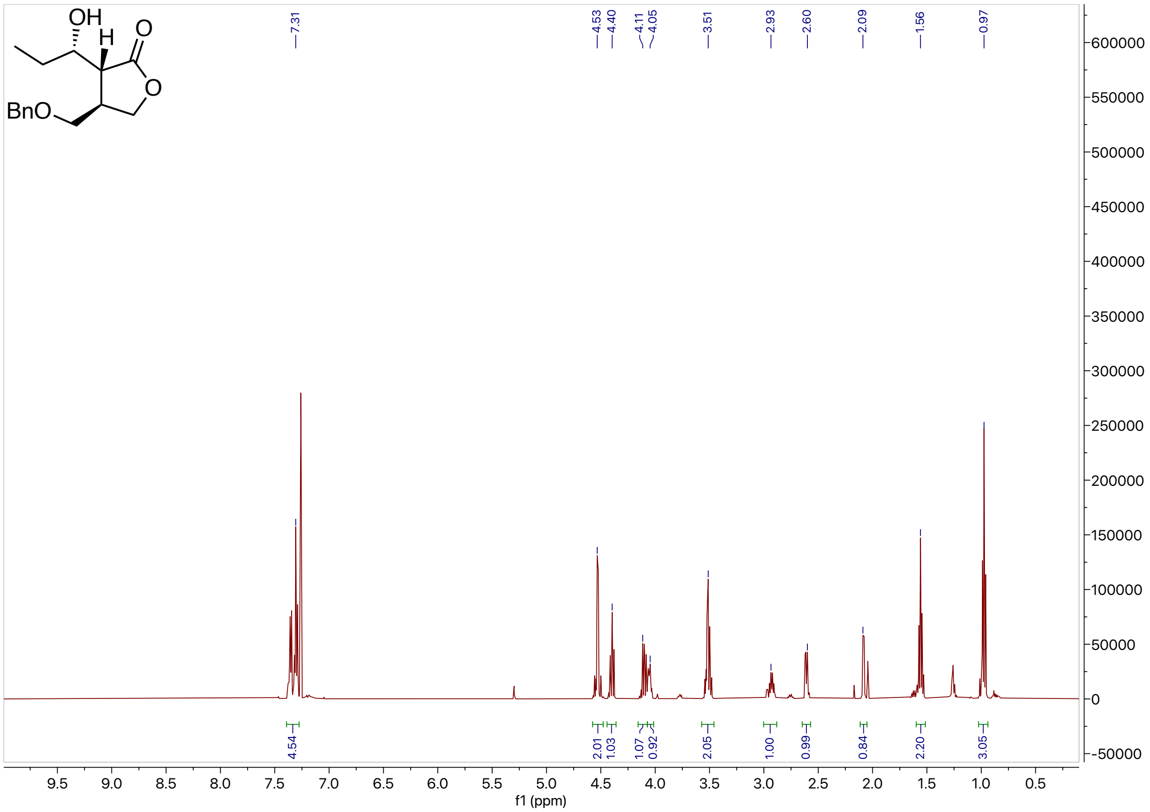

Figure S52. ^{1}H NMR Spectrum of Compound **7b**

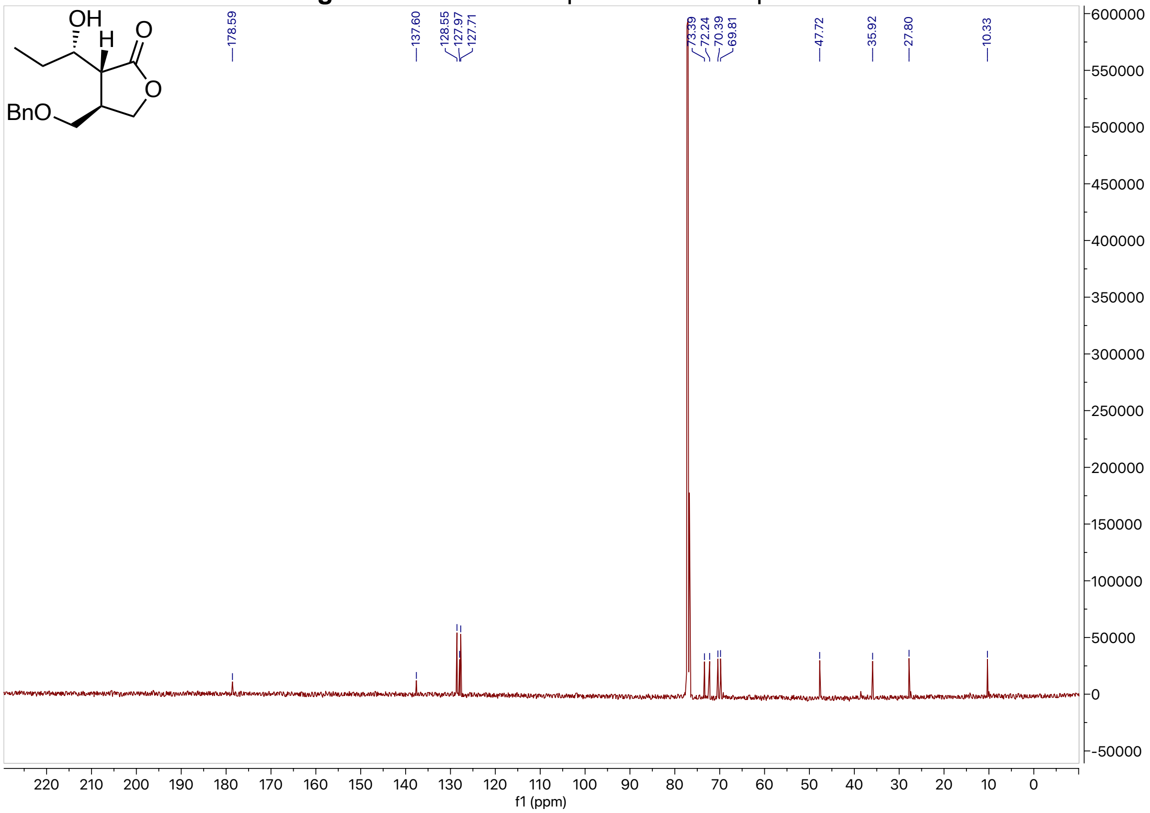

Figure S53. ^{13}C NMR Spectrum of Compound **7b**

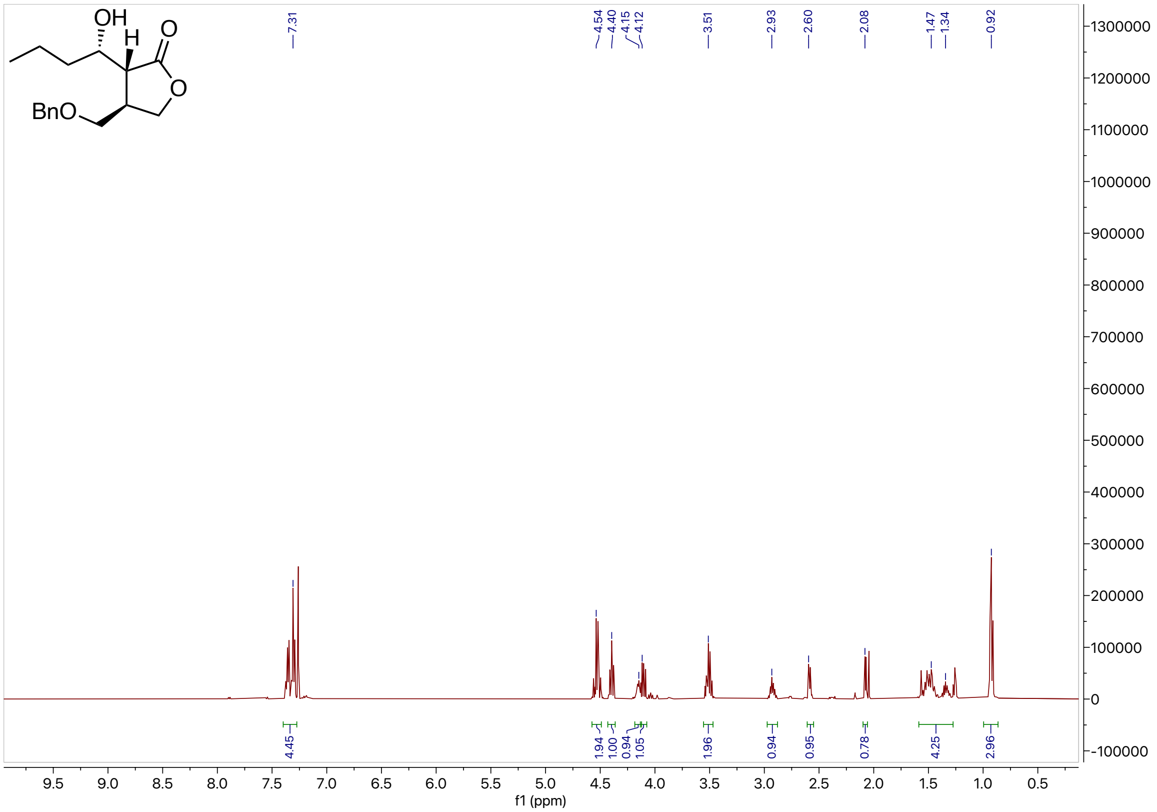

Figure S54. ^{1}H NMR Spectrum of Compound **7c**

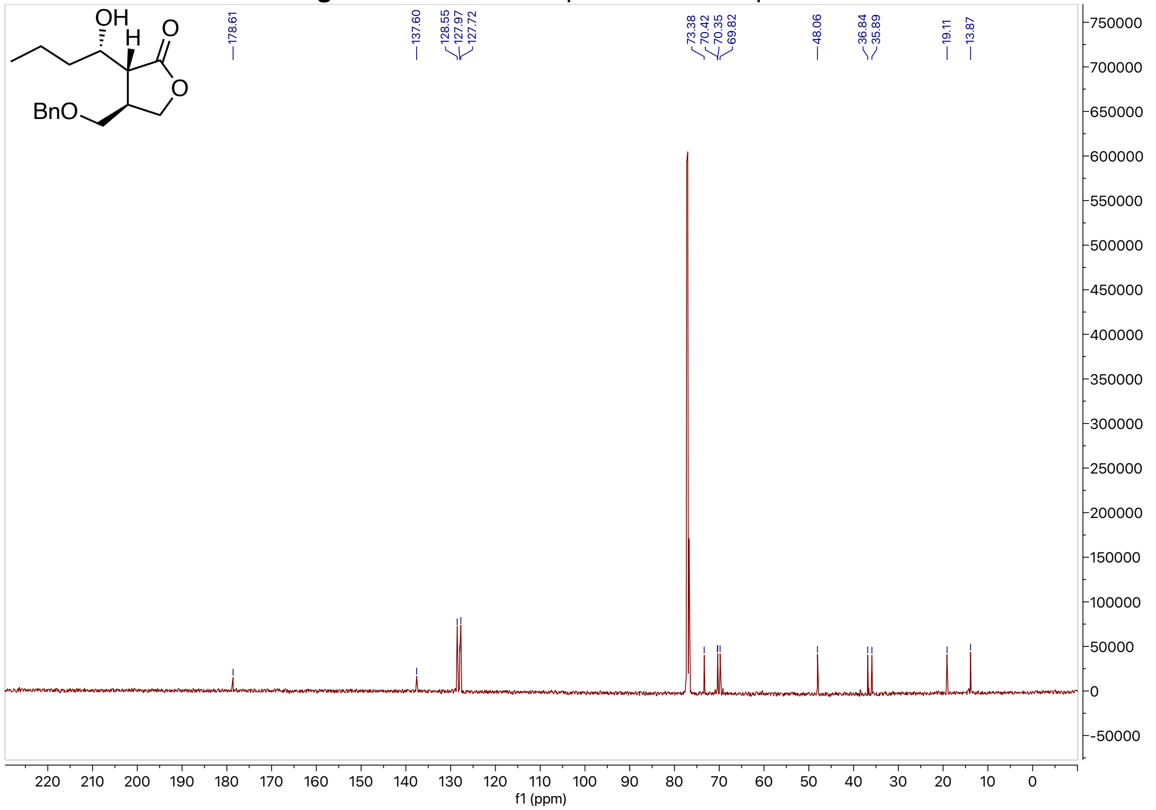

Figure S55. ^{13}C NMR Spectrum of Compound **7c**

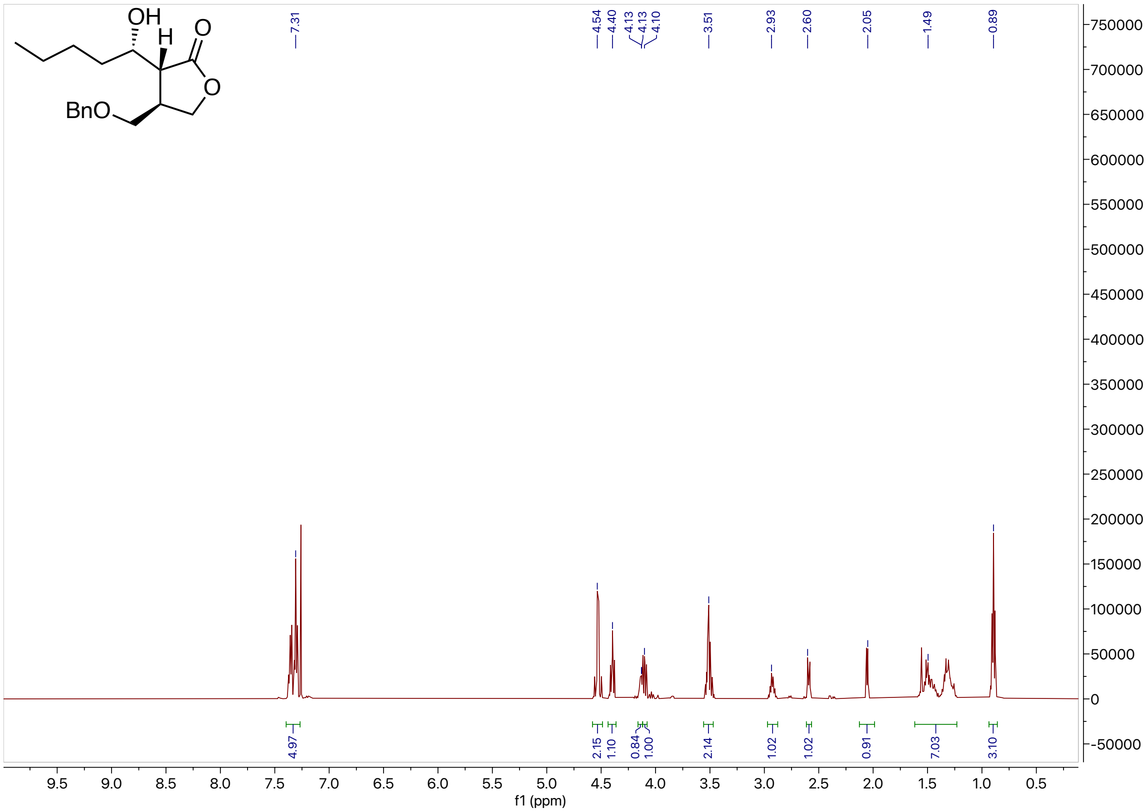

Figure S56. ^{1}H NMR Spectrum of Compound **7d**

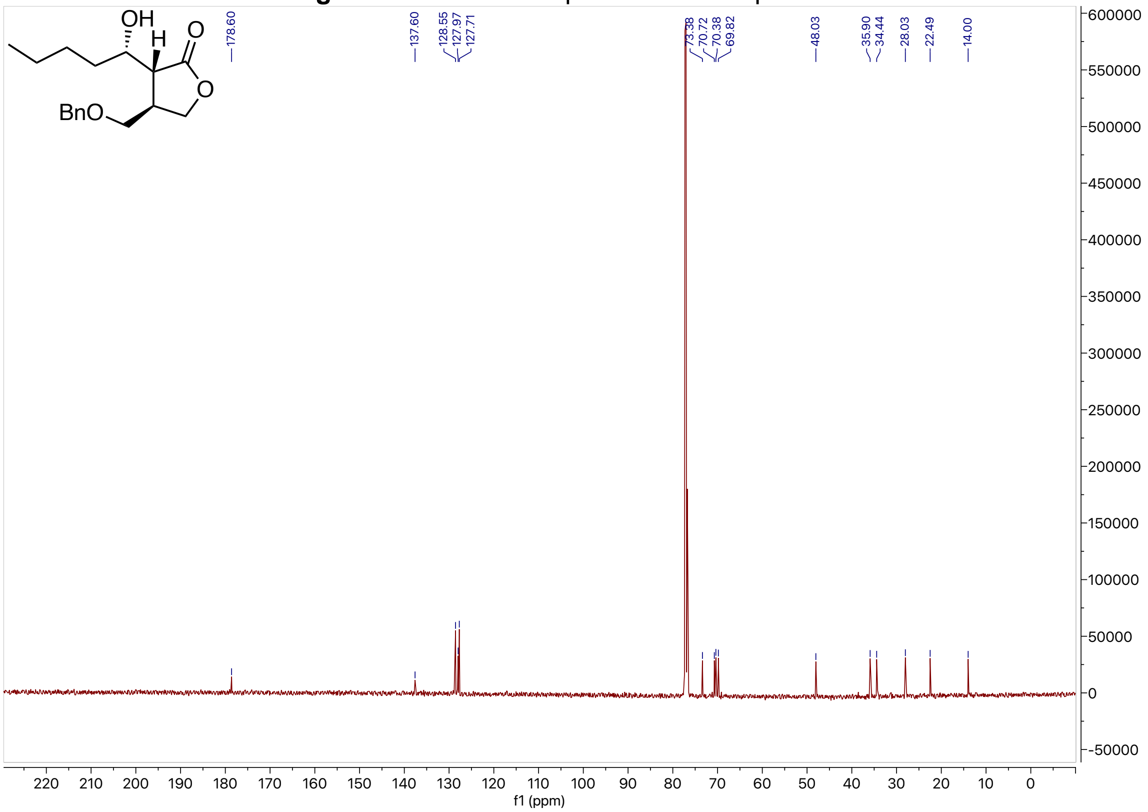

Figure S57. ^{13}C NMR Spectrum of Compound **7d**

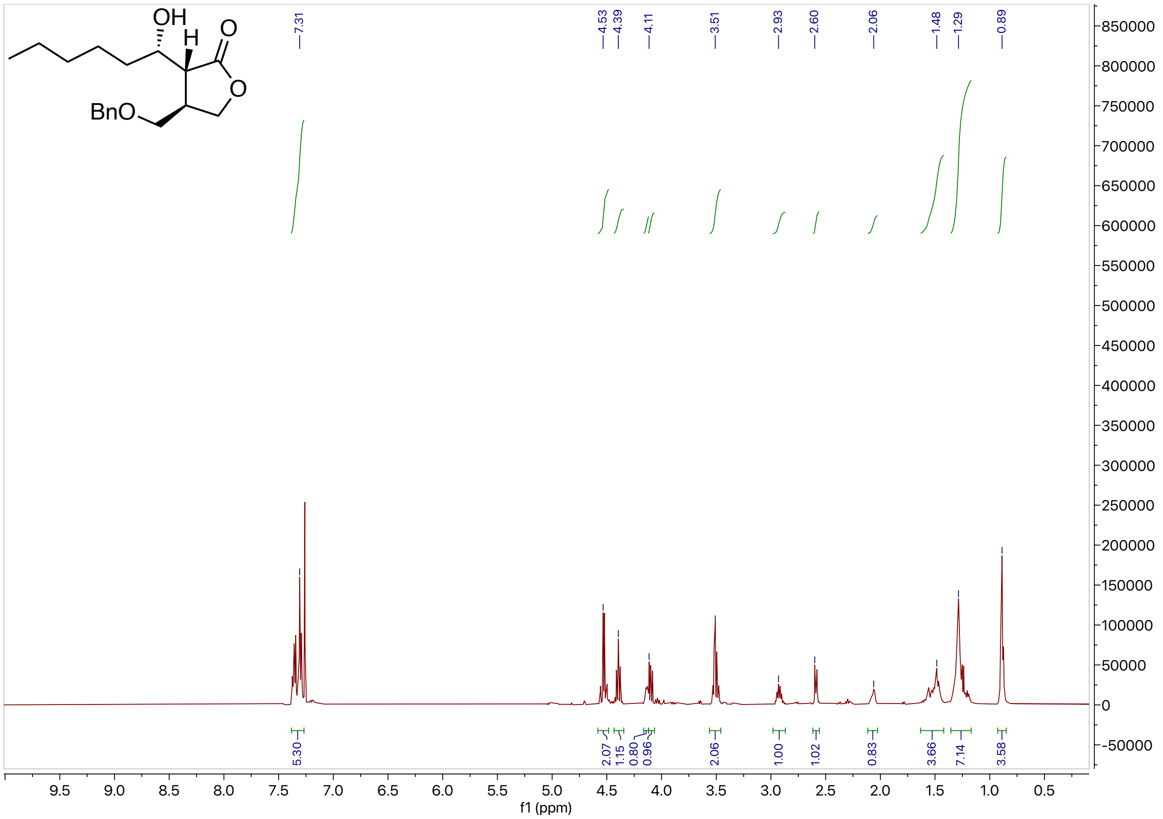

Figure S58. ^{1}H NMR Spectrum of Compound **7e**

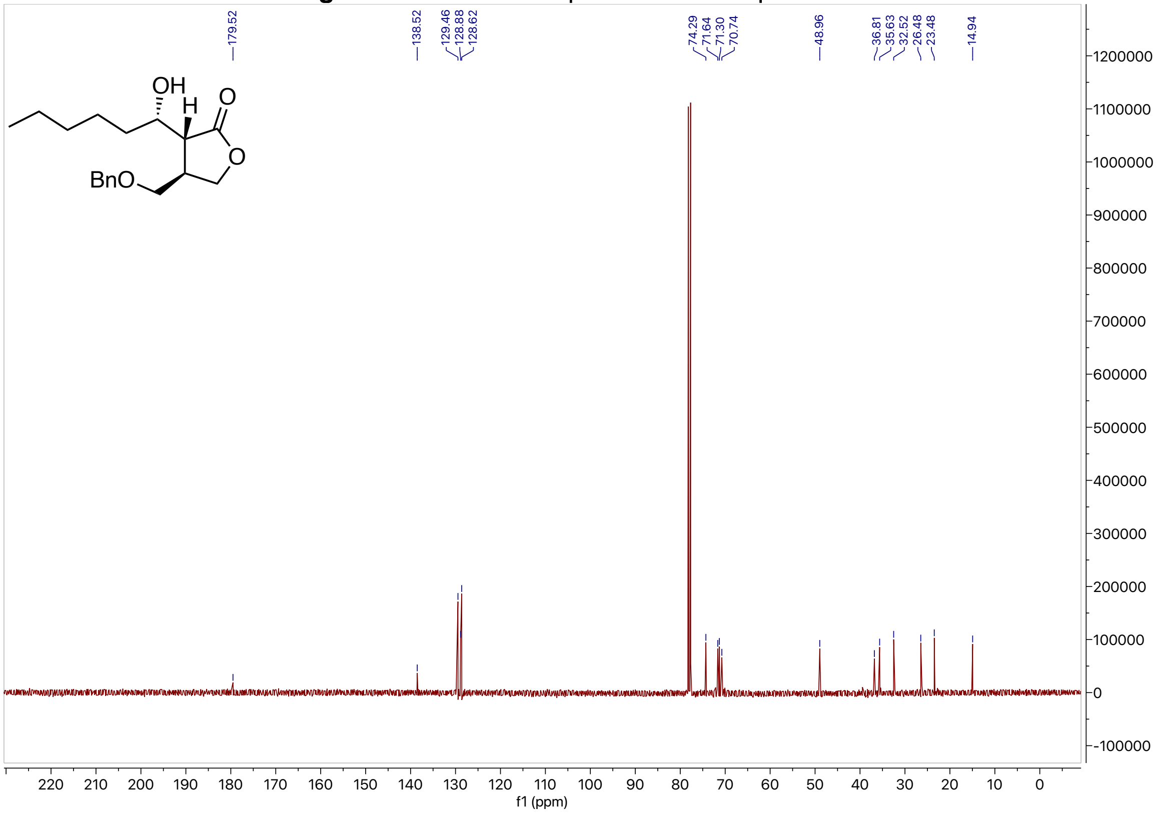

Figure S59. ^{13}C NMR Spectrum of Compound **7e**

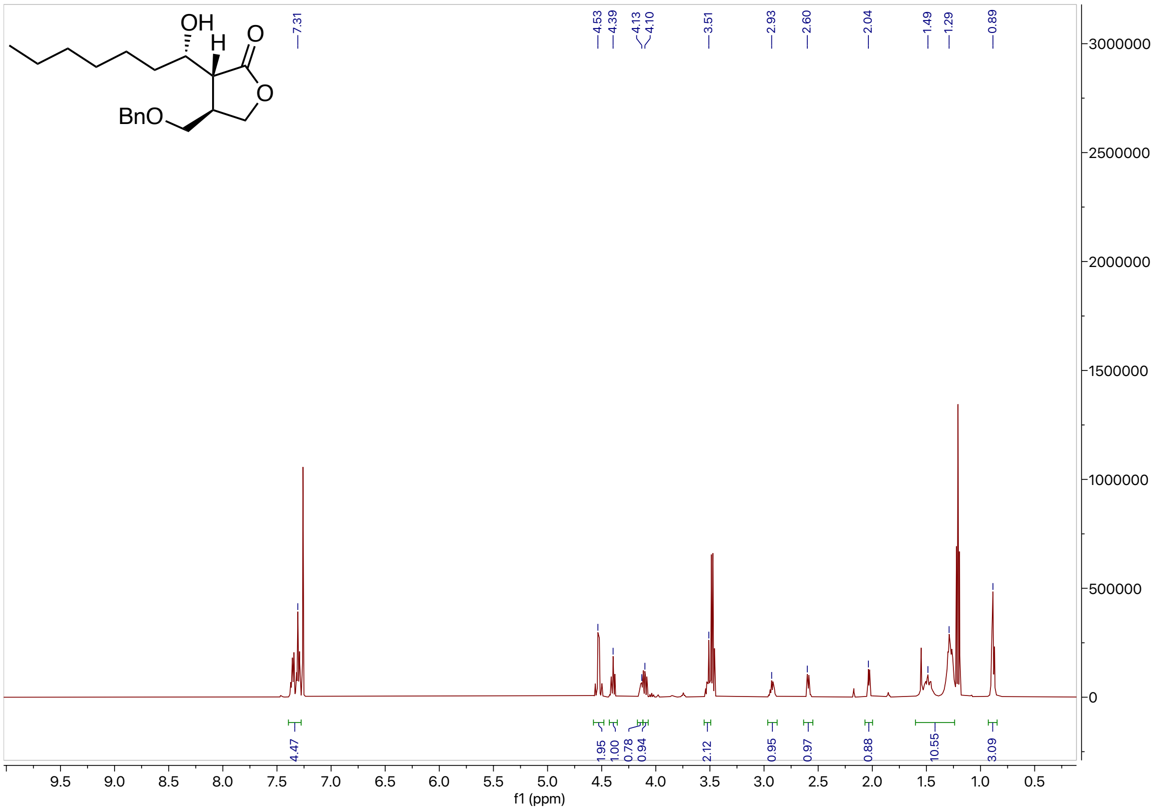

Figure S60. ^{1}H NMR Spectrum of Compound **7f**

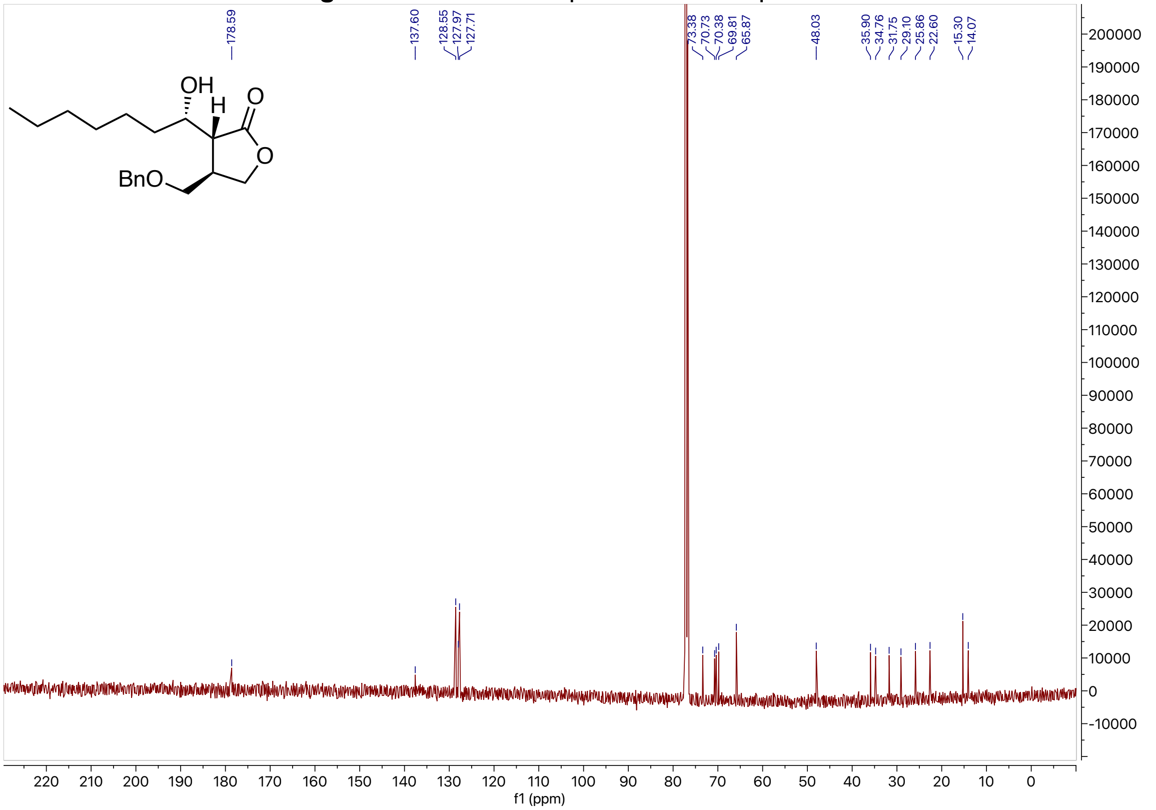

Figure S61. ^{13}C NMR Spectrum of Compound **7f**

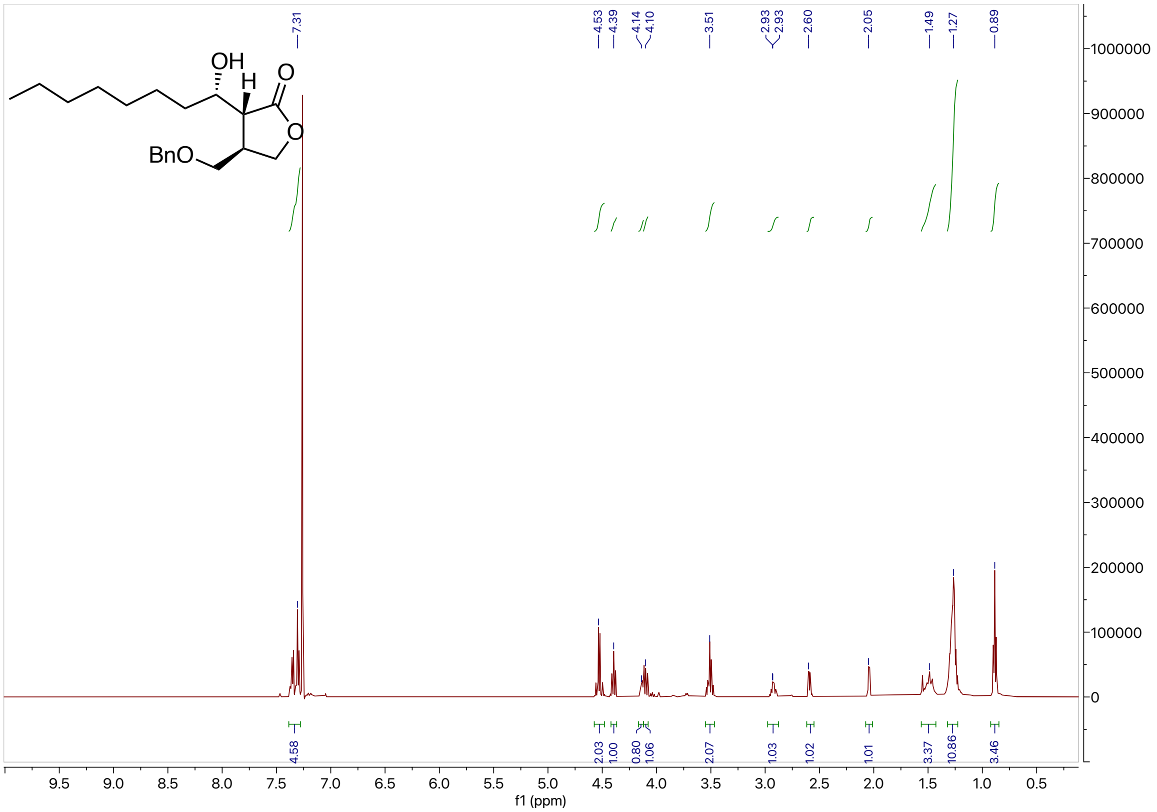

Figure S62. ^{1}H NMR Spectrum of Compound **7g**

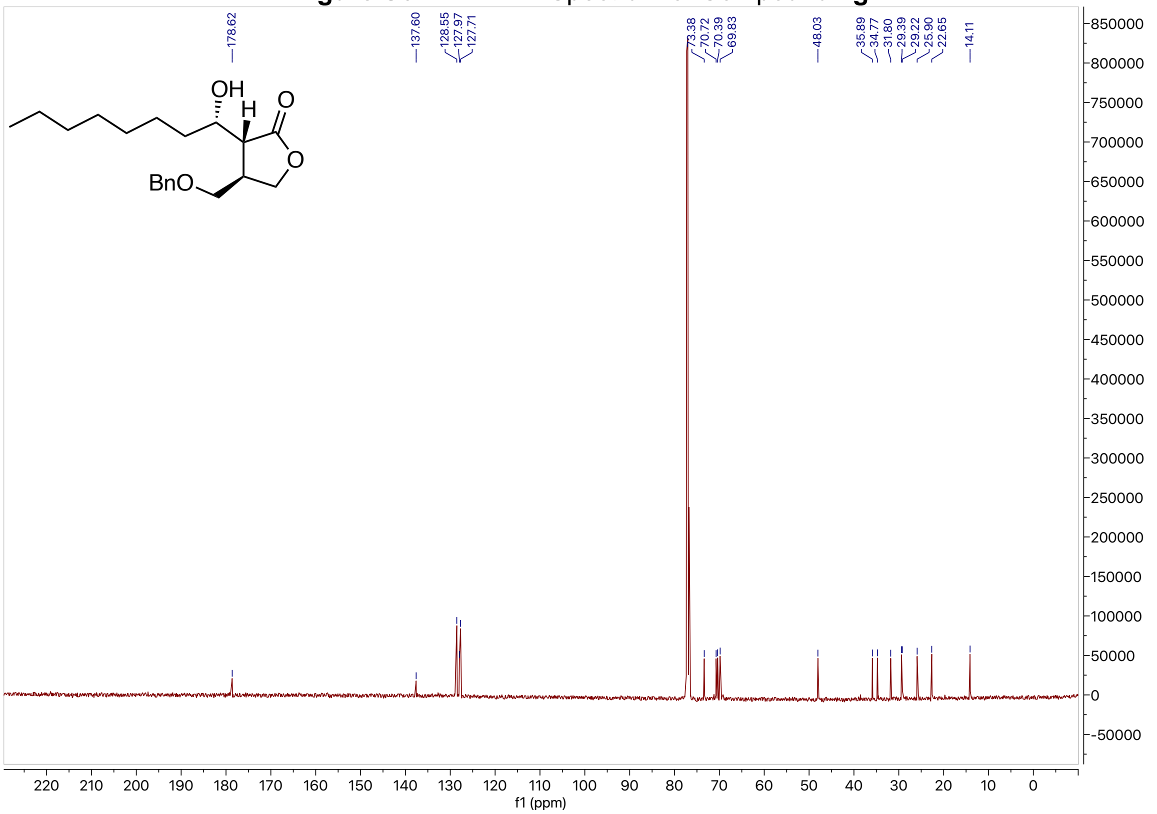

Figure S63. ^{13}C NMR Spectrum of Compound **7g**

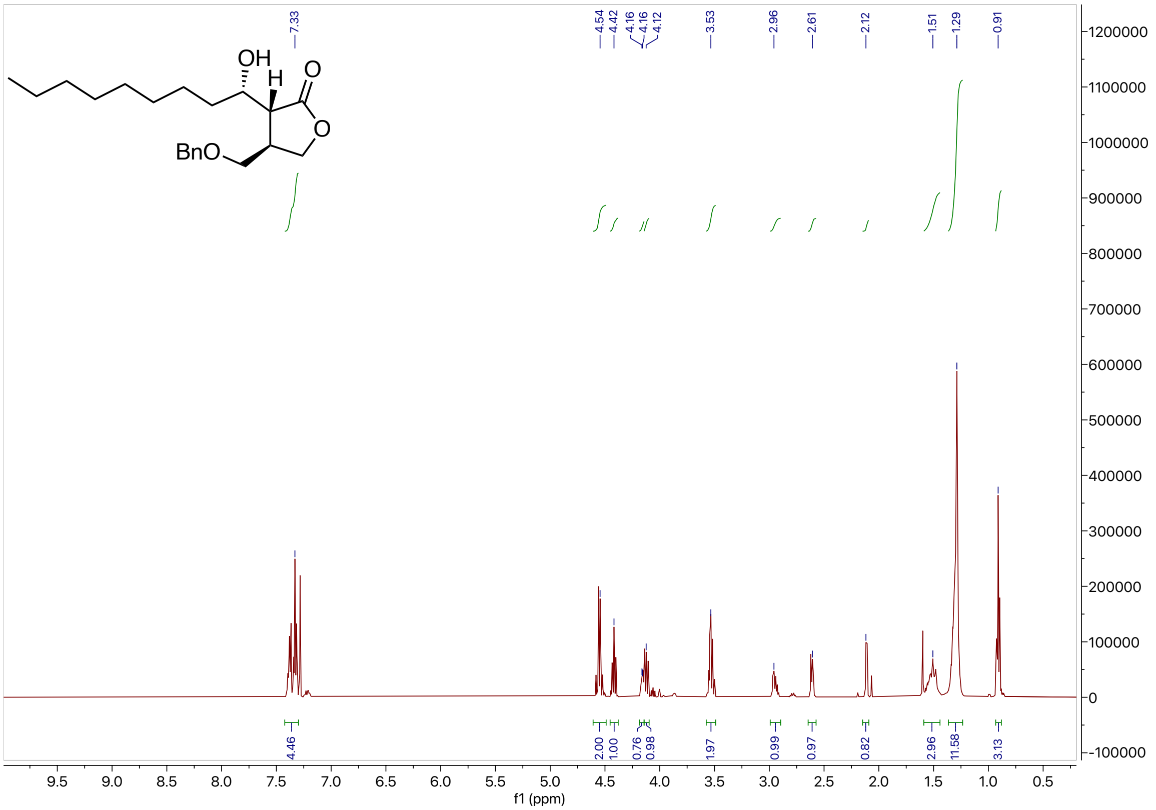

Figure S64. ^{1}H NMR Spectrum of Compound **7h**

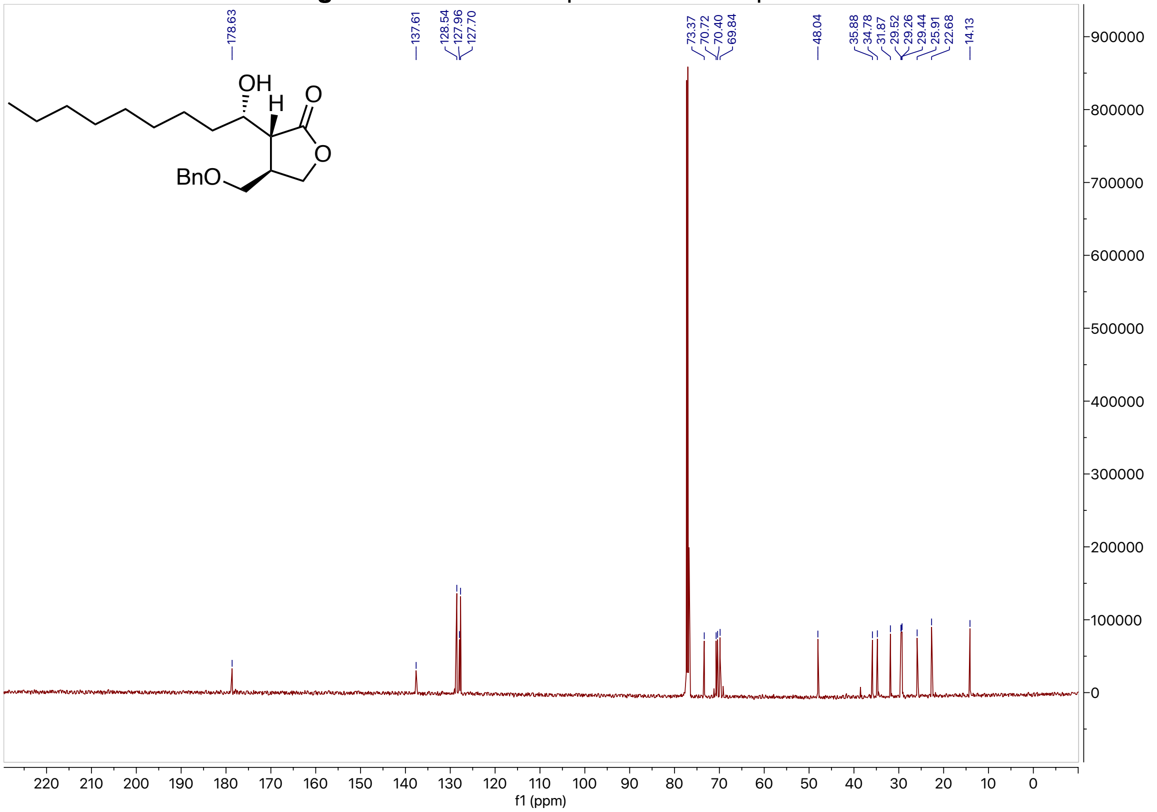

Figure S65. ^{13}C NMR Spectrum of Compound **7h**

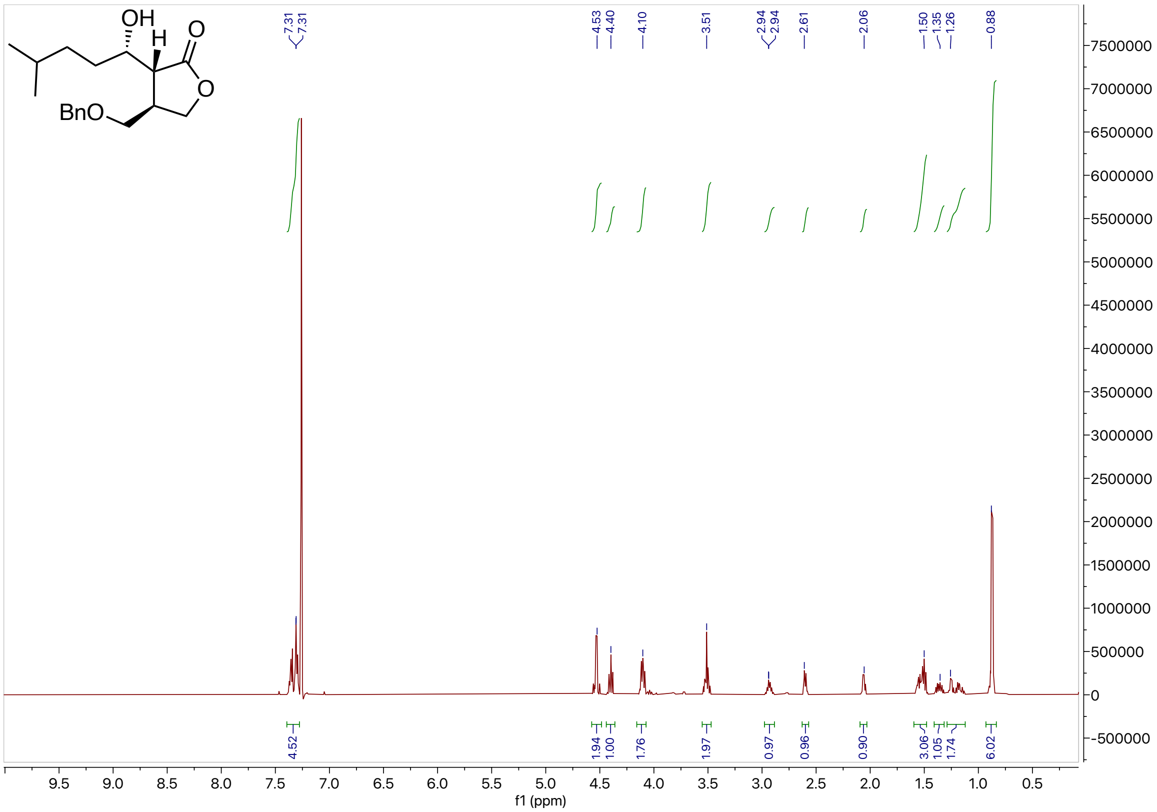

Figure S66. ^{1}H NMR Spectrum of Compound **7i**

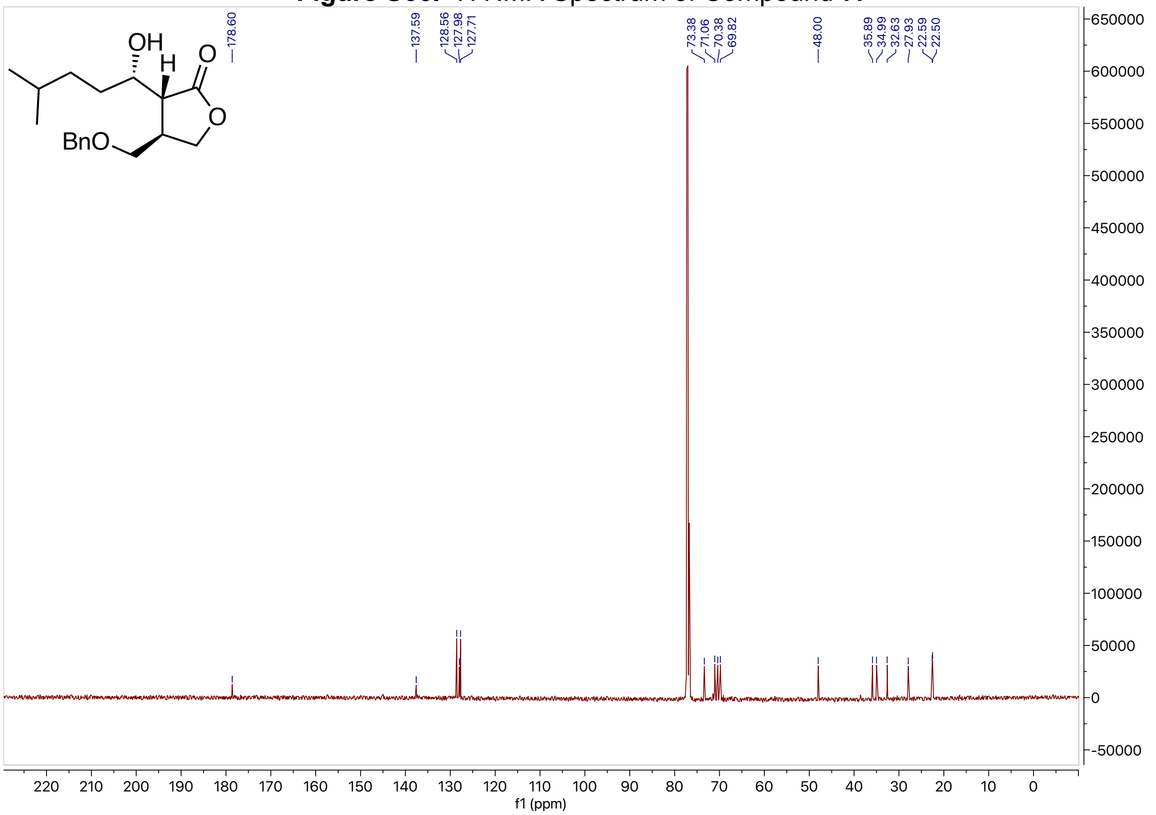

Figure S67. ^{13}C NMR Spectrum of Compound **7i**

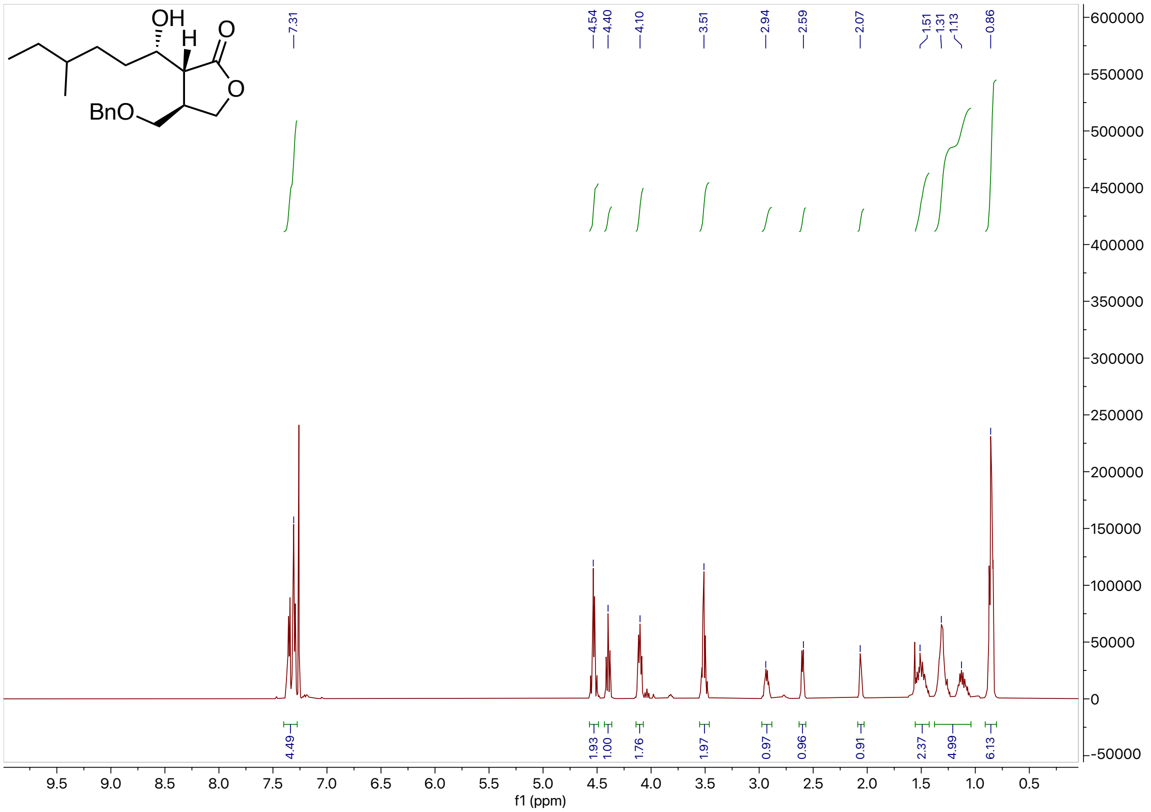

Figure S68. ^{1}H NMR Spectrum of Compound **7j**

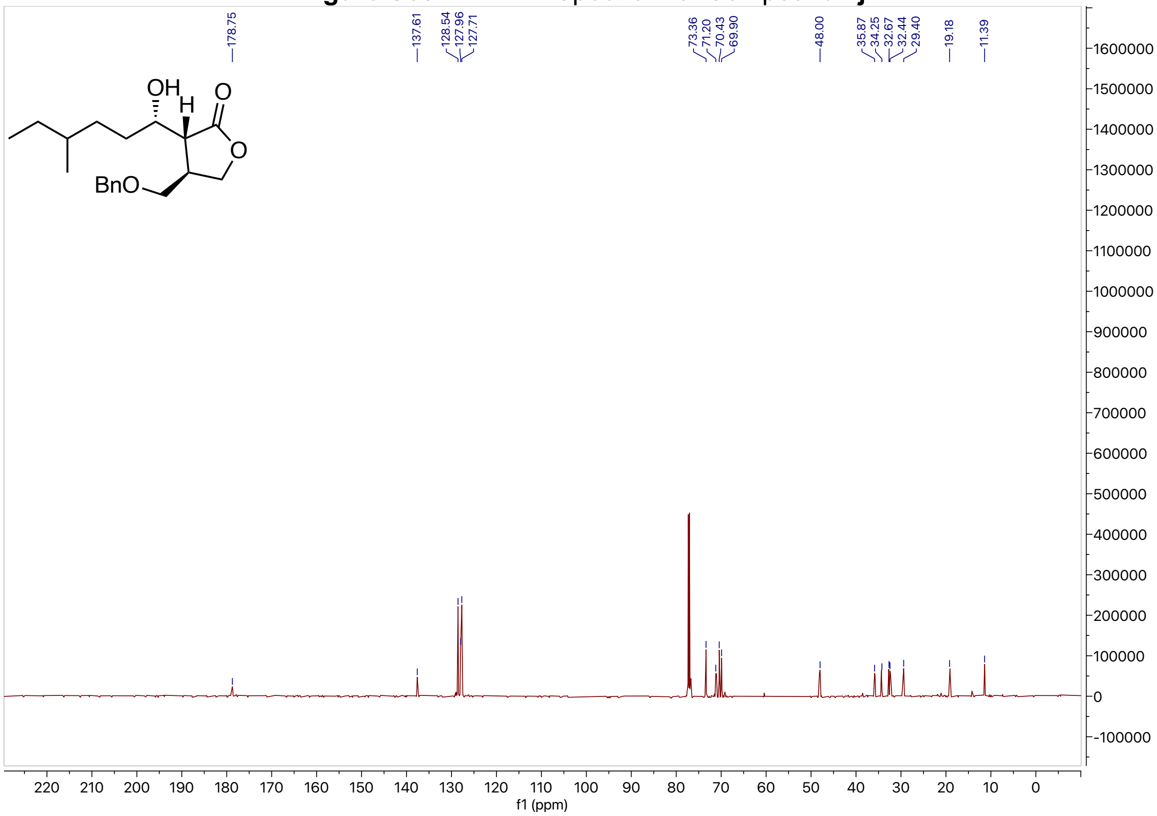

Figure S69. ^{13}C NMR Spectrum of Compound **7j**

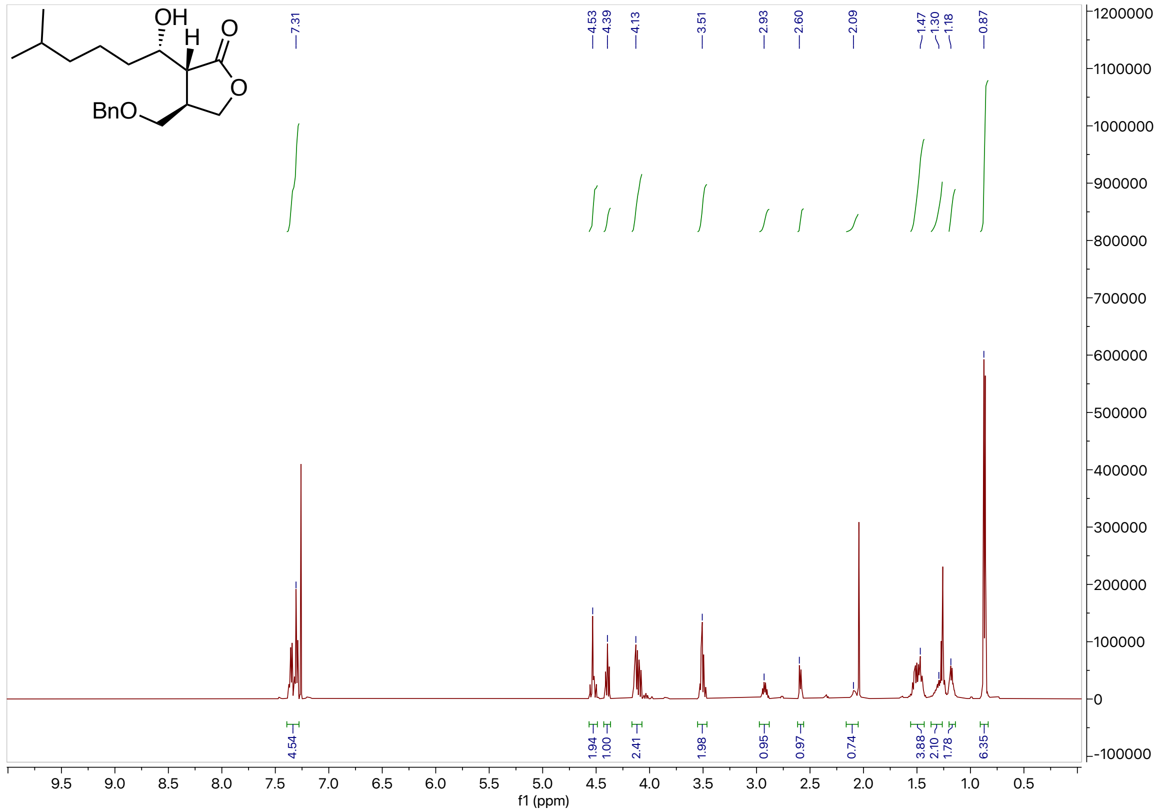

Figure S70. ^{1}H NMR Spectrum of Compound **7k**

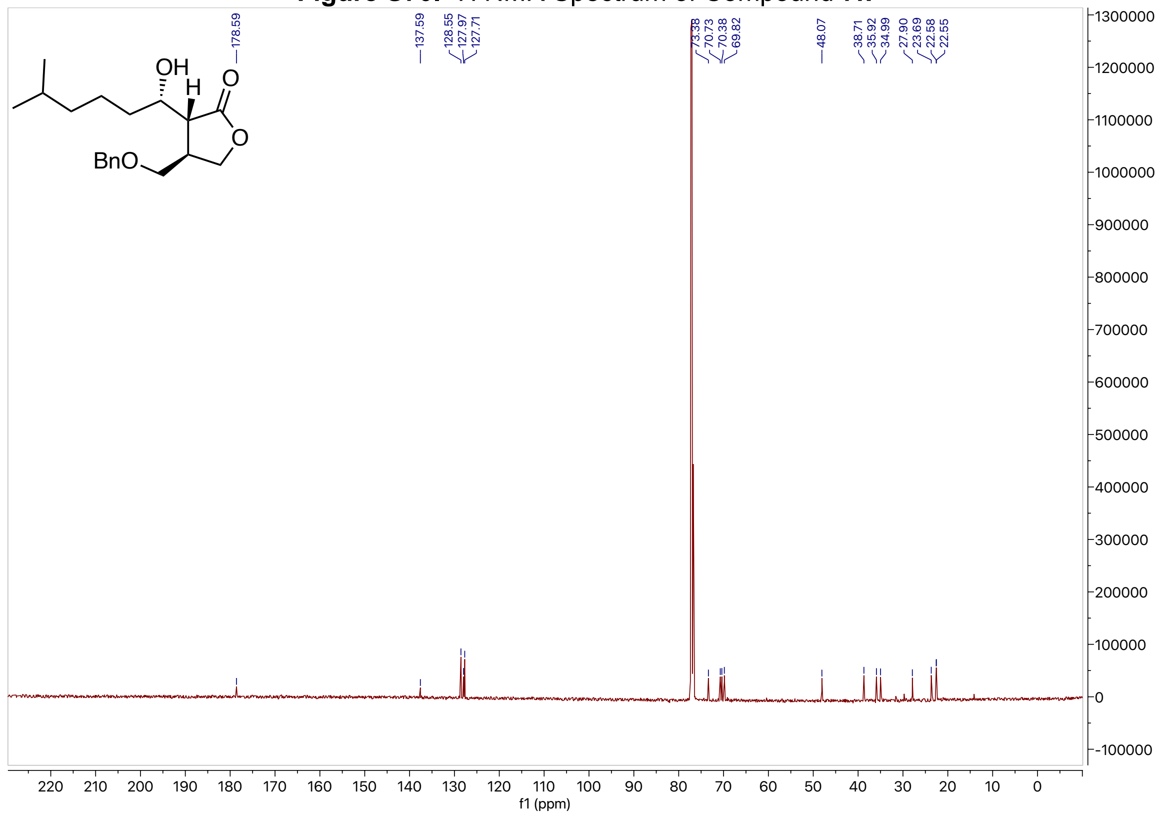

Figure S71. ^{13}C NMR Spectrum of Compound **7k**

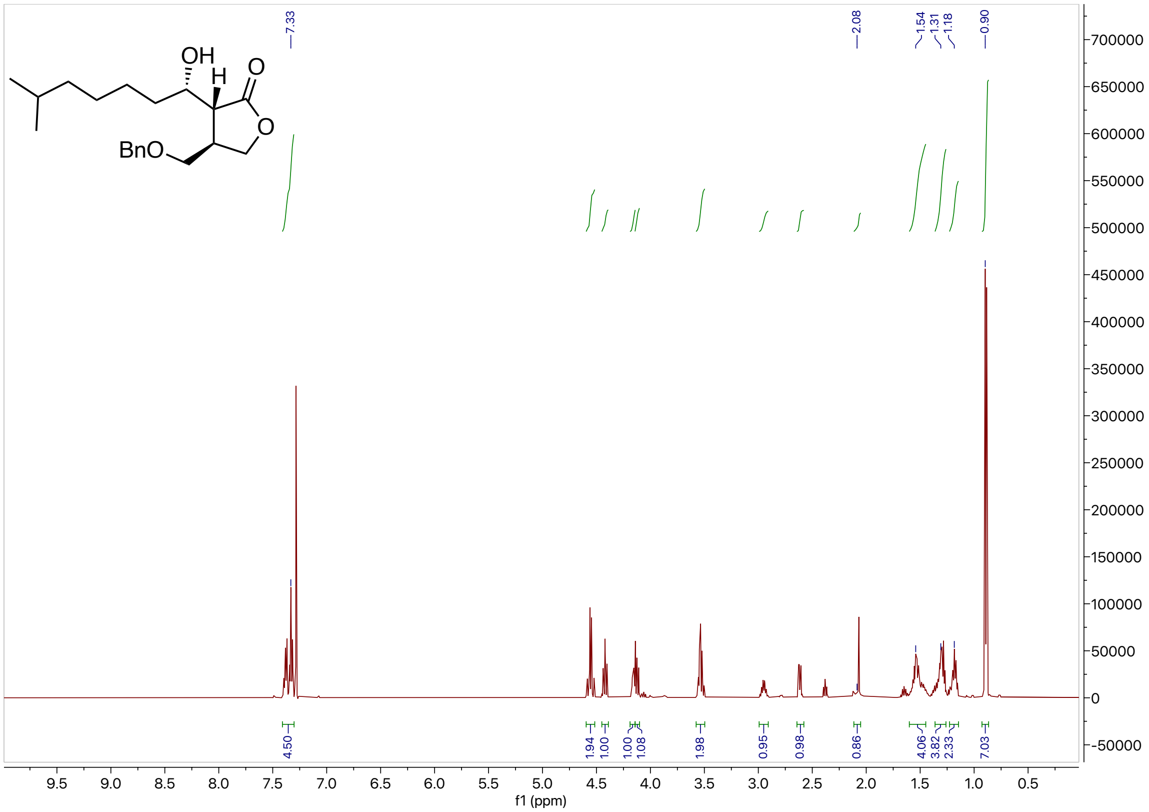

Figure S72. ^{1}H NMR Spectrum of Compound **7l**

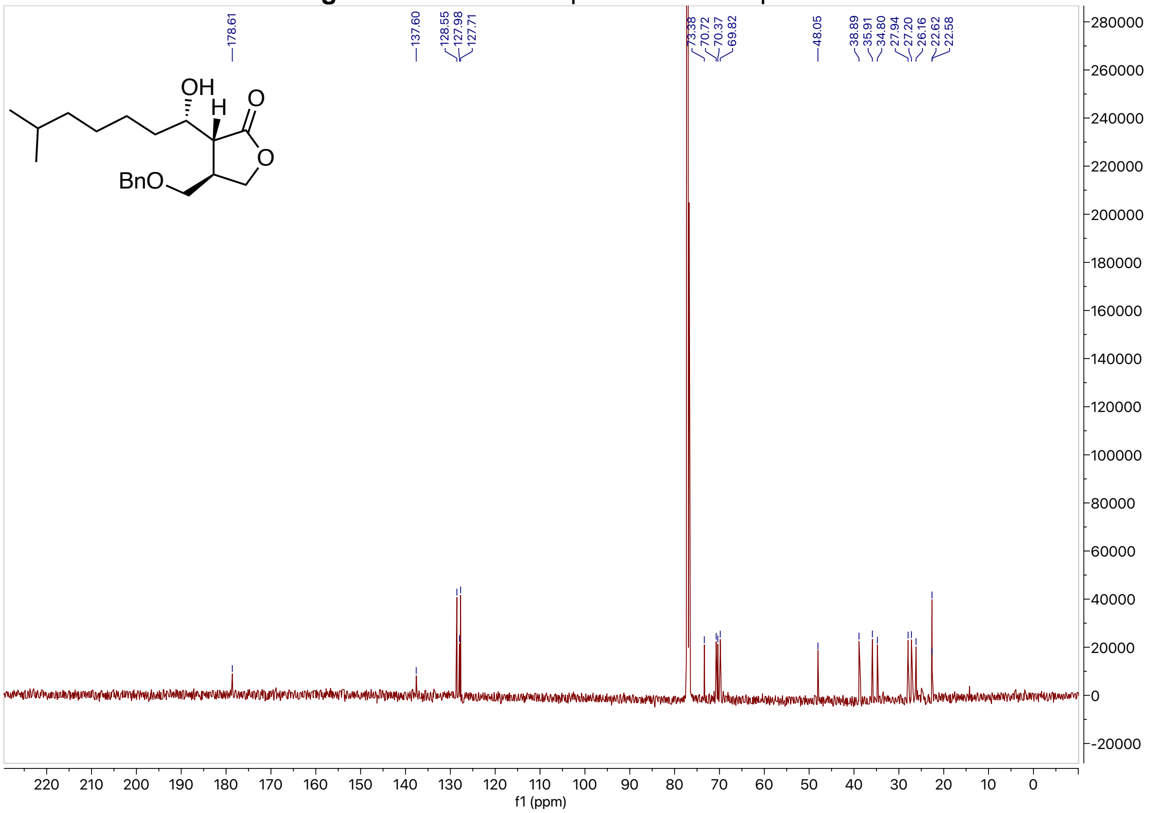

Figure S73. ^{13}C NMR Spectrum of Compound **7l**

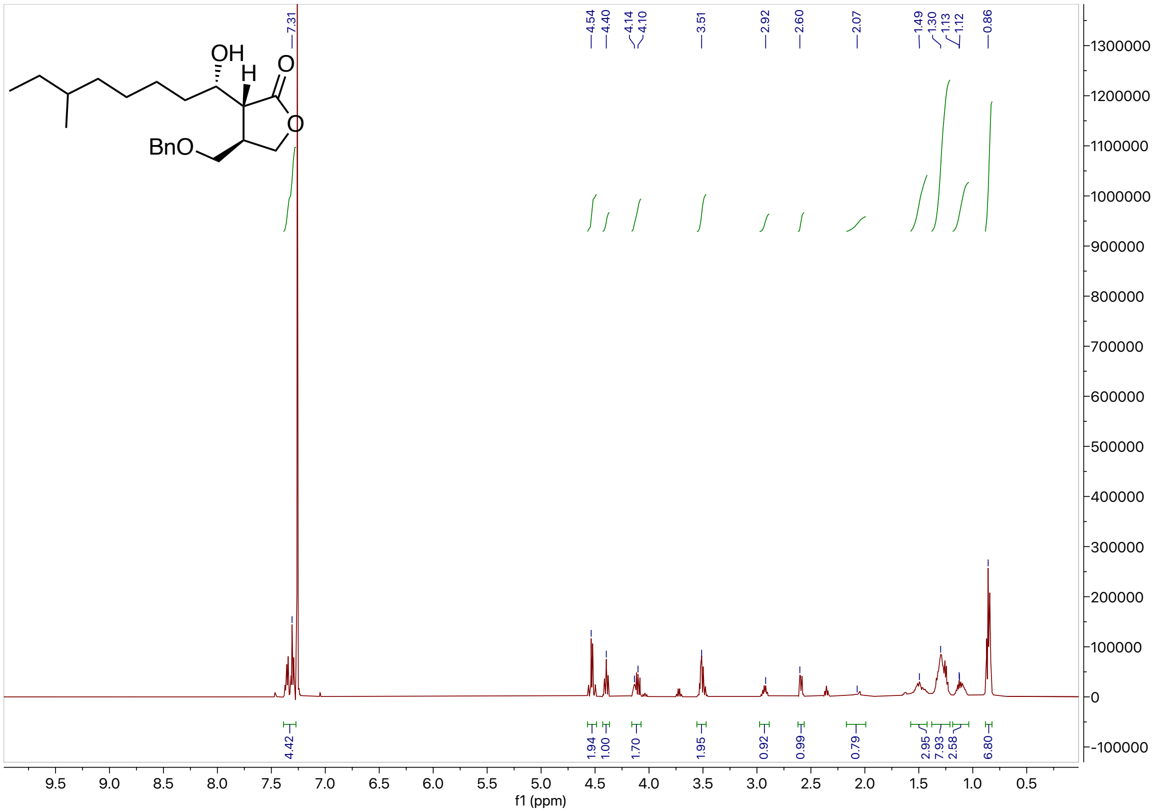

Figure S74. ^{1}H NMR Spectrum of Compound **7m**

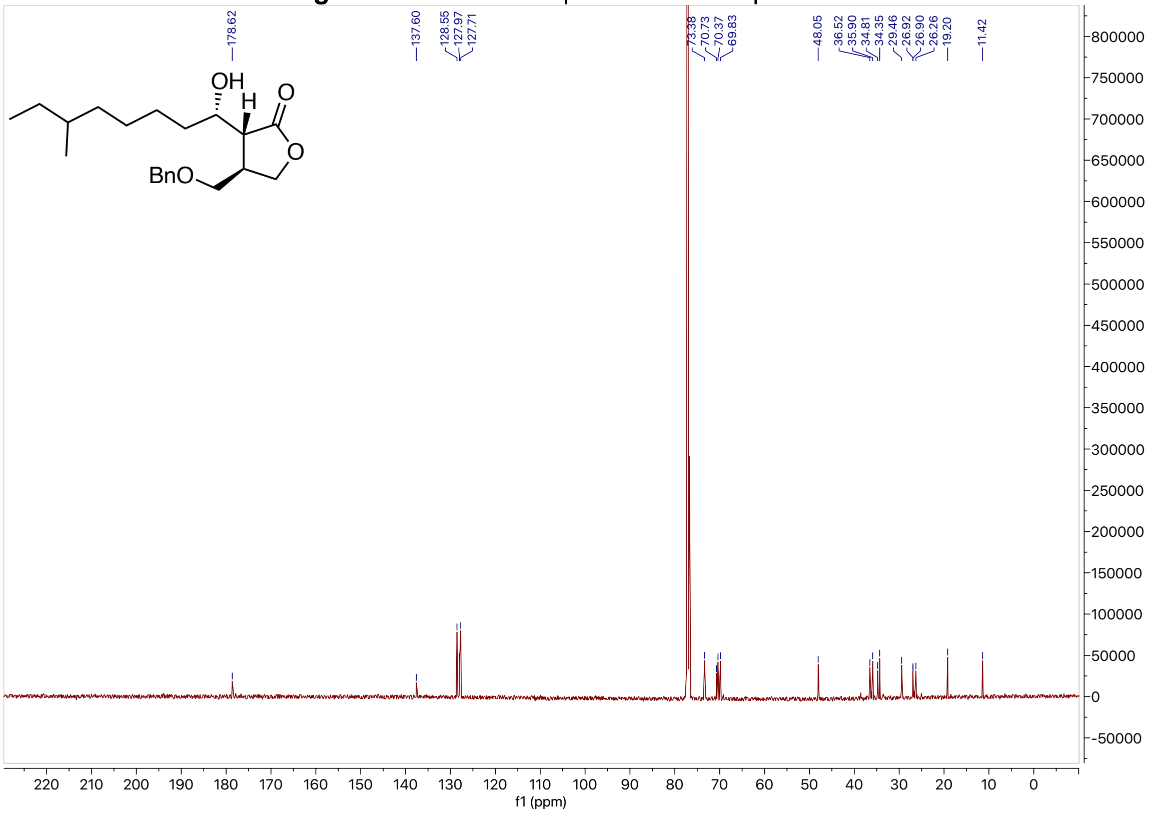

Figure S75. ^{13}C NMR Spectrum of Compound **7m**

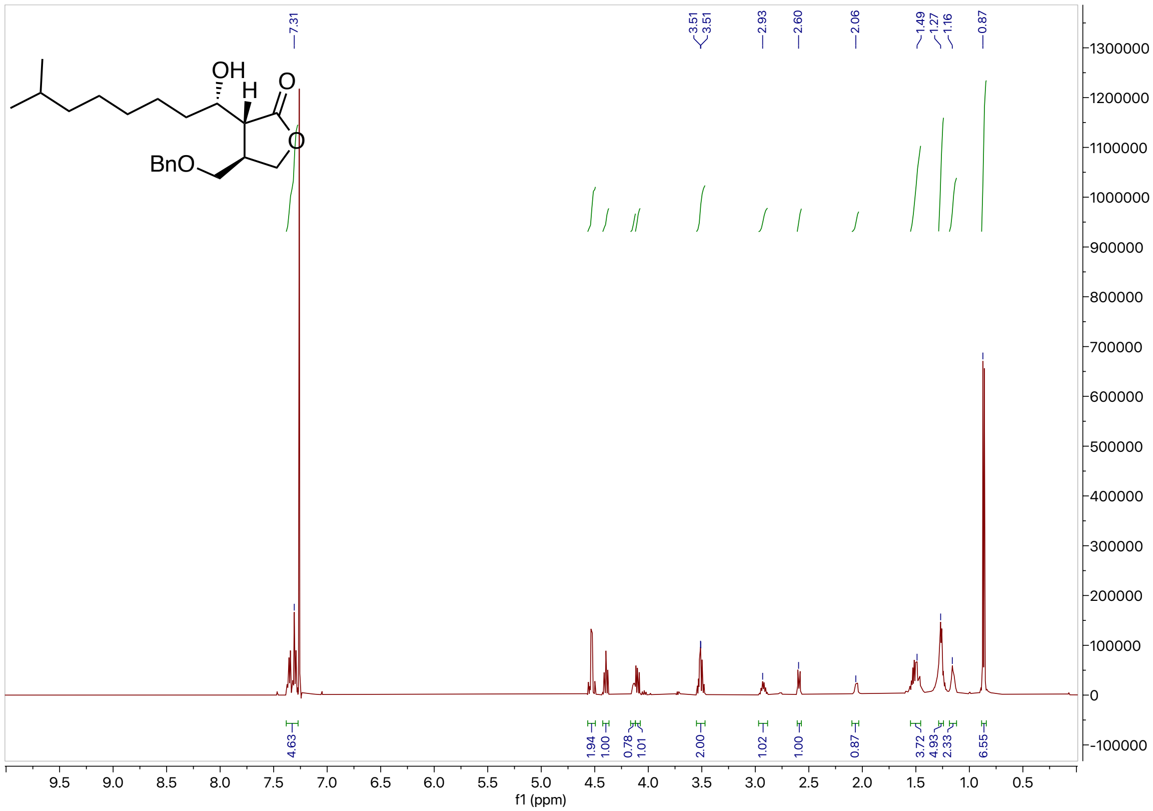

Figure S76. ^{1}H NMR Spectrum of Compound **7n**

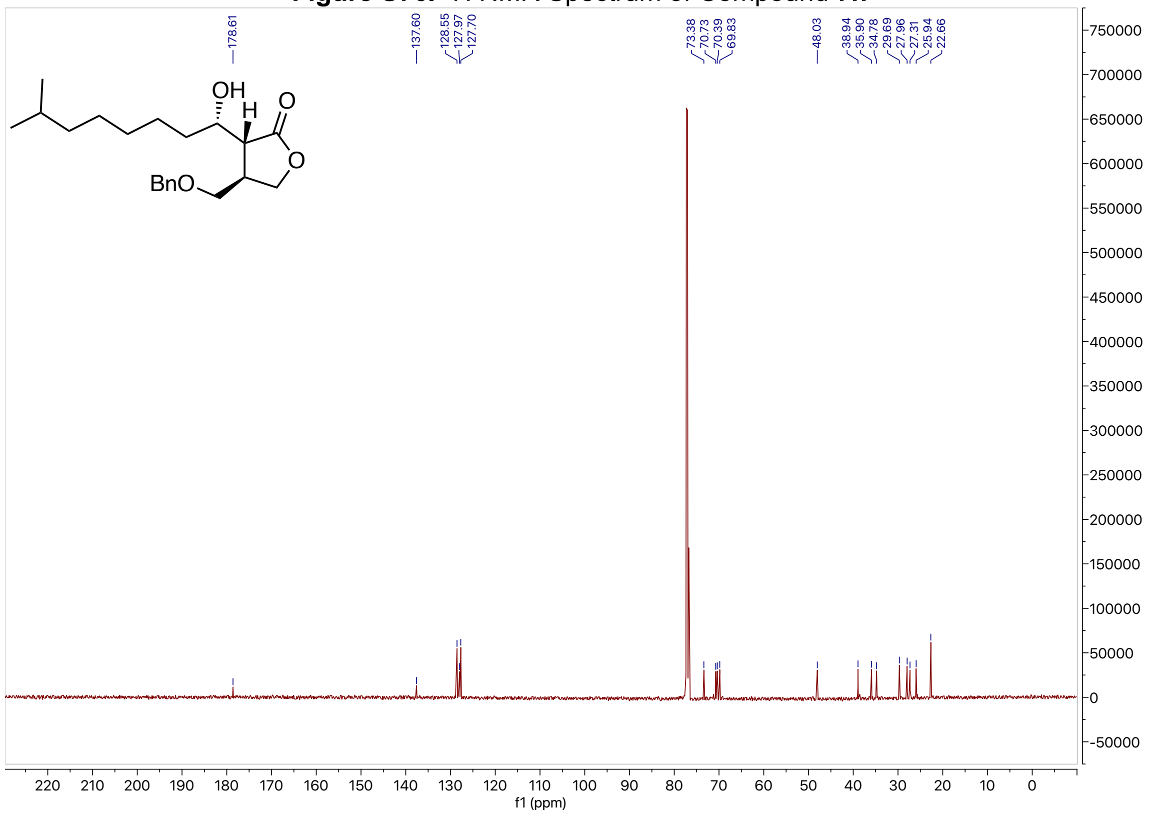

Figure S77. ^{13}C NMR Spectrum of Compound **7n**

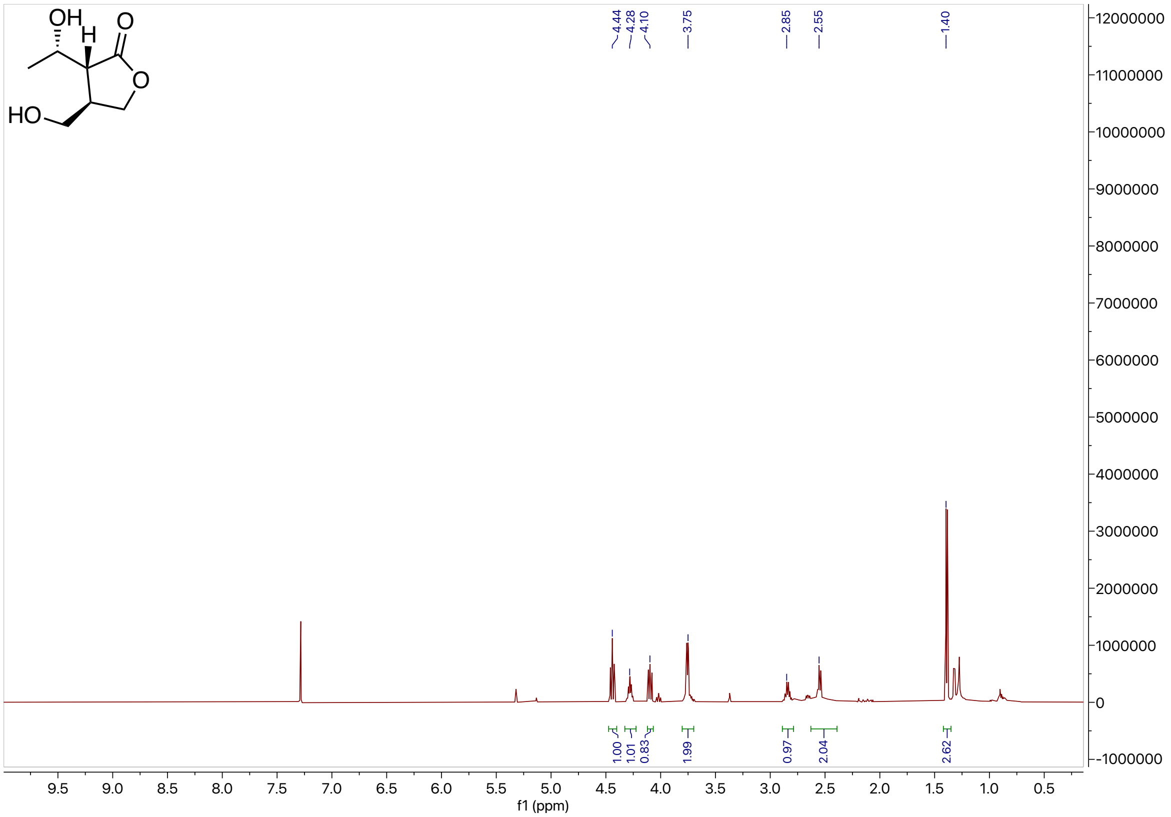

Figure S78. ^{1}H NMR Spectrum of Compound **8a**

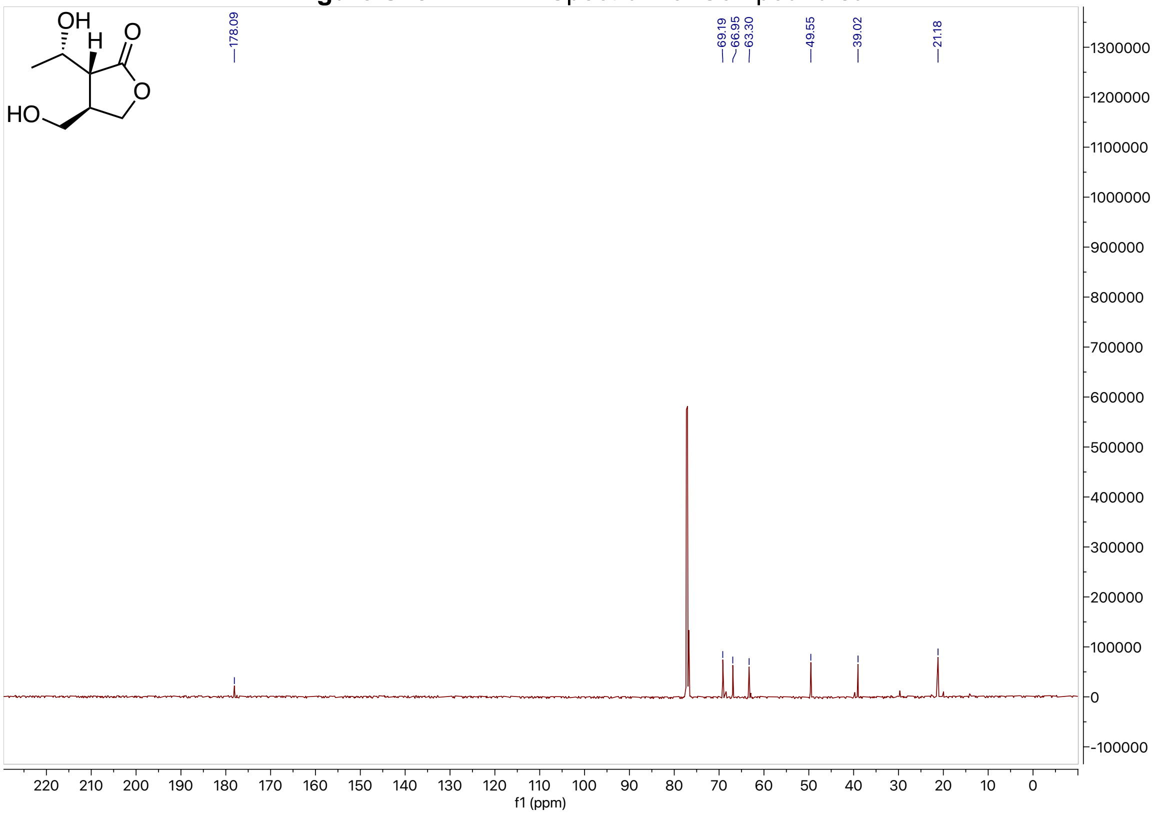

Figure S79. ^{13}C NMR Spectrum of Compound **8a**

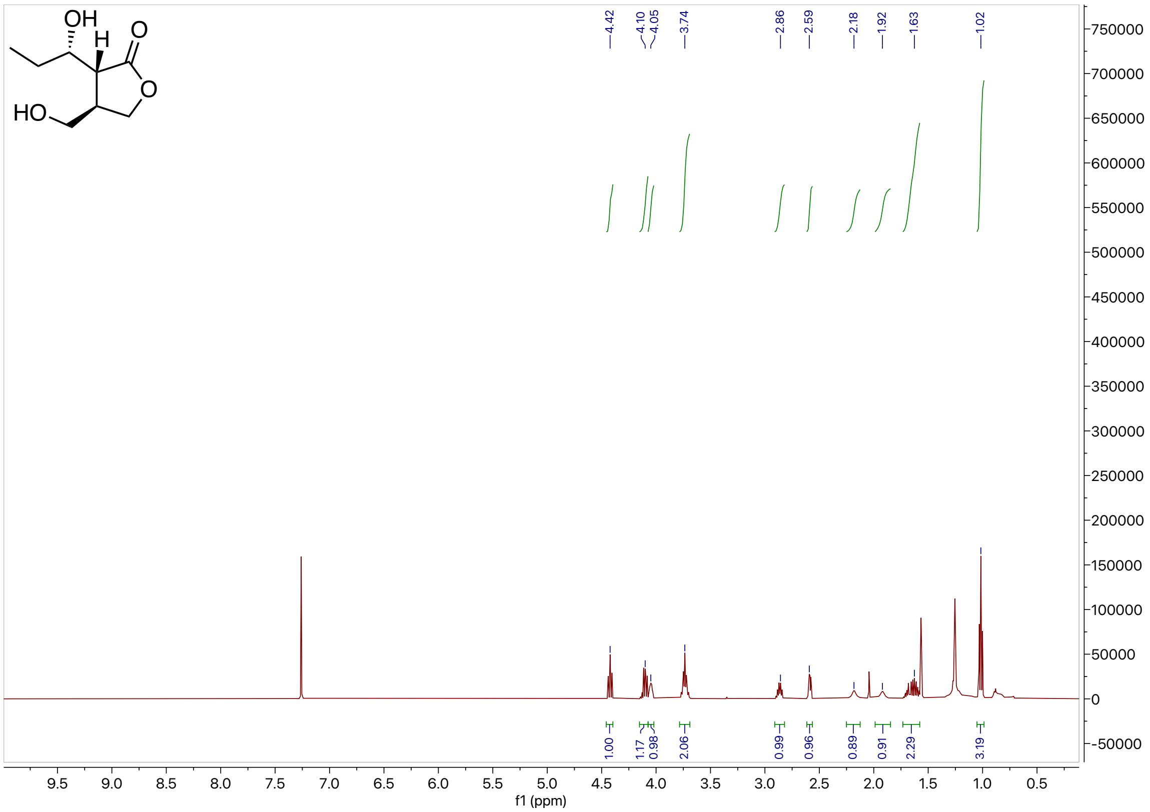

Figure S80. ^{1}H NMR Spectrum of Compound **8b**

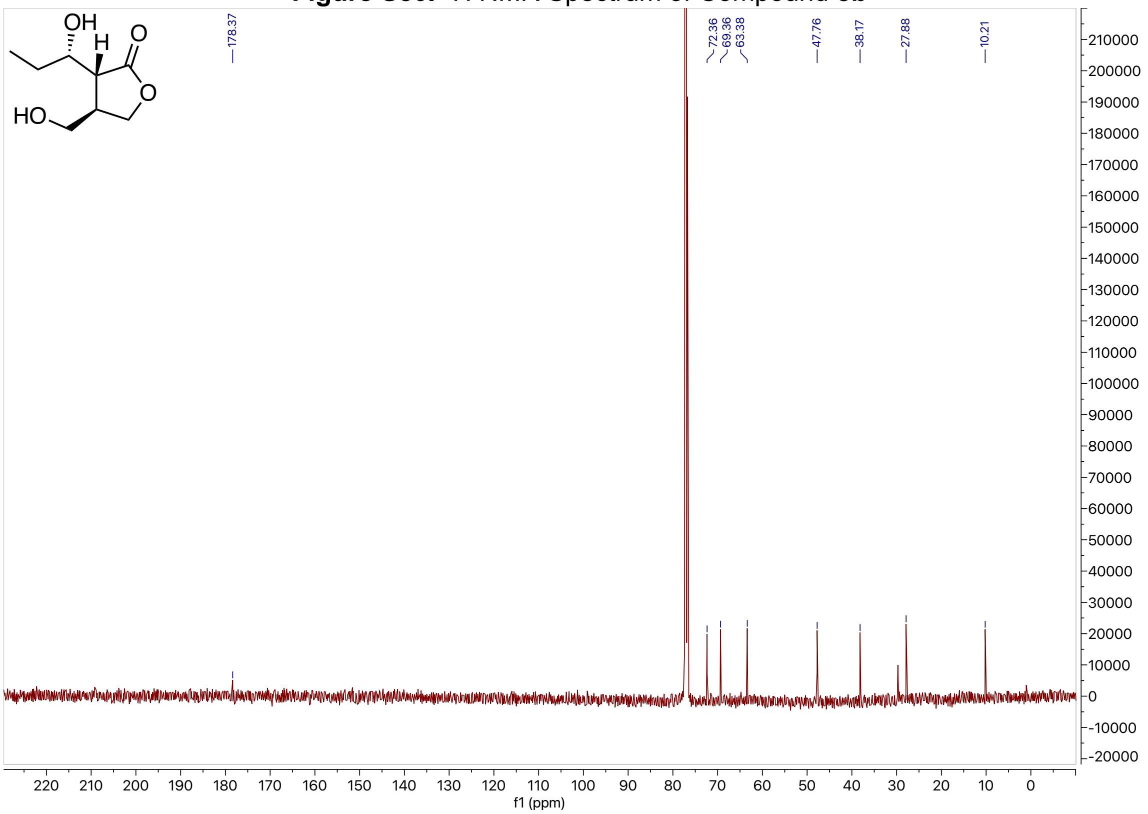

Figure S81. ^{13}C NMR Spectrum of Compound **8b**

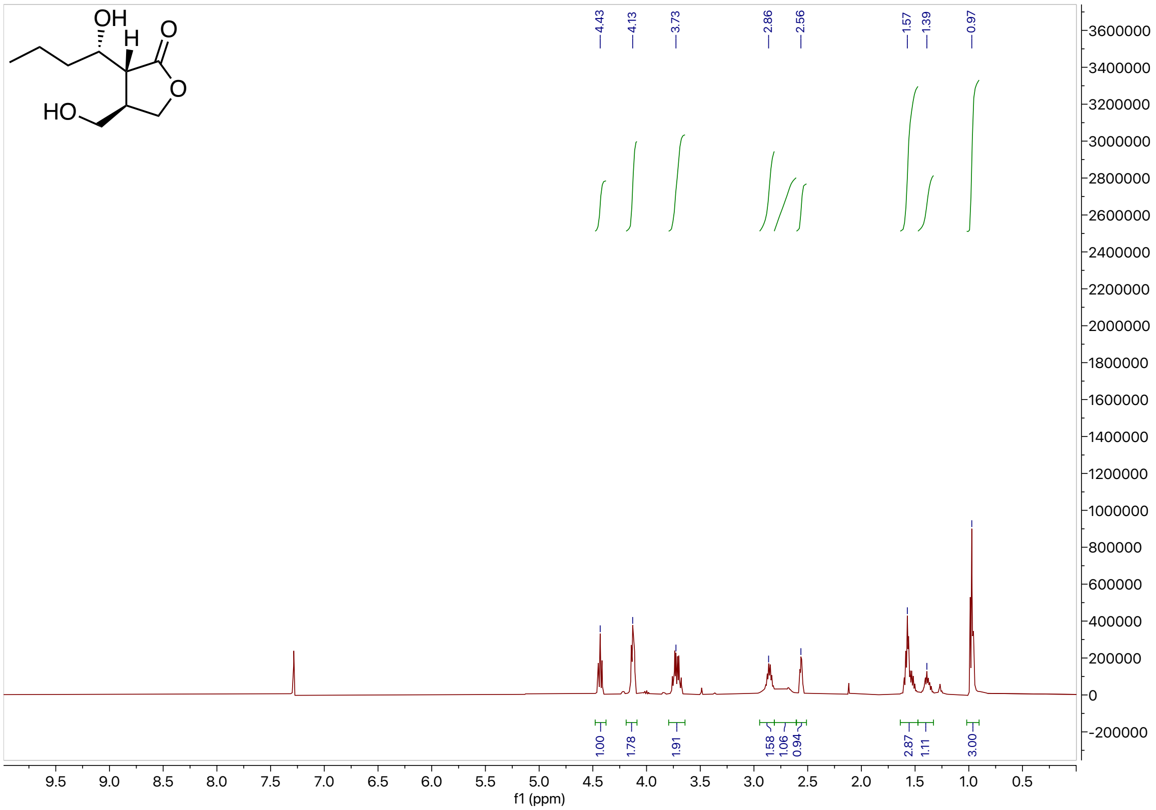

Figure S82. ^{1}H NMR Spectrum of Compound **8c**

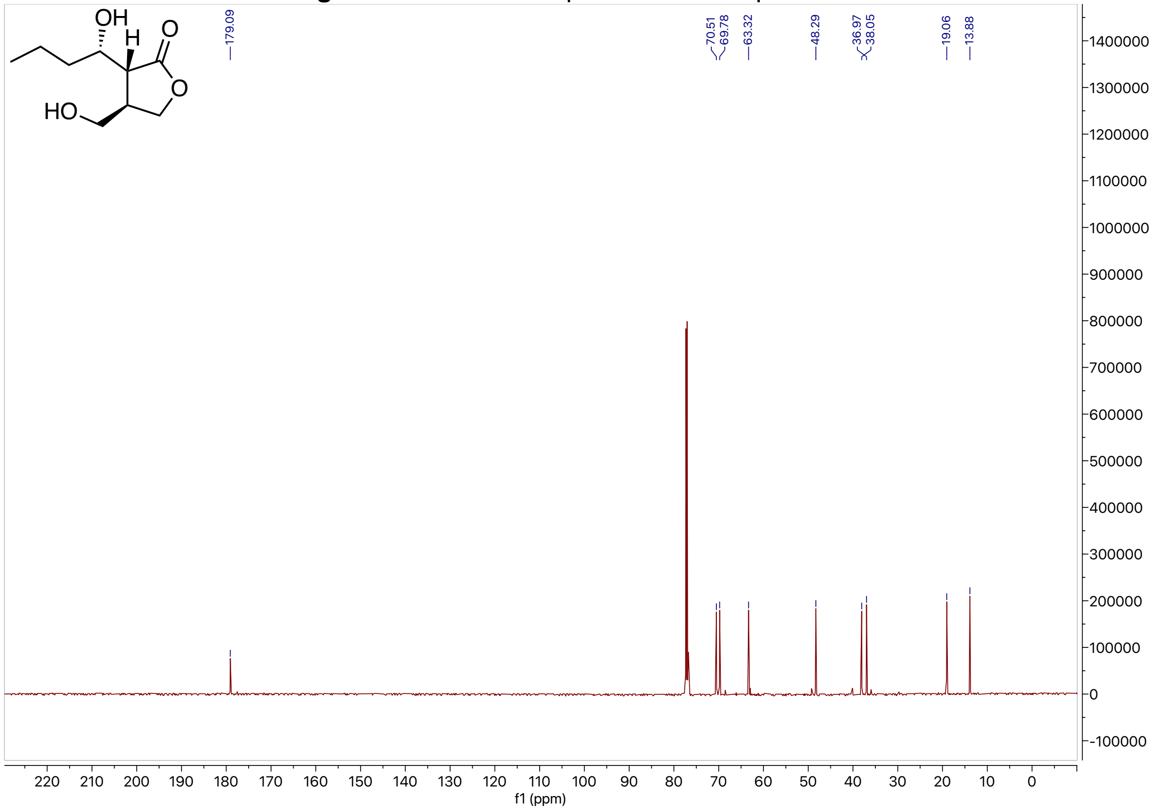

Figure S83. ^{13}C NMR Spectrum of Compound **8c**

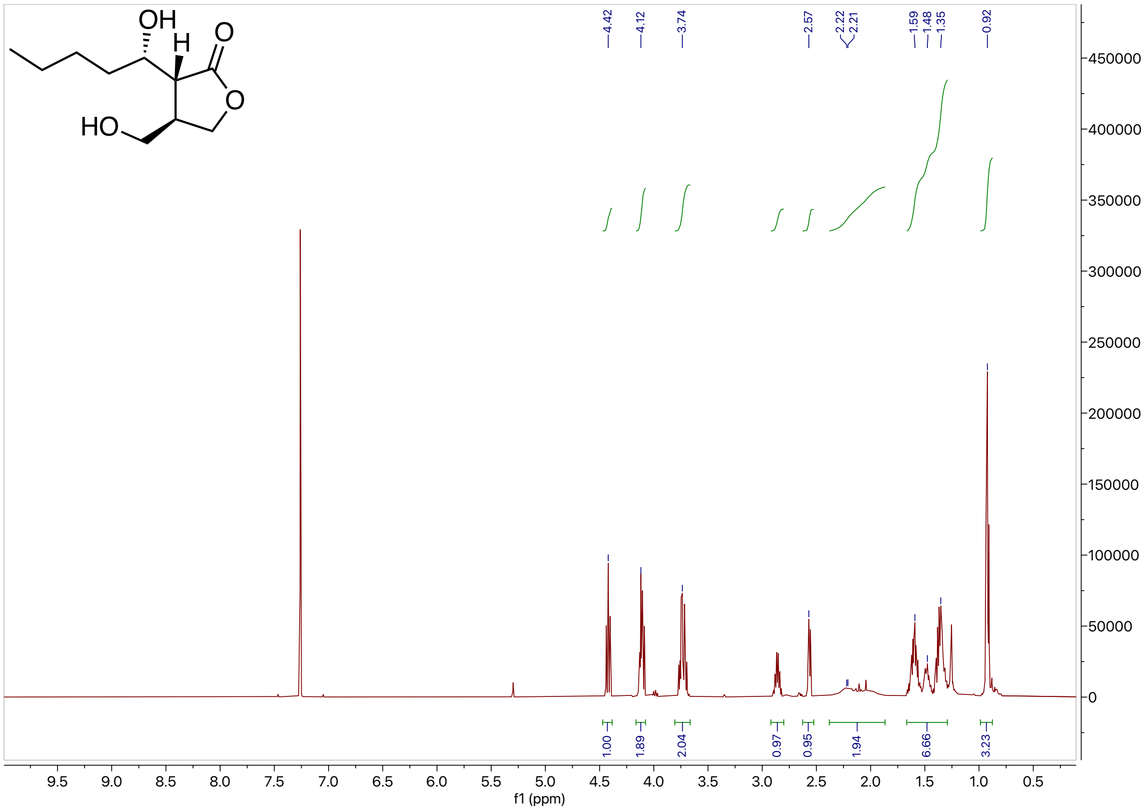

Figure S84. ^{1}H NMR Spectrum of Compound **8d**

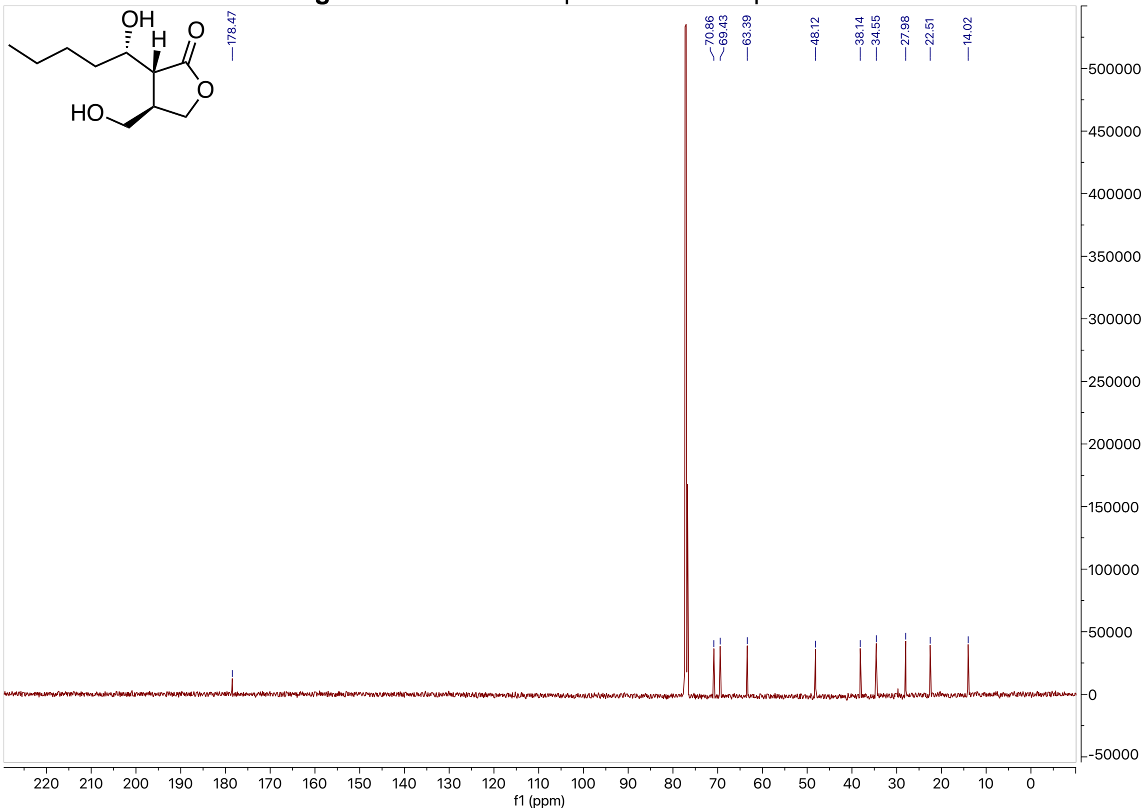

Figure S85. ^{13}C NMR Spectrum of Compound **8d**

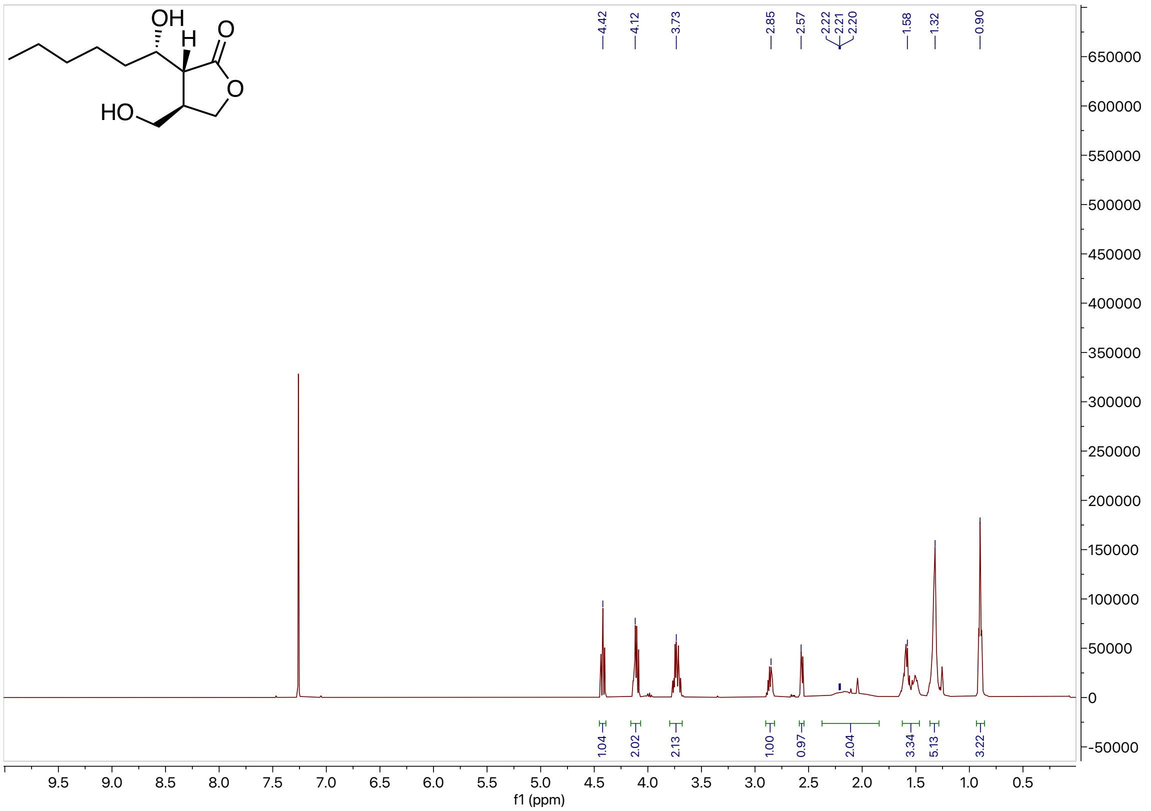

Figure S86. ^{1}H NMR Spectrum of Compound **8e**

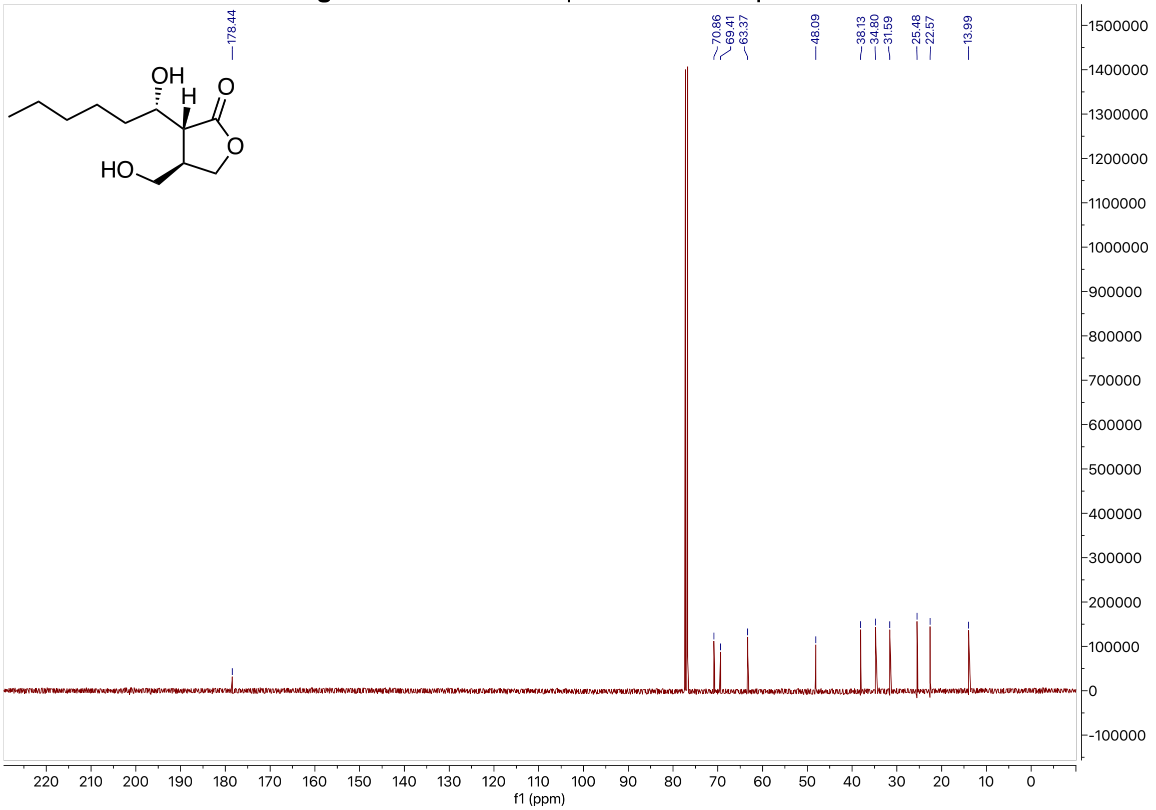

Figure S87. ^{13}C NMR Spectrum of Compound **8e**

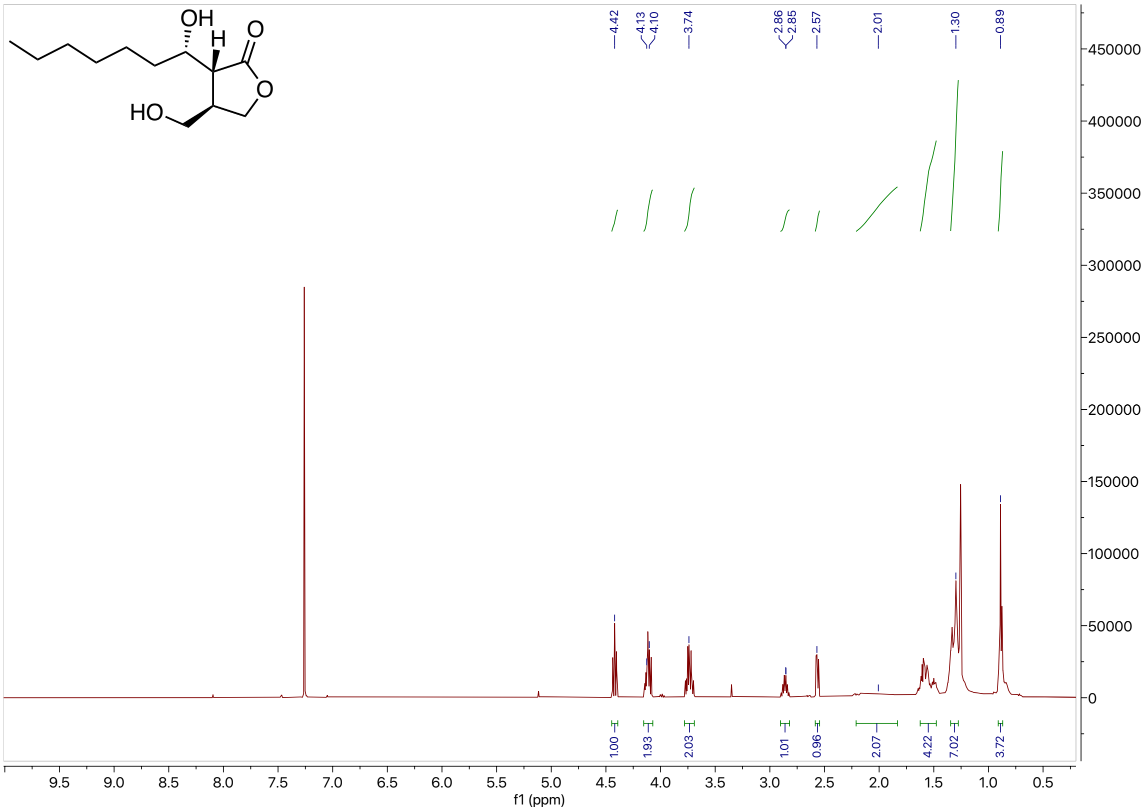

Figure S88. ^{1}H NMR Spectrum of Compound **8f**

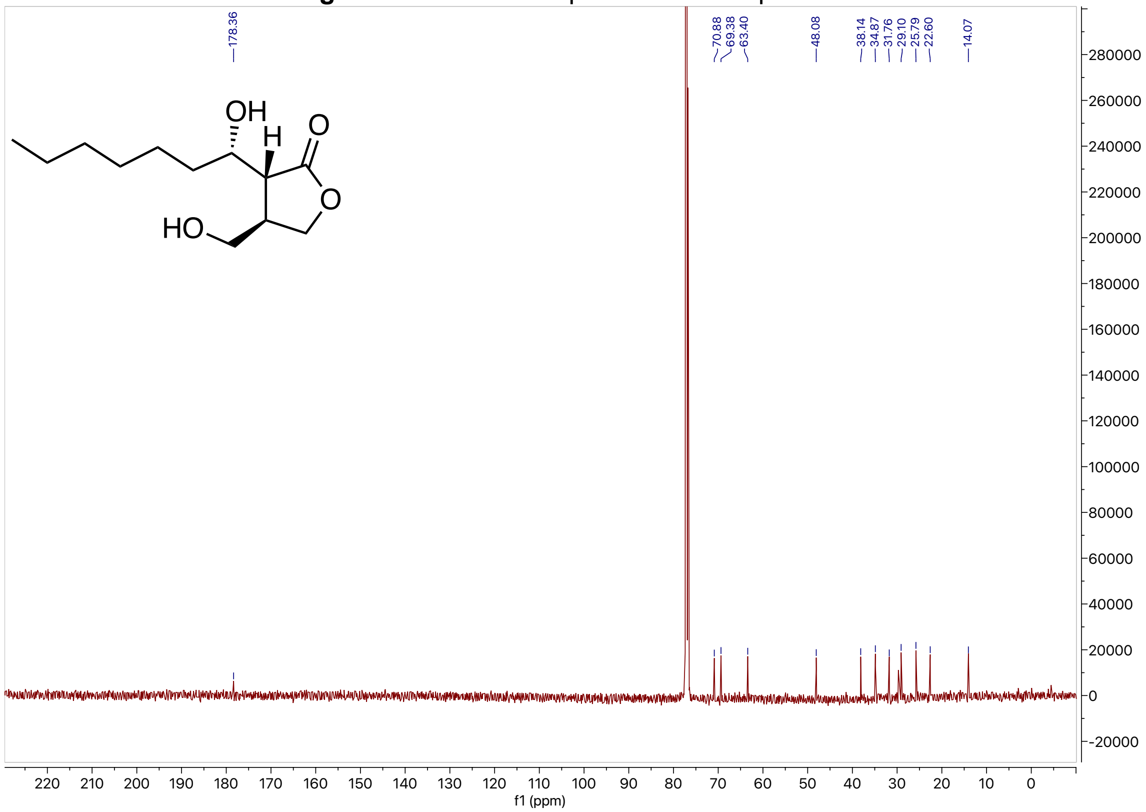

Figure S89. ^{13}C NMR Spectrum of Compound **8f**

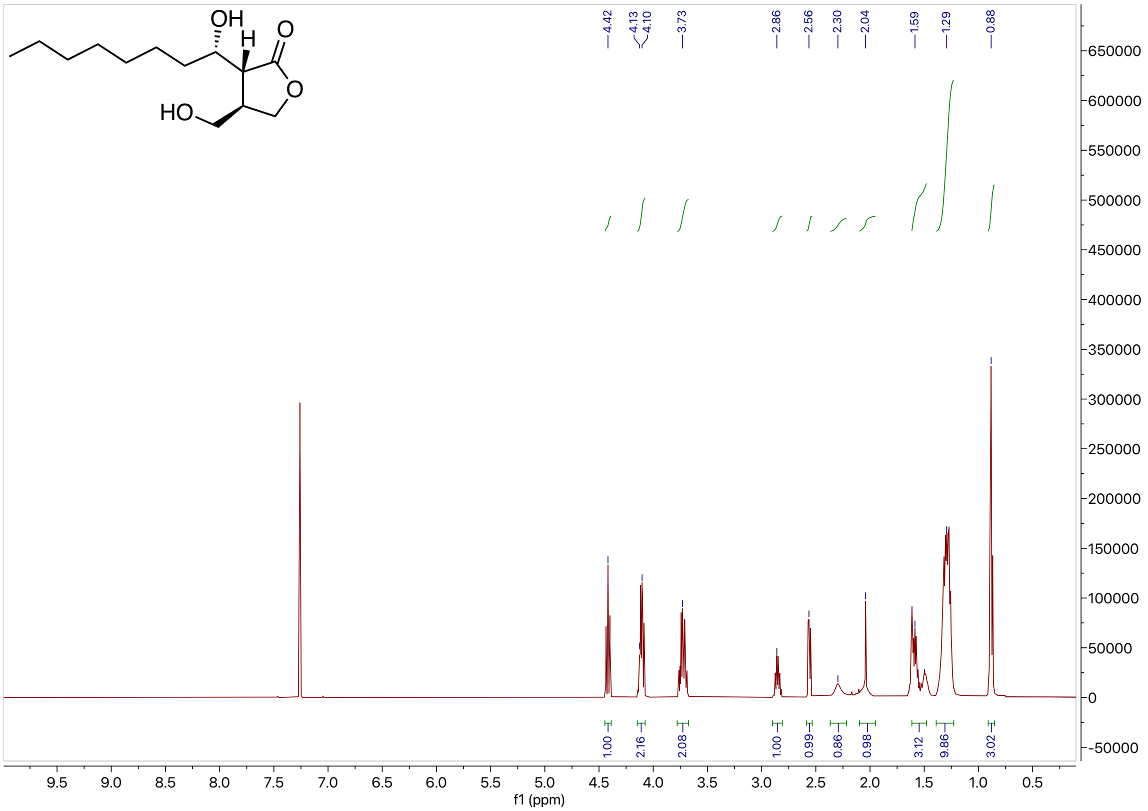

Figure S90. ^{1}H NMR Spectrum of Compound **8g**

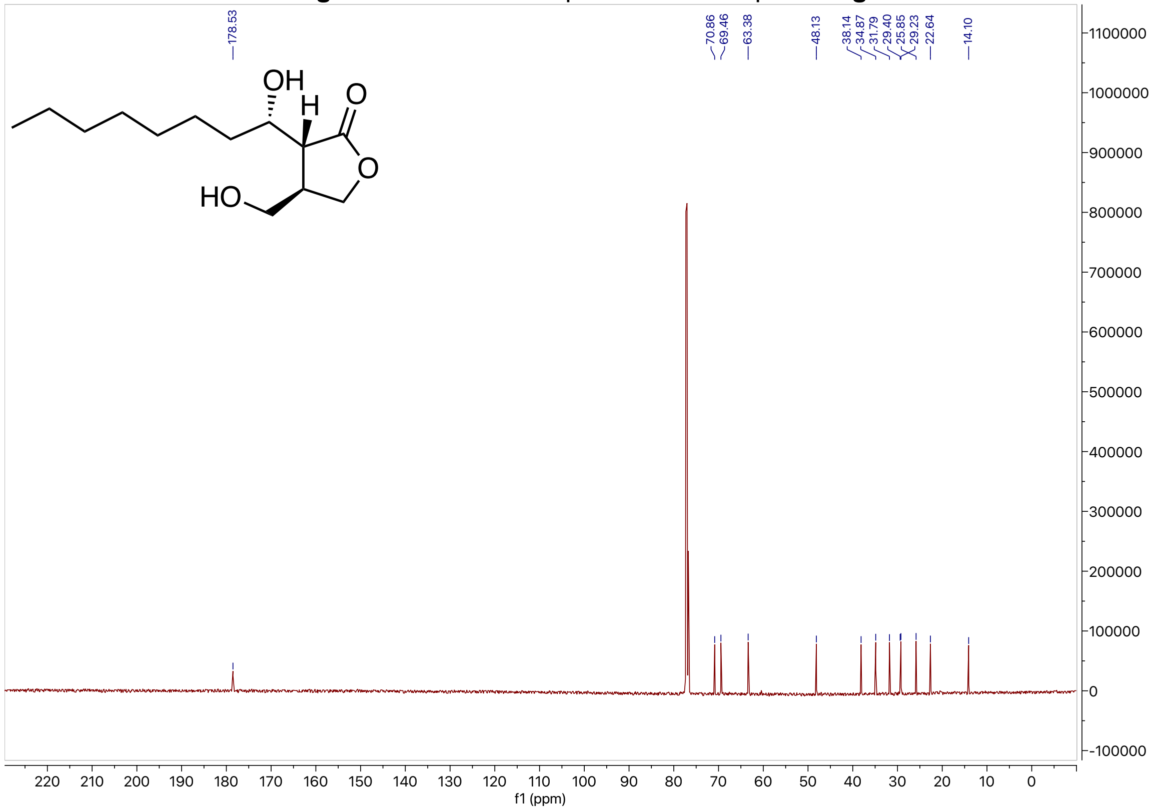

Figure 91. ^{13}C NMR Spectrum of Compound **8g**

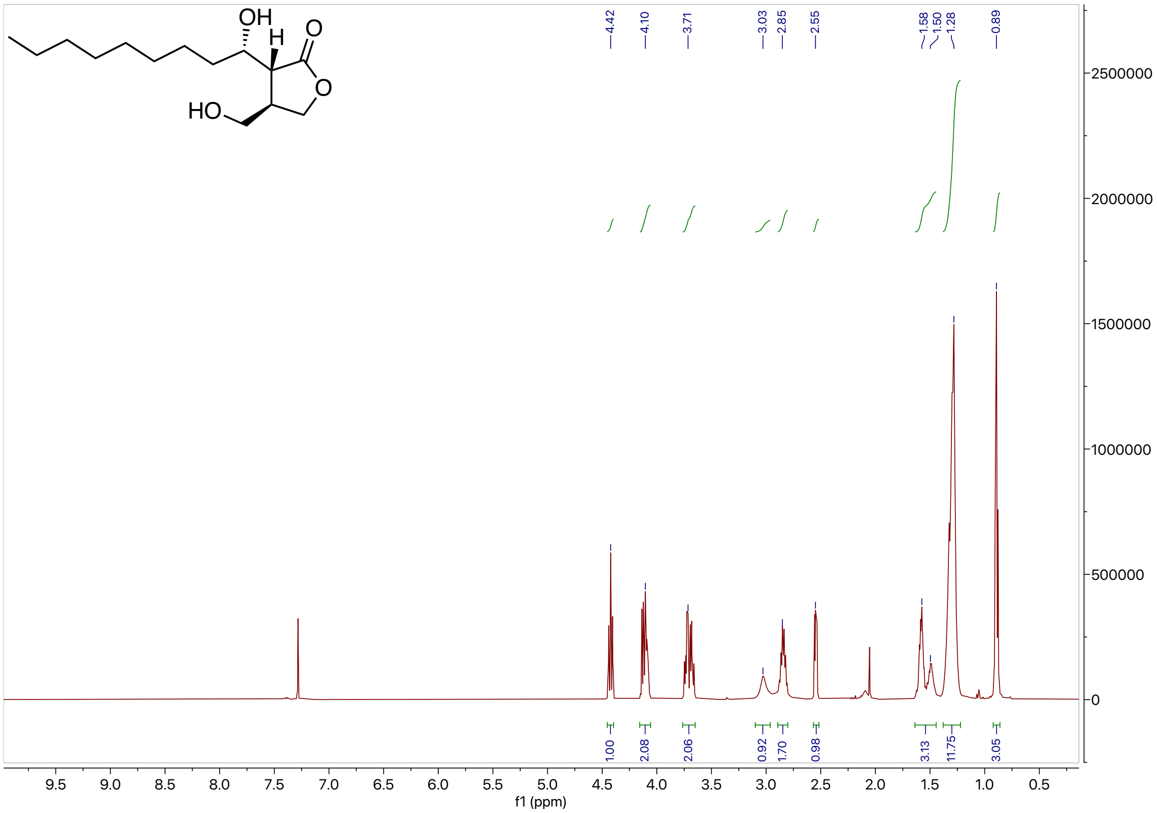

Figure S92. ^{1}H NMR Spectrum of Compound **8h**

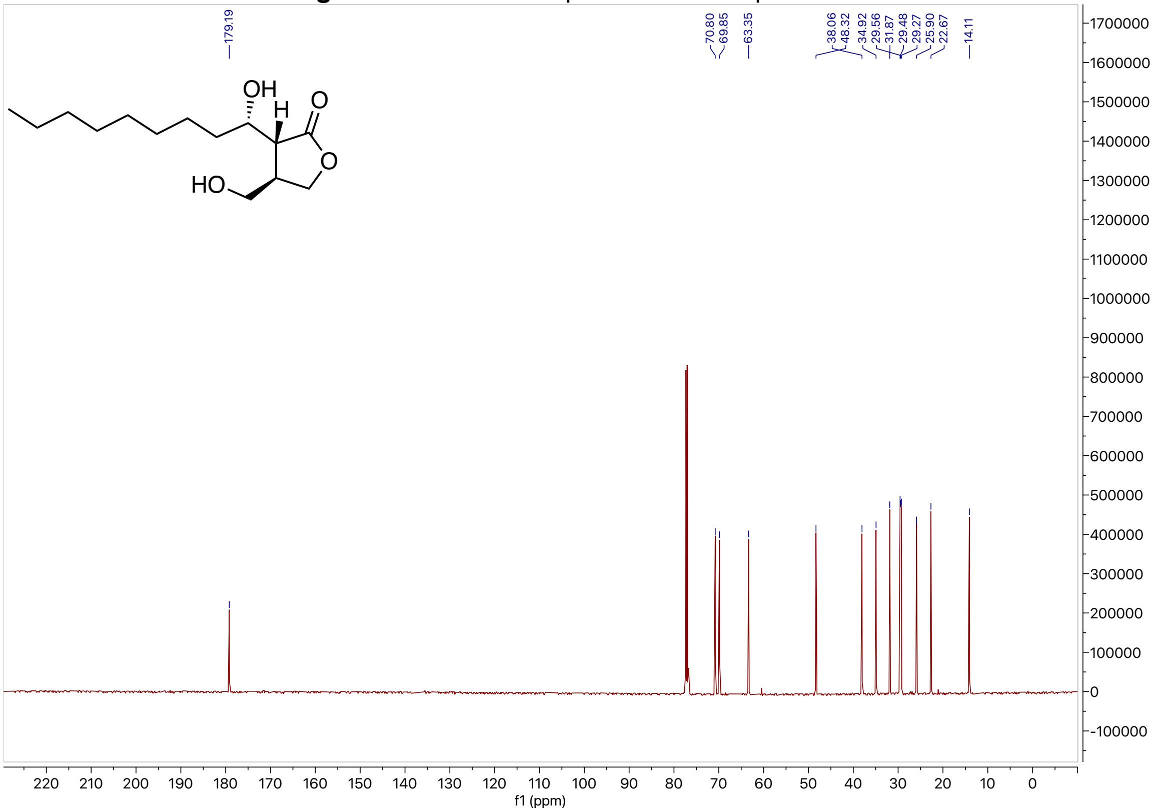

Figure S93. ^{13}C NMR Spectrum of Compound **8h**

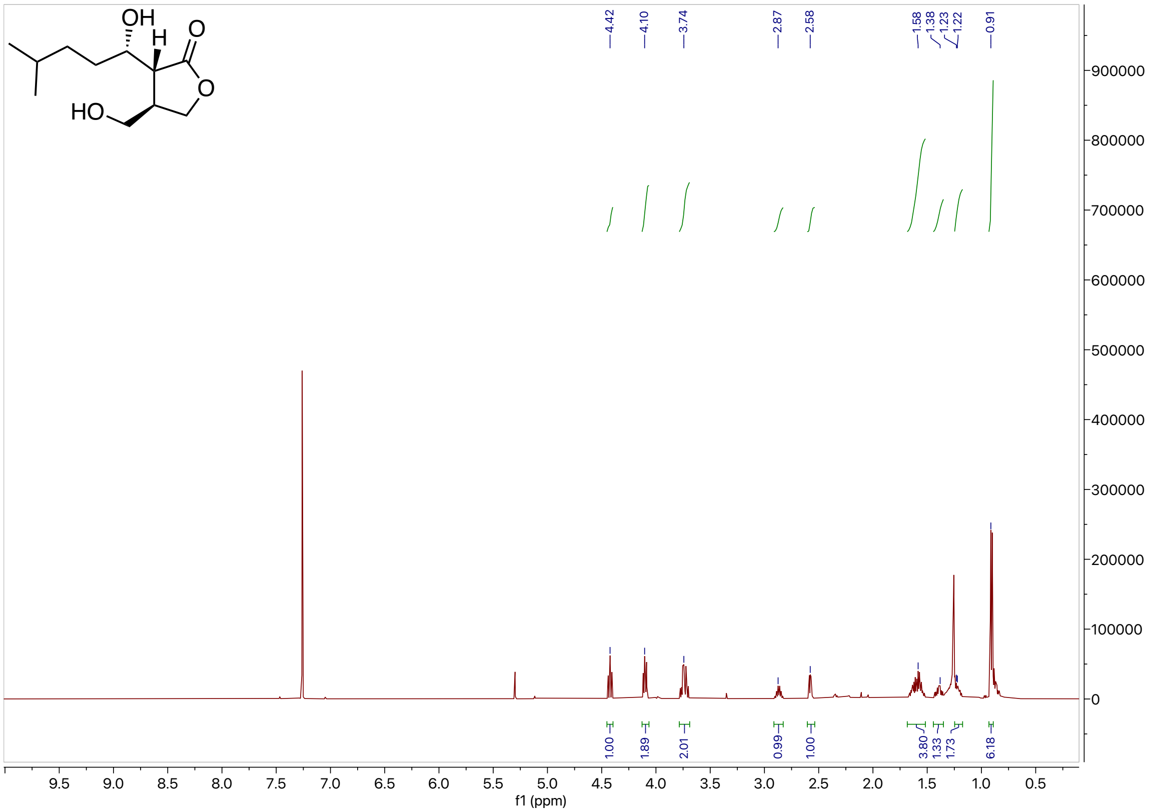

Figure S94. ^{1}H NMR Spectrum of Compound **8i**

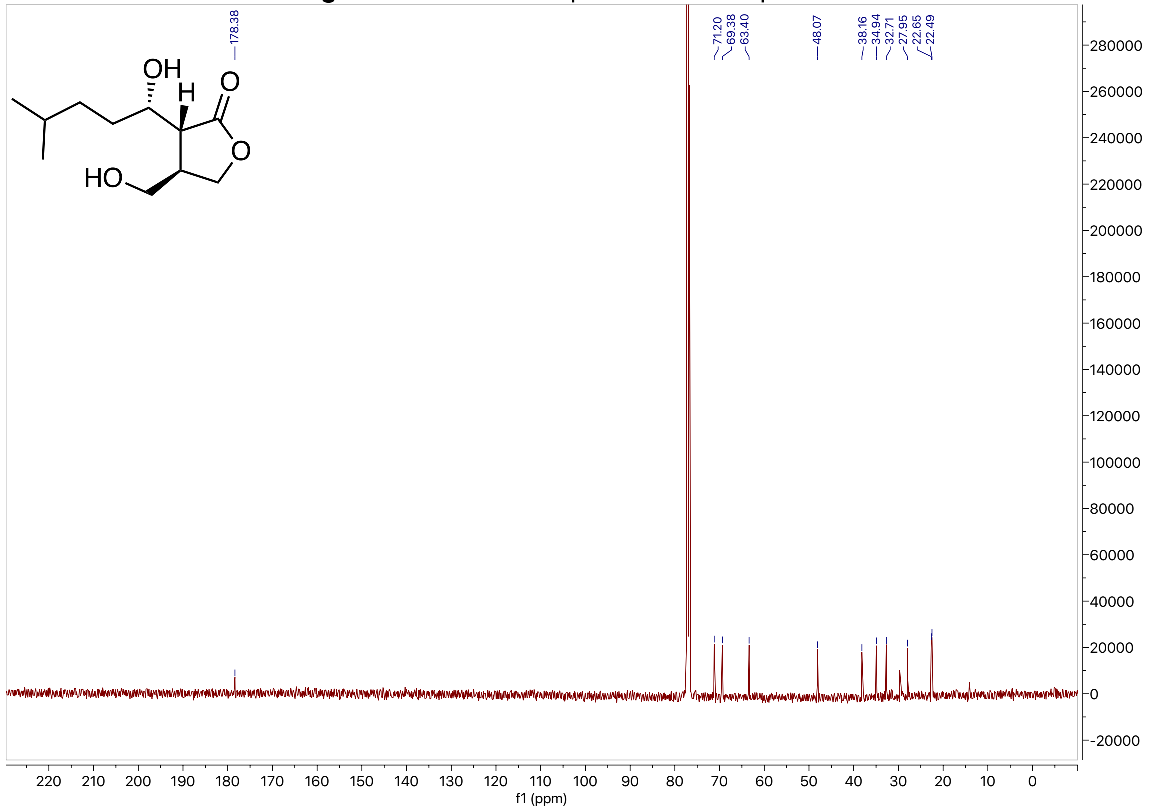

Figure S95. ^{13}C NMR Spectrum of Compound **8i**

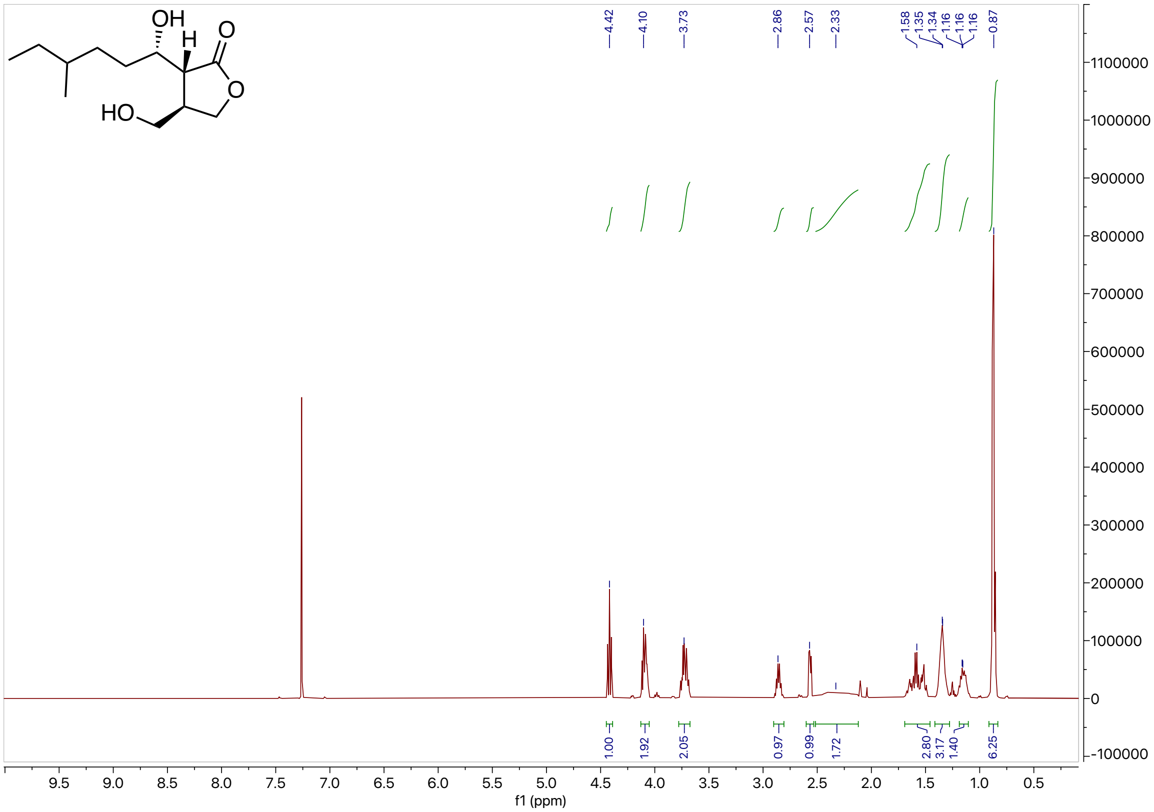

Figure S96. ^{1}H NMR Spectrum of Compound **8j**

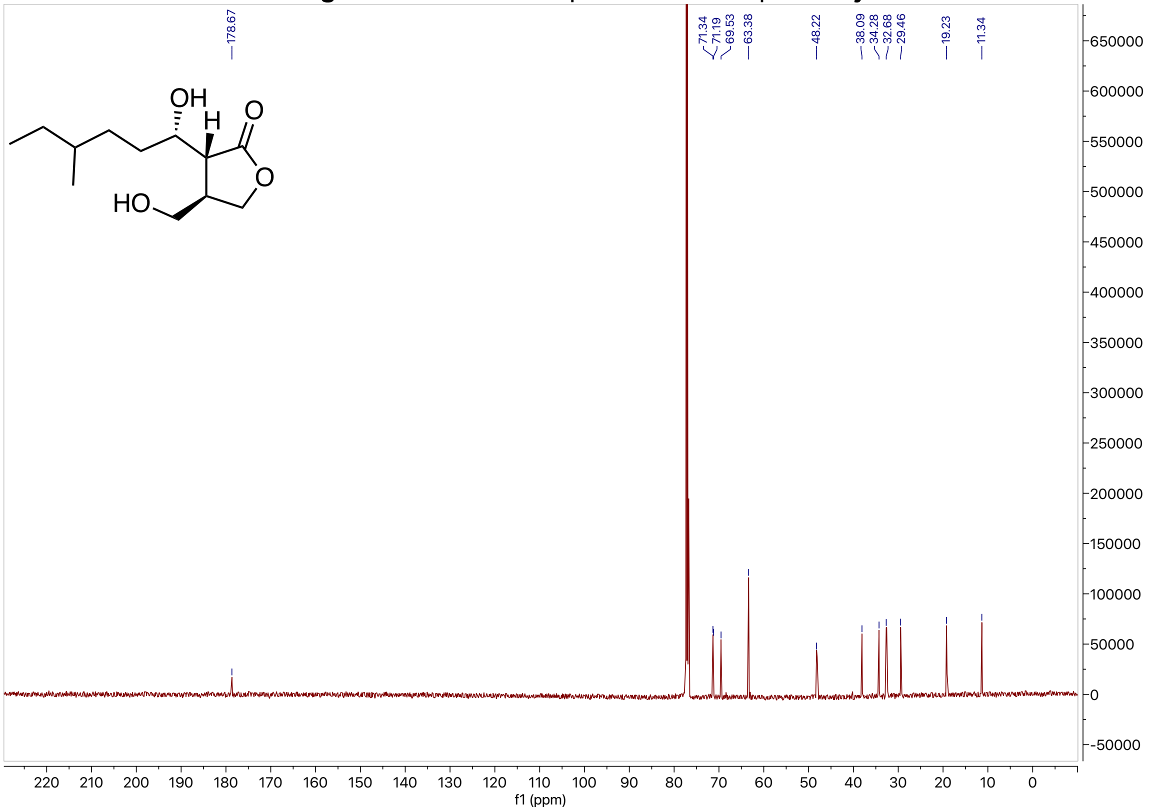

Figure S97. ^{13}C NMR Spectrum of Compound **8j**

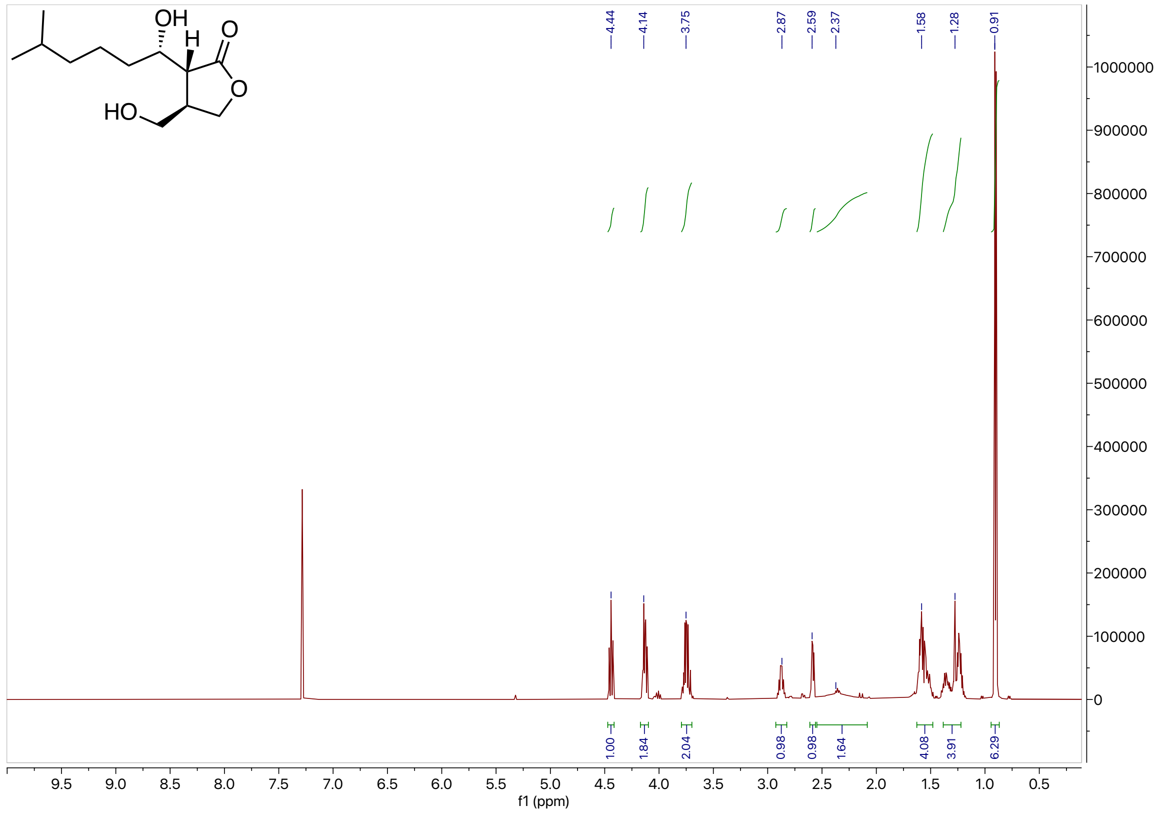

Figure S98. ^{1}H NMR Spectrum of Compound **8k**

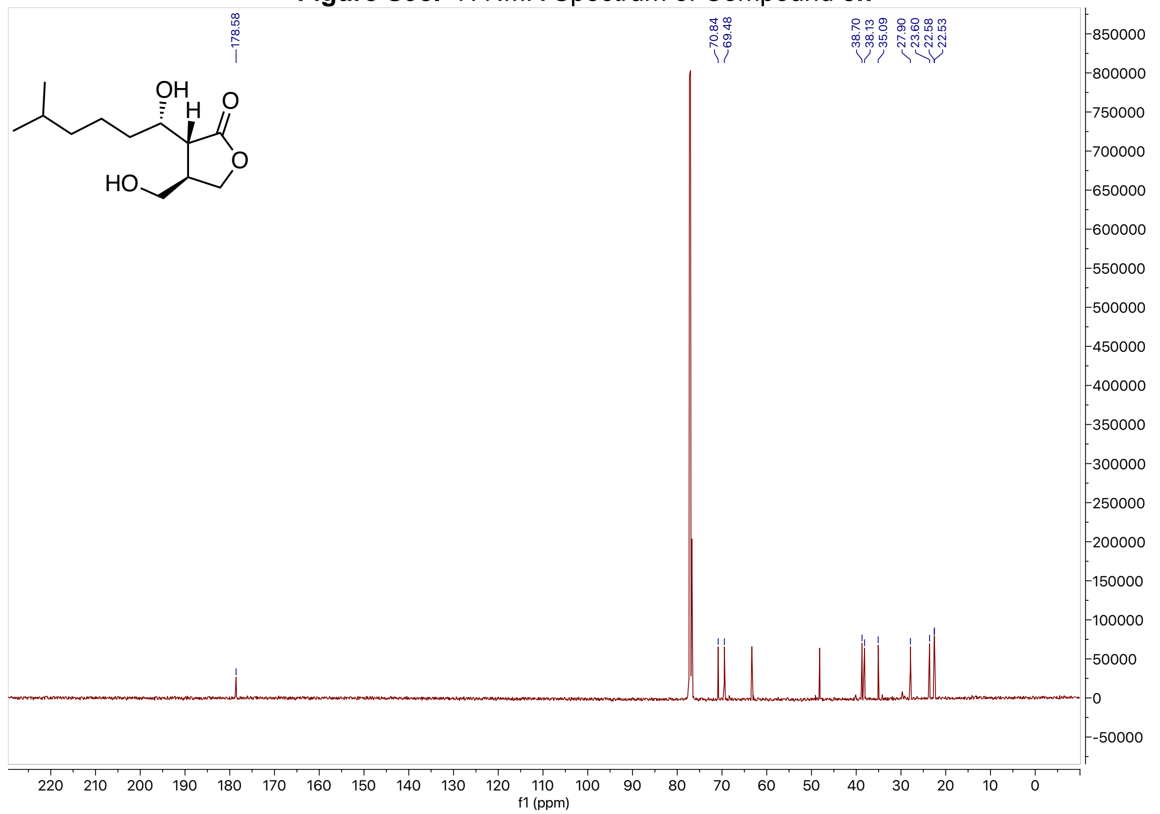

Figure S99. ^{13}C NMR Spectrum of Compound **8k**

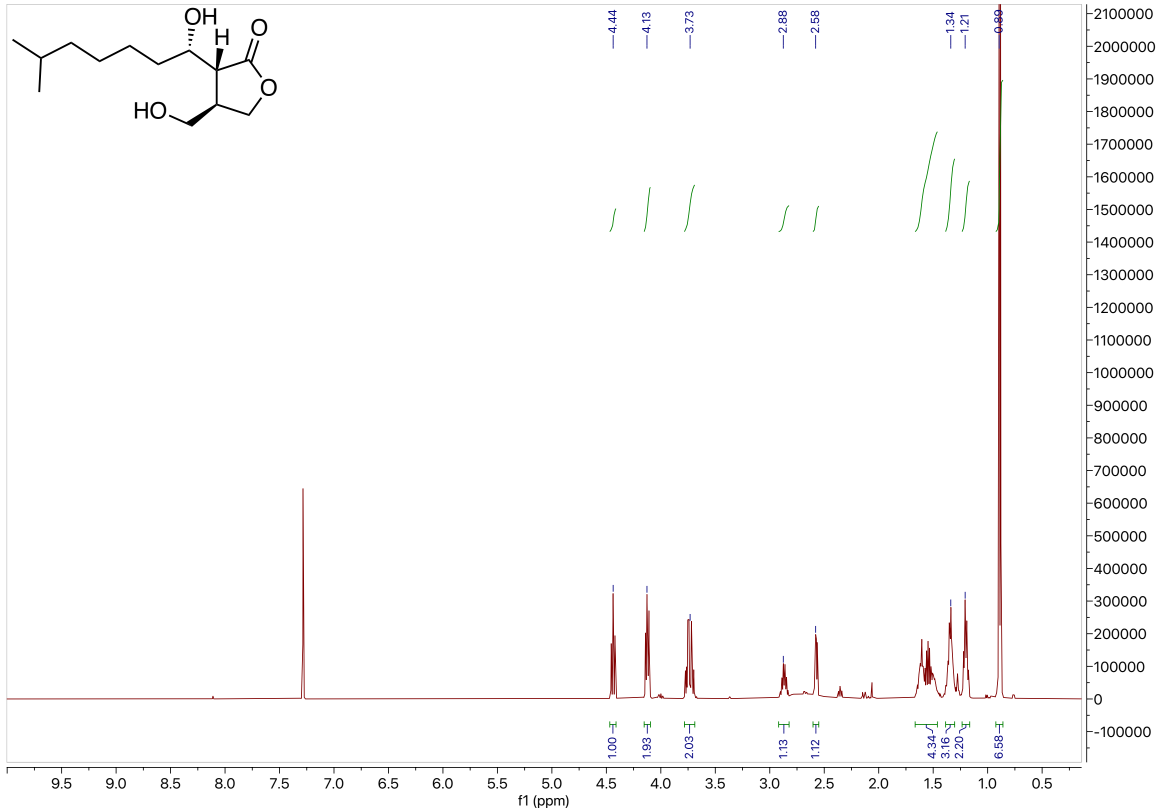

Figure S100. ^{1}H NMR Spectrum of Compound **8l**

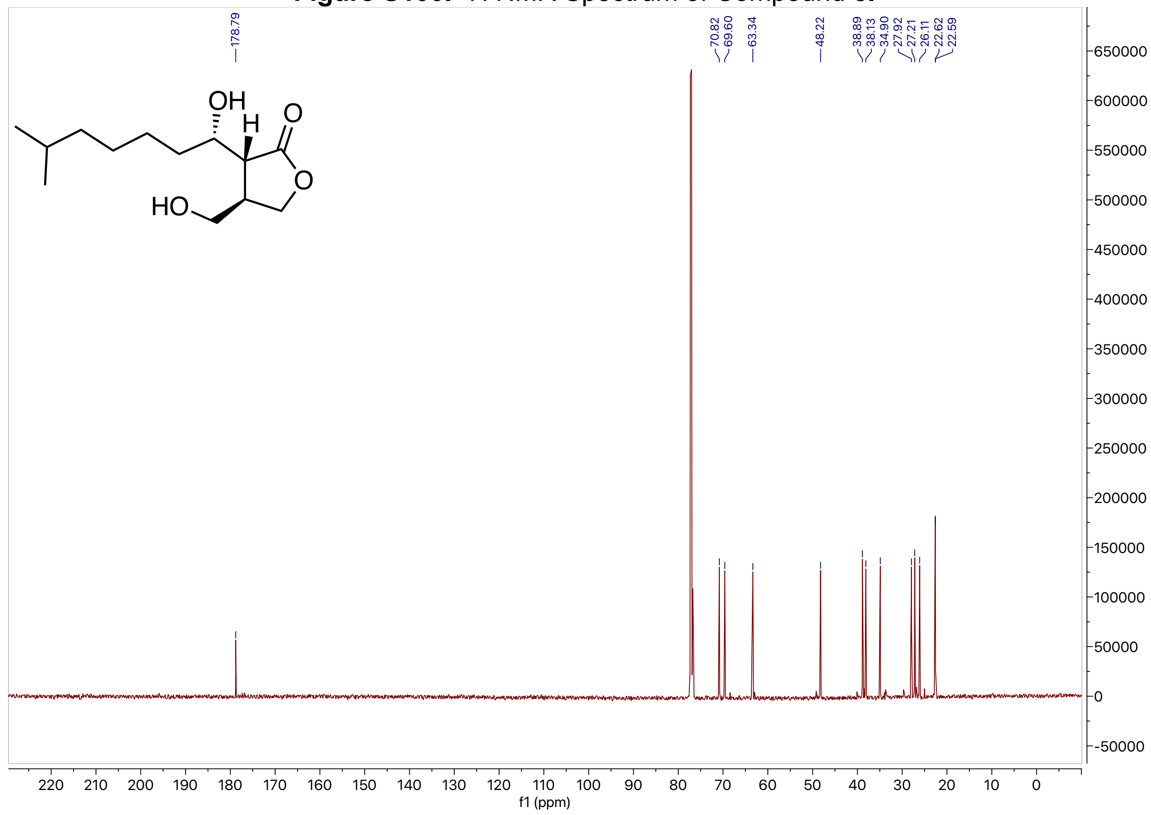

Figure S101. ^{13}C NMR Spectrum of Compound **8l**

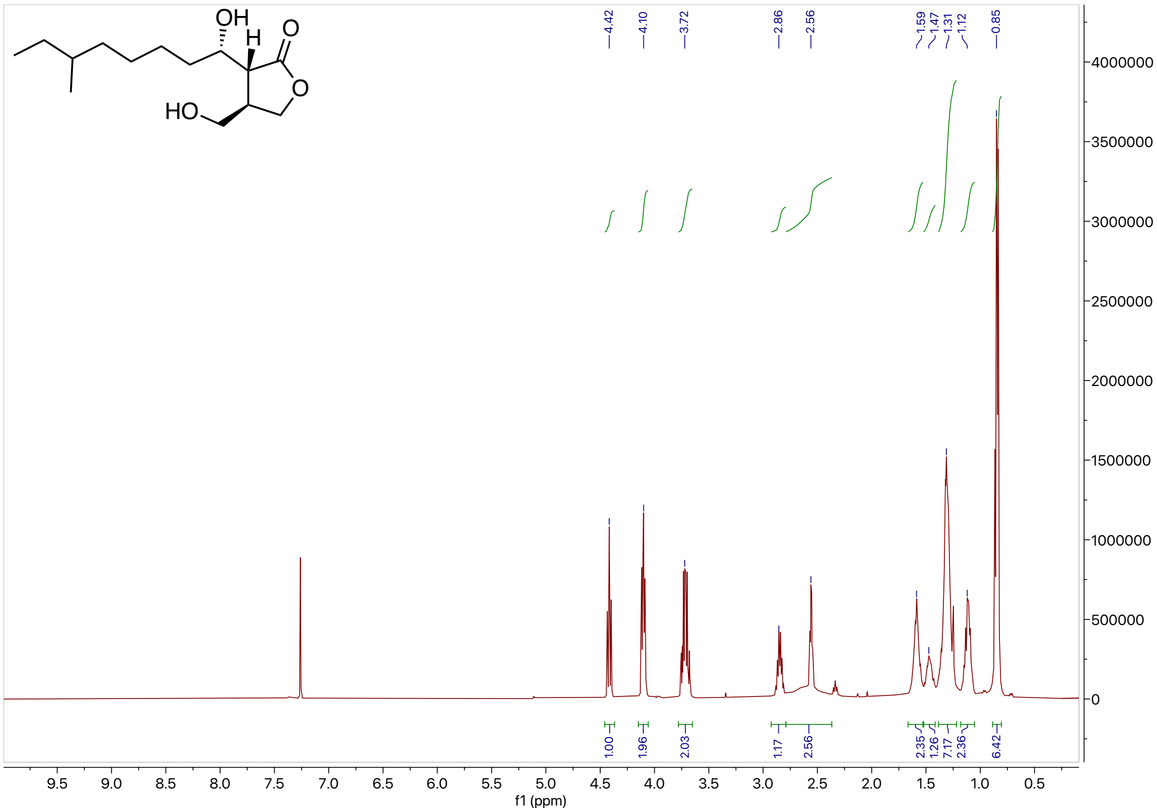

Figure S102. ^{1}H NMR Spectrum of Compound **8m**

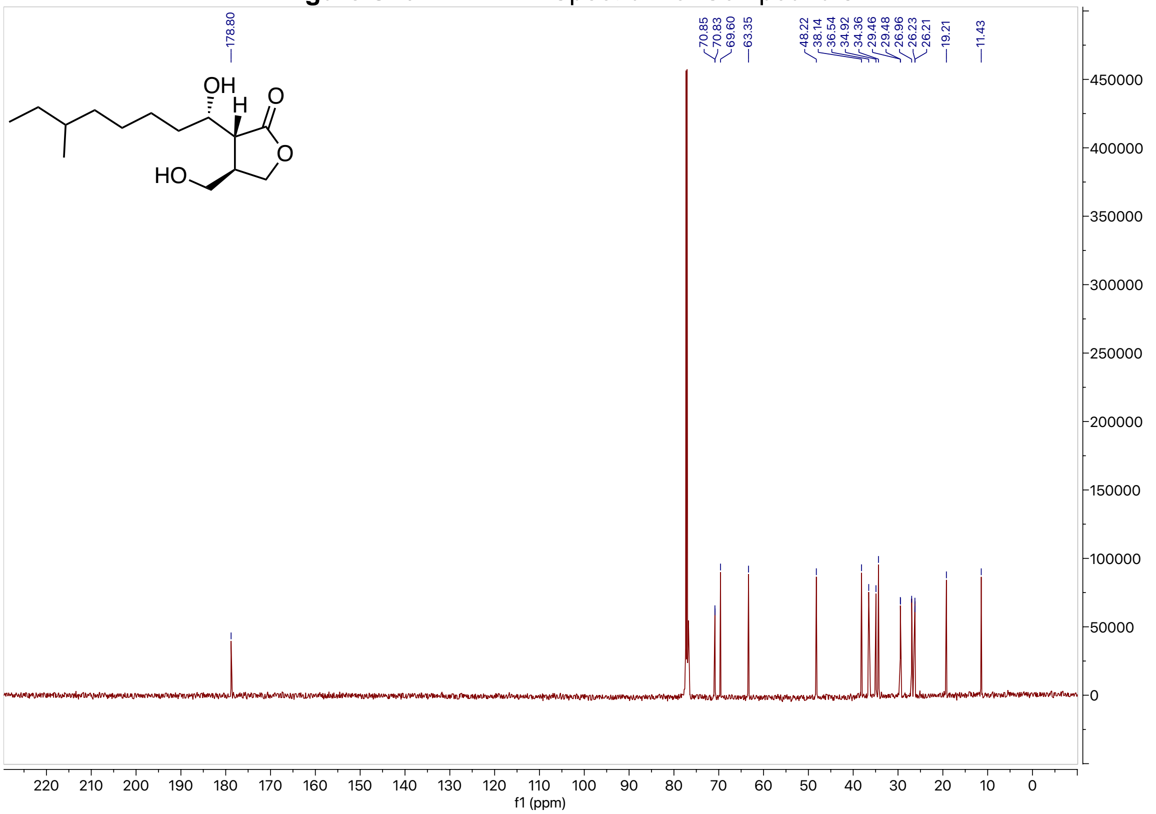

Figure S103. ^{13}C NMR Spectrum of Compound **8m**

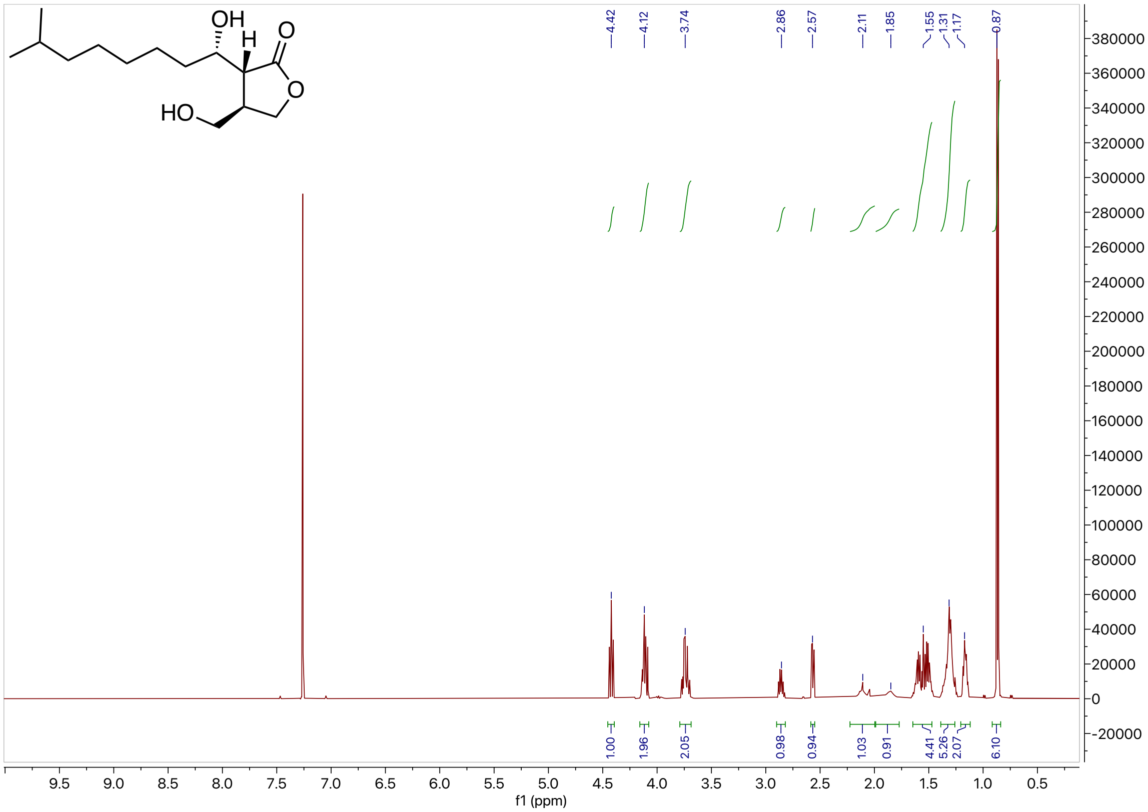

Figure S104. ^{1}H NMR Spectrum of Compound **8n**

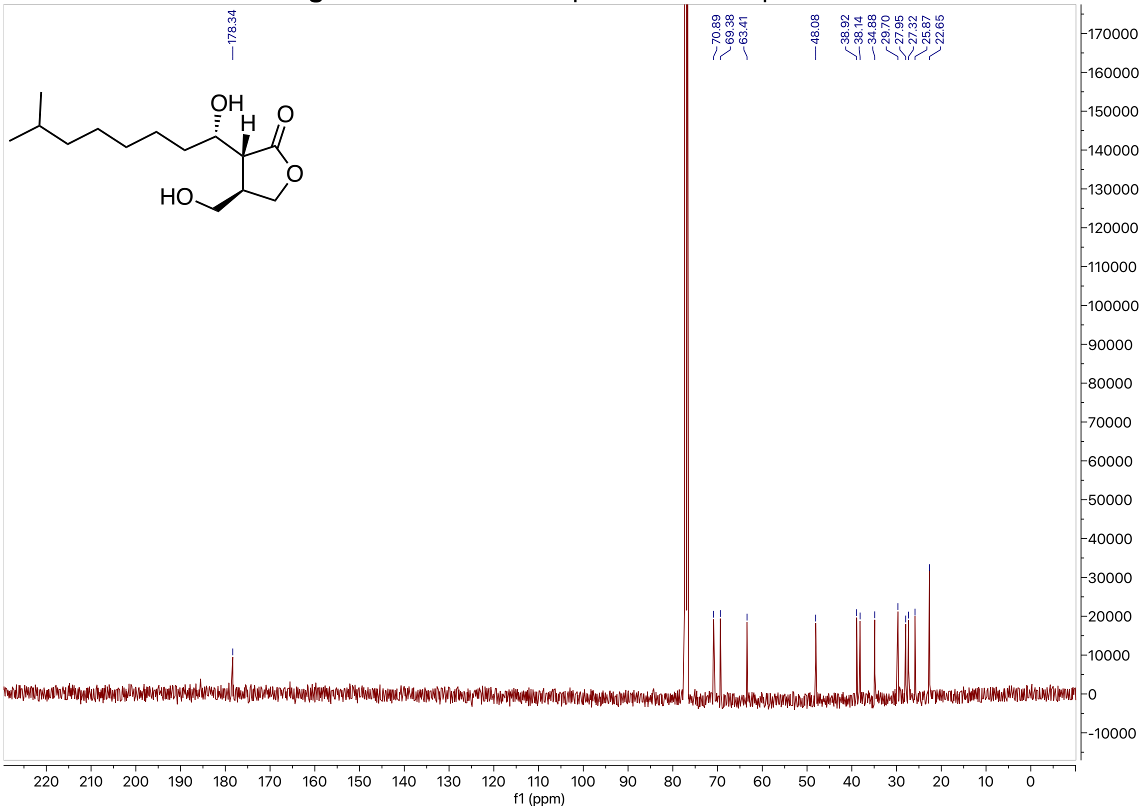

Figure S105. ^{13}C NMR Spectrum of Compound **8n**

Supplementary References

[1] R. P. Shetty, D. Endy, T. F. Knight, *J Biol Eng* **2008**, *2*, 5.

[2] H. Kinoshita, T. Tsuji, H. Ipposhi, T. Nihira, Y. Yamada, *J. Bacteriol.* **1999**, *181*, 5075–5080.

[3] J. Abramson, J. Adler, J. Dunger, R. Evans, T. Green, A. Pritzel, O. Ronneberger, L. Willmore, A. J. Ballard, J. Bambrick, S. W. Bodenstein, D. A. Evans, C.-C. Hung, M. O'Neill, D. Reiman, K. Tunyasuvunakool, Z. Wu, A. Žemgulytė, E. Arvaniti, C. Beattie, O. Bertolli, A. Bridgland, A. Cherepanov, M. Congreve, A. I. Cowen-Rivers, A. Cowie, M. Figurnov, F. B. Fuchs, H. Gladman, R. Jain, Y. A. Khan, C. M. R. Low, K. Perlin, A. Potapenko, P. Savy, S. Singh, A. Stecula, A. Thillaisundaram, C. Tong, S. Yakneen, E. D. Zhong, M. Zielinski, A. Žídek, V. Bapst, P. Kohli, M. Jaderberg, D. Hassabis, J. M. Jumper, *Nature* **2024**, *630*, 493–500.

[4] J. Schröer, P. Welzel, *Tetrahedron* **1994**, *50*, 6839–6858.

[5] U. Grafe, G. Reinhardt, W. Schade, I. Eritt, W. F. Fleck, L. Radics, *Biotechnol Lett* **1983**, *5*, 591–596.

[6] Y. Yamada, K. Sugamura, K. Kondo, H. Okada, M. Yanagimoto, *J Antibiot (Tokyo)* **1987**, *40*, 496–504.

[7] L. E. Wilbanks, H. E. Hennigan, C. D. Martinez-Brokaw, H. Lakkis, S. Thormann, A. S. Eggly, G. Buechel, E. I. Parkinson, *ACS Chem Biol* **2023**, DOI https://doi.org/10.1021/acschembio.3c00241.

[8] K. Ikeuchi, K. Murasawa, H. Yamada, *Synlett* **2019**, *30*, 1308–1312.

[9] J. E. Whiting, J. T. Edward, *Can J Chem* **1971**, *49*, 3799–3806.
